# Importance of Transcript Variants in Transcriptome Analyses

**DOI:** 10.3390/cells13171502

**Published:** 2024-09-08

**Authors:** Kevin Vo, Yashica Sharma, Anohita Paul, Ryan Mohamadi, Amelia Mohamadi, Patrick E. Fields, M. A. Karim Rumi

**Affiliations:** Department of Pathology and Laboratory Medicine, University of Kansas Medical Center, Kansas City, KS 66160, USA; kvo2@hawk.iit.edu (K.V.); yashica2025@gmail.com (Y.S.); apaul04@g.ucla.edu (A.P.); ryanm700@gmail.com (R.M.); amelia.mohamadi@ku.edu (A.M.); pfields@kumc.edu (P.E.F.)

**Keywords:** RNA sequencing, transcript variants, embryonic stem cells, trophoblast stem cells, differential expression of genes, differential expression of transcript variants

## Abstract

RNA sequencing (RNA-Seq) has become a widely adopted technique for studying gene expression. However, conventional RNA-Seq analyses rely on gene expression (GE) values that aggregate all the transcripts produced under a single gene identifier, overlooking the complexity of transcript variants arising from different transcription start sites or alternative splicing. Transcript variants may encode proteins with diverse functional domains, or noncoding RNAs. This study explored the implications of neglecting transcript variants in RNA-Seq analyses. Among the 1334 transcription factor (TF) genes expressed in mouse embryonic stem (ES) or trophoblast stem (TS) cells, 652 were differentially expressed in TS cells based on GE values (365 upregulated and 287 downregulated, ≥absolute 2-fold changes, false discovery rate (FDR) *p*-value ≤ 0.05). The 365 upregulated genes expressed 883 transcript variants. Further transcript expression (TE) based analyses identified only 174 (<20%) of the 883 transcripts to be upregulated. The remaining 709 transcripts were either downregulated or showed no significant changes. Meanwhile, the 287 downregulated genes expressed 856 transcript variants and only 153 (<20%) of the 856 transcripts were downregulated. The other 703 transcripts were either upregulated or showed no significant change. Additionally, the 682 insignificant TF genes (GE values < absolute 2-fold changes and/or FDR p-values > 0.05) between ES and TS cells expressed 2215 transcript variants. These included 477 (>21%) differentially expressed transcripts (276 upregulated and 201 downregulated, ≥absolute 2-fold changes, FDR p-value ≤ 0.05). Hence, GE based RNA-Seq analyses do not represent accurate expression levels due to divergent transcripts expression from the same gene. Our findings show that by including transcript variants in RNA-Seq analyses, we can generate a precise understanding of a gene’s functional and regulatory landscape; ignoring the variants may result in an erroneous interpretation.

## 1. Introduction

Understanding gene expression at the cellular level is crucial for unraveling cell-type-specific functions, identifying biomarkers, and pinpointing genes or pathways for targeted molecular interventions [1]. RNA sequencing (RNA-Seq) has emerged as a powerful tool for comprehensive transcriptome analysis, enabling the identification of lineage-specific gene expression patterns [2,3,4]. Integrating RNA-Seq with techniques such as ATAC-Seq, ChIP-Seq, Cut and Run, Ribo-Seq, and methyl-Seq has provided insights into the intricate interplay between epigenomic modifications and transcriptional regulations [5,6]. Moreover, a detailed examination of the transcriptome offers a window into the gene regulatory mechanisms within a distinct cell type [7]. 

A single gene does not express a single mRNA to encode a single protein [8]. Commonly, multiple mRNAs that are transcribed encode different proteins or noncoding RNAs [8]. Alternative transcription start sites (ATSS) can result in the expression of more than one transcript from a single gene [9]. Alternative transcription start sites occur due to alternative proximal promoter use as well as the availability of alternative transcriptional regulators in a particular cell type [10,11]. However, alternative splicing is another common mechanism underlying the generation of multiple mRNA variants from a single initial transcript [12]. RNA editing may further expand the repertoire of transcript variants [13]. Although the transcript variants are translated into peptides using the same open reading frame, they can encode a variety of proteins with different lengths and functional domains [14]. Some alternative transcripts do not encode any proteins and may act as long noncoding RNAs or other regulatory RNAs [15]. Thus, alternative transcripts, some of which encode noncoding RNAs, may play pivotal roles in lineage-specific divergent cellular functions. 

Despite the functional intricacies of transcript variants, conventional gene expression analyses typically overlook the diversity of transcripts. Current RNA-Seq methodologies often quantify gene expression (GE) values in reads per kilobase million (RPKM) or transcripts per million (TPM), aggregating all transcript counts under a single mRNA identifier without distinguishing between full-length transcripts and their variants, irrespective of their protein-coding potential [16,17]. However, RNA-Seq analyses can generate quantitative data regarding transcript variants’ expressions (TE values). While transcript expression (TE) values can be concurrently calculated, GE values predominantly drive the identification of differentially expressed genes across experimental conditions, largely due to analytical complexities and validation challenges hindering the widespread adoption of TE analyses [18]. Hence, to understand the biological function of the transcript variants in cells, TE-based analysis is required to elucidate the precise mechanisms.

This study aims to draw the attention of researchers in the field of transcriptomic analyses to two important issues. First, it is biologically inaccurate to consider the expression of a single transcript from a specific gene for differential expression analyses. Second, it can be misleading to conclude that similar expression trends occur in all the transcript variants expressed from a single gene. Therefore, we have evaluated the limitations of GE-based RNA-Seq analyses without considering the TE values of transcript variants. We have observed that while one transcript variant of a gene is upregulated, another transcript variant can be downregulated, which, ultimately, skews the results or masks the actual expression pattern. Our results indicate that GE-based RNA-Seq analyses incorrectly represented over 80% of the TE-based analyses. 

## 2. Materials and Methods

### 2.1. Experimental Model

**Figure 1:**
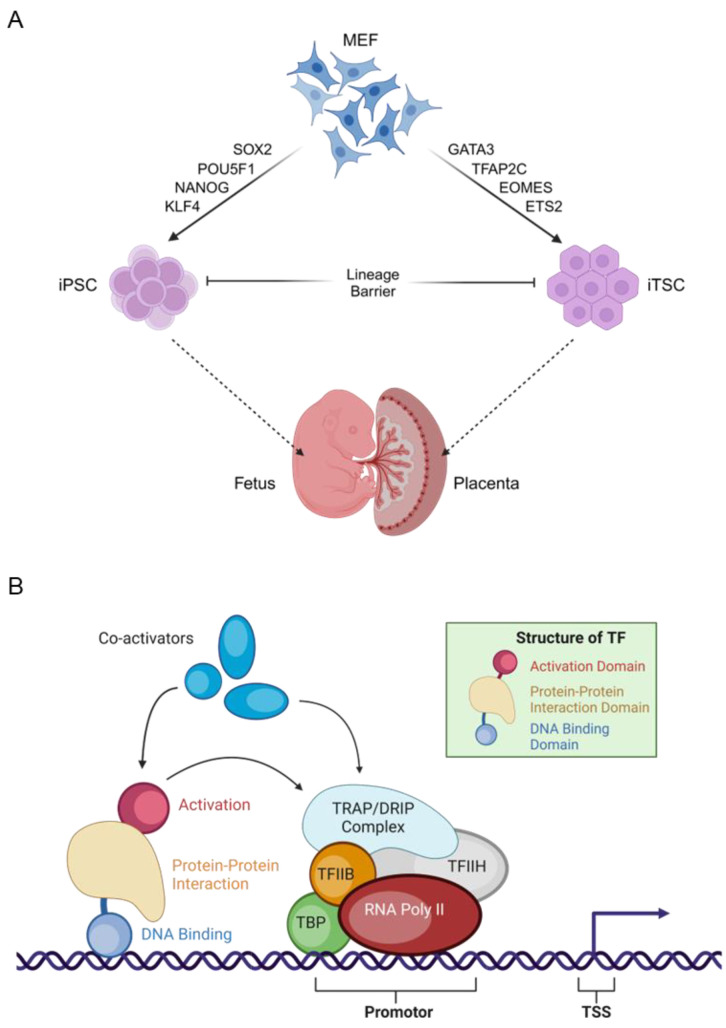
Transcription factors and early embryonic stem cells. The schematics explain the reasons for choosing TFs for this study (**A**). Transcript variants encoding distinct domains are easily understandable in TFs, and the role of TFs in determining cell fate is well known (**B**). The rightmost arrow indicates the start of gene transcription. MEF, mouse embryonic fibroblast; iPSC, induced pluripotent stem cells; iTSC, induced trophoblast stem cells; TF, transcription factor; RNA Pol II, RNA polymerase II; TSS, transcription start site.

We have used RNA sequencing data of two early embryonic stem cell lines, embryonic stem (ES) cells and trophoblast stem (TS) cells, and focused on the expression of transcription factors (TFs). Differential expression of lineage-specific TFs are characteristic determinants of ES and TS cell lineages. Ectopic expression of selective lineage-specific TFs can reprogram somatic cells into ES or TS cells [19,20] (Figure 1A). Moreover, TFs are appropriate for defining the role of transcript variants due to their well-defined functional domains [DNA binding domains (DBDs), transactivation domains (TADs), and signaling sensing domains (SSDs)] [21] (Figure 1B). Thus, transcript variants of the same TF gene may encode proteins carrying different DBDs, TADs, or SSDs that can be easily determined. We have systematically analyzed the differential expression of TFs between the ES and TS cells to understand the limitations of transcriptome analyses without considering the transcript variants. 

### 2.2. RNA Sequencing Data

This study included RNA-Seq data from mouse ES cells (*n* = 3 libraries) and mouse TS cells (*n* = 3 libraries). Mouse TS cell data were generated in our laboratory and have been submitted to the Sequencing Read Archive (PRJNA1131096; SRA, NCBI). The ES cell data were downloaded from the NCBI’s Gene Expression Omnibus (GEO) (SRR24044798, SRR24044809, SRR24044810) [22]. 

Mouse TS cells were maintained in feeder-free stem conditions [23]. TS cells were cultured for 48 h, and total RNA was extracted using TRI reagent (Millipore-Sigma, St. Louis, MO, USA). From each sample, 500 ng of total RNA (RIN value > 9) was used for the sequencing library preparation using a TruSeq Stranded mRNA kit (Illumina, San Diego, CA, USA). The cDNA libraries were evaluated for quality at the KUMC Genomics Core and sequenced on an Illumina HiSeq X sequencer at Novogene Corporation (Sacramento, CA, USA). 

### 2.3. RNA Sequencing Analysis 

RNA-Seq data were analyzed using CLC Genomics Workbench 24 (Qiagen Bioinformatics, Redwood City, CA, USA). The software has Linux, Macintosh, and Windows versions; we used the Windows version to analyze the RNA-Seq data. CLC Genomics Workbench uses the expectation–maximization (EM) estimation algorithm to categorize and assign annotated transcripts to the transcript variants within the reference genome, gene, and mRNA. All clean reads were obtained by removing low-quality reads and trimming the adapter sequences. The high-quality reads were aligned to the Mus musculus reference genome (GRCm39), gene (GRCm39.111_Gene), and mRNA sequences (GRCm39.111_mRNA) using the default parameters: (a) maximum number of allowable mismatches was 2; (b) minimum length and similarity fraction was set at 0.8; and (c) the minimum number of hits per read was 10. The expression values of individual genes (GE) or transcript variants (TE) in ES and TS cells were measured in TPM [24,25,26]. The threshold *p*-value was determined according to the false discovery rate (FDR). Differentially expressed genes were determined if the absolute fold change in expression was 2 with an FDR *p*-value of ≤ 0.05. 

Expression of 16,052 to 16,510 genes was detected in the ES- or TS-cell-derived RNA-Seq samples. We selectively analyzed the 1374 mouse TFs that were curated by the Gifford lab (https://cgs.csail.mit.edu/ReprogrammingRecovery/mouse_tf_list.html) from a list of human TFs [27]. Notably, about 90% of the TF genes in ES and TS cells expressed more than two transcript variants based on GRCm39.111_mRNA analyses. New tracks containing only the TFs were generated from each RNA-Seq data file containing GE or TE values, which were used in subsequent analyses. The threshold *p*-values were determined according to the false discovery rate (FDR) to identify the differentially expressed genes or transcript variants between ES and TS cells. A gene or a transcript variant was considered differentially expressed if the absolute fold change was ≥2 and the FDR *p*-value was ≤ 0.05 [24,25,26].

### 2.4. Analysis of the Transcript Variants

We analyzed the differential expression of genes using the RNA-Seq files containing GE values. The differentially expressed genes were divided into three groups: upregulated (≥2-fold changes and FDR *p* ≤ 0.05), downregulated (≤−2-fold changes and FDR *p* ≤ 0.05), and insignificant (either < absolute 2-fold changes and/or FDR *p* > 0.05). The transcript variants encoded by the upregulated, downregulated, or insignificant group of genes were further analyzed to identify the differentially expressed ones between mouse ES and TS cells. Differential expressions of the transcript variants were analyzed using the RNA-Seq files containing TE values. These analyses identified the differentially upregulated (≥2-fold changes and FDR *p* ≤ 0.05), downregulated (≤−2-fold changes and FDR *p* ≤ 0.05), and insignificant (either < absolute 2-fold changes or FDR *p* > 0.05) group of transcript variants. 

### 2.5. Statistical Analyses

For RNA Seq, each study group contained three library samples. In CLC Genomics Workbench 24, the ‘differential expression for RNA-Seq tool’ performs multi-factorial statistics on a set of expression tracks based on a negative binomial generalized linear model (GLM). The final GLM fit and dispersion estimate calculates the total likelihood of the model given the data and the uncertainty of each fitted coefficient [28]. Two statistical tests, the Wald and the likelihood ratio tests, use one of these values. The across-group (ANOVA-like) comparison uses the likelihood ratio test.

## 3. Results

### 3.1. Lineage-Specific Expression of Transcription Factors

**Figure 2:**
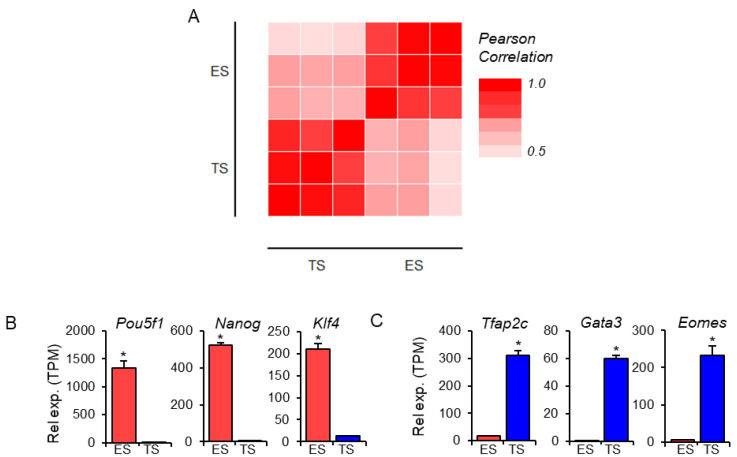
Quality and validity of the RNA-Seq data obtained from mouse ES cells and TS cells. (**A**) A matrix shows the Pearson correlation of 1365 transcription factors (TFs) expressed in three ES and three TS samples. ES-specific abundant expression of characteristic TFs (*Pou5f1*, *Nanog*, and *Klf4*) that differentiate them from TS cells (**B**) and TS-specific abundant expression of TFs (*Tfap2c*, *Gata3*, and *Eomes*) that distinguish those from ES cells (**C**) indicate the RNA-Seq data quality and validity. Data represent mean TPM ± SE, * indicates *p* < 0.05. Rel exp., relative expression; TPM, transcript per million. Rel exp., relative expression.

Expression of the TFs in mouse ES cells and TS cells was analyzed using RNA-Seq data. Differential expression of TF genes between ES and TS cells was evident in the heat map (Figure 2A). Based on the Pearson correlation matrix of the TFs, there was a high positive relation among the three ES samples and among the three TS samples (Figure 2A). The validity of ES and TS lineage identity was confirmed based on the expression of stem cell markers (*Pou5f1*, *Nanog*, and *Klf4* for ES; *Tfap2C*, *Gata3*, and *Eomes* for TS) [29,30,31]. High levels of *Pou5f1*, *Nanog*, and *Klf4* were expressed in mouse ES cells but were very low in TS cells (Figure 2B). In contrast, *Tfap2c*, *Gata3*, and *Eomes* expressions were very high in mouse TS cells but low in ES cells (Figure 2C).

### 3.2. Differential Expression of the Transcription Factor Genes and Transcript Variants

**Figure 3:**
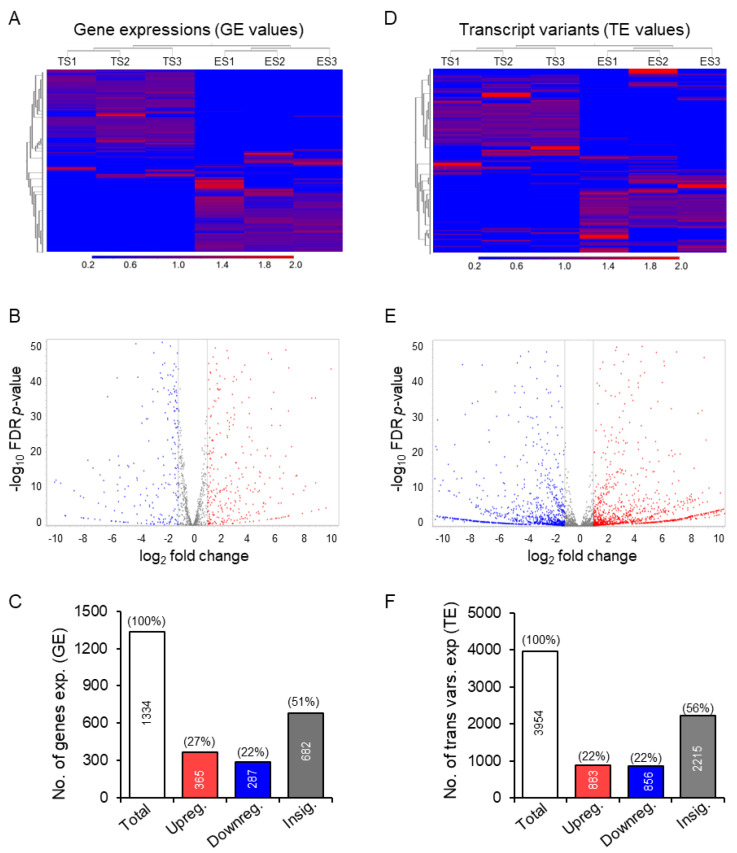
Differential expression of genes and transcript variants in mouse TS cells compared to ES cells. Heat maps, volcano plots, and bar graphs show that ~49% of the genes were differentially expressed (27% upregulated and 22% downregulated) in TS cells (**A**–**C**). Similarly, 44% of the transcript variants encoded by the TF genes were differentially expressed (22% upregulated and 22% downregulated) in TS cells. A 5% reduction in upregulated transcript variants was associated with increased variants in the insignificant group (**D**–**F**). No., number; exp., expression; vars., variants.

Of the 1374 TFs, 1334 were expressed in mouse ES or TS cells. TS cells showed differential expression of 652 TF genes compared to ES cells (365 upregulated and 287 downregulated; ≥absolute 2-fold changes, FDR *p*-value ≤ 0.05) (Figure 3A–C). The differential expressions of the GE values in TS cells are evident in heat maps (Figure 3A), volcano plots (Figure 3B), and bar graphs (Figure 3C). The 1334 TF genes expressed 3954 transcript variants in mouse TS or ES cells (Figure 3D–F). A total of 1739 of the 3954 transcript variants were differentially expressed in TS cells (883 upregulated and 856 downregulated; ≥absolute 2-fold changes, FDR *p*-value ≤ 0.05) (Figure 3D–F). The differential expressions of the TE values in TS cells are shown in heat maps (Figure 3D), volcano plots (Figure 3E), and bar graphs (Figure 3F).

### 3.3. Discrepancy between Gene Expression and Transcript Variants 

**Figure 4:**
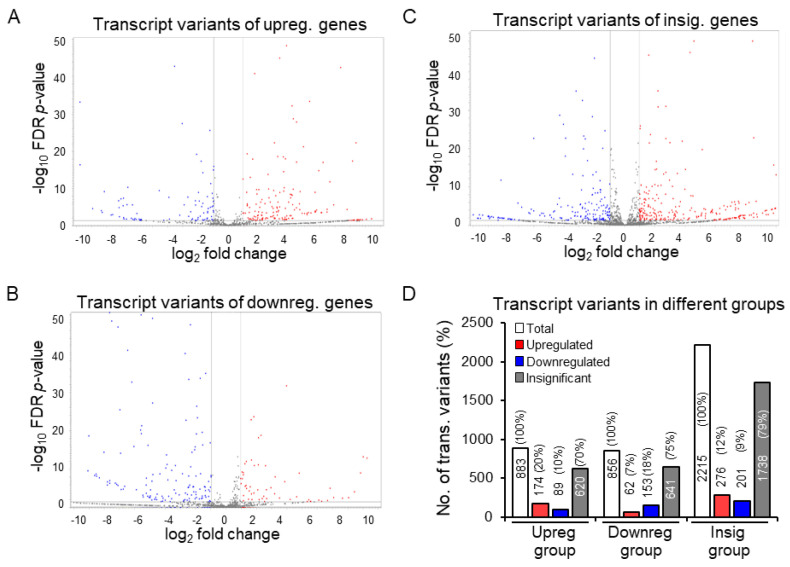
Discordant differential expression of the transcript variants expressed in mouse TS cells. Volcano plots show the differential expression of transcript variants corresponding to upregulated (**A**), downregulated (**B**), and insignificant genes (**C**). Of transcript variants of the upregulated genes, ~80% were either downregulated or insignificant (**D**). Similarly, ~82% of transcript variants of the downregulated genes were either upregulated or insignificant (**D**). In addition, ~21% of transcript variants of the insignificant genes were differentially expressed (**D**). Upreg., upregulated; downreg., downregulated; insig., insignificant; no., number; trans., transcript.

**Figure 5:**
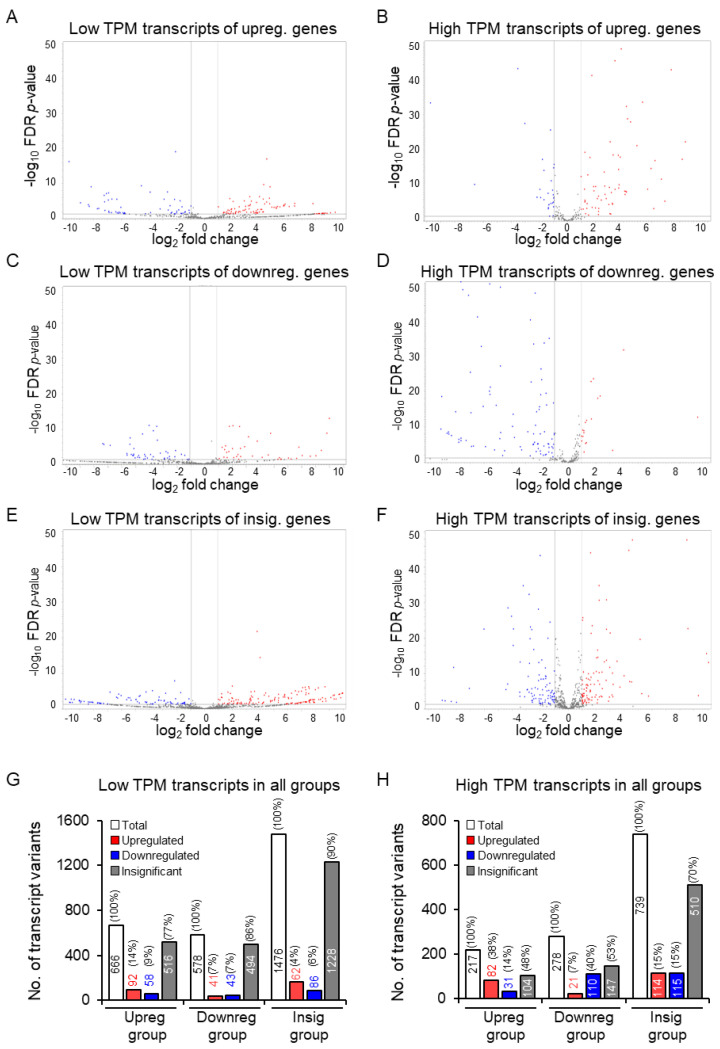
Differential expressions of the low-copy- and high-copy number transcript variants expressed in mouse ES or TS cells. Volcano plots showed discordant results among the low-copy-number (<5 TPM) (**A**,**C**,**E**) as well as the high-copy-number (**B**,**D**,**F**) transcript variants expressed by upregulated (**A**,**B**), downregulated (**C**,**D**), or insignificant (**E**,**F**) genes. While the low-copy-number transcripts of the upregulated genes showed discordant results in 86%, it was only 62% among the high-copy-number transcripts (**G**,**H**). Similarly, 93% of the low-copy-number transcripts encoded by downregulated genes were discordant, and only 60% of the high-copy-number genes were discordant (**G**,**H**). Remarkably only 10% of transcript variants of insignificant genes were differentially expressed, whereas it was 30% for the high-copy-number genes. Upreg., upregulated; downreg., downregulated; insig., insignificant; no., number.

Despite the overall similar differential expression of genes (based on GE values) and transcript variants (based on TE values) (Figure 3C,F), further analyses revealed a remarkable discrepancy between GE- and TE-based analyses (Figure 4 and Figure 5). The 365 upregulated genes in TS cells expressed 883 transcript variants. Of those 883 transcript variants, only 174 showed significant upregulation (≥2-fold upregulation, FDR *p*-values ≤ 0.05). The remaining 89 transcript variants were significantly downregulated (≤−2-fold downregulation, FDR *p*-values ≤ 0.05), and 620 showed insignificant differences based on TE values in TS cells (Figure 4A,D). The 287 downregulated genes expressed 856 transcript variants, of which only 153 were significantly downregulated (≤−2-fold downregulation, FDR *p*-values ≤ 0.05). The remaining 62 transcript variants were upregulated (≥2-fold upregulation, FDR *p*-values ≤ 0.05), and 641 showed insignificant differences based on TE values (Figure 4B,D). The 682 genes with no significant differential expression based on GE values (either < absolute 2-fold changes or FDR *p* > 0.05) contained 2215 transcript variants. Of those, 276 transcripts showed significant upregulation (≥2-fold upregulation, FDR *p*-values ≤ 0.05), and 201 showed significant downregulation (≤−2-fold downregulation, FDR *p*-values ≤ 0.05) (Figure 4C,D). We then further compared the transcript variants compressed to their respective gene groups (Supplementary Figure S1). We detected that about ~40% of the transcript variants overlapped in all groups, indicating that those genes encode transcript variants that can be either upregulated, downregulated, or insignificant despite their original gene group.

### 3.4. Increased Discrepancy among the Low-Abundance Transcript Variants

We further analyzed the transcript variants among the differentially expressed genes according to their abundance in mouse TS or ES cells (Figure 5A–H). The low-abundance transcript variants (TPM < 5 TE values) showed greater discrepancy compared to the moderately high-abundance transcripts (TPM ≥ 5 TE values) (Figure 5A–H). The 365 upregulated genes expressed 883 transcript variants; 666 were low-abundance, and 217 were moderate- to high-abundance (Figure 5G,H). The 287 downregulated genes expressed 856 transcript variants, 578 were low-abundance, and 278 were high-abundance (Figure 5G,H). The 682 insignificant genes included 2215 transcript variants, of which 1476 were low-abundance (TPM < 5 TE values), and 739 were high-abundance (TPM ≥ 5 TE values) (Figure 5G,H).

We observed that ~86% of the low-copy-number transcripts of upregulated genes showed discordant results (Figure 5A,G,H). In contrast, ~62% of the high-copy-number transcripts of upregulated genes showed discrepant results (Figure 5B,G,H). Similarly, 93% of the low-copy-number transcripts and 60% of the high-copy-number transcripts that were expressed by the downregulated genes were discrepant. (Figure 5C,D,G,H). Among the low-abundance transcript variants expressed by the insignificant genes, only ~10% showed differential expression, whereas it was ~30% among the high-copy-number transcript variants (Figure 5E–H). We have included detailed lists of the discrepant transcript variants and their biotypes in Supplemental Tables S1–S3. These tables may give an idea of how the variant interacts within the cell, but to find the function relevance of each variant requires experimentation with said variant [32]. This is unfortunately beyond the scope of this study, and due to the novelty of transcript variant analysis, a connection to proteomics (e.g., protein isoforms) was not available. Without proteomics, it is impossible to predict biological function accurately. 

### 3.5. The Basis of Discrepancy between Gene Expression and Transcript Variant Analyses

**Figure 6:**
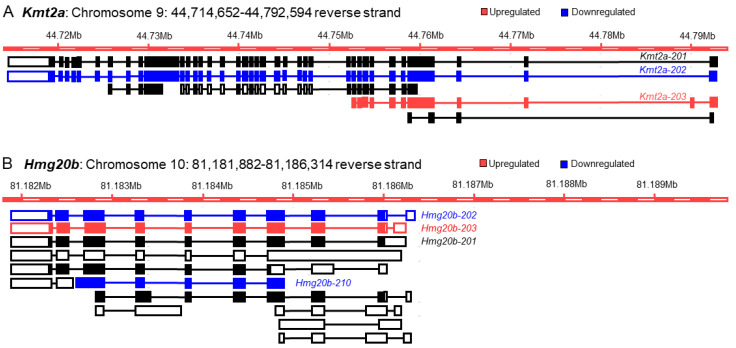
Impact of differentially expressed transcript variants on the upregulated genes. GE-based expression analyses identified both *Kmt2a* and *Hmg20b* as upregulated genes in mouse TS cells. We identified that transcript variant *Kmt2a-202* is significantly downregulated and expresses a full-length functional protein (**A**). However, this downregulation is masked by relatively higher upregulation of another transcript variant of *Kmt2a* (*Kmt2a-203*), which expresses a truncated protein (**A**). We observed the downregulation of *Hmg20b-202*, which encodes a full-length protein, and *Hmg20b-210*, which encodes a truncated protein. Downregulation of the two transcript variants of *Hmg20b* remains unknown due to a higher upregulation of *Hmg20b-203* that encodes a full-length protein (**B**).

**Figure 7:**
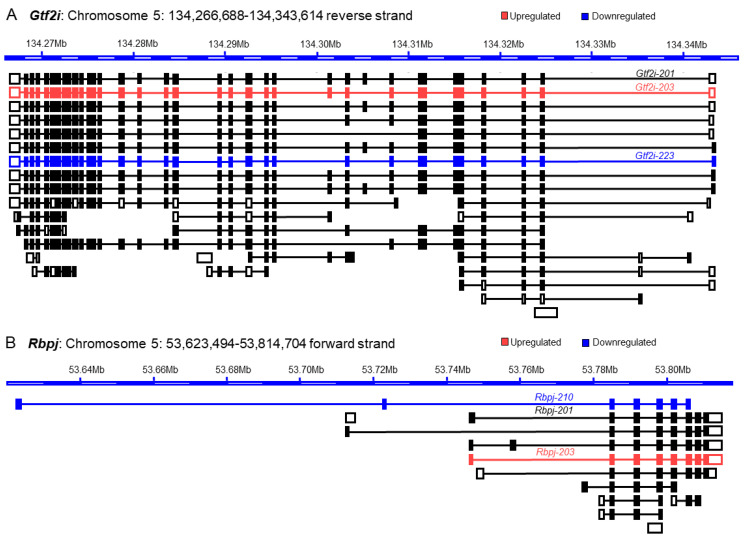
Impact of differential expression of transcript variants on the downregulated genes. GE-based expression analyses identified both *Gtf2i* and *Rbpj* as downregulated genes in mouse TS cells. We identified that transcript variant *Gtf2i-203* is significantly upregulated and lacks one exon (**A**). However, the upregulation of *Gtf2i-203* is masked by relatively higher downregulation of another transcript variant of *Gtf2i* (*Gtf2i-223*), which expresses a truncated protein lacking two protein-coding exons (**A**). We also observed that the upregulation of *Rbpj-203*, which encodes a full-length protein, remains unknown due to a higher downregulation of another transcript variant of *Rbpj* (*Rbpj-210*) that encodes a protein with a different domain at either end (**B**).

**Figure 8:**
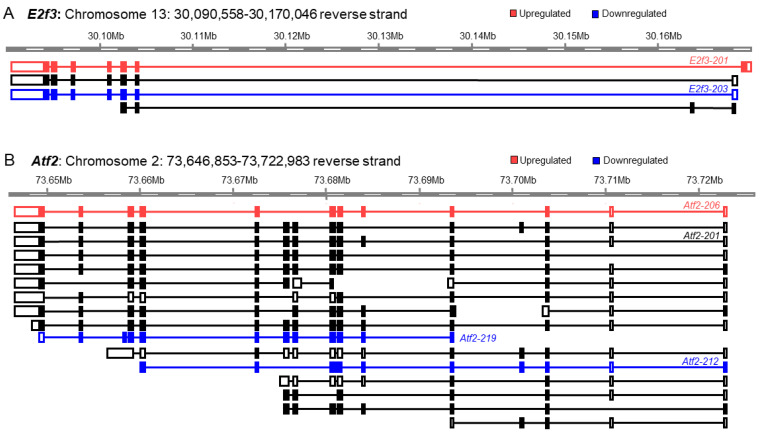
Impact of differential expression of transcript variants on the genes with insignificant differential expression. GE-based expression analyses did not identify both *E2f3* and *Atf2* as differentially expressed genes in mouse TS cells. However, we identified that a transcript variant of the *E2f3* gene (*E2f3-201*) was significantly upregulated, whereas another transcript variant of *E2f3* (*E2f3-203*) was significantly downregulated (**A**). Eventually, the upregulation of *E2f3-201* was masked by the downregulation of *E2f3-203*. Similarly, the significant upregulation of a transcript variant of *Atf2* (*Atf2-206*) and significant downregulation of two transcript variants of *Atf2* (*Atf2-219* and *Atf2-212*) remained undetected due to masking of *Atf2-206* results by those of *Atf2-219* and *Atf2-212* (**B**).

Our next step of investigation was directed towards elucidating the molecular basis of the discrepancy between analyses of gene expressions and transcript variants. In this analysis, we included two from each group of the upregulated, downregulated, or insignificant genes that demonstrated a discrepancy between their GE-based and TE-based analyses (as identified in Section 3.4) (Figure 5). Here, we have analyzed the transcript variants of *Kmt2a* and *Hmg20b* from the GE-based upregulated group (Figure 6), *Gtf2i* and *Rbpj* from the downregulated group (Figure 7), and *Atf2* and *E2f3* genes from the insignificant group (Figure 8). We identified that the downregulation of a transcript variant can be masked by relatively higher upregulation of another transcript variant while proteins are encoded with different functional domains (Figure 6A,B).

A similar mechanism also underlies the masked upregulated transcript variants of *Gtf2i* (*Gtf2i-203*) due to the higher downregulation of *Gtf2i-223*, which does not encode two exons of the full-length protein (Figure 7A). Another downregulated gene*, Rbpj,* also expresses an upregulated transcript variant, *Rbpj-203,* that expresses full-length protein, but this result remains unknown at the gene level due to higher downregulation of another transcript variant, *Rbpj-210*, which encodes a protein truncated at the amino terminus and insertion at the carboxy terminus (Figure 7B). 

We also observed that both significantly upregulated and downregulated transcript variants may mask each other, and their differential expressions remain unidentified during the analyses of GE-based gene expression (Figure 8A,B). GE-based expression analyses did not identify either *E2f3* or *Atf2* as differentially expressed genes in mouse TS cells (Figure 8A,B). We identified that a transcript variant of the *E2f3* gene (*E2f3-201*) was significantly upregulated. However, the upregulation of *E2f3-201* was masked by significant downregulation of another transcript variant of *E2f3* (*E2f3-203*) (Figure 8A). Similarly, the significant upregulation of a transcript variant of *Atf2* (*Atf2-206*) remained masked by the significant downregulation of two transcript variants of *Atf2* (*Atf2-219* and *Atf2-212*) (Figure 8B). 

## 4. Discussion

In RNA-Seq analyses, it is often assumed that any specific gene expresses only one transcript, leading to the inference that one gene encodes one mRNA and one protein. However, this assumption overlooks the fact that a single gene can often encode multiple transcripts due to alternative transcription start sites and alternative splicing [8,33,34]. These transcript variants can encode multiple in-frame peptides containing different structural and functional domains [35]. Moreover, some of the transcript variants can serve as noncoding regulatory RNAs or may undergo nonsense-mediated decay [36,37]. Therefore, aggregating all transcript variants under a single gene name is not biologically accurate; future studies should include TE-based differential analyses of transcript variants.

In this study, we have used the Windows version of the CLC Genomics Workbench to analyze our GE-based and TE-based RNA-Seq data. The statistical methods employed in our study are the EM estimation algorithm during RNA sequencing and GLMs followed by a negative binomial distribution during differential expression analysis. However, due to a large variety in RNA-Seq methodology across experiments, here we acknowledge other potential choices for analysis and their impacts. 

For instance, other software for RNA-Seq analyses, including Partek (Chesterfield, MO, USA), Lasergene (DNASTAR Inc., Madison, WI, USA), and Ugene [38,39,40], can also perform similar analyses, including alignments and identification of splice variants or transcript isoforms. For RNA-Seq differential expression, currently, there are two major differential expression statistical packages used [41]. The first is DESeq2, and the second is known as edgeR. Previously, both packages had differing normalization methods where DESeq2 had a computed scaling factor as the median of the ratio of its geometric mean across lanes, and edgeR applied TMM, trimmed mean of M-values, which computes the weighted mean of log ratios between the test sample and the reference [42]. Now, these packages use an optimized approach of applying a GLM to each gene, assuming read counts with a negative binomial distribution, and Wald tests or likelihood ratio tests [43]. In addition, after read alignment, CLC and other genomic software packages apply quality control measures of count data, allowing for high reproducibility among technical replicates [17]. Overall, CLC’s model follows a nearly identical sequence to the modified models of DESeq2 and edgeR. Once applied, the general and most important trend of our analysis stands: it is inaccurate to analyze RNA sequences based on GE, since transcript variants are summed regardless of their upregulation, downregulation, or neutrality [32]. This will be the case regardless of performing different algorithms on the data.

Additionally, almost all RNA-Seq processes, including CLC, begin with an alignment to a reference genome. Not only is the reference genome used during this study, Mouse Genome Assembly 39, the most recent and comprehensive Ensembl available during the manuscript preparation; but CLC’s specific aligner algorithm, which utilizes the expectation–maximum algorithm, is described as the most consistently accurate performer when benchmarked against other popular aligners, namely STAR and NOVOALIGN at the “junction level” [44]. CLC also stands as a top performer in all data sets except for those that were the least complex, where complexity is determined by the difficulty of alignment in a specific region.

This study was premised on the quantitative differences in the expression of the transcript variants of TF genes in mouse ES and TS cells. All the transcript variants are included under one gene name in GE-based analyses, but we have detected that the expression trends of these variants are not uniform. For instance, while a transcript variant expressed by a gene shows lineage-specific upregulation, another transcript expressed by the same gene can be downregulated simultaneously. These diverse patterns of transcript variant expression in the same cell type may lead to erroneous estimations in GE-based differential gene expression analyses. Therefore, we quantified the potential errors arising from ignoring the differential expression of transcript variants (Figure 4 and Figure 5).

Although the overall differential expression of TF genes and transcript variants was comparable between mouse ES and TS cells (Figure 4), deeper analyses illuminated a different picture of discrepancy (Figure 5, Figure 6, Figure 7 and Figure 8). We determined that only 14% (51 out of 365) of upregulated genes did not express any downregulated or insignificant variants, and only 17% (48 out of 287) of the downregulated genes did not express any upregulated or insignificant transcript variants. Collectively, transcript variants expressed by more than 80% of the differentially expressed genes yielded inaccurate interpretations. Many upregulated genes included transcript variants that were either downregulated or had no significant changes (Figure 4, Figure 5, Figure 6, Figure 7 and Figure 8). Many downregulated genes included upregulated transcript variants and transcripts without significant differences (Figure 4, Figure 5, Figure 6, Figure 7 and Figure 8). Furthermore, genes that did not show significant changes in GE-based analyses expressed differentially expressed transcript variants (Figure 4, Figure 5, Figure 6, Figure 7 and Figure 8).

More detailed analyses revealed that the transcript variants’ copy number (TPM value) plays an important role in determining the discrepancy between GE- and TE-based analyses. Among the upregulated genes, approximately 86% of the low-copy-number transcript variants were discordant, whereas it was about 62% in the case of high-copy-number transcripts (Figure 5G,H). Among the downregulated genes, approximately 93% of the low-copy-number transcript variants were discordant, whereas it was about 60% in the case of high-copy-number transcripts (Figure 5G,H). These observations indicate that low-copy-number transcript variants show more discrepancy. We suspect that statistical analyses sort the low-copy-number transcript variants more towards the insignificant group (Figure 5G). In contrast, the high-copy-number transcript variants stay more within the expected differentially expressed gene groups (Figure 5H). Notably, we detected a different pattern among the transcript variants corresponding to the insignificant group. While 90% of the low-copy-number transcript variants remained insignificant, it was reduced to 70% among the high-copy-number transcripts (Figure 5G,H). The high-copy-number transcript variants were sorted from the insignificant to the differentially expressed group (Figure 5G,H). Based on these findings, we can assume that if the sample size is increased, the genes or transcript variants in the insignificant group will decrease, and those in the upregulated or downregulated groups will increase. However, as most RNA-Seq studies include three samples in each group, we have used three libraries in each group. 

We elucidated the underlying mechanism that results in the discrepant behavior of RNA-Seq between GE-based and TE-based differential expression analyses (Figure 6, Figure 7 and Figure 8). The chosen genes specifically have notable up- and downregulations, but these display contradicting regulations when compared to the expression value and fold change of their individual transcript variants. We have also included statistically insignificant genes that have statistically significant transcript variants—another example of the discordance between both outputs of RNA sequencing. A gene can be identified as upregulated on a GE basis if one or more of its transcript variants are highly upregulated despite one or more of its transcript variants’ low-level downregulations (Figure 6). Similarly, a gene can be identified as downregulated on a GE basis if one or more of its transcript variants are highly downregulated despite one or more of its transcript variants’ low-level upregulations (Figure 7). As expected, when a transcript variant of a gene shows upregulation and another variant shows downregulation, that may result in an insignificant difference in GE-based expression analysis despite the presence of two differentially expressed transcript variants (Figure 8). 

If transcript variants are not included in RNA-Seq analyses, we fail to identify mechanisms that define a particular cell type. Cell-lineage-specific transcriptional and posttranscriptional machinery generates transcript variants of divergent molecular functions [45]. Without transcript variant consideration, we ignore transcriptional and posttranscriptional mechanisms. Alternative promoters can also express transcript variants, suggesting that without exploration of alternative transcript start sites, different regulatory mechanisms involved in gene expression and transcription initiation will remain unexplored. Lastly, genes also express transcript variants as long noncoding RNAs that have gene regulatory roles. Thus, we potentially fail to uncover valuable gene regulatory information if we do not analyze the transcript variants at this level.

## 5. Conclusions

During RNA-Seq analyses, GE values are considered, and TE values of the transcript variants are ignored to avoid relative procedural complexity. This study demonstrates the errors that are made when GE-value-based differentially expressed genes are identified and transcript variants are not considered. Our results clearly indicate that gene expression analyses based on GE values are substantially inaccurate and do not enable a comprehensive analysis or interpretation of differential gene expression in cells. RNA-Seq analyses should consider TE values of the transcript variants to identify their differential expression.

## Data Availability

SRA, NCBI.

## Supplementary Materials

The following supporting information can be downloaded at: https://www.mdpi.com/article/10.3390/cells13171502/s1. Figure S1: Reverse correlation of the differentially expressed transcript variants with corresponding genes; Table S1-1: Transcript variants (TE) of upregulated genes (GE); Table S1-2: Expression values and metadata of downregulated transcript variants of upregulated genes; Table S1-3: Expression values and metadata of insignificant transcript variants of upregulated genes; Table S2-1: Transcript variants (TE) of downregulated genes (GE); Table S2-2: Expression values and metadata of downregulated transcript variants of downregulated genes; Table S2-3: Expression values and metadata of insignificant transcript variants of downregulated genes; Table S3-1: Transcript variants (TE) of insignificant genes (GE); Table S3-2: Expression values and metadata of downregulated transcript variants of insignificant genes; Table S3-3: Expression values and metadata of insignificant transcript variants of insignificant genes.

## References

[1] Zhang S., Pyne S., Pietrzak S., Halberg S., McCalla S.G., Siahpirani A.F., Sridharan R., Roy S. (2023). Inference of cell type-specific gene regulatory networks on cell lineages from single cell omic datasets. Nat. Commun..

[2] Lowe R., Shirley N., Bleackley M., Dolan S., Shafee T. (2017). Transcriptomics technologies. PLoS Comput. Biol..

[3] Chu Y., Corey D.R. (2012). RNA sequencing: Platform selection, experimental design, and data interpretation. Nucleic Acid.

[4] Wang Z., Gerstein M., Snyder M. (2009). RNA-Seq: A revolutionary tool for transcriptomics. Nat. Rev. Genet..

[5] Li W., Ballard J., Zhao Y., Long Q. (2024). Knowledge-guided learning methods for integrative analysis of multi-omics data. Comput. Struct. Biotechnol. J..

[6] Limbu M.S., Xiong T., Wang S. (2024). A review of Ribosome profiling and tools used in Ribo-seq data analysis. Comput. Struct. Biotechnol. J..

[7] Samuels D.S., Lybecker M.C., Yang X.F., Ouyang Z., Bourret T.J., Boyle W.K., Stevenson B., Drecktrah D., Caimano M.J. (2021). Gene Regulation and Transcriptomics. Curr. Issues Mol. Biol..

[8] Pal S., Gupta R., Kim H., Wickramasinghe P., Baubet V., Showe L.C., Dahmane N., Davuluri R.V. (2011). Alternative transcription exceeds alternative splicing in generating the transcriptome diversity of cerebellar development. Genome Res..

[9] Reyes A., Huber W. (2018). Alternative start and termination sites of transcription drive most transcript isoform differences across human tissues. Nucleic Acids Res..

[10] Alfonso-Gonzalez C., Hilgers V. (2024). (Alternative) transcription start sites as regulators of RNA processing. Trends Cell Biol..

[11] Xin D., Hu L., Kong X. (2008). Alternative promoters influence alternative splicing at the genomic level. PLoS ONE.

[12] Kelemen O., Convertini P., Zhang Z., Wen Y., Shen M., Falaleeva M., Stamm S. (2013). Function of alternative splicing. Gene.

[13] Piazzi M., Bavelloni A., Salucci S., Faenza I., Blalock W.L. (2023). Alternative splicing, RNA editing, and the current limits of next generation sequencing. Genes.

[14] Ha I., Roberts S., Maldonado E., Sun X., Kim L.U., Green M., Reinberg D. (1993). Multiple functional domains of human transcription factor IIB: Distinct interactions with two general transcription factors and RNA polymerase II. Genes Dev.

[15] Sonam D., Manoj B.M. (2018). Non-coding transcript variants of protein-coding genes—What are they good for?. RNA Biol..

[16] Johnson K.A., Krishnan A. (2022). Robust normalization and transformation techniques for constructing gene coexpression networks from RNA-seq data. Genome Biol..

[17] Conesa A., Madrigal P., Tarazona S., Gomez-Cabrero D., Cervera A., McPherson A., Szcześniak M.W., Gaffney D.J., Elo L.L., Zhang X. (2016). A survey of best practices for RNA-seq data analysis. Genome Biol..

[18] Jiang Z., Zhou X., Li R., Michal J.J., Zhang S., Dodson M.V., Zhang Z., Harland R.M. (2015). Whole transcriptome analysis with sequencing: Methods, challenges and potential solutions. Cell Mol. Life Sci..

[19] Takahashi K., Yamanaka S. (2016). A decade of transcription factor-mediated reprogramming to pluripotency. Nat. Rev. Mol. Cell Biol..

[20] Kubaczka C., Senner C.E., Cierlitza M., Araúzo-Bravo M.J., Kuckenberg P., Peitz M., Hemberger M., Schorle H. (2015). Direct Induction of Trophoblast Stem Cells from Murine Fibroblasts. Cell Stem Cell.

[21] Johnston A.D., Simões-Pires C.A., Thompson T.V., Suzuki M., Greally J.M. (2019). Functional genetic variants can mediate their regulatory effects through alteration of transcription factor binding. Nat. Commun..

[22] Barrett T., Wilhite S.E., Ledoux P., Evangelista C., Kim I.F., Tomashevsky M., Marshall K.A., Phillippy K.H., Sherman P.M., Holko M. (2013). NCBI GEO: Archive for functional genomics data sets--update. Nucleic Acids Res..

[23] Tanaka S., Kunath T., Hadjantonakis A.K., Nagy A., Rossant J. (1998). Promotion of trophoblast stem cell proliferation by FGF4. Science.

[24] Chakravarthi V.P., Ratri A., Masumi S., Borosha S., Ghosh S., Christenson L.K., Roby K.F., Wolfe M.W., Rumi M.A.K. (2021). Granulosa cell genes that regulate ovarian follicle development beyond the antral stage: The role of estrogen receptor β. Mol. Cell Endocrinol.

[25] Khristi V., Chakravarthi V.P., Singh P., Ghosh S., Pramanik A., Ratri A., Borosha S., Roby K.F., Wolfe M.W., Rumi M.A.K. (2018). ESR2 regulates granulosa cell genes essential for follicle maturation and ovulation. Mol. Cell Endocrinol.

[26] Khristi V., Ratri A., Ghosh S., Pathak D., Borosha S., Dai E., Roy R., Chakravarthi V.P., Wolfe M.W., Karim Rumi M.A. (2019). Disruption of ESR1 alters the expression of genes regulating hepatic lipid and carbohydrate metabolism in male rats. Mol. Cell Endocrinol.

[27] Lambert S.A., Jolma A., Campitelli L.F., Das P.K., Yin Y., Albu M., Chen X., Taipale J., Hughes T.R., Weirauch M.T. (2018). The Human Transcription Factors. Cell.

[28] Nelder J.A., Wedderburn R.W. (1972). Generalized linear models. J. R. Stat. Soc. Ser. A: Stat. Soc..

[29] Lin J., Khan M., Zapiec B., Mombaerts P. (2016). Efficient derivation of extraembryonic endoderm stem cell lines from mouse postimplantation embryos. Sci. Rep..

[30] Ralston A., Cox B.J., Nishioka N., Sasaki H., Chea E., Rugg-Gunn P., Guo G., Robson P., Draper J.S., Rossant J. (2010). Gata3 regulates trophoblast development downstream of Tead4 and in parallel to Cdx2. Development.

[31] Takahashi K., Yamanaka S. (2006). Induction of pluripotent stem cells from mouse embryonic and adult fibroblast cultures by defined factors. Cell.

[32] Soneson C., Love M.I., Robinson M.D. (2015). Differential analyses for RNA-seq: Transcript-level estimates improve gene-level inferences. F1000Research.

[33] Stamm S., Ben-Ari S., Rafalska I., Tang Y., Zhang Z., Toiber D., Thanaraj T., Soreq H. (2005). Function of alternative splicing. Gene.

[34] Ashkenas J. (1997). Gene regulation by mRNA editing. Am. J. Hum. Genet..

[35] Ray T.A., Cochran K., Kozlowski C., Wang J., Alexander G., Cady M.A., Spencer W.J., Ruzycki P.A., Clark B.S., Laeremans A. (2020). Comprehensive identification of mRNA isoforms reveals the diversity of neural cell-surface molecules with roles in retinal development and disease. Nat. Commun..

[36] Sun B., Chen L. (2023). Mapping genetic variants for nonsense-mediated mRNA decay regulation across human tissues. Genome Biol..

[37] Marchese F.P., Raimondi I., Huarte M. (2017). The multidimensional mechanisms of long noncoding RNA function. Genome Biol..

[38] Okonechnikov K., Golosova O., Fursov M. (2012). Unipro UGENE: A unified bioinformatics toolkit. Bioinformatics.

[39] Golosova O., Henderson R., Vaskin Y., Gabrielian A., Grekhov G., Nagarajan V., Oler A.J., Quiñones M., Hurt D., Fursov M. (2014). Unipro UGENE NGS pipelines and components for variant calling, RNA-seq and ChIP-seq data analyses. PeerJ.

[40] Rose R., Golosova O., Sukhomlinov D., Tiunov A., Prosperi M. (2019). Flexible design of multiple metagenomics classification pipelines with UGENE. Bioinformatics.

[41] Lee G.Y., Ham S., Lee S.V. (2024). Brief guide to RNA sequencing analysis for nonexperts in bioinformatics. Mol. Cells.

[42] Dillies M.A., Rau A., Aubert J., Hennequet-Antier C., Jeanmougin M., Servant N., Keime C., Marot G., Castel D., Estelle J. (2013). A comprehensive evaluation of normalization methods for Illumina high-throughput RNA sequencing data analysis. Brief Bioinform.

[43] Yi L., Pimentel H., Bray N.L., Pachter L. (2018). Gene-level differential analysis at transcript-level resolution. Genome Biol..

[44] Baruzzo G., Hayer K.E., Kim E.J., Di Camillo B., FitzGerald G.A., Grant G.R. (2017). Simulation-based comprehensive benchmarking of RNA-seq aligners. Nat. Methods.

[45] Ju W., Greene C.S., Eichinger F., Nair V., Hodgin J.B., Bitzer M., Lee Y.-s., Zhu Q., Kehata M., Li M. (2013). Defining cell-type specificity at the transcriptional level in human disease. Genome Res..

