# Supplementary material for: Importance of Transcript Variants in Transcriptome Analyses
Source: Cells. 2024 Sep 8;13(17):1502. doi: 10.3390/cells13171502 (PMC11394320; doi:10.3390/cells13171502)
Supplement: Supplementary file 1 [file cells-13-01502-s001.zip › cells-3125475-supplementary.pdf]

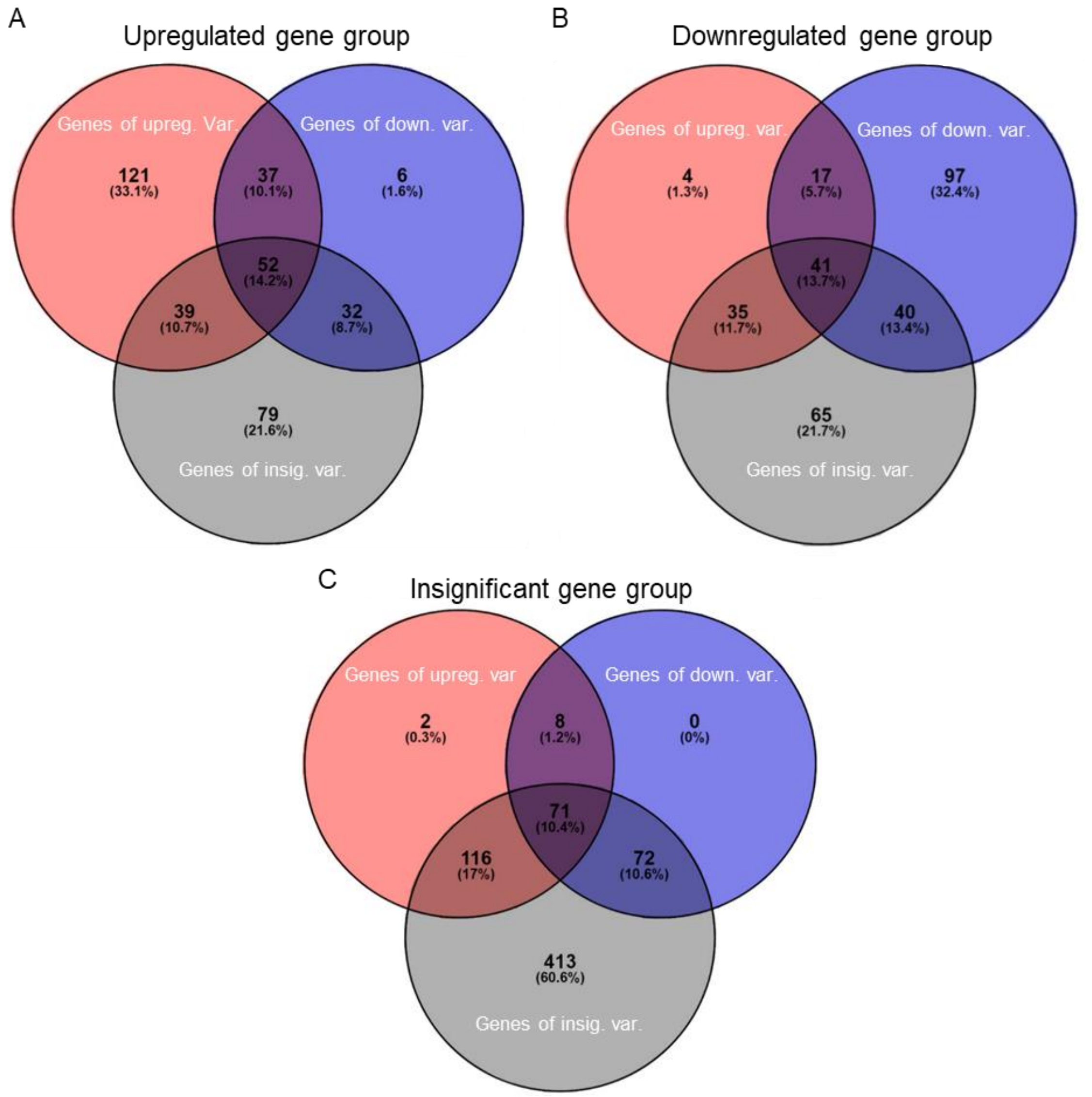

Figure S1. Reverse correlation of the differentially expressed transcript variants with corresponding genes. We observed that the transcript variants encoded by upregulated, downregulated, and insignificant genes show discordance with their respective gene group (**Figure 4**). We have performed a reverse correlation of the differentially expressed transcript variants compressed to their respective genes. The results are shown according to original gene expression groups. Either the upregulated (**A**) or the downregulated (**B**) group showed ~44% of the transcript variants to be overlapping, ~22% to be insignificant, and ~34% correlating to the respective gene group. The transcript variants of the insignificant (C) group showed ~40% to be overlapping, ~60% to be insignificant, but almost none to be upregulated or downregulated. These findings indicate that at least 40% of the genes encode transcript variants that can be either upregulated, downregulated, or insignificant despite their original gene group. upreg. var, upregulated variants; down. var., downregulated variants; insig. var., insignificant variants.

Supplemental Tables: Expression Value & Metadata Tables

S.1. Transcript Variants (TE) of Upregulated Genes (GE)

Table S1-1. Expression values & metadata of upregulated transcript variants of upregulated genes

Name	Chromosome	Max group mean	Fold change	FDR p-value	ENSEMBL	Biotype
Ahdc1-203	4	17.32	2.74	1.47E-05	ENSMUST00000105915	protein coding
Arid3a-202	10	30.36	7.31	5.50E-07	ENSMUST00000105376	protein coding
Atoh8-201	6	0.89	13.19	3.00E-02	ENSMUST00000042646	protein coding
Barx1-201	13	25.93	35.43	5.08E-22	ENSMUST00000021813	protein coding
Bcl11b-203	12	1.29	5.13	9.54E-03	ENSMUST00000109891	protein coding
Cdc5lrt1-201	10	0.98	4.97	3.00E-02	ENSMUST00000219147	protein coding
Cdc5lrt4-201	10	0.8	4.96	4.00E-02	ENSMUST00000219565	protein coding
Cdx2-201	5	106.83	42.79	3.85E-93	ENSMUST00000031650	protein coding
Cebpa-201	7	8.7	1,698.94	9.08E-03	ENSMUST00000042985	protein coding
Cebpb-201	2	30.27	2.24	5.07E-04	ENSMUST00000070642	protein coding
Cux1-205	5	3.34	535.65	3.00E-02	ENSMUST00000175998	protein coding
Dlx1-201	2	0.9	6.08	4.28E-04	ENSMUST00000037119	protein coding
Dlx3-201	11	25.62	3.82	2.21E-10	ENSMUST00000092768	protein coding
Dmbx1-202	4	1.22	29.64	6.12E-05	ENSMUST00000084338	protein coding
Dmrt2-201	19	1.71	2.7	2.00E-02	ENSMUST00000053068	protein coding
Dmrta2-201	4	2.29	6.07	9.42E-03	ENSMUST00000061187	protein coding
Ebf2-203	14	0.87	11.43	1.11E-05	ENSMUST00000176161	protein coding
Ebf3-201	7	0.51	4.42	4.00E-02	ENSMUST00000033378	protein coding
Egr2-201	10	1.47	33.11	5.44E-04	ENSMUST00000048289	protein coding
Elf2-201	3	28.82	4.92	2.16E-03	ENSMUST00000062009	protein coding
Elf2-204	3	10.48	2,020.82	7.08E-03	ENSMUST00000108053	protein coding

Elf4-202	X	8.3	3,837.86	2.00E-02	ENSMUST00000114958	protein coding
Elf5-201	2	336.4	4,445.84	5.08E-33	ENSMUST00000028609	protein coding
Elf5-203	2	20.64	149.06	2.83E-06	ENSMUST00000126290	protein coding
Eomes-201	9	181.17	6.98	5.03E-89	ENSMUST00000035020	protein coding
Eomes-202	9	41.29	21.89	1.45E-29	ENSMUST00000111763	protein coding
Eomes-203	9	10.28	6.77	1.58E-10	ENSMUST00000150633	protein coding
Esrra-206	19	5.41	9.23	1.00E-02	ENSMUST00000239322	protein coding
Fbxl19-201	7	65.02	41.27	5.05E-63	ENSMUST00000033081	protein coding
Fosb-201	7	2.46	81.73	3.47E-04	ENSMUST00000003640	protein coding
Fosl1-201	19	26.34	13.57	3.11E-19	ENSMUST00000025850	protein coding
Foxc2-201	8	2.31	8.48	3.43E-04	ENSMUST00000054691	protein coding
Foxd2-201	4	0.74	5.84	2.00E-02	ENSMUST00000068654	protein coding
Foxe1-201	4	13.65	89.67	9.28E-18	ENSMUST00000095097	protein coding
Foxf1-201	8	2.28	61.32	2.61E-04	ENSMUST00000181504	protein coding
Foxg1-201	12	0.99	17.46	1.00E-02	ENSMUST00000021333	protein coding
Foxl2-201	9	1.6	385.88	4.00E-02	ENSMUST00000051312	protein coding
Gata2-201	6	34.96	363.6	4.06E-18	ENSMUST00000015197	protein coding
Gata2-202	6	31.59	6.59	1.75E-14	ENSMUST00000170089	protein coding
Gata3-201	2	51.76	207.1	1.79E-43	ENSMUST00000102976	protein coding
Glis1-201	4	3.87	108.45	2.72E-04	ENSMUST00000046005	protein coding
Gsc-201	12	2.55	19.72	2.49E-03	ENSMUST00000021513	protein coding
Hand1-201	11	344.31	1,946.71	3.38E-34	ENSMUST00000036917	protein coding
Hand1-203	11	9.2	18.87	4.21E-09	ENSMUST00000160392	protein coding
Heyl-201	4	0.41	20.74	1.00E-02	ENSMUST00000040821	protein coding
Hic1-201	11	3.45	897.55	2.00E-02	ENSMUST00000055619	protein coding
Hlx-201	1	1.5	8.45	2.49E-03	ENSMUST00000048572	protein coding

Hmg20b-201	10	29.16	9.48	1.03E-08	ENSMUST00000020454	protein coding
Hmg20b-203	10	3.85	442.08	4.00E-02	ENSMUST00000105324	protein coding
Hmga1-203	17	46.56	5,488.18	1.61E-03	ENSMUST00000117600	protein coding
Hmga1-206	17	69.97	5,330.37	1.68E-03	ENSMUST00000119486	protein coding
Hmga1-211	17	11.21	8.81	9.66E-03	ENSMUST00000231825	protein coding
Hmga1-216	17	507.71	63,162.20	2.68E-05	ENSMUST00000232265	protein coding
Hmga1-217	17	30.17	2,042.16	6.93E-03	ENSMUST00000232552	protein coding
Hmx1-201	5	3.03	37.11	6.10E-04	ENSMUST00000087674	protein coding
Hoxa3-201	6	3.01	4.03	1.00E-02	ENSMUST00000114434	protein coding
Hoxa5-201	6	1.01	3.95	3.00E-02	ENSMUST00000048794	protein coding
Hoxa7-201	6	5.4	2.94	2.00E-02	ENSMUST00000048715	protein coding
Hoxc10-201	15	3.03	3.99	2.40E-03	ENSMUST00000001699	protein coding
Hoxc6-201	15	1.21	11.21	2.00E-02	ENSMUST00000001711	protein coding
Hoxd9-201	2	2.22	15	2.50E-05	ENSMUST00000059272	protein coding
Hsf4-201	8	5.76	84.12	3.49E-04	ENSMUST00000036127	protein coding
Insm1-201	2	23.55	47.26	2.70E-34	ENSMUST00000089257	protein coding
Irf2-201	8	23.65	4,365.98	2.33E-03	ENSMUST00000034041	protein coding
Irf2-210	8	1.2	8.94	2.00E-02	ENSMUST00000210284	nonsense mediated decay
Irx1-201	13	6.6	19.92	4.60E-09	ENSMUST00000077337	protein coding
Irx1-203	13	3.84	18.66	2.60E-03	ENSMUST00000223460	protein coding
Irx2-201	13	21.96	13.81	3.10E-15	ENSMUST00000074372	protein coding
Irx3-201	8	3.21	21.12	2.51E-06	ENSMUST00000093312	protein coding
Irx3-202	8	64.39	13,115.95	4.14E-04	ENSMUST00000175795	protein coding
Irx4-202	13	8.65	20.64	6.99E-09	ENSMUST00000176684	protein coding
Irx5-201	8	2.83	9.77	2.24E-04	ENSMUST00000034184	protein coding
Isl2-201	9	1.2	14.39	2.00E-02	ENSMUST00000034869	protein coding

Jun-201	4	13.2	4.24	2.11E-10	ENSMUST00000107094	protein coding
Jun-202	4	12.88	3.89	3.42E-09	ENSMUST00000249436	protein coding
Junb-201	8	112.5	2.45	3.15E-06	ENSMUST00000064922	protein coding
Klf14-201	6	4.9	10.54	5.09E-07	ENSMUST00000101589	protein coding
Lhx2-201	2	6.21	61.41	1.18E-09	ENSMUST00000000253	protein coding
Lhx6-202	2	1.91	468.21	4.00E-02	ENSMUST00000112961	protein coding
Lmx1b-201	2	3.88	21.7	1.69E-10	ENSMUST00000041730	protein coding
Maff-201	15	26.63	11.5	1.36E-09	ENSMUST00000096350	protein coding
Mafk-201	5	30.35	2.01	5.66E-04	ENSMUST00000018287	protein coding
Mbnl2-202	14	1.19	399.09	4.00E-02	ENSMUST00000167459	protein coding
Mef2d-203	3	1.31	3.71	5.00E-02	ENSMUST00000107559	protein coding
Meis1-201	11	37.22	15.06	1.01E-18	ENSMUST00000068264	protein coding
Meis1-206	11	1.05	50.68	4.32E-04	ENSMUST00000144988	protein coding
Meis1-207	11	1.22	14.26	2.00E-02	ENSMUST00000177357	nonsense mediated decay
Meis1-208	11	1.24	22.92	6.42E-05	ENSMUST00000177417	protein coding
Meis3-205	7	4.39	5.05	2.00E-02	ENSMUST00000176506	protein coding
Msx1-201	5	14.46	13.84	5.66E-10	ENSMUST00000063116	protein coding
Mycl-201	4	14.98	4.2	2.42E-04	ENSMUST00000030407	protein coding
Nfatc1-201	18	2.93	4.13	1.77E-03	ENSMUST00000035800	protein coding
Nfatc1-203	18	1.39	477.54	3.00E-02	ENSMUST00000167977	protein coding
Nfatc1-204	18	1.72	586.05	3.00E-02	ENSMUST00000170905	protein coding
Nfatc1-205	18	3.95	17.45	5.08E-07	ENSMUST00000236310	protein coding
Nfatc1-206	18	1.41	505.83	3.00E-02	ENSMUST00000236711	protein coding
Nfatc2-202	2	2.76	2.67	2.00E-02	ENSMUST00000074618	protein coding
Nfix-204	8	2.81	3.75	1.87E-06	ENSMUST00000109764	protein coding
Nkx6-1-201	5	3.09	108.57	4.63E-05	ENSMUST00000044125	protein coding

Nr2f2-201	7	1.49	25.36	1.71E-05	ENSMUST00000032768	protein coding
Olig2-201	16	1.01	7.98	4.70E-03	ENSMUST00000035608	protein coding
Onecut1-201	9	0.78	6.47	0.01	ENSMUST00000056006	protein coding
Onecut2-201	18	0.54	3.23	3.00E-02	ENSMUST00000175965	protein coding
Otx1-201	11	6.09	6.87	3.61E-08	ENSMUST00000006071	protein coding
Pax3-201	1	0.89	15.99	2.64E-03	ENSMUST00000004994	protein coding
Pax9-201	12	1.43	10.82	1.02E-04	ENSMUST00000001538	protein coding
Pbx3-201	2	32.78	8.6	3.66E-10	ENSMUST00000040638	protein coding
Pbx3-202	2	33.22	2.44	5.15E-06	ENSMUST00000113132	protein coding
Pbx3-207	2	2.46	15.97	2.00E-02	ENSMUST00000153278	Nonsense mediated decay
Peg3-201	7	71.66	2.45	4.47E-20	ENSMUST00000051209	protein coding
Peg3-205	7	5.11	4.98	3.33E-04	ENSMUST00000239104	protein coding
Phf19-201	2	85.71	3.18	1.19E-18	ENSMUST00000028232	protein coding
Phf19-205	2	18.26	4.90	2.00E-02	ENSMUST00000202907	protein coding
Pitx1-201	13	3.82	29.15	1.45E-06	ENSMUST00000021968	protein coding
Pou3f1-201	4	112.71	13.03	3.32E-113	ENSMUST00000053491	protein coding
Pou3f3-201	1	0.31	15.85	3.00E-02	ENSMUST00000054883	protein coding
Prdm6-201	18	1.29	3.65	5.47E-03	ENSMUST00000091900	protein coding
Prox1-202	1	2.86	4.83	1.59E-03	ENSMUST00000175916	protein coding
Runx1-203	16	5.38	10.15	2.20E-05	ENSMUST00000168195	protein coding
Satb1-208	17	3.71	280.2	5.21E-05	ENSMUST00000144331	protein coding
Satb2-201	1	6.21	124.27	1.68E-12	ENSMUST00000042857	protein coding
Satb2-202	1	26.88	10.45	2.32E-19	ENSMUST00000114415	protein coding
Scmh1-201	4	62.86	2.39	1.18E-09	ENSMUST00000000087	protein coding
Sim2-201	16	1.03	310.71	5.00E-02	ENSMUST00000072182	protein coding
Six2-201	17	4	63.29	1.83E-04	ENSMUST00000163568	protein coding

Six3-202	17	1.02	14.69	6.64E-03	ENSMUST00000175898	protein coding
Six3-203	17	0.95	7.43	4.82E-03	ENSMUST00000176081	protein coding
Snai1-201	2	32.33	4.63	5.39E-07	ENSMUST00000052631	protein coding
Snai3-201	8	36.73	2.45	1.59E-06	ENSMUST00000006762	protein coding
Sox10-201	15	3.48	3.21	1.00E-02	ENSMUST00000040019	protein coding
Sox21-201	14	40.86	11.47	4.83E-46	ENSMUST00000170662	protein coding
Sox3-201	X	106.7	10.68	6.16E-57	ENSMUST00000135107	protein coding
Sox9-201	11	4.02	30.27	7.63E-10	ENSMUST00000000579	protein coding
Sp6-202	11	19.34	8.39	2.60E-05	ENSMUST00000107622	protein coding
Srebf2-203	15	2.34	62	1.46E-04	ENSMUST00000229336	protein coding
Stat5a-202	11	11.71	2.54	1.00E-02	ENSMUST00000107356	protein coding
T-201	17	8.11	4.91	1.00E-07	ENSMUST00000074667	protein coding
T-202	17	3.07	12.87	0.01	ENSMUST00000136922	protein coding
T-203	17	2.45	21.39	1.00E-02	ENSMUST00000177118	protein coding
Tbx15-201	3	12.63	8.94	4.76E-23	ENSMUST00000029462	protein coding
Tbx18-201	9	7.35	73.44	1.53E-15	ENSMUST00000034991	protein coding
Tbx2-201	11	0.9	10.2	1.72E-03	ENSMUST00000000095	protein coding
Tcf7-202	11	44.08	2.7	3.21E-04	ENSMUST00000109071	protein coding
Tead3-206	17	59.8	428.53	4.32E-23	ENSMUST00000156862	protein coding
Tfap2c-201	2	243.46	3.48	7.52E-42	ENSMUST00000030391	protein coding
Tfap2c-202	2	20.36	14.15	6.59E-11	ENSMUST00000099058	protein coding
Tlx3-201	11	13.7	10.02	1.18E-05	ENSMUST00000037746	protein coding
Tshz1-201	18	1.74	66.16	7.57E-05	ENSMUST00000060303	protein coding
Tshz1-202	18	2.98	3.38	3.77E-03	ENSMUST00000175783	protein coding
Vdr-201	15	9.43	20.52	4.30E-33	ENSMUST00000023119	protein coding
Wt1-204	2	10.59	3.55	1.60E-04	ENSMUST00000139585	protein coding

Wt1-205	2	9.28	19.26	3.61E-08	ENSMUST00000143043	protein coding
Zbtb10-201	3	38.4	2.83	9.82E-14	ENSMUST00000155203	protein coding
Zbtb4-203	11	1.82	4.46	3.00E-02	ENSMUST00000108640	protein coding
Zbtb42-201	12	4.15	2.88	2.00E-02	ENSMUST00000169593	protein coding
Zbtb7b-201	3	60.09	2,185.64	1.59E-14	ENSMUST00000029677	protein coding
Zbtb7b-203	3	4.17	4.44	1.31E-05	ENSMUST00000107433	protein coding
Zfhx3-204	8	0.36	463.25	3.00E-02	ENSMUST00000220518	protein coding
Zfp202-201	9	2.73	700.5	2.00E-02	ENSMUST00000026693	protein coding
Zfp28-206	7	2.41	15.09	2.00E-02	ENSMUST00000208949	Nonsense mediated decay
Zfp316-201	5	1.49	340.2	4.00E-02	ENSMUST00000051665	protein coding
Zfp362-202	4	18.77	5.68	1.36E-06	ENSMUST00000106072	protein coding
Zfp429-201	13	2.95	3.48	3.00E-02	ENSMUST00000109732	protein coding
Zfp467-206	6	1.73	41.65	5.50E-04	ENSMUST00000114561	protein coding
Zfp469-202	8	2.78	25.42	7.33E-18	ENSMUST00000187142	protein coding
Zfp503-201	14	2.13	10.56	2.10E-04	ENSMUST00000043409	protein coding
Zfp579-201	7	3.26	41.94	1.27E-03	ENSMUST00000108572	protein coding
Zfp641-202	15	1.14	366.59	4.00E-02	ENSMUST00000169721	protein coding
Zfp651-201	9	4.71	2.06	4.60E-04	ENSMUST00000093772	protein coding
Zfp703-202	8	28.69	25.42	1.00E-28	ENSMUST00000154256	protein coding
Zfpm1-201	8	107.37	15.82	2.29E-49	ENSMUST00000054052	protein coding
Zic1-201	9	1.15	44.72	3.47E-04	ENSMUST00000034927	protein coding
Zic1-202	9	4.58	7.92	8.66E-06	ENSMUST00000065360	protein coding
Zkscan17-201	11	113.53	2	3.12E-13	ENSMUST00000013262	protein coding

Table S1-2. Expression values & metadata of downregulated transcript variants of upregulated genes						
**Name**	**Chromosome**	**Max group mean**	**Fold change**	**FDR p-value**	**ENSEMBL**	**Biotype**
Ahdc1-201	4	15.14	-3.64	4.38E-18	ENSMUST00000044521	protein coding
Bmal2-201	6	0.22	-142.93	2.00E-02	ENSMUST00000080530	protein coding
Creb3l4-201	3	2.03	-3.27	0.05	ENSMUST00000029547	protein coding
Cux1-204	5	0.56	-2.78	0.02	ENSMUST00000175975	protein coding
Cux1-206	5	3.34	-2.71	1.00E-02	ENSMUST00000176172	protein coding
Cux1-208	5	1.68	-16.81	2.72E-08	ENSMUST00000176423	protein coding
Cux1-209	5	0.56	-77.15	6.13E-04	ENSMUST00000176486	protein coding
Dpf3-204	12	0.06	-69.14	2.00E-02	ENSMUST00000147469	protein coding
Ebf3-204	7	0.15	-100.25	1.00E-02	ENSMUST00000209578	protein coding
Egr1-201	18	32.72	-2.15	2.15E-09	ENSMUST00000064795	protein coding
Ehf-203	2	0.05	-63.89	2.00E-02	ENSMUST00000111176	protein coding
Elf2-203	3	14.95	-7.28	8.69E-58	ENSMUST00000108051	protein coding
Elf2-208	3	7.81	-8.98	2.69E-28	ENSMUST00000183463	nonsense mediated decay
Elf2-212	3	0.82	-3.55	8.62E-06	ENSMUST00000194641	protein coding
Elf4-201	X	11.51	-2.09	4.24E-08	ENSMUST00000033429	protein coding
Elf4-203	X	0.28	-145.01	8.06E-03	ENSMUST00000140486	protein coding
Esrra-203	19	0.83	-179.55	1.61E-05	ENSMUST00000172975	protein coding
Ets1-202	9	0.13	-93.65	2.00E-02	ENSMUST00000050797	protein coding
Ets1-207	9	0.09	-63.87	4.00E-02	ENSMUST00000238819	protein coding
Fbxl19-203	7	27.64	-2.66	1.89E-04	ENSMUST00000186207	protein coding
Fbxl19-206	7	1.16	-5.48	2.00E-02	ENSMUST00000205689	protein coding
Fbxl19-207	7	63.55	-2.4	2.09E-26	ENSMUST00000206893	protein coding
Fosb-205	7	0.32	-417.87	1.13E-04	ENSMUST00000208326	protein coding
Glis3-209	19	2.6	-2.19	0.02	ENSMUST00000162022	protein coding

Hic2-202	16	0.39	-261.49	1.57E-03	ENSMUST00000232082	protein coding
Hivep1-201	13	7.21	-2.04	3.86E-07	ENSMUST00000060148	protein coding
Hivep1-205	13	0.37	-21.59	0.05	ENSMUST00000222854	protein coding
Hmg20b-202	10	6.81	-4.79	2.27E-07	ENSMUST00000105323	protein coding
Hmg20b-210	10	11.05	-12.86	8.24E-44	ENSMUST00000141171	protein coding
Hmg20b-212	10	2.26	-6.59	3.81E-03	ENSMUST00000154609	protein coding
Hmga1-201	17	300.02	-4.65	3.68E-59	ENSMUST00000114888	protein coding
Hmga1-204	17	1.93	-362.79	7.58E-10	ENSMUST00000118570	protein coding
Hmga1-207	17	21.64	-118.67	5.11E-11	ENSMUST00000231243	protein coding
Hmga1-215	17	55.63	-1,148.36	4.26E-34	ENSMUST00000232253	protein coding
Klf6-203	13	2.73	-4.25	0.02	ENSMUST00000222857	nonsense mediated decay
Kmt2a-202	9	6.53	-2.61	3.06E-05	ENSMUST00000114689	protein coding
Lhx2-203	2	0.14	-84.9	2.00E-02	ENSMUST00000143783	protein coding
Lhx6-204	2	5.27	-2.05	1.08E-04	ENSMUST00000112966	protein coding
Maff-202	15	11.9	-4.13	1.33E-09	ENSMUST00000163691	protein coding
Mbnl2-204	14	4.32	-6.84	2.42E-08	ENSMUST00000227012	protein coding
Mbnl2-208	14	14.72	-3.36	5.49E-15	ENSMUST00000227594	protein coding
Mef2a-201	7	2.22	-4.22	8.52E-03	ENSMUST00000032776	protein coding
Mef2d-201	3	35.4	-2.04	1.07E-15	ENSMUST00000001455	protein coding
Nfatc2-209	2	0.33	-5.7	0.03	ENSMUST00000171689	protein coding
Nfia-204	4	0.02	-79.64	2.00E-02	ENSMUST00000107057	protein coding
Nfic-203	10	9.44	-2.31	4.97E-12	ENSMUST00000105321	protein coding
Nfkb2-201	19	17.34	-2.17	9.43E-06	ENSMUST00000073116	protein coding
Npas1-202	7	0.09	-62.82	0.03	ENSMUST00000210748	protein coding
Nr1d1-201	11	16.87	-2.06	5.28E-06	ENSMUST00000064941	protein coding
Ovol2-202	2	0.31	-16.46	2.00E-02	ENSMUST00000103171	protein coding

Pbx4-205	8	2.21	-3.07	3.00E-02	ENSMUST0000013 4777	nonsense mediated decay
Purg-201	8	5.52	-2.6	2.91E-05	ENSMUST0000007 0340	protein coding
Sall2-201	14	0.35	-628.65	3.10E-05	ENSMUST0000005 8326	protein coding
Satb1-203	17	7.49	-3.37	1.14E-06	ENSMUST0000012 9667	protein coding
Satb1-213	17	0.18	-399.54	3.40E-04	ENSMUST0000016 9480	protein coding
Scmh1-203	4	2.44	-4.79	5.00E-02	ENSMUST0000010 6298	protein coding
Smad3-204	9	0.4	-67.02	4.00E-02	ENSMUST0000013 7713	protein coding
Smad3-205	9	0.34	-87.82	3.00E-02	ENSMUST0000015 4323	protein coding
Sp4-203	12	8.15	-2.72	1.65E-08	ENSMUST0000022 2314	protein coding
Sp6-201	11	1.28	-10.06	1.77E-03	ENSMUST0000004 7997	protein coding
Srebf2-202	15	3.03	-184.03	2.27E-07	ENSMUST0000022 9009	protein coding
Tbx4-202	11	0.45	-123.25	2.51E-06	ENSMUST0000010 8045	protein coding
Tcf24-201	1	1.01	-5.96	2.06E-04	ENSMUST0000009 7824	protein coding
Tcf7-201	11	100.59	-2	1.05E-16	ENSMUST0000008 6844	protein coding
Tead3-201	17	0.82	-148.31	4.75E-06	ENSMUST0000011 4799	protein coding
Tead4-203	6	12.86	-2.35	1.32E-05	ENSMUST0000013 0454	protein coding
Trafd1-202	5	11.09	-2.69	4.24E-04	ENSMUST0000012 0784	protein coding
Vezf1-202	11	3.77	-2.01	2.53E-04	ENSMUST0000014 3052	protein coding
Wt1-202	2	0.41	-95.23	3.06E-06	ENSMUST0000011 1099	protein coding
Xpa-204	4	2.8	-3.38	3.00E-02	ENSMUST0000013 2358	nonsense mediated decay
Zbtb20-202	16	0.25	-243.62	1.37E-03	ENSMUST0000011 4690	protein coding
Zbtb42-203	12	0.42	-4.57	5.00E-02	ENSMUST0000017 4780	protein coding
Zbtb46-202	2	3.33	-4.53	5.91E-20	ENSMUST0000008 7409	protein coding
Zbtb7b-202	3	4.15	-1,145.26	4.05E-17	ENSMUST0000010 7432	protein coding
Zfp174-201	16	2.01	-2.23	4.22E-03	ENSMUST0000004 1778	protein coding
Zfp202-202	9	1.5	-3.22	4.60E-04	ENSMUST0000016 8691	nonsense mediated decay

Zfp202-203	9	0.39	-145.62	4.69E-08	ENSMUST00000168832	protein coding
Zfp358-202	8	0.5	-126.52	2.00E-02	ENSMUST00000207318	protein coding
Zfp362-201	4	0.56	-163.68	7.14E-08	ENSMUST00000071108	protein coding
Zfp429-204	13	4.6	-26.74	3.86E-10	ENSMUST00000224825	protein coding
Zfp46-205	4	0.45	-176.74	4.69E-03	ENSMUST00000144217	protein coding
Zfp628-201	7	5.81	-2.09	4.00E-02	ENSMUST00000116354	protein coding
Zfp708-203	13	0.07	-66.32	4.00E-02	ENSMUST00000190566	protein coding
Zfp773-201	7	1.57	-8.18	2.99E-06	ENSMUST00000032622	protein coding
Zfp787-204	7	7.72	-2.52	5.00E-02	ENSMUST00000207628	protein coding
Zfp787-205	7	5.02	-3.97	2.75E-07	ENSMUST00000207957	protein coding
Zfp791-201	8	1.72	-3.81	3.56E-03	ENSMUST00000098550	protein coding
Zfp820-202	17	3.87	-4.76	3.13E-06	ENSMUST00000232918	protein coding
Zkscan17-202	11	13.7	-21.8	1.30E-110	ENSMUST00000101150	protein coding

Table S1-3. Expression values & metadata of insignificant transcript variants of upregulated genes						
**Name**	**Chromosome**	**Max group mean**	**Fold change**	**FDR p-value**	**ENSEMBL**	**Biotype**
Aebp1-201	11	9.8	1.36	0.38	ENSMUST00000102923	protein coding
Aebp1-202	11	1.33	-2.6	0.11	ENSMUST00000109829	protein coding
Ahdc1-202	4	57.91	-1.25	0.2	ENSMUST00000105914	protein coding
Ahdc1-204	4	0.12	-2.08	0.61	ENSMUST00000105916	protein coding
Alx1-201	10	0.16	31.41	0.27	ENSMUST00000040859	protein coding
Alx1-203	10	0.09	17.83	0.38	ENSMUST00000167156	protein coding
Alx1-204	10	0.09	-1.04	0.99	ENSMUST00000217946	protein coding
Alx1-205	10	0.01	2.99	NaN	ENSMUST00000218282	protein coding
Alx1-208	10	0.14	-5.69	0.29	ENSMUST00000219194	protein coding
Alx4-201	2	0.34	1.89	0.36	ENSMUST00000042078	protein coding
Ar-201	X	0.18	-1.25	0.77	ENSMUST00000052837	protein coding
Arid3a-201	10	31.22	1.45	0.03	ENSMUST00000019708	protein coding
Arid3a-203	10	0.14	54.43	0.24	ENSMUST00000105377	protein coding
Arid3a-204	10	0.36	21.52	0.35	ENSMUST00000131118	protein coding
Arid3c-202	4	0.76	-1.2	0.84	ENSMUST00000150809	protein coding
Arid3c-203	4	1.34	-1.52	0.18	ENSMUST00000171251	protein coding
Ascl2-201	7	14.82	1.57	0.06	ENSMUST00000009392	protein coding
Ascl5-201	1	0.36	16.25	0.38	ENSMUST00000180436	protein coding
Atoh8-202	6	0.21	-1.65	0.72	ENSMUST00000206425	nonsense mediated decay
Barhl1-201	2	0.11	2.68	0.49	ENSMUST00000050776	protein coding
Barhl1-203	2	0.01	2.99	NaN	ENSMUST00000113849	protein coding
Barhl2-201	5	0.39	1.35	0.76	ENSMUST00000086795	protein coding
Batf3-201	1	12.44	2.39	0.1	ENSMUST00000027943	protein coding
Bcl11b-201	12	0.77	1.09	0.89	ENSMUST00000066060	protein coding

Bhlha9-201	11	0.39	35.21	0.26	ENSMUST00000056184	protein coding
Bhlhe41-201	6	2.53	1.46	0.48	ENSMUST00000032386	protein coding
Bhlhe41-202	6	0.61	-4.81	0.1	ENSMUST00000111703	protein coding
Bmal2-202	6	0.05	8.19	0.52	ENSMUST00000111636	protein coding
Bmal2-203	6	0.19	-2.53	0.51	ENSMUST00000111638	protein coding
Bmal2-204	6	3.64	1.74	0.23	ENSMUST00000111639	protein coding
Bmal2-206	6	0.85	104.43	0.13	ENSMUST00000129788	nonsense mediated decay
Bmyc-201	2	49.68	-1.21	0.51	ENSMUST00000061483	protein coding
Bnc1-201	7	0.29	8.08	0.09	ENSMUST00000026096	protein coding
Cbx2-201	11	79.05	-1.26	0.02	ENSMUST00000026662	protein coding
Ccdc17-201	4	0.71	-1.88	0.3	ENSMUST00000051869	protein coding
Cdc5lrt10-201	10	0.22	40.87	0.25	ENSMUST00000178998	protein coding
Cdc5lrt7-201	10	0.13	1.39	0.81	ENSMUST00000219760	protein coding
Cebpa-202	7	2.73	-1.56	0.35	ENSMUST00000205391	protein coding
Cebpd-201	16	1.18	-1.3	0.68	ENSMUST00000096232	protein coding
Creb3-201	4	35.15	-1.62	4.12E-03	ENSMUST00000102944	protein coding
Creb3-203	4	0.22	-3.48	0.37	ENSMUST00000132631	protein coding
Creb3l2-201	6	12.14	-1.44	7.01E-03	ENSMUST00000041093	protein coding
Creb3l3-201	10	1.35	-1.97	0.26	ENSMUST00000117422	protein coding
Creb3l4-202	3	7.09	-1.52	0.47	ENSMUST00000107369	protein coding
Crebzf-201	7	49.22	-1.29	0.09	ENSMUST00000061767	protein coding
Crebzf-202	7	0.42	2.7	0.51	ENSMUST00000107205	nonsense mediated decay
Crebzf-203	7	2.73	183.56	0.08	ENSMUST00000107206	nonsense mediated decay
Crebzf-204	7	0.02	-6.17	NaN	ENSMUST00000126041	nonsense mediated decay
Crebzf-205	7	15.64	-1.2	0.36	ENSMUST00000143408	nonsense mediated decay
Crebzf-207	7	8.43	-1.6	0.23	ENSMUST00000207252	nonsense mediated decay

Crebzf-208	7	4.15	-1.67	0.29	ENSMUST00000208379	nonsense mediated decay
Cux1-201	5	3.32	-1.52	0.14	ENSMUST00000004097	protein coding
Cux1-203	5	0.05	-4.22	0.3	ENSMUST00000175918	nonsense mediated decay
Cux1-207	5	6.21	-1.92	0.24	ENSMUST00000176216	protein coding
Cux1-211	5	23.51	-1.71	0.03	ENSMUST00000176745	protein coding
Cux1-212	5	0.44	181.17	0.08	ENSMUST00000176778	protein coding
Dlx2-201	2	0.59	2.41	0.29	ENSMUST00000024159	protein coding
Dlx4-201	11	1.74	-1.78	0.3	ENSMUST00000021241	protein coding
Dlx5-201	6	0.73	7.26	0.14	ENSMUST00000052609	protein coding
Dlx5-202	6	0.01	-6.17	NaN	ENSMUST00000142635	protein coding
Dlx6-201	6	0.81	38.98	0.25	ENSMUST00000031768	protein coding
Dlx6-202	6	0.36	4.66	0.34	ENSMUST00000160937	protein coding
Dlx6-203	6	0.09	7.7	0.53	ENSMUST00000171311	protein coding
Dmbx1-201	4	0.13	55.79	0.22	ENSMUST00000064806	protein coding
Dmbx1-203	4	1.95	1.48	0.41	ENSMUST00000124071	protein coding
Dmrt3-201	19	3.24	-1.88	0.06	ENSMUST00000048935	protein coding
Dmrt3-202	19	0.36	10.57	0.46	ENSMUST00000237821	protein coding
Dnttip1-201	2	43.41	-2	1.81E-07	ENSMUST00000017443	protein coding
Dnttip1-202	2	1.41	-1.11	0.93	ENSMUST00000109326	protein coding
Dnttip1-203	2	17.48	-1.59	0.29	ENSMUST00000109327	protein coding
Dpf3-202	12	1.05	-2.21	0.35	ENSMUST00000140327	protein coding
Dpf3-203	12	1.97	-1.39	0.44	ENSMUST00000144237	protein coding
Dpf3-205	12	2.98	285.48	0.05	ENSMUST00000177959	protein coding
Dpf3-206	12	0.95	224.06	0.07	ENSMUST00000178756	protein coding
Ebf2-201	14	0.09	32.59	0.28	ENSMUST00000022637	protein coding
Ebf2-202	14	0.22	1.33	0.85	ENSMUST00000176029	protein coding

Ebf3-202	7	0.04	11.46	0.46	ENSMUST0000010 6118	protein coding
Ebf3-203	7	0.17	63.09	0.21	ENSMUST0000016 9486	protein coding
Ebf3-207	7	0.17	38.99	0.25	ENSMUST0000021 0774	protein coding
Ebf4-201	2	1.41	279.27	0.06	ENSMUST0000011 0286	protein coding
Ebf4-203	2	1.28	284.19	0.05	ENSMUST0000011 0288	protein coding
Ebf4-204	2	0.38	80.66	0.15	ENSMUST0000012 6740	nonsense mediated decay
Ebf4-206	2	0.89	2.43	0.36	ENSMUST0000014 0169	nonsense mediated decay
Egr2-202	10	0.2	44.46	0.24	ENSMUST0000010 5438	protein coding
Egr2-203	10	0.04	-35.18	0.08	ENSMUST0000012 7820	protein coding
Egr2-204	10	0.04	-6.17	NaN	ENSMUST0000013 0933	protein coding
Egr2-205	10	0.04	-11.42	NaN	ENSMUST0000014 5754	protein coding
Egr2-206	10	0.02	-6.17	NaN	ENSMUST0000014 5936	protein coding
Ehf-201	2	0.31	99.11	0.13	ENSMUST0000009 0475	protein coding
Ehf-202	2	0.14	-7.28	0.15	ENSMUST0000011 1174	protein coding
Ehf-204	2	0.31	1.56	7.90E-01	ENSMUST0000012 5788	protein coding
Ehf-207	2	0.02	-6.17	NaN	ENSMUST0000014 0503	protein coding
Ehf-208	2	0.06	-11.42	NaN	ENSMUST0000015 1265	protein coding
Elf1-201	14	2.73	3.66	0.06	ENSMUST0000004 0131	protein coding
Elf1-202	14	14.05	-1.63	0.03	ENSMUST0000011 0835	protein coding
Elf1-204	14	2.83	1.04	0.97	ENSMUST0000022 7192	protein coding
Elf2-202	3	0.32	58.51	0.24	ENSMUST0000009 1144	protein coding
Elf2-206	3	12.64	-1.81	0.07	ENSMUST0000016 3748	protein coding
Elf2-207	3	0.99	1.69	5.70E-01	ENSMUST0000018 3338	nonsense mediated decay
Elf2-209	3	2.24	-2	0.28	ENSMUST0000018 4677	nonsense mediated decay
Elf2-213	3	0.52	-1.61	0.37	ENSMUST0000019 5432	protein coding
Elf5-206	2	0.62	120.42	1.20E-01	ENSMUST0000016 4172	protein coding

Elf5-207	2	0.55	104.97	0.13	ENSMUST00000239273	protein coding
Emx1-201	6	1.06	2.34	0.18	ENSMUST00000045942	protein coding
Emx2-201	19	0.07	14.36	0.4	ENSMUST00000062216	protein coding
Emx2-202	19	0.12	15.63	0.39	ENSMUST00000174353	protein coding
En1-201	1	1.62	-1.01	0.99	ENSMUST00000079721	protein coding
Erg-203	16	0.08	-60.84	0.05	ENSMUST00000122199	protein coding
Erg-204	16	0.07	-1.77	0.71	ENSMUST00000171646	protein coding
Erg-205	16	0.31	-2.19	0.49	ENSMUST00000176345	protein coding
Erg-206	16	0.43	10.57	0.12	ENSMUST00000233269	protein coding
Erg-207	16	0.09	20.34	0.36	ENSMUST00000233664	protein coding
Erg-208	16	0.36	-1.47	0.6	ENSMUST00000233881	protein coding
Esrra-201	19	29.52	1.33	0.39	ENSMUST00000025906	protein coding
Esrra-205	19	0.85	2.46	0.56	ENSMUST00000173635	protein coding
Esrrg-201	1	0.06	25.16	0.33	ENSMUST00000027906	protein coding
Esrrg-203	1	0.01	-9.65	0.17	ENSMUST00000110939	protein coding
Esx1-201	X	15.71	1.28	0.34	ENSMUST00000074698	protein coding
Esx1-202	X	38.98	1.47	0.15	ENSMUST00000113066	protein coding
Ets1-201	9	3.57	-1.39	0.05	ENSMUST00000034534	protein coding
Ets1-205	9	0.43	-10.48	0.19	ENSMUST00000184364	protein coding
Ets2-201	16	101.79	1.7	3.09E-08	ENSMUST00000023612	protein coding
Ets2-202	16	0.79	-1.11	0.95	ENSMUST00000232792	protein coding
Fbxl19-202	7	1.7	449.86	0.08	ENSMUST00000186116	protein coding
Fbxl19-204	7	26.44	1.7	0.16	ENSMUST00000188580	protein coding
Fbxl19-205	7	0.89	21.94	0.34	ENSMUST00000189562	protein coding
Fezf1-201	6	1.07	194.23	7.00E-02	ENSMUST00000031709	protein coding
Fli1-201	9	0.07	-1	1	ENSMUST00000016231	protein coding

Fli1-202	9	0.05	4.88	0.6	ENSMUST00000183767	protein coding
Fos-201	12	20.89	1.5	9.00E-02	ENSMUST00000021674	protein coding
Fosb-206	7	0.52	43.72	0.25	ENSMUST00000208446	protein coding
Fosb-207	7	0.15	2.14	0.63	ENSMUST00000208505	protein coding
Fosl1-203	19	0.59	1.32	0.87	ENSMUST00000236774	protein coding
Fosl2-201	5	14.6	1.55	0.02	ENSMUST00000031017	protein coding
Foxa1-201	12	0.6	-1.22	0.77	ENSMUST00000044380	protein coding
Foxa2-202	2	3.65	1.49	0.42	ENSMUST00000109964	protein coding
Foxc1-201	13	0.25	3.66	0.24	ENSMUST00000062292	protein coding
Foxf2-201	13	0.51	-1.47	0.54	ENSMUST00000042054	protein coding
Foxg1-204	12	0.29	1.24	0.81	ENSMUST00000179669	protein coding
Foxj1-201	11	3.43	1.71	0.2	ENSMUST00000036215	protein coding
Foxj2-201	6	17.82	1.33	0.1	ENSMUST00000003238	protein coding
Foxj2-202	6	1.45	3	0.46	ENSMUST00000203075	protein coding
Foxk1-201	5	29.33	-1.35	0.02	ENSMUST00000072837	protein coding
Foxk1-202	5	5.50E-01	1.76	0.71	ENSMUST00000198422	protein coding
Foxl1-201	8	0.14	27.63	0.3	ENSMUST00000181609	protein coding
Gata1-201	X	13.38	1.6	0.02	ENSMUST00000033502	protein coding
Gata1-202	X	0.02	-6.17	NaN	ENSMUST00000125418	protein coding
Gata1-203	X	0.24	-1.44	0.81	ENSMUST00000128449	protein coding
Gata2-203	6	0.11	-17.04	0.17	ENSMUST00000203480	protein coding
Gata3-202	2	0.33	16.77	0.38	ENSMUST00000130615	protein coding
Gcm1-201	9	0.62	-1.89	2.40E-01	ENSMUST00000024104	protein coding
Gfi1b-201	2	0.64	3.3	0.27	ENSMUST00000028156	protein coding
Gfi1b-204	2	0.52	71.33	0.18	ENSMUST00000164290	protein coding
Glis1-202	4	0.17	-5.51	0.2	ENSMUST00000106738	protein coding

Glis1-205	4	0.13	4.88	0.6	ENSMUST00000135835	protein coding
Glis3-201	19	0.41	1.74	0.49	ENSMUST00000065113	nonsense mediated decay
Glis3-202	19	0.03	8.19	0.52	ENSMUST00000112612	protein coding
Glis3-208	19	0.01	-6.17	NaN	ENSMUST00000161328	protein coding
Gm28047-201	15	0.52	3.68	0.36	ENSMUST00000184772	nonsense mediated decay
Grhl2-201	15	35.19	1.21	0.24	ENSMUST00000022895	protein coding
Grhl2-202	15	0.1	6.63	0.55	ENSMUST00000159221	protein coding
Grhl2-203	15	1.28	195.6	0.07	ENSMUST00000161405	protein coding
Grhl2-204	15	1.57	76.87	0.16	ENSMUST00000161532	protein coding
Gsx1-201	5	1.05	1.42	0.61	ENSMUST00000065382	protein coding
Gtf2ird2-201	5	4.08	-1.79	8.41E-04	ENSMUST00000016086	protein coding
Gtf2ird2-202	5	0.05	-1.39	0.83	ENSMUST00000123941	nonsense mediated decay
Gtf2ird2-204	5	0.1	-22.63	0.14	ENSMUST00000135588	protein coding
Hand2-201	8	0.27	1.07	0.97	ENSMUST00000040104	protein coding
Hes5-201	4	0.5	-1.5	0.65	ENSMUST00000049621	protein coding
Hes7-201	11	1.1	1.27	0.75	ENSMUST00000024543	protein coding
Hic1-202	11	2.30E+00	3.18	0.06	ENSMUST00000131720	protein coding
Hic2-201	16	81.06	-1.64	2.20E-08	ENSMUST00000090190	protein coding
Hif3a-201	7	0.09	2.4	0.6	ENSMUST00000037762	protein coding
Hif3a-202	7	24.07	1.36	0.06	ENSMUST00000108492	protein coding
Hif3a-203	7	1.01	-1.28	0.85	ENSMUST00000139224	protein coding
Hif3a-204	7	0.07	-2.25	0.62	ENSMUST00000153833	protein coding
Hivep1-203	13	0.02	-6.17	NaN	ENSMUST00000220801	nonsense mediated decay
Hivep2-201	10	1.04	-1.23	0.64	ENSMUST00000015645	protein coding
Hivep2-203	10	0.17	22.99	0.35	ENSMUST00000186989	protein coding
Hivep2-204	10	0.04	-6.47	2.10E-01	ENSMUST00000187083	protein coding

Hivep2-205	10	0.08	56.78	0.24	ENSMUST00000191138	protein coding
Hivep3-201	4	4.98E-03	-6.17	NaN	ENSMUST00000084306	protein coding
Hivep3-202	4	0.23	1.31	0.47	ENSMUST00000106307	protein coding
Hivep3-209	4	0.04	-1.43	0.8	ENSMUST00000166542	protein coding
Hlx-202	1	0.04	-11.41	0.23	ENSMUST00000174257	protein coding
Hmg20b-204	10	1.86	-1.8	0.52	ENSMUST00000122993	protein coding
Hmga1-202	17	2.41	162.8	0.09	ENSMUST00000117254	protein coding
Hmga1-205	17	0.69	54.06	0.22	ENSMUST00000118599	protein coding
Hmga1-208	17	4.42	-1.25	0.77	ENSMUST00000231358	protein coding
Hmga1-209	17	0.35	-18.35	0.05	ENSMUST00000231780	protein coding
Hmga1-210	17	0.56	-3.38	0.25	ENSMUST00000231796	protein coding
Hmga1-212	17	3.49	-2.7	0.06	ENSMUST00000231866	protein coding
Hmga1-213	17	12.68	1.94	0.01	ENSMUST00000231874	protein coding
Hmga1-214	17	18.22	-1.01	0.99	ENSMUST00000232013	protein coding
Hmgn3-201	9	8.88	-1.92	0.07	ENSMUST00000161796	protein coding
Hmgn3-202	9	6.53	-1.56	0.35	ENSMUST00000162246	protein coding
Hmgn3-203	9	2.45	-1.39	0.54	ENSMUST00000185315	protein coding
Hmgn3-205	9	0.45	-2.25	0.6	ENSMUST00000187193	protein coding
Hmx1-202	5	0.05	2.99	NaN	ENSMUST00000172923	protein coding
Hmx2-201	7	0.07	-2.53	0.4	ENSMUST00000051997	protein coding
Hmx2-202	7	0.49	59.82	2.10E-01	ENSMUST00000183219	protein coding
Hmx3-201	7	0.23	3.83	0.39	ENSMUST00000046093	protein coding
Hoxa1-201	6	9.79	-1.07	0.74	ENSMUST00000000964	protein coding
Hoxa1-202	6	2.48	-1.8	0.2	ENSMUST00000120363	protein coding
Hoxa10-201	6	0.35	1.23	0.88	ENSMUST00000121043	protein coding
Hoxa10-202	6	0.67	1.16	0.85	ENSMUST00000125581	protein coding

Hoxa11-201	6	0.57	-1.36	0.63	ENSMUST00000048026	protein coding
Hoxa13-201	6	1.03	1.55	0.54	ENSMUST00000147595	protein coding
Hoxa2-201	6	0.35	3.64	0.36	ENSMUST00000014848	protein coding
Hoxa4-201	6	0.75	-1	1	ENSMUST00000101395	protein coding
Hoxa6-201	6	0.25	1.46	0.79	ENSMUST00000062829	protein coding
Hoxa7-202	6	0.14	10.07	0.48	ENSMUST00000134367	protein coding
Hoxa7-203	6	0.07	4.88	0.6	ENSMUST00000140316	protein coding
Hoxa7-204	6	0.04	2.99	NaN	ENSMUST00000150041	protein coding
Hoxa7-205	6	0.26	39.63	0.26	ENSMUST00000153280	nonsense mediated decay
Hoxa9-201	6	0.15	2.04	0.59	ENSMUST00000048680	protein coding
Hoxa9-202	6	0.08	-1.84	0.66	ENSMUST00000114425	protein coding
Hoxb13-201	11	5.26	1.76	0.19	ENSMUST00000062709	protein coding
Hoxb2-201	11	0.13	1.46	0.79	ENSMUST00000100523	protein coding
Hoxb3-201	11	0.04	6.56	0.55	ENSMUST00000055334	protein coding
Hoxb3-202	11	0.19	3.59	0.36	ENSMUST00000093944	protein coding
Hoxb4-201	11	2.06	3	0.05	ENSMUST00000049241	protein coding
Hoxb6-201	11	0.18	18.31	0.37	ENSMUST00000000704	protein coding
Hoxb6-202	11	0.04	6.63	0.55	ENSMUST00000173432	protein coding
Hoxb7-201	11	1.07	-1.59	0.42	ENSMUST00000049352	protein coding
Hoxb8-201	11	0.2	-1.84	0.55	ENSMUST00000052650	protein coding
Hoxb8-202	11	0.07	4.88	0.6	ENSMUST00000125410	protein coding
Hoxc11-201	15	0.25	1.05	0.98	ENSMUST00000001701	protein coding
Hoxc8-201	15	0.16	3.55	0.42	ENSMUST00000001703	protein coding
Hoxc9-201	15	1.05	2.68	1.30E-01	ENSMUST00000001706	protein coding
Hoxd10-201	2	0.48	5.48	0.2	ENSMUST00000061745	protein coding
Hoxd11-202	2	0.44	37.1	0.26	ENSMUST00000142312	protein coding

Hoxd13-201	2	2.61	-1.23	0.59	ENSMUST00000001872	protein coding
Hoxd8-201	2	0.39	6.5	0.12	ENSMUST00000019749	protein coding
Hoxd8-202	2	0.06	-4.83	0.24	ENSMUST00000074721	protein coding
Hoxd8-203	2	0.04	2.99	NaN	ENSMUST00000151380	protein coding
Hsf4-204	8	0.25	-2.35	0.6	ENSMUST00000173102	nonsense mediated decay
Hsf4-205	8	0.63	1.59	0.71	ENSMUST00000173640	nonsense mediated decay
Hsf4-206	8	0.1	-2.38	0.58	ENSMUST00000173859	protein coding
Hsf4-207	8	0.2	-7.15	0.26	ENSMUST00000174837	protein coding
Irf2-202	8	0.3	16.74	0.38	ENSMUST00000207105	protein coding
Irf2-203	8	0.06	2.99	NaN	ENSMUST00000207571	protein coding
Irf2-204	8	0.08	4.88	0.6	ENSMUST00000208433	protein coding
Irf2-205	8	0.6	18.2	0.37	ENSMUST00000208507	protein coding
Irf4-201	13	0.05	-8.22	0.08	ENSMUST00000021784	protein coding
Irf4-202	13	0.2	1.14	0.9	ENSMUST00000110307	protein coding
Irf6-201	1	14.83	-1.3	0.16	ENSMUST00000076521	protein coding
Irf7-201	7	0.72	103.78	0.14	ENSMUST00000026571	protein coding
Irf7-202	7	0.36	1.56	0.8	ENSMUST00000097952	protein coding
Irf7-203	7	0.5	-1.13	0.89	ENSMUST00000106023	protein coding
Irf7-204	7	0.09	-1.01	1	ENSMUST00000123525	protein coding
Irf7-212	7	0.15	17.83	0.38	ENSMUST00000209899	nonsense mediated decay
Irx1-202	13	0.07	2.99	NaN	ENSMUST00000223379	protein coding
Irx2-203	13	0.42	-5.58	0.23	ENSMUST00000167067	protein coding
Irx2-205	13	1.5	-3.42	0.18	ENSMUST00000172353	protein coding
Irx4-201	13	1.86	295.31	0.05	ENSMUST00000022095	protein coding
Irx5-202	8	0.32	60.65	0.22	ENSMUST00000210246	protein coding
Isl1-201	13	0.98	1	0.99	ENSMUST00000036060	protein coding

Isl1-202	13	0.08	-3.82	0.37	ENSMUST00000176044	protein coding
Isl2-202	9	0.61	3.67	0.39	ENSMUST00000114290	nonsense mediated decay
Isl2-203	9	9.06E-03	-6.17	NaN	ENSMUST00000164373	nonsense mediated decay
Jazf1-201	6	1.43	1.44	0.33	ENSMUST00000074541	protein coding
Jund-201	8	98.14	1.26	0.22	ENSMUST00000095267	protein coding
Klf6-201	13	46.91	-1.56	8.98E-04	ENSMUST00000000080	protein coding
Kmt2a-201	9	34.64	-1.89	1.37E-13	ENSMUST00000002095	protein coding
Kmt2a-203	9	0.01	4.88	0.6	ENSMUST00000128768	protein coding
Kmt2a-204	9	4.61	-2.27	0.29	ENSMUST00000138119	nonsense mediated decay
Kmt2a-206	9	0.41	-10.15	0.09	ENSMUST00000215489	protein coding
L3mbtl3-201	10	10.95	1.32	0.16	ENSMUST00000040219	protein coding
L3mbtl3-202	10	0.26	87.5	0.16	ENSMUST00000105519	protein coding
L3mbtl3-203	10	2.3	-2.51	0.06	ENSMUST00000174766	protein coding
Lbx2-201	6	1.09	-1.17	0.86	ENSMUST00000041265	protein coding
Lhx1-201	11	0.73	2.49	0.22	ENSMUST00000018842	protein coding
Lhx1-204	11	0.08	8.67	0.5	ENSMUST00000184646	protein coding
Lhx2-207	2	0.3	13.31	0.42	ENSMUST00000176229	nonsense mediated decay
Lhx4-201	1	0.12	7.21	0.21	ENSMUST00000027740	protein coding
Lhx5-201	5	0.51	1.02	0.98	ENSMUST00000031591	protein coding
Lhx6-201	2	0.78	1.1	0.99	ENSMUST00000112960	protein coding
Lhx6-203	2	0.24	62.85	0.22	ENSMUST00000112963	protein coding
Lhx6-205	2	0.2	49.53	0.24	ENSMUST00000112967	protein coding
Lhx9-202	1	0.23	2.09	0.48	ENSMUST00000046870	protein coding
Lhx9-203	1	0.03	14.11	0.41	ENSMUST00000093486	protein coding
Lhx9-204	1	0.09	1.28	0.88	ENSMUST00000112026	protein coding
Lhx9-207	1	0.01	-6.17	NaN	ENSMUST00000194557	nonsense mediated decay

Lmx1b-203	2	1.17	128.69	0.11	ENSMUST00000176067	protein coding
Ltf-201	9	0.5	8.32	0.09	ENSMUST00000035077	protein coding
Ltf-211	9	0.18	5.1	NaN	ENSMUST00000199815	nonsense mediated decay
Maf-201	8	0.02	5.1	NaN	ENSMUST00000069009	protein coding
Maf-202	8	0.35	2.57	0.13	ENSMUST00000109104	protein coding
Mafb-201	2	0.48	1.62	0.53	ENSMUST00000099126	protein coding
Maff-203	15	0.17	25.61	0.34	ENSMUST00000229130	protein coding
Maff-204	15	0.16	-2.09	0.57	ENSMUST00000229285	protein coding
Mafk-202	5	1.88	-1.62	0.29	ENSMUST00000110836	protein coding
Mbnl2-201	14	60.53	1.05	0.71	ENSMUST00000088419	protein coding
Mbnl2-203	14	1.4	1.11	0.91	ENSMUST00000226800	protein coding
Mef2a-202	7	0.27	-7.25	0.18	ENSMUST00000072460	nonsense mediated decay
Mef2a-203	7	1.14	-3.34	0.25	ENSMUST00000076325	protein coding
Mef2a-204	7	4.03	-1.59	0.07	ENSMUST00000107476	protein coding
Mef2a-206	7	0.35	-8.77	0.35	ENSMUST00000135493	nonsense mediated decay
Mef2a-208	7	5.88	-1.37	0.15	ENSMUST00000156690	protein coding
Mef2d-202	3	3.37	-1.4	0.35	ENSMUST00000107558	protein coding
Mef2d-204	3	41.04	1.15	0.51	ENSMUST00000119251	protein coding
Meis1-209	11	0.47	9.74	0.08	ENSMUST00000185131	protein coding
Meis3-201	7	8.19	-1.05	0.91	ENSMUST00000002495	protein coding
Meis3-203	7	0.63	31.98	0.29	ENSMUST00000176342	protein coding
Meis3-204	7	0.18	-1.18	0.91	ENSMUST00000176446	nonsense mediated decay
Meis3-207	7	0.08	-49.51	0.06	ENSMUST00000177156	nonsense mediated decay
Mnx1-201	5	0.78	4.52	0.06	ENSMUST00000165512	protein coding
Msx2-201	13	21.09	1.84	1.06E-03	ENSMUST00000021922	protein coding
Mxd4-201	5	5.48	-1.9	0.08	ENSMUST00000042701	protein coding

Mycl-202	4	25.38	-1.71	2.09E-04	ENSMUST00000106252	protein coding
Mycl-204	4	0.06	2.99	NaN	ENSMUST00000144998	protein coding
Mypop-201	7	10.34	1.95	0.24	ENSMUST00000059331	protein coding
Mypop-203	7	2.12	-3.02	0.15	ENSMUST00000131087	protein coding
Nfatc1-202	18	1	3.63	0.68	ENSMUST00000078049	protein coding
Nfatc2-201	2	0.55	-1.7	0.53	ENSMUST00000029057	protein coding
Nfatc2-204	2	4.36	-1.39	0.35	ENSMUST00000109184	protein coding
Nfatc2-205	2	0.16	27.84	0.32	ENSMUST00000137451	protein coding
Nfia-201	4	0.11	10.75	0.17	ENSMUST00000052018	protein coding
Nfia-202	4	0.06	42.92	0.26	ENSMUST00000075448	protein coding
Nfia-205	4	0.03	1.66	0.77	ENSMUST00000107062	protein coding
Nfia-207	4	0.05	-3.72	0.37	ENSMUST00000148930	protein coding
Nfia-208	4	0.16	12.46	0.43	ENSMUST00000152023	protein coding
Nfic-201	10	10.91	1.18	0.72	ENSMUST00000020461	protein coding
Nfic-202	10	0.33	45.69	0.33	ENSMUST00000078185	protein coding
Nfic-204	10	1.2	2.72	0.27	ENSMUST00000117966	protein coding
Nfix-201	8	0.91	1.83	0.57	ENSMUST00000076715	protein coding
Nfix-202	8	1.01	129.03	0.11	ENSMUST00000099070	protein coding
Nfix-203	8	0.07	14.11	0.41	ENSMUST00000109762	protein coding
Nfkb2-202	19	13.48	2.09	0.07	ENSMUST00000111881	protein coding
Nfkb2-204	19	0.35	6.01	0.24	ENSMUST00000236591	protein coding
Nfkb2-206	19	2.23	-1.27	0.54	ENSMUST00000237330	protein coding
Nfkb2-208	19	0.02	-22.46	0.14	ENSMUST00000237791	nonsense mediated decay
Nkx1-1-201	5	1.43	126.04	0.12	ENSMUST00000173348	protein coding
Nkx2-2-201	2	0.11	19.76	0.36	ENSMUST00000067075	protein coding
Nkx2-2-203	2	0.19	16.25	0.38	ENSMUST00000109970	protein coding

Nkx2-3-201	19	0.33	1.16	0.91	ENSMUST00000057178	protein coding
Nkx2-5-201	17	1.59	-1.01	0.99	ENSMUST00000015723	protein coding
Nkx2-9-201	12	4.02	1.91	0.18	ENSMUST00000072631	protein coding
Npas1-201	7	0.52	80.7	0.16	ENSMUST00000002053	protein coding
Npas2-201	1	2.28	1.91	0.18	ENSMUST00000056815	protein coding
Npas2-203	1	0.33	14.11	0.41	ENSMUST00000173050	protein coding
Nr1h3-201	2	0.18	-4.94	0.29	ENSMUST00000002177	protein coding
Nr1h3-202	2	1.56	-2.31	0.3	ENSMUST00000111354	protein coding
Nr1h3-203	2	0.1	12.77	0.42	ENSMUST00000111355	protein coding
Nr1h3-204	2	0.8	115.77	0.12	ENSMUST00000111356	protein coding
Nr2e1-201	10	0.07	2.98	0.48	ENSMUST00000019938	protein coding
Nr2f1-201	13	0.06	1.31	0.84	ENSMUST00000091458	protein coding
Nr2f1-205	13	0.03	6.56	0.55	ENSMUST00000150498	protein coding
Nr2f2-202	7	0.22	4.24	0.41	ENSMUST00000089565	protein coding
Nr2f2-204	7	0.78	37.87	0.28	ENSMUST00000208081	protein coding
Nr2f6-201	8	33.77	1.1	0.65	ENSMUST00000002466	protein coding
Nr2f6-205	8	0.48	30.47	0.3	ENSMUST00000137058	protein coding
Nr4a3-201	4	0.02	11.79	0.45	ENSMUST00000030025	protein coding
Nr4a3-203	4	0.07	-2.7	0.49	ENSMUST00000153369	protein coding
Nr5a1-201	2	0.1	23.52	3.40E-01	ENSMUST00000028084	protein coding
Nr5a1-202	2	0.13	12.32	0.45	ENSMUST00000112883	protein coding
Olig1-201	16	3.23	2.66	0.07	ENSMUST00000056882	protein coding
Onecut3-201	10	0.14	50.37	0.22	ENSMUST00000051773	protein coding
Otx1-202	11	0.04	-11.42	NaN	ENSMUST00000147486	protein coding
Ovol2-201	2	12.08	-1.44	0.04	ENSMUST00000037423	protein coding
Pax3-202	1	0.99	289	0.05	ENSMUST00000087086	protein coding

Pax7-201	4	0.22	-1.11	0.88	ENSMUST00000030508	protein coding
Pax7-202	4	0.04	6.63	0.55	ENSMUST00000174681	protein coding
Pax9-203	12	0.75	236.54	0.07	ENSMUST00000153250	protein coding
Pbx3-203	2	0.75	2.07	0.58	ENSMUST00000127353	protein coding
Pbx3-204	2	0.64	21.94	0.34	ENSMUST00000138021	protein coding
Pbx3-205	2	0.22	8.67	0.5	ENSMUST00000141653	protein coding
Pbx3-209	2	0.73	-2.26	0.56	ENSMUST00000175855	nonsense mediated decay
Pbx4-201	8	4.11	1.67	0.32	ENSMUST00000081503	protein coding
Pbx4-202	8	1.14	1.09	0.96	ENSMUST00000131637	protein coding
Pbx4-204	8	0.98	3.76	0.35	ENSMUST00000132899	nonsense mediated decay
Pbx4-209	8	1	5.52	2.20E-01	ENSMUST00000156319	nonsense mediated decay
Pdx1-201	5	0.68	-1.03	0.97	ENSMUST00000085591	protein coding
Peg3-202	7	0.28	17.07	0.38	ENSMUST00000143703	nonsense mediated decay
Peg3-203	7	0.25	60.24	0.22	ENSMUST00000150182	nonsense mediated decay
Pgr-201	9	0.07	-1.12	0.94	ENSMUST00000070463	protein coding
Pgr-202	9	0.04	-1.18	0.91	ENSMUST00000098986	protein coding
Pgr-204	9	1.30E-01	-1.24	0.83	ENSMUST00000189181	protein coding
Phox2a-201	7	8.04	1.29	0.59	ENSMUST00000008090	protein coding
Pitx3-201	19	0.58	2.02	0.53	ENSMUST00000026259	protein coding
Pitx3-202	19	0.21	-25.65	0.12	ENSMUST00000172971	protein coding
Pogk-201	1	0.04	-11.41	0.23	ENSMUST00000127596	protein coding
Pogk-202	1	1.55	2.16	0.3	ENSMUST00000128861	protein coding
Pogk-203	1	0.8	499.99	0.08	ENSMUST00000131487	protein coding
Pogk-204	1	0.23	1.39	0.82	ENSMUST00000135673	protein coding
Pogk-205	1	0.4	-2.11	0.33	ENSMUST00000148243	nonsense mediated decay
Pogk-206	1	1.7	-1.82	0.08	ENSMUST00000169324	protein coding

Pou3f3-202	1	0.05	-2.26	0.56	ENSMUST00000239323	protein coding
Ppard-201	17	40.17	-1.8	7.98E-05	ENSMUST00000002320	protein coding
Ppard-205	17	0.24	-7.86	0.17	ENSMUST00000166744	protein coding
Ppard-206	17	3.01	217.1	0.07	ENSMUST00000169040	protein coding
Pparg-201	6	0.45	60.24	0.23	ENSMUST00000000450	protein coding
Pparg-202	6	1.79	285.86	0.05	ENSMUST00000171644	protein coding
Pparg-204	6	1.08	146.7	0.1	ENSMUST00000203732	protein coding
Pparg-206	6	0.06	-11.41	0.23	ENSMUST00000204305	protein coding
Pparg-207	6	0.04	2.99	NaN	ENSMUST00000205213	protein coding
Prdm6-205	18	1.2	9.18	0.2	ENSMUST00000236096	protein coding
Prdm8-201	5	4.93E-03	2.99	NaN	ENSMUST00000112959	protein coding
Prdm8-202	5	0.26	63.64	0.21	ENSMUST00000210477	protein coding
Prox1-201	1	0.54	-1.12	0.9	ENSMUST00000010319	protein coding
Prox1-203	1	0.02	-27.96	0.11	ENSMUST00000177288	protein coding
Prox2-201	12	0.03	-2.91	0.49	ENSMUST00000110249	protein coding
Prox2-202	12	0.83	1.1	0.82	ENSMUST00000177289	protein coding
Prrx1-201	1	0.16	2.4	0.54	ENSMUST00000027878	protein coding
Prrx1-202	1	0.06	23.88	0.35	ENSMUST00000075805	protein coding
Prrx1-204	1	0.03	-1.12	0.94	ENSMUST00000174397	protein coding
Purb-201	11	60.16	-1.7	2.34E-08	ENSMUST00000179343	protein coding
Purg-202	8	5.49	-1.37	0.46	ENSMUST00000078058	protein coding
Rbpjl-201	2	0.19	5.4	0.3	ENSMUST00000017151	protein coding
Rel-201	11	5.44	-1.17	0.42	ENSMUST00000102864	protein coding
Runx1-201	16	1.03	1.11	0.85	ENSMUST00000023673	protein coding
Runx1-202	16	4	1.71	0.22	ENSMUST00000113956	protein coding
Runx1-205	16	0.06	4.88	0.6	ENSMUST00000187242	protein coding

Runx2-201	17	0.03	-16.19	0.17	ENSMUST00000113568	nonsense mediated decay
Runx2-203	17	0.16	76.94	0.16	ENSMUST00000113572	protein coding
Runx2-204	17	6.00E-03	-6.17	NaN	ENSMUST00000159943	protein coding
Runx2-207	17	0.01	2.99	NaN	ENSMUST00000160673	protein coding
Runx2-209	17	0.05	2.99	NaN	ENSMUST00000162130	protein coding
Runx2-210	17	0.15	17.83	0.38	ENSMUST00000162373	protein coding
Runx2-212	17	0.02	-2.36	0.59	ENSMUST00000162816	nonsense mediated decay
Runx2-213	17	0.02	4.88	0.6	ENSMUST00000162878	protein coding
Runx2-214	17	0.1	15.54	0.39	ENSMUST00000238400	protein coding
Runx3-201	4	3.04	-1.69	0.09	ENSMUST00000056977	protein coding
Runx3-202	4	0.44	62.77	0.21	ENSMUST00000119564	protein coding
Rxra-201	2	71.58	1.42	8.98E-04	ENSMUST00000077257	protein coding
Rxra-202	2	2.96	-3.46	0.19	ENSMUST00000100251	protein coding
Rxra-203	2	1.73	257.92	0.07	ENSMUST00000113934	protein coding
Rxra-204	2	0.11	5.1	NaN	ENSMUST00000129514	protein coding
Rxra-206	2	0.2	23.83	0.33	ENSMUST00000238859	protein coding
Sall2-202	14	11.89	-1.14	6.20E-01	ENSMUST00000135523	protein coding
Satb1-201	17	0.18	-11.47	0.14	ENSMUST00000124222	protein coding
Satb1-204	17	20.97	1.35	0.37	ENSMUST00000133574	protein coding
Satb1-207	17	5.04	-1.74	0.04	ENSMUST00000140979	protein coding
Satb1-211	17	1.52	1.7	0.23	ENSMUST00000152830	protein coding
Satb1-214	17	1.26	2.25	0.26	ENSMUST00000176669	protein coding
Satb2-203	1	0.41	12.46	0.43	ENSMUST00000176759	protein coding
Satb2-205	1	0.2	-2.59	0.42	ENSMUST00000177424	protein coding
Scmh1-202	4	0.75	-1.17	0.89	ENSMUST00000064991	protein coding
Scmh1-205	4	0.13	5.1	NaN	ENSMUST00000122860	protein coding

Scmh1-206	4	4.44	1.4	0.49	ENSMUST00000132116	protein coding
Scx-201	15	2.78	1.98	0.3	ENSMUST00000043089	protein coding
Setbp1-201	18	0.69	-1.98	0.03	ENSMUST00000025430	protein coding
Shox2-201	3	0.17	1.31	0.87	ENSMUST00000029422	protein coding
Shox2-203	3	0.47	2.66	0.35	ENSMUST00000162098	protein coding
Sim1-201	10	0.1	8.69	0.22	ENSMUST00000020071	protein coding
Sim1-202	10	0.04	6.78	0.54	ENSMUST00000219436	protein coding
Sim2-203	16	0.01	-16.66	0.16	ENSMUST00000231688	nonsense mediated decay
Six3-201	17	0.02	4.88	0.6	ENSMUST00000162695	protein coding
Smad3-201	9	45.39	1.33	0.05	ENSMUST00000034973	protein coding
Smad3-202	9	0.58	18.15	0.36	ENSMUST00000133108	protein coding
Sox10-202	15	0.04	2.99	NaN	ENSMUST00000230261	protein coding
Sox10-203	15	0.6	125.47	0.12	ENSMUST00000230532	protein coding
Sox14-201	9	0.14	24.24	0.34	ENSMUST00000054819	protein coding
Sox14-202	9	0.03	2.99	NaN	ENSMUST00000183065	protein coding
Sox15-201	11	42.83	-1.83	0.02	ENSMUST00000047373	protein coding
Sox18-201	2	2.36	1.28	0.65	ENSMUST00000054491	protein coding
Sox30-201	11	0.19	-1.76	0.51	ENSMUST00000049038	protein coding
Sox8-201	17	0.9	-2.02	0.09	ENSMUST00000025003	protein coding
Sox8-202	17	0.07	5.1	NaN	ENSMUST00000173447	protein coding
Sox8-203	17	0.06	-20.91	0.14	ENSMUST00000174560	protein coding
Sp100-201	1	0.09	12.77	0.42	ENSMUST00000054279	protein coding
Sp100-202	1	0.07	15.1	0.4	ENSMUST00000066427	protein coding
Sp100-204	1	0.14	14.36	0.4	ENSMUST00000132641	protein coding
Sp100-209	1	0.03	8.67	0.5	ENSMUST00000145440	nonsense mediated decay
Sp100-212	1	0.08	11.79	0.45	ENSMUST00000153574	protein coding

Sp100-213	1	0.04	5.1	NaN	ENSMUST0000015 5094	protein coding
Sp4-201	12	11.16	1.24	0.46	ENSMUST0000002 6367	protein coding
Sp4-202	12	1.74	52.24	0.22	ENSMUST0000022 1844	protein coding
Srebf2-201	15	195.01	-1.74	4.21E-09	ENSMUST0000002 3100	protein coding
Srebf2-206	15	1.52	1.82	0.57	ENSMUST0000023 0955	protein coding
Srf-201	17	63.42	-1.59	6.82E-03	ENSMUST0000001 5749	protein coding
Srf-202	17	4.68	2.82	0.09	ENSMUST0000023 3104	protein coding
Stat5a-203	11	3.28	-1.6	0.29	ENSMUST0000010 7357	protein coding
Stat5a-206	11	2.66	120.52	0.12	ENSMUST0000013 8083	protein coding
Stat5b-201	11	19.74	-1.15	0.37	ENSMUST0000000 4143	protein coding
Stat5b-202	11	0.33	130.73	0.12	ENSMUST0000010 7358	protein coding
Tbr1-201	2	0.19	3.29	0.41	ENSMUST0000004 8934	protein coding
Tbx1-201	16	0.88	123.82	0.12	ENSMUST0000000 9241	protein coding
Tbx1-202	16	0.4	1.62	0.78	ENSMUST0000023 2143	nonsense mediated decay
Tbx1-203	16	0.04	8.35	0.51	ENSMUST0000023 2335	protein coding
Tbx20-201	9	1.27	-1.9	0.02	ENSMUST0000005 2946	protein coding
Tbx20-202	9	0.26	-5.65	0.15	ENSMUST0000016 6018	protein coding
Tbx20-203	9	0.1	-16.96	0.18	ENSMUST0000021 5802	protein coding
Tbx21-201	11	1.56	-1.54	0.49	ENSMUST0000000 1484	protein coding
Tbx4-201	11	0.35	79.13	1.90E-01	ENSMUST0000000 0096	protein coding
Tbx4-203	11	1.06	250.61	0.06	ENSMUST0000010 8047	protein coding
Tcf24-202	1	2.79	1.43	0.36	ENSMUST0000018 5184	protein coding
Tcf7-203	11	16.36	-1	1	ENSMUST0000023 8914	protein coding
Tcf7l1-201	6	91.46	1.07	0.59	ENSMUST0000006 9536	protein coding
Tcf7l1-202	6	38.24	1.79	0.04	ENSMUST0000011 4053	protein coding
Tcf7l1-204	6	0.79	1.25	8.70E-01	ENSMUST0000014 9446	protein coding

Tead3-205	17	0.68	142.61	0.1	ENSMUST00000154873	protein coding
Tead3-207	17	2.84	2.86	0.15	ENSMUST00000219703	protein coding
Tead4-201	6	57.15	1.04	0.84	ENSMUST00000006311	protein coding
Tead4-202	6	0.15	22.99	0.35	ENSMUST00000112157	protein coding
Tead4-204	6	0.61	3.83	0.4	ENSMUST00000133118	protein coding
Tead4-205	6	0.06	2.99	NaN	ENSMUST00000143004	protein coding
Tet3-202	6	12.65	1.52	5.53E-03	ENSMUST00000089622	protein coding
Tet3-203	6	3.48	-1.22	0.35	ENSMUST00000186548	protein coding
Tfap2c-206	2	27.24	2.03	0.34	ENSMUST00000170744	protein coding
Tfap2e-201	4	1.58	1.29	0.5	ENSMUST00000048194	protein coding
Tgif2-201	2	31.51	-1.45	0.05	ENSMUST00000073352	protein coding
Tgif2-202	2	89.19	-1.54	1.03E-04	ENSMUST00000081335	protein coding
Tgif2-203	2	6	-1.38	0.55	ENSMUST00000150078	nonsense mediated decay
Thap3-201	4	29.62	-1.28	0.26	ENSMUST00000036680	protein coding
Thap3-202	4	1.63	-2.33	0.2	ENSMUST00000105665	protein coding
Tlx1-201	19	1.72	-1.01	0.99	ENSMUST00000026236	protein coding
Trafd1-201	5	20.52	1.78	0.02	ENSMUST00000042312	protein coding
Trafd1-206	5	3.86	177.43	0.08	ENSMUST00000152265	protein coding
Trafd1-207	5	0.18	6.78	0.55	ENSMUST00000155379	protein coding
Trafd1-208	5	5.3	1.56	3.80E-01	ENSMUST00000156158	nonsense mediated decay
Trafd1-209	5	0.81	-2.15	0.41	ENSMUST00000202064	protein coding
Tshz3-201	7	1.8	-1.55	0.35	ENSMUST00000021641	protein coding
Twist1-201	12	1.64	-1.99	0.19	ENSMUST00000049089	protein coding
Twist2-201	1	2.71	270.09	0.06	ENSMUST00000007949	protein coding
Twist2-202	1	0.02	4.88	0.6	ENSMUST00000186075	protein coding
Uncx-201	5	0.1	2.98	0.47	ENSMUST00000172997	protein coding

Uncx-202	5	0.02	2.99	NaN	ENSMUST0000017 4792	protein coding
Vax2-201	6	5.47	1.24	0.67	ENSMUST0000003 7807	protein coding
Vdr-202	15	0.21	6.78	0.55	ENSMUST0000012 6568	protein coding
Vezf1-201	11	52.65	-1.25	0.05	ENSMUST0000001 8521	protein coding
Wt1-201	2	3.97	-1.42	4.90E-01	ENSMUST0000011 1098	protein coding
Wt1-207	2	0.6	3.21	0.48	ENSMUST0000014 6842	protein coding
Xpa-201	4	18.28	-1.84	2.80E-03	ENSMUST0000003 0013	protein coding
Xpa-202	4	0.58	-2.05	0.53	ENSMUST0000005 8232	protein coding
Xpa-206	4	0.78	2.77	0.48	ENSMUST0000014 2380	protein coding
Zbtb16-201	9	0.65	-2.37	0.07	ENSMUST0000009 3852	protein coding
Zbtb16-202	9	0.3	57.68	0.23	ENSMUST0000021 6150	protein coding
Zbtb20-201	16	0.08	18.7	0.37	ENSMUST0000007 9441	protein coding
Zbtb20-203	16	0.1	23.85	0.35	ENSMUST0000011 4691	protein coding
Zbtb20-204	16	0.31	-1.7	2.40E-01	ENSMUST0000011 4694	protein coding
Zbtb20-205	16	0.07	-1.1	0.96	ENSMUST0000011 4695	protein coding
Zbtb20-216	16	0.03	-6.17	NaN	ENSMUST0000014 8775	protein coding
Zbtb20-217	16	0.03	2.99	NaN	ENSMUST0000015 1244	protein coding
Zbtb20-219	16	0.02	-6.17	NaN	ENSMUST0000015 6367	protein coding
Zbtb4-201	11	0.52	7.77	0.13	ENSMUST0000010 8638	protein coding
Zbtb4-202	11	2.31	1.07	0.83	ENSMUST0000010 8639	protein coding
Zbtb4-204	11	0.02	-6.17	NaN	ENSMUST0000010 8642	protein coding
Zbtb42-202	12	0.19	-1.44	0.81	ENSMUST0000017 3942	protein coding
Zbtb46-201	2	4.84	-1.02	0.95	ENSMUST0000002 9106	protein coding
Zbtb46-205	2	0.17	-8.71	0.1	ENSMUST0000018 0222	protein coding
Zbtb7b-204	3	0.62	2.16	0.29	ENSMUST0000010 7435	protein coding
Zbtb7b-205	3	7.44	-2.13	0.1	ENSMUST0000012 4783	protein coding

Zbtb7b-206	3	0.75	2.37	0.51	ENSMUST00000126027	protein coding
Zbtb7b-209	3	4.07	146.92	0.09	ENSMUST00000148361	protein coding
Zfhx3-201	8	6.71	1.69	3.63E-03	ENSMUST00000043896	protein coding
Zfhx3-202	8	0.34	29.53	0.29	ENSMUST00000188994	protein coding
Zfp276-201	8	10.72	-1.58	0.09	ENSMUST00000001092	protein coding
Zfp276-206	8	0.13	8.35	0.51	ENSMUST00000154450	protein coding
Zfp28-201	7	6.57	-1.23	0.37	ENSMUST00000081022	protein coding
Zfp28-202	7	1.39	4.44	0.2	ENSMUST00000207086	nonsense mediated decay
Zfp28-203	7	0.04	-11.41	0.23	ENSMUST00000207465	protein coding
Zfp28-204	7	2.39	1.02	9.90E-01	ENSMUST00000207809	protein coding
Zfp28-205	7	0.32	-5.87	0.19	ENSMUST00000208338	protein coding
Zfp316-202	5	2.45	-1.43	0.06	ENSMUST00000161448	protein coding
Zfp319-201	8	4.83	-1.6	0.09	ENSMUST00000057717	protein coding
Zfp334-201	2	13.85	-1.97	1.46E-06	ENSMUST00000103084	protein coding
Zfp358-201	8	15.18	-1.14	0.71	ENSMUST00000061508	protein coding
Zfp358-203	8	0.36	3.56	0.36	ENSMUST00000208423	protein coding
Zfp362-204	4	1.05	87.45	0.15	ENSMUST00000170934	protein coding
Zfp382-201	7	19.06	-1.85	0.02	ENSMUST00000098596	protein coding
Zfp382-202	7	2.72	-2.02	0.4	ENSMUST00000153792	protein coding
Zfp385c-201	11	1.36	-1.38	0.6	ENSMUST00000103119	protein coding
Zfp385c-203	11	0.27	16.25	0.38	ENSMUST00000151589	protein coding
Zfp385c-204	11	0.68	21.89	0.34	ENSMUST00000153494	protein coding
Zfp429-203	13	0.34	14.33	0.41	ENSMUST00000224684	protein coding
Zfp46-201	4	5.76	-1.68	0.03	ENSMUST00000069195	protein coding
Zfp46-203	4	1.02	-2.83	0.06	ENSMUST00000130658	protein coding
Zfp467-201	6	0.26	13.1	4.30E-01	ENSMUST00000101443	protein coding

Zfp467-202	6	0.16	-1.44	0.8	ENSMUST00000114556	protein coding
Zfp467-203	6	0.01	-16.66	0.15	ENSMUST00000114558	protein coding
Zfp467-205	6	0.09	1.79	0.73	ENSMUST00000114560	protein coding
Zfp467-207	6	0.2	1.29	0.88	ENSMUST00000114563	protein coding
Zfp467-209	6	0.61	-3.82	0.38	ENSMUST00000114566	protein coding
Zfp467-211	6	0.1	-16.96	0.18	ENSMUST00000141449	nonsense mediated decay
Zfp469-201	8	0.17	2.41	0.59	ENSMUST00000055537	protein coding
Zfp524-201	7	9.69	-1.2	0.82	ENSMUST00000086349	protein coding
Zfp524-202	7	2.50E-01	-12.32	0.08	ENSMUST00000207901	protein coding
Zfp524-203	7	0.78	7.45	0.21	ENSMUST00000209030	protein coding
Zfp575-201	7	0.99	1.17	0.63	ENSMUST00000094705	protein coding
Zfp579-206	7	7.71	-1.06	0.8	ENSMUST00000162731	protein coding
Zfp641-201	15	0.01	-16.66	0.15	ENSMUST00000023722	protein coding
Zfp651-203	9	1.89	1.58	5.30E-01	ENSMUST00000214732	nonsense mediated decay
Zfp697-201	3	2.37	1.26	0.51	ENSMUST00000056096	protein coding
Zfp703-201	8	0.38	16.26	0.38	ENSMUST00000127097	protein coding
Zfp703-203	8	1.15	30.98	0.28	ENSMUST00000209411	protein coding
Zfp708-201	13	0.95	1.26	0.78	ENSMUST00000109742	protein coding
Zfp768-201	7	7.6	-1.73	2.91E-03	ENSMUST00000060783	protein coding
Zfp768-202	7	0.29	55.61	0.22	ENSMUST00000205266	protein coding
Zfp771-201	7	16.94	1.71	0.03	ENSMUST00000052509	protein coding
Zfp771-203	7	0.38	-6.05	0.09	ENSMUST00000205583	protein coding
Zfp773-202	7	6.81	-1.18	0.63	ENSMUST00000211240	protein coding
Zfp787-201	7	11.76	-1.55	0.2	ENSMUST00000094870	protein coding
Zfp787-202	7	0.31	2.42	0.55	ENSMUST00000207315	protein coding
Zfp787-206	7	0.55	-1.97	0.6	ENSMUST00000208746	protein coding

Zfp791-202	8	6.05	-1.4	0.04	ENSMUST00000211109	protein coding
Zfp820-201	17	14.52	-1.2	0.51	ENSMUST00000084141	protein coding
Zfp865-201	7	3.24	1.01	0.99	ENSMUST00000076251	protein coding
Zfp865-202	7	14.77	-1.07	0.78	ENSMUST00000085427	protein coding
Zfp9-201	6	1.01	-1.19	0.78	ENSMUST00000161170	protein coding
Zfp92-201	X	0.12	2.27	0.55	ENSMUST00000033740	protein coding
Zfp92-202	X	0.02	2.99	NaN	ENSMUST00000086470	protein coding
Zfp947-201	17	4.58	-1.64	0.11	ENSMUST00000080249	protein coding
Zhx2-201	15	1.13	1.16	0.82	ENSMUST00000096430	protein coding
Zhx3-201	2	0.53	2.72	0.19	ENSMUST00000103111	protein coding
Zhx3-202	2	0.72	-1.48	0.48	ENSMUST00000103112	protein coding
Zhx3-203	2	1.54	2.04	0.38	ENSMUST00000109460	protein coding
Zic2-201	14	50.51	1.87	1.90E-04	ENSMUST00000075888	protein coding
Zic4-201	9	0.05	16.25	0.38	ENSMUST00000066384	protein coding
Zic4-204	9	0.06	14.35	0.4	ENSMUST00000172646	protein coding
Zic4-207	9	0.25	14.08	0.41	ENSMUST00000173054	protein coding
Zic4-208	9	0.1	2.99	NaN	ENSMUST00000173342	protein coding
Zic4-209	9	0.03	4.88	0.6	ENSMUST00000173933	protein coding
Zic5-201	14	23.56	-1.09	0.57	ENSMUST00000039118	protein coding
Zscan2-201	7	1.79	-2.49	0.05	ENSMUST00000044115	protein coding
Zscan2-202	7	0.78	1.07	0.97	ENSMUST00000132163	protein coding
Zscan2-203	7	1.03	14.35	0.07	ENSMUST00000147125	protein coding
Zscan2-204	7	0.17	11.67	0.45	ENSMUST00000155128	protein coding
Zscan2-205	7	0.34	1.61	0.77	ENSMUST00000205361	protein coding

S.2. Transcript Variants (TE) of Downregulated Genes (GE)

Table S2-1. Expression values & metadata of upregulated transcript variants of downregulated genes

Name	Chromosome	Max group mean	Fold change	FDR p-value	ENSEMBL	Biotype
Arid3b-202	9	4.69	10.29	1.57E-08	ENSMUST00000098686	protein coding
Arid5a-201	1	2.4	3.44	0.01	ENSMUST00000097778	protein coding
Dpf1-202	7	3.15	32.27	1.63E-09	ENSMUST00000065181	protein coding
Dpf1-204	7	0.69	6.21	0.04	ENSMUST00000108231	protein coding
Fiz1-208	7	2.94	4.83	0.01	ENSMUST00000208944	protein coding
Gli1-204	10	0.53	28.63	2.37E-03	ENSMUST00000219671	protein coding
Gtf2i-202	5	0.32	441.84	1.26E-04	ENSMUST00000082057	protein coding
Gtf2i-203	5	12.45	16,239.01	7.96E-14	ENSMUST00000111261	protein coding
Jdp2-201	12	5.49	804.8	1.83E-13	ENSMUST00000050687	protein coding
Klf10-201	15	43.9	2.05	8.71E-07	ENSMUST00000074043	protein coding
Klf15-201	6	2.12	591.6	1.42E-09	ENSMUST00000032174	protein coding
Msantd3-203	4	10.83	10.04	4.04E-04	ENSMUST00000107704	protein coding
Msantd4-202	9	18.38	3.75	2.39E-24	ENSMUST00000212075	protein coding
Mtf2-201	5	43.09	2	3.96E-08	ENSMUST00000081567	protein coding
Mtf2-203	5	16.61	3.35	5.89E-13	ENSMUST00000124195	nonsense mediated decay
Mycn-201	12	14.72	2.6	2.69E-06	ENSMUST00000043396	protein coding
Nfkb1-201	3	4.23	3.01	7.61E-05	ENSMUST00000029812	protein coding
Phf21a-204	2	9.25	2.28	4.22E-09	ENSMUST00000111293	protein coding
Phf21a-205	2	2.69	4.64	1.02E-11	ENSMUST00000111294	protein coding
Plscr1-201	9	4.65	3.83	1.54E-11	ENSMUST00000093801	protein coding
Prdm16-203	4	0.14	333.92	4.18E-03	ENSMUST00000097759	protein coding
Prdm4-207	10	2.74	6.5	1.80E-11	ENSMUST00000220032	protein coding
Rarg-201	15	12.49	2.49	4.39E-06	ENSMUST00000043172	protein coding

Rarg-205	15	1.27	55.84	0.04	ENSMUST00000135466	protein coding
Rbak-202	5	2.22	8.4	1.02E-05	ENSMUST00000165318	protein coding
Rbpj-201	5	3.92	3.16	6.36E-06	ENSMUST00000037618	protein coding
Rbpj-203	5	33.78	19.85	7.58E-278	ENSMUST00000113865	protein coding
Rest-201	5	32.32	2.62	1.33E-12	ENSMUST00000080359	protein coding
Stat1-205	1	3.37	2.28	0.03	ENSMUST00000186574	protein coding
Tcf4-205	18	2.6	1,698.51	3.03E-08	ENSMUST00000114980	protein coding
Tcf4-225	18	0.59	165.26	4.01E-03	ENSMUST00000201091	protein coding
Tcf4-246	18	1.1	16.22	2.66E-07	ENSMUST00000202116	protein coding
Tcf4-254	18	1.23	5.6	7.52E-03	ENSMUST00000202674	protein coding
Trerf1-203	17	3.74	3.35	4.56E-04	ENSMUST00000190020	protein coding
Trp53-203	11	68.88	17.67	1.98E-32	ENSMUST00000108658	protein coding
Tsc22d1-202	14	7.73	2.46	5.31E-12	ENSMUST00000048371	protein coding
Tsc22d1-204	14	1.61	671.47	7.96E-14	ENSMUST00000110888	protein coding
Tsc22d1-213	14	5.62	5.24	1.96E-19	ENSMUST00000176886	nonsense mediated decay
Usf1-208	1	23.93	2.17	4.32E-08	ENSMUST00000161241	protein coding
Zbed5-202	5	0.66	12.66	5.18E-03	ENSMUST00000077320	protein coding
Zbtb45-201	7	14.28	2.14	7.88E-10	ENSMUST00000051390	protein coding
Zfp109-202	7	1.5	2.66	1.62E-05	ENSMUST00000206362	protein coding
Zfp317-202	9	12.74	2.28	1.11E-09	ENSMUST00000208694	protein coding
Zfp322a-203	13	2.47	4.5	4.02E-05	ENSMUST00000145451	protein coding
Zfp423-203	8	0.18	250.2	5.80E-03	ENSMUST00000165770	protein coding
Zfp438-201	18	0.84	139.45	1.39E-05	ENSMUST00000063989	protein coding
Zfp451-202	1	6.11	4.67	8.11E-19	ENSMUST00000044455	protein coding
Zfp518b-203	5	9.08	2.3	1.19E-03	ENSMUST00000179555	protein coding
Zfp532-204	18	0.27	47.16	0.05	ENSMUST00000182319	protein coding

Zfp553-202	7	7.27	3.29	1.53E-23	ENSMUST00000106312	protein coding
Zfp62-201	11	3.61	2.91	0.01	ENSMUST00000061757	protein coding
Zfp644-213	5	0.14	54.91	0.04	ENSMUST00000137285	protein coding
Zfp644-216	5	0.81	20.9	0.01	ENSMUST00000155495	protein coding
Zfp74-203	7	0.76	6.86	7.07E-03	ENSMUST00000108211	protein coding
Zfp740-207	15	0.43	81.52	0.02	ENSMUST00000133986	protein coding
Zfp827-203	8	0.07	93.27	0.04	ENSMUST00000119254	protein coding
Zfp93-202	7	1.13	5.99	1.86E-03	ENSMUST00000108438	protein coding
Zkscan2-202	7	0.61	50.77	0.02	ENSMUST00000125356	protein coding
Zscan10-202	17	3.38	2,363.72	1.67E-08	ENSMUST00000115509	protein coding
Zscan10-206	17	0.48	123.26	0.02	ENSMUST00000122285	nonsense mediated decay
Zscan18-205	7	2.44	6.79	3.27E-05	ENSMUST00000211392	protein coding
Zup1-207	10	0.91	3.74	2.62E-04	ENSMUST00000219457	nonsense mediated decay

Table S2-2. Expression values & metadata of downregulated transcript variants of downregulated genes						
**Name**	**Chromosome**	**Max group mean**	**Fold change**	**FDR p-value**	**ENSEMBL**	**Biotype**
Atf1-206	15	35	-278.15	2.09E-06	ENSMUST00000168103	nonsense mediated decay
Atf1-209	15	22.99	-32.13	3.64E-09	ENSMUST00000172154	protein coding
Atf5-202	7	15.83	-40.38	7.79E-17	ENSMUST00000107893	protein coding
Bnc2-202	4	11.56	-406.29	2.75E-08	ENSMUST00000107198	protein coding
Csrnp3-203	2	1.77	-41.16	1.78E-03	ENSMUST00000112397	nonsense mediated decay
Csrnp3-204	2	1.95	-22.97	2.51E-09	ENSMUST00000122912	protein coding
Ctcfl-201	2	0.74	-29.5	0.01	ENSMUST00000094287	protein coding
Dmrt1-201	19	51.21	-69.25	7.36E-81	ENSMUST00000025755	protein coding
Etv1-201	12	1.36	-10.7	1.50E-07	ENSMUST00000095767	protein coding
Etv1-202	12	3.04	-35.86	3.94E-05	ENSMUST00000159334	protein coding
Etv5-203	16	124.32	-56.13	4.64E-51	ENSMUST00000168774	protein coding
Foxd3-201	4	10.81	-16.85	2.57E-14	ENSMUST00000087285	protein coding
Foxn4-201	5	4.94	-11.05	1.53E-11	ENSMUST00000044790	protein coding
Gbx1-201	5	1.01	-9.21	7.46E-04	ENSMUST00000088311	protein coding
Gli2-201	1	24.71	-3.02	6.57E-17	ENSMUST00000062483	protein coding
Gm21411-201	4	108.15	-167.81	7.85E-48	ENSMUST00000238583	protein coding
Gm3854-201	7	4.63	-16.41	8.39E-12	ENSMUST00000207173	protein coding
Gm49496-201	13	6.31	-10.88	3.02E-04	ENSMUST00000231096	protein coding
Gtf2i-216	5	17.68	-7.89	2.28E-09	ENSMUST00000173888	protein coding
Gtf2i-222	5	59.07	-3.46	2.38E-05	ENSMUST00000174354	protein coding
Ikzf3-201	11	0.89	-20.74	1.89E-03	ENSMUST00000103141	protein coding
Jdp2-202	12	19.52	-247.62	1.40E-07	ENSMUST00000171754	protein coding
Klf10-202	15	26.02	-57.54	7.89E-21	ENSMUST00000226363	protein coding
Klf2-201	8	66.63	-2.28	2.54E-07	ENSMUST00000067912	protein coding

Klf3-201	5	30.73	-2.03	1.57E-08	ENSMUST00000165536	protein coding
Klf3-204	5	28.4	-3.21	7.06E-03	ENSMUST00000197879	protein coding
Klf4-201	4	163.42	-5.4	2.01E-48	ENSMUST00000107619	protein coding
Klf8-201	X	16.98	-3.1	3.14E-20	ENSMUST00000039545	protein coding
Klf8-202	X	2.08	-5.72	7.34E-03	ENSMUST00000112574	protein coding
Lef1-204	3	3.85	-13.16	2.97E-10	ENSMUST00000106341	protein coding
Lin28b-201	10	9.4	-2.17	0.03	ENSMUST00000079390	protein coding
Meox1-201	11	0.79	-7.57	0.04	ENSMUST00000057054	protein coding
Msantd3-201	4	39.3	-2.79	5.68E-06	ENSMUST00000061135	protein coding
Msantd4-201	9	17.69	-509.34	6.32E-09	ENSMUST00000047173	protein coding
Msc-201	1	24.65	-32.46	6.20E-28	ENSMUST00000027062	protein coding
Msc-204	1	7.13	-6.99	0.01	ENSMUST00000190719	protein coding
Mtf2-202	5	48.53	-153.05	3.66E-26	ENSMUST00000112626	protein coding
Mtf2-207	5	62.02	-17.99	8.31E-83	ENSMUST00000134026	nonsense mediated decay
Mtf2-210	5	62.15	-4.07	8.44E-20	ENSMUST00000143412	nonsense mediated decay
Myb-201	10	0.57	-21.65	0.05	ENSMUST00000020158	protein coding
Myb-203	10	0.93	-28.05	3.33E-03	ENSMUST00000188495	protein coding
Mycn-202	12	39.55	-87.2	2.04E-33	ENSMUST00000130990	protein coding
Nanog-201	6	395.77	-476.14	2.47E-138	ENSMUST00000012540	protein coding
Nanog-202	6	11.89	-3,574.87	0.05	ENSMUST00000112580	protein coding
Neurod1-201	2	2.08	-36.18	1.53E-03	ENSMUST00000041099	protein coding
Nfib-201	4	2.45	-43.58	3.36E-08	ENSMUST00000050872	protein coding
Nkx6-3-201	8	4.57	-44.2	2.07E-06	ENSMUST00000071588	protein coding
Nr5a2-201	1	29.59	-77.36	2.07E-80	ENSMUST00000027649	protein coding
Nr5a2-202	1	4.39	-167.81	2.65E-06	ENSMUST00000168126	protein coding
Nr5a2-203	1	13	-248.86	6.95E-07	ENSMUST00000192357	protein coding

Nr5a2-205	1	33.27	-474.76	2.83E-09	ENSMUST00000192929	protein coding
Otx2-201	14	14.31	-4,545.63	0.04	ENSMUST00000118578	protein coding
Otx2-202	14	2.43	-50.94	2.71E-03	ENSMUST00000119070	protein coding
Pax8-201	2	2.27	-15.2	1.79E-03	ENSMUST00000028355	protein coding
Pou2f3-202	9	4.94	-2.59	3.19E-03	ENSMUST00000176636	protein coding
Pou4f2-201	8	4.55	-178.57	1.36E-06	ENSMUST00000034115	protein coding
Pou5f1-201	17	1,689.61	-234.2	0	ENSMUST00000025271	protein coding
Pou5f1-205	17	3.05	-13.19	8.10E-03	ENSMUST00000173934	protein coding
Prdm1-201	10	1.35	-51.63	9.56E-04	ENSMUST00000039174	protein coding
Prdm14-201	1	22.37	-700.83	3.92E-10	ENSMUST00000047577	protein coding
Prdm16-205	4	0.54	-20.2	0.03	ENSMUST00000105637	protein coding
Prdm5-201	6	8.47	-4.65	5.80E-06	ENSMUST00000031973	protein coding
Rarg-202	15	92.91	-3.34	2.19E-09	ENSMUST00000063339	protein coding
Rbpj-202	5	140.24	-3.39	2.16E-34	ENSMUST00000087360	protein coding
Rex2-201	4	163.11	-29.3	2.61E-160	ENSMUST00000075775	protein coding
Rhox10-201	X	12.89	-33.51	1.90E-07	ENSMUST00000115150	protein coding
Rhox13-201	X	6.94	-26.27	5.13E-04	ENSMUST00000152730	protein coding
Sohlh2-201	3	21.61	-325.8	5.03E-15	ENSMUST00000029369	protein coding
Sox1-201	8	0.79	-6.3	0.02	ENSMUST00000180353	protein coding
Sox11-201	12	2.15	-2.1	0.04	ENSMUST00000079063	protein coding
Sox17-208	1	0.65	-5.64	0.04	ENSMUST00000195555	protein coding
Sox7-201	14	4.78	-2.29	0.05	ENSMUST00000079652	protein coding
Stat1-203	1	2.41	-6.26	6.25E-04	ENSMUST00000186057	protein coding
Stat4-201	1	5.23	-190.98	6.79E-07	ENSMUST00000027277	protein coding
Stat4-202	1	14.29	-463.94	9.77E-09	ENSMUST00000168302	protein coding
Tal1-201	4	0.49	-14.15	0.05	ENSMUST00000030489	protein coding

Tbx3-201	5	34.49	-2.1	7.60E-06	ENSMUST00000018748	protein coding
Tcf23-201	5	0.55	-7.4	0.02	ENSMUST00000006818	protein coding
Tcf4-203	18	5.6	-15.76	9.86E-08	ENSMUST00000114977	protein coding
Terf1-201	1	201.05	-2.03	6.26E-09	ENSMUST00000027057	protein coding
Tet1-201	10	16.86	-4.06	3.68E-03	ENSMUST00000050826	protein coding
Tet2-204	3	38.02	-4.01	4.30E-27	ENSMUST00000196398	protein coding
Tfcp2l1-201	1	158.23	-2.62	1.05E-35	ENSMUST00000027629	protein coding
Tgif1-202	17	135.93	-2.16	8.71E-05	ENSMUST00000118283	protein coding
Tgif1-211	17	8.23	-70.43	3.31E-05	ENSMUST00000186358	protein coding
Trp53-201	11	397.51	-2.11	1.67E-17	ENSMUST00000005371	protein coding
Trps1-201	15	6.4	-55.68	6.49E-20	ENSMUST00000165201	protein coding
Trps1-204	15	6.26	-277.64	3.14E-07	ENSMUST00000183997	protein coding
Trps1-205	15	2.69	-122.11	4.69E-06	ENSMUST00000184458	protein coding
Tsc22d1-214	14	4.45	-38.24	3.62E-03	ENSMUST00000177207	protein coding
Usf1-202	1	11.73	-3.55	9.11E-03	ENSMUST00000159207	protein coding
Zbtb8a-201	4	26.22	-2.28	3.67E-05	ENSMUST00000030610	protein coding
Zeb1-201	18	5.34	-4.17	8.53E-09	ENSMUST00000025081	protein coding
Zfp105-201	9	28.82	-54.71	6.03E-22	ENSMUST00000051667	protein coding
Zfp108-201	7	1.62	-19.82	6.09E-03	ENSMUST00000072713	protein coding
Zfp169-207	13	2.13	-4.35	8.31E-03	ENSMUST00000177530	protein coding
Zfp239-207	6	10.65	-5.61	2.21E-06	ENSMUST00000172088	protein coding
Zfp260-201	7	8.44	-5.33	3.35E-11	ENSMUST00000050735	protein coding
Zfp260-202	7	14.42	-79.07	1.68E-16	ENSMUST00000108200	protein coding
Zfp42-201	8	614.52	-3.31	2.37E-59	ENSMUST00000082120	protein coding
Zfp42-202	8	120.61	-5.84	4.06E-34	ENSMUST00000209356	protein coding
Zfp42-203	8	21.79	-2.68	1.21E-03	ENSMUST00000211248	protein coding

Zfp423-201	8	17.85	-4.8	6.14E-06	ENSMUST00000052250	protein coding
Zfp454-201	11	3.08	-36.64	3.89E-03	ENSMUST00000050595	protein coding
Zfp459-201	13	30.97	-28.64	1.14E-58	ENSMUST00000056470	protein coding
Zfp459-202	13	24.11	-130.46	2.41E-05	ENSMUST00000223644	protein coding
Zfp518b-201	5	55.43	-3.88	4.74E-24	ENSMUST00000057258	protein coding
Zfp532-205	18	10.9	-2.66	5.97E-05	ENSMUST00000182478	nonsense mediated decay
Zfp532-210	18	5.22	-6.04	9.35E-06	ENSMUST00000182979	protein coding
Zfp532-224	18	10.6	-147.94	1.02E-14	ENSMUST00000237714	protein coding
Zfp532-225	18	21.5	-5	4.94E-15	ENSMUST00000237756	nonsense mediated decay
Zfp534-201	4	85.33	-217.01	3.25E-78	ENSMUST00000117638	protein coding
Zfp551-201	7	1.06	-17.92	0.02	ENSMUST00000080348	protein coding
Zfp57-201	17	153.55	-4.33	1.96E-16	ENSMUST00000069250	protein coding
Zfp57-202	17	9.46	-3.05	5.11E-03	ENSMUST00000089968	protein coding
Zfp57-205	17	32.87	-17.4	7.20E-11	ENSMUST00000172540	protein coding
Zfp57-211	17	99.37	-14.07	4.65E-53	ENSMUST00000174524	protein coding
Zfp57-212	17	106.7	-4.42	1.19E-15	ENSMUST00000174672	protein coding
Zfp59-202	7	36.89	-93.27	8.02E-72	ENSMUST00000205701	protein coding
Zfp607a-201	7	1.35	-33.85	0.02	ENSMUST00000053722	protein coding
Zfp607b-202	7	5.67	-2.28	2.06E-03	ENSMUST00000120004	protein coding
Zfp62-206	11	10.17	-2.68	6.56E-06	ENSMUST00000136539	protein coding
Zfp623-201	15	20.89	-5.85	6.83E-06	ENSMUST00000037260	protein coding
Zfp74-201	7	4	-6.84	2.67E-04	ENSMUST00000032797	protein coding
Zfp786-201	6	2.41	-3.1	1.54E-03	ENSMUST00000058844	protein coding
Zfp850-202	7	22.48	-4.17	6.22E-04	ENSMUST00000180024	protein coding
Zfp945-201	17	8.74	-4.63	1.21E-04	ENSMUST00000088696	protein coding
Zfp978-202	4	172.9	-56.35	8.90E-94	ENSMUST00000154154	protein coding

Zfp979-201	4	79.88	-299.12	1.21E-55	ENSMUST00000037565	protein coding
Zfp980-201	4	106.47	-52.51	1.77E-147	ENSMUST00000105738	protein coding
Zfp980-202	4	161.29	-112.17	7.36E-81	ENSMUST00000180014	protein coding
Zfp981-201	4	49.88	-19.23	2.46E-75	ENSMUST00000105735	protein coding
Zfp981-202	4	25.2	-5.86	9.04E-09	ENSMUST00000140089	protein coding
Zfp984-201	4	52.52	-11.5	1.25E-62	ENSMUST00000105734	protein coding
Zfp984-202	4	44.58	-106.4	9.66E-42	ENSMUST00000122309	protein coding
Zfp985-201	4	79.26	-39.16	1.10E-94	ENSMUST00000081742	protein coding
Zfp985-202	4	18.75	-144.45	3.24E-05	ENSMUST00000139784	protein coding
Zfp985-203	4	12.55	-43.95	2.96E-04	ENSMUST00000143885	protein coding
Zfp986-201	4	96.35	-226.29	2.02E-49	ENSMUST00000123460	protein coding
Zfp987-201	4	61.16	-249.99	1.14E-51	ENSMUST00000091878	protein coding
Zfp987-202	4	5.75	-29	0.01	ENSMUST00000105730	protein coding
Zfp988-201	4	81.92	-101.25	8.15E-97	ENSMUST00000148762	protein coding
Zfp989-202	4	45.69	-668.52	2.59E-19	ENSMUST00000146688	protein coding
Zfp990-201	4	151.17	-24.25	1.02E-128	ENSMUST00000105741	protein coding
Zfp990-202	4	31.26	-13,603.24	0.02	ENSMUST00000105742	protein coding
Zfp990-204	4	3.71	-26.74	0.01	ENSMUST00000136309	protein coding
Zfp991-201	4	98.01	-32.74	3.95E-50	ENSMUST00000084149	protein coding
Zfp991-202	4	41.51	-11.65	3.94E-05	ENSMUST00000151022	protein coding
Zfp992-201	4	73.89	-6.93	6.86E-41	ENSMUST00000105733	protein coding
Zfp993-202	4	56.62	-6.65	2.11E-23	ENSMUST00000130825	protein coding
Zic3-203	X	59.39	-26,161.31	9.11E-03	ENSMUST00000088631	protein coding
Zmat4-201	8	3.53	-4.18	1.36E-04	ENSMUST00000042352	protein coding
Zscan18-201	7	7.35	-5.14	9.00E-04	ENSMUST00000046245	protein coding

Table S2-3. Expression values & metadata of insignificant transcript variants of downregulated genes						
**Name**	**Chromosome**	**Max group mean**	**Fold change**	**FDR p-value**	**ENSEMBL**	**Biotype**
Ankzf1-205	1	3.54	2.14	0.09	ENSMUST00000145459	nonsense mediated decay
Ankzf1-207	1	35.86	1.45	0.12	ENSMUST00000152233	protein coding
Arid3b-201	9	25.09	1.52	0.1	ENSMUST00000004780	protein coding
Arid3b-203	9	0.02	16.19	0.16	ENSMUST00000114165	nonsense mediated decay
Arid3b-204	9	0.49	-94.43	0.33	ENSMUST00000164010	protein coding
Arid3b-205	9	1.28	1.19	0.77	ENSMUST00000164035	nonsense mediated decay
Arid3b-208	9	0.16	2.29	0.62	ENSMUST00000170477	protein coding
Arid3b-209	9	15.03	1.86	2.84E-05	ENSMUST00000171444	protein coding
Arid3b-210	9	0.11	16.71	0.16	ENSMUST00000171949	protein coding
Arid5a-203	1	0.21	5.61	0.3	ENSMUST00000115031	protein coding
Arid5a-204	1	1.04	1.42	0.55	ENSMUST00000115032	protein coding
Arid5a-205	1	4.33	1.33	0.62	ENSMUST00000116629	nonsense mediated decay
Arid5a-207	1	0.65	-86.05	0.33	ENSMUST00000126413	protein coding
Arid5a-208	1	0.21	1.27	0.82	ENSMUST00000137906	protein coding
Arid5a-211	1	0.25	4.42	0.44	ENSMUST00000142319	protein coding
Ascl4-202	10	0.21	1.05	0.97	ENSMUST00000170396	protein coding
Atf1-201	15	72.45	1.54	7.21E-04	ENSMUST00000023769	protein coding
Atf1-202	15	0.27	5.06	0.33	ENSMUST00000163855	protein coding
Atf1-203	15	0.54	-2.63	0.62	ENSMUST00000165804	protein coding
Atf1-204	15	1.31	-62.36	0.39	ENSMUST00000166435	protein coding
Atf1-207	15	0.41	-45.77	0.44	ENSMUST00000169408	nonsense mediated decay
Atf1-208	15	0.91	2.41	0.51	ENSMUST00000171869	protein coding
Atf4-201	15	271.94	1.74	7.96E-14	ENSMUST00000109605	protein coding
Atf4-202	15	0.91	8.6	0.07	ENSMUST00000229828	protein coding

Atf5-201	7	9.84	1.39	0.21	ENSMUST00000047356	protein coding
Atf6-201	1	24.13	1.71	3.82E-06	ENSMUST00000027974	protein coding
Batf-201	12	0.25	-2.97	0.6	ENSMUST00000040536	protein coding
Baz2b-201	2	5.69	-2.45	0.06	ENSMUST00000090925	protein coding
Baz2b-202	2	4.48	1.01	0.98	ENSMUST00000112550	protein coding
Baz2b-204	2	4.27	-2.38	0.21	ENSMUST00000130637	protein coding
Baz2b-213	2	1.44	1.15	0.86	ENSMUST00000153136	protein coding
Bcl11a-201	11	0.36	-2.08	0.31	ENSMUST00000000881	protein coding
Bcl11a-202	11	0.33	-3.77	0.17	ENSMUST00000109514	protein coding
Bcl11a-203	11	0.01	-5.79	0.71	ENSMUST00000109516	protein coding
Bcl11a-204	11	0.25	-2.41	0.62	ENSMUST00000118955	protein coding
Bcl6-201	16	3.04	1.68	0.2	ENSMUST00000023151	protein coding
Bend2-202	X	0.06	-47.21	0.42	ENSMUST00000207320	protein coding
Bend2-204	X	0.06	-18.56	0.55	ENSMUST00000207800	protein coding
Bend2-205	X	0.04	-7.88	0.67	ENSMUST00000207853	protein coding
Bhlhe23-201	2	0.3	-9.43	0.17	ENSMUST00000108878	protein coding
Bnc2-201	4	2.97	-1,801.85	0.08	ENSMUST00000102820	protein coding
Bnc2-206	4	0.09	2.17	0.7	ENSMUST00000175800	protein coding
Bnc2-207	4	0.63	-61.83	0.39	ENSMUST00000175969	protein coding
Bnc2-210	4	0.03	-3.61	0.78	ENSMUST00000176370	protein coding
Bnc2-211	4	0.1	-18.53	0.55	ENSMUST00000176418	protein coding
Bnc2-215	4	0.64	-1.73	0.73	ENSMUST00000176691	protein coding
Bnc2-216	4	0.08	-9.98	0.63	ENSMUST00000176702	protein coding
Bnc2-218	4	0.53	-2.73	0.51	ENSMUST00000176971	protein coding
Bnc2-219	4	0.22	-10.46	0.63	ENSMUST00000176998	protein coding
Bnc2-222	4	0.94	-79.77	0.33	ENSMUST00000177277	protein coding

Cdc5l-201	17	247.35	1.38	9.66E-05	ENSMUST00000024727	protein coding
Cdx1-201	18	3.57	1.7	0.09	ENSMUST00000025521	protein coding
Cggbp1-201	16	54.25	1.62	2.20E-08	ENSMUST00000067744	protein coding
Cpeb1-201	7	0.22	3.86	0.44	ENSMUST00000098331	protein coding
Cpeb1-203	7	1.25	-1.62	0.67	ENSMUST00000130310	protein coding
Cpeb1-209	7	0.34	-1.35	0.8	ENSMUST00000178892	protein coding
Crebl2-201	6	4.46	1.91	0.02	ENSMUST00000046303	protein coding
Crebl2-202	6	0.57	1.42	0.79	ENSMUST00000111937	protein coding
Csrnp3-201	2	0.36	-240.6	0.24	ENSMUST00000053910	protein coding
Csrnp3-202	2	0.03	-10.46	0.63	ENSMUST00000112394	protein coding
Csrnp3-207	2	0.53	-2.84	0.54	ENSMUST00000145598	protein coding
Csrnp3-208	2	0.82	-358.78	0.19	ENSMUST00000176109	protein coding
Dach2-201	X	0.2	-6.34	0.24	ENSMUST00000067219	protein coding
Dach2-202	X	0.04	-1.27	0.9	ENSMUST00000113378	protein coding
Dach2-203	X	0.07	-26.92	0.5	ENSMUST00000113379	protein coding
Dach2-205	X	0.06	6.34	0.24	ENSMUST00000113382	protein coding
Dbx2-201	15	0.32	-4.74	0.07	ENSMUST00000054244	protein coding
Dmrt1-202	19	0.07	-12.77	0.61	ENSMUST00000087525	protein coding
Dmrtc2-201	7	1.01	-10.44	0.1	ENSMUST00000011493	protein coding
Dpf1-201	7	10	-1.24	0.33	ENSMUST00000049977	protein coding
Dpf1-207	7	1.41	-1.66	0.69	ENSMUST00000137848	protein coding
Dpf1-209	7	6.77	-1.97	0.23	ENSMUST00000142958	protein coding
Duxbl1-201	14	0.69	-10.08	0.12	ENSMUST00000049793	protein coding
Duxbl1-203	14	0.26	-7.12	0.25	ENSMUST00000172667	protein coding
Duxbl1-204	14	0.33	-28.92	0.5	ENSMUST00000173155	protein coding
Duxbl1-205	14	0.1	-15.08	0.59	ENSMUST00000173580	protein coding

E2f2-201	4	16.84	-1.18	0.36	ENSMUST00000061721	protein coding
Ebf1-201	11	0.13	-114.58	0.3	ENSMUST00000081265	protein coding
Ebf1-202	11	0.21	-105.38	0.32	ENSMUST00000101326	protein coding
Ebf1-203	11	0.32	-2.39	0.47	ENSMUST00000109268	protein coding
Egr4-201	6	0.72	-3.43	0.33	ENSMUST00000095759	protein coding
Epas1-201	17	31.1	-1.43	0.1	ENSMUST00000024954	protein coding
Esr2-202	12	0.12	-58.95	0.41	ENSMUST00000101291	protein coding
Esr2-203	12	0.25	-1.26	0.86	ENSMUST00000110421	protein coding
Etv1-203	12	5.93	-1,780.66	0.08	ENSMUST00000160244	protein coding
Etv1-204	12	0.1	-24.32	0.52	ENSMUST00000160701	protein coding
Etv1-205	12	5.52	-1,217.03	0.1	ENSMUST00000160856	protein coding
Etv1-206	12	2.88	-363.75	0.19	ENSMUST00000160996	protein coding
Etv1-208	12	0.95	-2.77	0.55	ENSMUST00000161513	protein coding
Etv1-210	12	0.1	1.39	0.86	ENSMUST00000161980	protein coding
Etv1-211	12	0.21	-5.05	0.46	ENSMUST00000162563	protein coding
Etv5-201	16	120.6	1.47	2.54E-07	ENSMUST00000079601	protein coding
Etv5-204	16	0.67	-1.07	0.97	ENSMUST00000170393	protein coding
Etv5-205	16	0.16	23.33	0.12	ENSMUST00000170803	nonsense mediated decay
Etv6-201	6	7.66	-2.59	0.24	ENSMUST00000081028	protein coding
Etv6-202	6	5.64	-1	0.99	ENSMUST00000111963	protein coding
Etv6-204	6	0.09	-10.14	0.63	ENSMUST00000164648	protein coding
Fiz1-201	7	56.58	1.52	9.65E-04	ENSMUST00000077385	protein coding
Fiz1-202	7	2.01	2.63	0.28	ENSMUST00000165320	protein coding
Fiz1-203	7	4.26	1.11	0.78	ENSMUST00000167804	protein coding
Fiz1-204	7	0.38	3.88	0.28	ENSMUST00000207030	protein coding
Fiz1-205	7	0.19	6.49	0.27	ENSMUST00000207412	protein coding

Fiz1-209	7	4.42	-1.51	0.68	ENSMUST00000209060	protein coding
Foxi3-201	6	0.83	1.34	0.79	ENSMUST00000069634	protein coding
Foxn2-201	17	22.28	1.82	3.06E-05	ENSMUST00000112238	protein coding
Foxn2-203	17	0.96	-267.71	0.23	ENSMUST00000141052	nonsense mediated decay
Foxn2-204	17	0.32	1.61	0.78	ENSMUST00000155640	protein coding
Foxr2-201	X	2.39	-1,140.69	0.1	ENSMUST00000096265	protein coding
Foxr2-202	X	7.57E-03	8.44	0.31	ENSMUST00000163801	protein coding
Gabpa-201	16	37.39	1.84	1.42E-08	ENSMUST00000009120	protein coding
Gabpa-202	16	64.39	-1.3	0.29	ENSMUST00000114184	protein coding
Gata6-201	18	3.31	1.6	0.09	ENSMUST00000047762	protein coding
Gata6-203	18	0.11	16.19	0.16	ENSMUST00000234512	protein coding
Gata6-206	18	0.07	-10.14	0.63	ENSMUST00000235081	protein coding
Gbx2-201	1	35.85	-1.52	2.63E-04	ENSMUST00000036954	protein coding
Gli1-201	10	18.98	-1.89	5.89E-09	ENSMUST00000026474	protein coding
Gli1-202	10	0.3	6.68	0.25	ENSMUST00000218236	protein coding
Gli2-204	1	0.12	2.4	0.62	ENSMUST00000160991	nonsense mediated decay
Gli2-205	1	0.36	-60.8	0.4	ENSMUST00000161056	nonsense mediated decay
Gli2-206	1	1.78	-8.57	0.08	ENSMUST00000161301	protein coding
Gm10037-201	13	1.48	-123.21	0.3	ENSMUST00000221266	protein coding
Gm10037-203	13	2.32	-238.01	0.23	ENSMUST00000222626	protein coding
Gm10037-204	13	0.23	-18.53	0.55	ENSMUST00000231606	protein coding
Gm26920-201	7	4.78	-1.23	0.75	ENSMUST00000182746	nonsense mediated decay
Gm26965-201	13	0.57	-1.97	0.63	ENSMUST00000167914	nonsense mediated decay
Gm28557-203	13	3.57	-1.47	0.46	ENSMUST00000186303	nonsense mediated decay
Gm42715-201	7	2.74	1.16	0.58	ENSMUST00000189629	nonsense mediated decay
Gm42715-202	7	0.76	2.67	0.51	ENSMUST00000206825	nonsense mediated decay

Gtf2i-201	5	1.12	2.41	0.2	ENSMUST00000059042	protein coding
Gtf2i-206	5	0.3	-9.65	0.13	ENSMUST00000172715	protein coding
Gtf2i-210	5	19.4	1.15	0.45	ENSMUST00000173341	protein coding
Gtf2i-211	5	4.47	-1.3	0.78	ENSMUST00000173485	protein coding
Gtf2i-212	5	0.13	3.02	0.55	ENSMUST00000173504	protein coding
Gtf2i-214	5	0.24	-2.34	0.68	ENSMUST00000173651	nonsense mediated decay
Gtf2i-218	5	2.09	-7.21	0.06	ENSMUST00000174133	protein coding
Gtf2i-219	5	2.01	2.92	0.18	ENSMUST00000174155	protein coding
Gtf2i-221	5	0.64	-2.07	0.58	ENSMUST00000174188	protein coding
Gtf2i-223	5	0.02	-12.26	0.62	ENSMUST00000174513	protein coding
Gtf2i-224	5	1.19	-1.72	0.69	ENSMUST00000174772	protein coding
Hesx1-202	14	1.23	-179.27	0.25	ENSMUST00000224331	protein coding
Hey2-201	10	0.87	-1.84	0.54	ENSMUST00000019924	protein coding
Hlf-201	11	0.51	-1.29	0.78	ENSMUST00000004051	protein coding
Hlf-206	11	0.09	16.19	0.15	ENSMUST00000176001	protein coding
Hnf1a-201	5	0.67	-1.27	0.81	ENSMUST00000031535	protein coding
Hnf1a-202	5	0.17	-33.42	0.47	ENSMUST00000176550	nonsense mediated decay
Hnf1a-203	5	0.3	-1.46	0.82	ENSMUST00000176911	nonsense mediated decay
Hnf1b-201	11	0.35	2.47	0.59	ENSMUST00000021016	protein coding
Hnf1b-202	11	0.59	1.91	0.63	ENSMUST00000108113	protein coding
Hnf1b-203	11	0.86	-1.29	0.8	ENSMUST00000108114	protein coding
Hnf1b-204	11	0.02	-3.61	0.78	ENSMUST00000135975	protein coding
Hnf1b-205	11	0.05	8.44	0.31	ENSMUST00000146786	protein coding
Hnf4a-201	2	0.13	-86.38	0.33	ENSMUST00000018094	protein coding
Hnf4a-202	2	0.14	-92.82	0.33	ENSMUST00000109411	protein coding
Hnf4a-204	2	0.13	1.6	0.79	ENSMUST00000137449	protein coding

Hnf4a-205	2	0.03	-3.61	0.78	ENSMUST00000143911	protein coding
Hoxb1-201	11	0.29	-90.65	0.33	ENSMUST00000019117	protein coding
Hoxb9-201	11	0.35	1.56	0.74	ENSMUST00000000010	protein coding
Hoxb9-202	11	2.45	-5.61	0.06	ENSMUST00000174042	protein coding
Hoxc4-201	15	0.53	-12.86	0.08	ENSMUST00000100164	protein coding
Hoxc4-202	15	0.03	5.94	0.29	ENSMUST00000165375	protein coding
Hsf5-201	11	0.4	-1.58	0.58	ENSMUST00000093956	protein coding
Kdm5b-201	1	61.09	1.03	0.78	ENSMUST00000047714	protein coding
Kdm5b-203	1	196.24	-1.33	7.21E-04	ENSMUST00000112198	protein coding
Klf10-203	15	0.06	1.08	0.97	ENSMUST00000227920	protein coding
Klf10-204	15	0.09	-5.79	0.71	ENSMUST00000228416	protein coding
Klf10-206	15	0.58	4.47	0.26	ENSMUST00000228772	protein coding
Klf12-201	14	2.6	-5.32	0.1	ENSMUST00000097079	protein coding
Klf12-204	14	2.86	-308.22	0.2	ENSMUST00000226774	protein coding
Klf12-205	14	4.33	1.29	0.58	ENSMUST00000228216	protein coding
Klf13-201	7	15.83	1.55	0.04	ENSMUST00000063694	protein coding
Klf13-202	7	0.03	-3.61	0.78	ENSMUST00000183817	protein coding
Klf13-204	7	0.17	1.58	0.81	ENSMUST00000185175	protein coding
Klf15-202	6	0.17	-62.63	0.4	ENSMUST00000113530	protein coding
Klf15-203	6	0.74	-6.09	0.25	ENSMUST00000203039	protein coding
Klf15-204	6	11.94	-1.21	0.59	ENSMUST00000203607	protein coding
Klf15-205	6	0.85	-1.42	0.82	ENSMUST00000205136	protein coding
Klf17-201	4	0.51	-2.72	0.23	ENSMUST00000062747	protein coding
Klf3-205	5	20.71	-2.45	0.09	ENSMUST00000198946	protein coding
Klf3-206	5	6.04	-5.99	0.08	ENSMUST00000200384	nonsense mediated decay
Klf4-203	4	1.01	-3.83	0.4	ENSMUST00000132746	protein coding

Klf5-201	14	162.18	1.1	0.3	ENSMUST00000005279	protein coding
Klf5-202	14	1.24	2.11	0.05	ENSMUST00000226784	protein coding
Klf8-203	X	0.88	-34.07	0.46	ENSMUST00000143880	protein coding
Lef1-202	3	4.88	-2,473.29	0.06	ENSMUST00000066849	protein coding
Lef1-203	3	2.52	-860.71	0.12	ENSMUST00000098611	protein coding
Mef2b-201	8	2.9	-4.33	0.06	ENSMUST00000110140	protein coding
Mef2b-202	8	0.46	1.82	0.74	ENSMUST00000110141	protein coding
Mef2b-203	8	7.08	-4.35	0.19	ENSMUST00000110143	protein coding
Mef2b-204	8	10.5	-1.34	0.6	ENSMUST00000110146	protein coding
Mesp2-201	7	2.12	1.22	0.77	ENSMUST00000107394	protein coding
Mitf-201	6	0.45	-2.55	0.54	ENSMUST00000043628	protein coding
Mitf-202	6	5.12	-1.44	0.09	ENSMUST00000043637	protein coding
Mitf-205	6	8.43E-03	-3.61	0.78	ENSMUST00000139462	nonsense mediated decay
Mitf-208	6	3.25	-516.54	0.15	ENSMUST00000203938	protein coding
Msantd3-202	4	27.93	-2.15	0.12	ENSMUST00000064807	protein coding
Mtf2-204	5	5.14	-1.17	0.86	ENSMUST00000129921	nonsense mediated decay
Mtf2-208	5	18.58	-1,974.23	0.07	ENSMUST00000137996	protein coding
Mtf2-213	5	0.06	1.52	0.82	ENSMUST00000170319	protein coding
Mtf2-214	5	1.63	3.23	0.15	ENSMUST00000172045	protein coding
Myrf-201	19	19.84	1.11	0.67	ENSMUST00000088013	protein coding
Myrf-202	19	0.06	-33.54	0.47	ENSMUST00000186056	protein coding
Myrf-203	19	2.79	-1.12	0.9	ENSMUST00000186854	protein coding
Myrf-205	19	1.84	1.93	0.21	ENSMUST00000189897	protein coding
Nanog-203	6	1.45	-1.67	0.5	ENSMUST00000112581	protein coding
Neurog3-201	10	0.54	-125.47	0.3	ENSMUST00000050103	protein coding
Nfatc4-201	14	3	-1.26	0.64	ENSMUST00000024179	protein coding

Nfatc4-202	14	3.81	-1.15	0.69	ENSMUST00000172271	protein coding
Nfatc4-209	14	0.07	3.36	0.51	ENSMUST00000226979	protein coding
Nfe2-201	15	0.28	-1.82	0.68	ENSMUST00000075192	protein coding
Nfe2-202	15	0.09	-10.46	0.63	ENSMUST00000131364	protein coding
Nfe2-204	15	0.39	-37.74	0.46	ENSMUST00000132836	protein coding
Nfe2-205	15	0.36	-2.59	0.52	ENSMUST00000133600	protein coding
Nfe2-206	15	0.06	3.96	0.44	ENSMUST00000134193	protein coding
Nfe2-209	15	0.04	-5.79	0.71	ENSMUST00000154510	protein coding
Nfe2-210	15	0.04	-12.32	0.62	ENSMUST00000156927	protein coding
Nfib-202	4	0.7	1.06	0.95	ENSMUST00000064770	protein coding
Nfib-203	4	0.76	-1.57	0.48	ENSMUST00000107245	protein coding
Nfib-204	4	0.47	-216.87	0.23	ENSMUST00000107246	protein coding
Nfib-205	4	0.64	-312.54	0.2	ENSMUST00000107247	protein coding
Nfib-212	4	0.22	-24.32	0.52	ENSMUST00000155821	protein coding
Nfkb1-203	3	7.22	-2.18	0.14	ENSMUST00000132668	protein coding
Nfkb1-206	3	19.35	-1.08	0.55	ENSMUST00000164430	protein coding
Nfkb1-207	3	5.87	1.06	0.91	ENSMUST00000184550	nonsense mediated decay
Nfkb1-209	3	1.02	-471.81	0.17	ENSMUST00000196469	nonsense mediated decay
Nhlh1-201	1	0.17	-1.87	0.63	ENSMUST00000059794	protein coding
Nkx1-2-201	7	0.35	-173.13	0.25	ENSMUST00000054562	protein coding
Nkx2-4-201	2	0.44	1.57	0.49	ENSMUST00000067020	protein coding
Npas3-201	12	0.17	-3.33	0.22	ENSMUST00000101432	protein coding
Npas3-203	12	0.02	16.71	0.16	ENSMUST00000223057	protein coding
Nr0b1-201	X	45.5	1.58	7.80E-04	ENSMUST00000026036	protein coding
Nr1d2-201	14	11.23	1.02	0.87	ENSMUST00000090543	protein coding
Nr1d2-204	14	0.09	-1.02	0.99	ENSMUST00000225491	protein coding

Nr2c1-201	10	4.84	-1.17	0.78	ENSMUST00000092213	protein coding
Nr2c1-202	10	0.08	3.57	0.52	ENSMUST00000099343	protein coding
Nr2c1-203	10	3.34	1.57	0.28	ENSMUST00000105290	protein coding
Nr5a2-206	1	0.1	-12.08	0.62	ENSMUST00000195428	protein coding
Otx2-203	14	0.22	-18.84	0.55	ENSMUST00000119739	protein coding
Otx2-204	14	10.66	-2,749.52	0.06	ENSMUST00000122009	protein coding
Otx2-208	14	0.46	-73.32	0.36	ENSMUST00000152018	protein coding
Otx2-209	14	0.38	-146.02	0.29	ENSMUST00000226501	protein coding
Ovol1-201	19	0.79	1.5	0.67	ENSMUST00000025861	protein coding
Ovol1-202	19	0.09	16.71	0.16	ENSMUST00000238060	protein coding
Pax5-201	4	0.06	-2.07	0.56	ENSMUST00000014174	protein coding
Pax5-212	4	0.02	-3.61	0.78	ENSMUST00000173733	protein coding
Pbx1-202	1	0.19	2.26	0.63	ENSMUST00000072863	protein coding
Pbx1-205	1	5.45	-1.76	0.22	ENSMUST00000176540	protein coding
Pbx1-206	1	2.44	-2.79	0.36	ENSMUST00000176790	protein coding
Pbx1-208	1	0.69	-1.51	0.47	ENSMUST00000188912	protein coding
Phf21a-201	2	4.29	1.21	0.67	ENSMUST00000090586	protein coding
Phf21a-202	2	8.29	-1.77	0.16	ENSMUST00000111291	protein coding
Phf21a-203	2	0.1	-37.34	0.46	ENSMUST00000111292	protein coding
Phf21a-206	2	0.17	-18.91	0.55	ENSMUST00000128781	protein coding
Phf21a-207	2	2.26	-3.43	0.35	ENSMUST00000138995	protein coding
Phf21a-209	2	1.63	3.03	0.12	ENSMUST00000159366	protein coding
Phf21a-210	2	6.27	-517.21	0.15	ENSMUST00000159727	protein coding
Phf21a-211	2	8.86	1.05	0.91	ENSMUST00000159961	protein coding
Phf21a-215	2	2.23	-2.3	0.28	ENSMUST00000162146	protein coding
Phf21a-217	2	1.09	-1.23	0.89	ENSMUST00000162497	protein coding

Plag1-201	4	4.46	-1.09	0.72	ENSMUST00000003369	protein coding
Plag1-202	4	1.67	-1.43	0.79	ENSMUST00000137439	nonsense mediated decay
Plag1-204	4	0.15	1.99	0.73	ENSMUST00000151543	nonsense mediated decay
Plscr1-202	9	25.41	1.23	0.38	ENSMUST00000186364	protein coding
Pou4f3-201	18	0.15	1.82	0.64	ENSMUST00000025374	protein coding
Pou4f3-202	18	0.21	-1.75	0.7	ENSMUST00000237247	protein coding
Pou5f1-202	17	0.05	8.44	0.31	ENSMUST00000172651	protein coding
Pou5f1-203	17	4.63	-319.13	0.19	ENSMUST00000173256	protein coding
Pou5f1-204	17	0.38	1.14	0.94	ENSMUST00000173805	protein coding
Pou5f1-206	17	1.91	-194.63	0.24	ENSMUST00000174782	protein coding
Prdm1-202	10	0.76	-1.65	0.55	ENSMUST00000105490	protein coding
Prdm1-204	10	0.84	-6.06	0.09	ENSMUST00000218369	protein coding
Prdm16-201	4	0.4	-1.35	0.78	ENSMUST00000030902	protein coding
Prdm16-202	4	0.32	5.84	0.15	ENSMUST00000070313	protein coding
Prdm16-204	4	0.23	2.32	0.6	ENSMUST00000105636	protein coding
Prdm16-206	4	0.18	-234.95	0.24	ENSMUST00000105638	protein coding
Prdm16-207	4	0.12	30.35	0.1	ENSMUST00000124771	protein coding
Prdm4-201	10	31.32	1.71	1.38E-03	ENSMUST00000037646	protein coding
Prdm4-206	10	5.39	-1.09	0.82	ENSMUST00000219370	nonsense mediated decay
Prdm5-202	6	9.43	-1.29	0.54	ENSMUST00000031976	protein coding
Prdm5-203	6	0.47	-2.13	0.62	ENSMUST00000081219	protein coding
Prdm5-204	6	1	-5.9	0.2	ENSMUST00000172638	protein coding
Pura-201	18	19.28	1.03	0.9	ENSMUST00000051301	protein coding
Rarg-204	15	1.33	-3.7	0.4	ENSMUST00000130204	protein coding
Rbak-201	5	9.21	1.32	0.29	ENSMUST00000049861	protein coding
Rbpj-207	5	4.31	1.76	0.18	ENSMUST00000201883	protein coding

Rbpj-208	5	2.3	-1.65	0.36	ENSMUST00000201912	protein coding
Rbpj-210	5	1.6	-162.84	0.26	ENSMUST00000201991	protein coding
Rbsn-201	6	16.9	1.37	0.02	ENSMUST00000014694	protein coding
Rbsn-202	6	0.11	1.73	0.78	ENSMUST00000124635	protein coding
Rbsn-205	6	0.04	-3.61	0.78	ENSMUST00000203516	protein coding
Rest-202	5	50.66	-1.44	0.01	ENSMUST00000113449	protein coding
Rfx2-201	17	6.04	-2.28	0.11	ENSMUST00000002444	protein coding
Rfx2-202	17	18.01	1.39	0.06	ENSMUST00000086801	protein coding
Rfx2-203	17	0.72	1.86	0.62	ENSMUST00000233000	protein coding
Rfx4-202	10	0.24	-126.37	0.3	ENSMUST00000060397	protein coding
Rfx4-203	10	0.34	-1.42	0.64	ENSMUST00000095388	protein coding
Rorb-201	19	1.97	-2,560.79	0.06	ENSMUST00000040153	protein coding
Rorb-203	19	3.63E-03	8.44	0.31	ENSMUST00000112832	protein coding
Rsl1-201	13	0.44	1.52	0.62	ENSMUST00000021997	protein coding
Rsl1-202	13	1.44	-1.54	0.74	ENSMUST00000225334	nonsense mediated decay
Rsl1-203	13	5.64	1.65	0.14	ENSMUST00000249257	protein coding
Rxrg-201	1	0.96	-11.68	0.06	ENSMUST00000015987	protein coding
Rxrg-202	1	0.12	-29.16	0.5	ENSMUST00000111380	protein coding
Rxrg-203	1	0.31	-93.45	0.34	ENSMUST00000111384	protein coding
Rxrg-204	1	0.45	-5.12	0.33	ENSMUST00000111386	protein coding
Sall1-201	8	72.73	-1.32	3.58E-03	ENSMUST00000034090	protein coding
Scml4-201	10	0.04	5.54	0.35	ENSMUST00000063063	protein coding
Scml4-202	10	0.16	-2.2	0.54	ENSMUST00000105494	protein coding
Scml4-203	10	0.02	8.44	0.31	ENSMUST00000105495	protein coding
Scml4-204	10	0.11	26.67	0.1	ENSMUST00000125576	protein coding
Setdb2-202	14	0.11	1.53	0.8	ENSMUST00000111253	protein coding

Setdb2-205	14	12.49	1.58	1.74E-06	ENSMUST00000161459	protein coding
Six1-201	12	1.25	-1.17	0.85	ENSMUST00000050029	protein coding
Skor1-202	9	0.1	-1.18	0.91	ENSMUST00000116613	protein coding
Snai2-201	16	0.49	-1.87	0.39	ENSMUST00000023356	protein coding
Snapc5-201	9	36.88	1.47	2.84E-05	ENSMUST00000034965	protein coding
Sox17-201	1	0.29	-138.52	0.29	ENSMUST00000027035	protein coding
Sox17-202	1	0.05	23.93	0.1	ENSMUST00000116652	protein coding
Sox17-204	1	0.91	-2.59	0.51	ENSMUST00000191939	protein coding
Sox17-206	1	0.02	-10.03	0.63	ENSMUST00000192650	protein coding
Sp110-201	1	0.17	-1.43	0.82	ENSMUST00000093508	protein coding
Sp8-201	12	0.09	1.13	0.91	ENSMUST00000063918	protein coding
Sp8-202	12	0.05	2.1	0.69	ENSMUST00000223305	protein coding
Spdef-201	17	0.21	1.96	0.62	ENSMUST00000025054	protein coding
Spdef-202	17	0.06	-14.28	0.59	ENSMUST00000114870	protein coding
Spi1-201	2	0.08	-25.6	0.51	ENSMUST00000002180	protein coding
Spi1-205	2	0.05	5.94	0.28	ENSMUST00000249728	protein coding
Spi1-206	2	0.14	-44.01	0.44	ENSMUST00000249729	protein coding
Spib-201	7	0.55	-4.03	0.22	ENSMUST00000035323	protein coding
Spib-202	7	0.04	-5.79	0.71	ENSMUST00000098483	protein coding
Spib-203	7	0.09	2.73	0.59	ENSMUST00000205506	protein coding
Spic-201	10	2.81	-733.88	0.13	ENSMUST00000004473	protein coding
Spic-202	10	1.36	-273.76	0.21	ENSMUST00000133724	nonsense mediated decay
Spic-203	10	2.38	-229.76	0.23	ENSMUST00000138734	protein coding
Stat1-201	1	0.04	2.17	0.68	ENSMUST00000070968	protein coding
Stat1-206	1	8.35	1.01	0.98	ENSMUST00000186857	protein coding
Stat1-207	1	0.45	-1.55	0.82	ENSMUST00000188681	protein coding

Stat1-210	1	0.44	-60.52	0.4	ENSMUST00000189336	nonsense mediated decay
Stat1-211	1	1.38	-117.52	0.3	ENSMUST00000189347	protein coding
Tal1-203	4	0.06	-33.41	0.47	ENSMUST00000161601	protein coding
Tal1-204	4	0.56	-3.03	0.3	ENSMUST00000162489	protein coding
Tbx3-202	5	1.58	-1.2	0.81	ENSMUST00000079719	protein coding
Tbx3-203	5	15.39	-1.25	0.13	ENSMUST00000121021	protein coding
Tcf15-201	2	63	1.13	0.45	ENSMUST00000089112	protein coding
Tcf4-201	18	0.25	7.34	0.1	ENSMUST00000066717	protein coding
Tcf4-202	18	10.53	1.14	0.51	ENSMUST00000078486	protein coding
Tcf4-204	18	0.14	4.38	0.41	ENSMUST00000114978	protein coding
Tcf4-207	18	0.51	-1.03	0.97	ENSMUST00000114985	protein coding
Tcf4-210	18	0.02	8.44	0.31	ENSMUST00000128706	protein coding
Tcf4-218	18	0.07	26.67	0.1	ENSMUST00000200862	protein coding
Tcf4-231	18	0.2	39.76	0.06	ENSMUST00000201235	protein coding
Tcf4-232	18	0.18	-22.78	0.53	ENSMUST00000201288	protein coding
Tcf4-234	18	0.92	1.36	0.82	ENSMUST00000201410	protein coding
Tcf4-235	18	0.05	16.19	0.14	ENSMUST00000201537	protein coding
Tcf4-237	18	0.02	-3.61	0.78	ENSMUST00000201627	protein coding
Tcf4-243	18	0.51	-151.16	0.29	ENSMUST00000202057	protein coding
Tcf4-248	18	0.95	-2.8	0.46	ENSMUST00000202354	protein coding
Tcf4-249	18	2.17	-797.25	0.14	ENSMUST00000202435	protein coding
Tcf4-251	18	0.5	-138.7	0.3	ENSMUST00000202474	protein coding
Tcf4-257	18	0.28	-90.04	0.33	ENSMUST00000202772	protein coding
Tcfl5-201	2	26.66	1.61	2.69E-03	ENSMUST00000037877	protein coding
Tcfl5-203	2	1.04	30.85	0.1	ENSMUST00000161425	nonsense mediated decay
Terb1-201	8	0.13	-2.36	0.66	ENSMUST00000064576	protein coding

Terb1-202	8	0.02	-7.91	0.67	ENSMUST00000159713	nonsense mediated decay
Terb1-203	8	0.22	1.65	0.67	ENSMUST00000161520	nonsense mediated decay
Terf1-203	1	43.89	1.63	0.02	ENSMUST00000188371	protein coding
Tet1-203	10	0.49	2.61	0.35	ENSMUST00000173087	protein coding
Tet1-204	10	0.07	16.19	0.16	ENSMUST00000173905	nonsense mediated decay
Tet1-205	10	8.12	-1.92	0.07	ENSMUST00000174121	protein coding
Tet1-206	10	34.88	-1.33	6.20E-03	ENSMUST00000174189	protein coding
Tet1-207	10	2.68	3.23	0.23	ENSMUST00000218438	protein coding
Tet1-208	10	0.79	-55.83	0.4	ENSMUST00000218782	protein coding
Tet1-209	10	18.05	-1,480.60	0.08	ENSMUST00000227494	protein coding
Tet1-210	10	1.43	-1.71	0.69	ENSMUST00000228901	protein coding
Tet2-201	3	3.52	1.15	0.72	ENSMUST00000098603	protein coding
Tet2-207	3	0.5	-35.54	0.47	ENSMUST00000198974	protein coding
Tfec-201	6	1.54	-405.74	0.18	ENSMUST00000031533	protein coding
Tgif1-201	17	135.37	-1.17	0.3	ENSMUST00000059775	protein coding
Tgif1-204	17	23.02	-1.84	0.12	ENSMUST00000127719	protein coding
Tgif1-206	17	5.17	1.31	0.76	ENSMUST00000134654	protein coding
Tgif1-207	17	4.08	1.97	0.04	ENSMUST00000135007	protein coding
Tgif1-208	17	1.58	-3.71	0.36	ENSMUST00000156484	protein coding
Tgif1-209	17	6.89	-1.86	0.17	ENSMUST00000166395	protein coding
Tgif1-210	17	1.2	3.46	0.43	ENSMUST00000172229	protein coding
Thyn1-201	9	26.79	-1.97	0.13	ENSMUST00000039161	protein coding
Thyn1-202	9	14.92	1.24	0.45	ENSMUST00000213683	protein coding
Thyn1-203	9	168.27	1.25	0.33	ENSMUST00000213770	protein coding
Topors-201	4	71.82	1.62	1.67E-08	ENSMUST00000042575	protein coding
Trerf1-204	17	7.69	1.42	3.80E-03	ENSMUST00000190080	protein coding

Trp53-202	11	121.65	-1.12	0.48	ENSMUST00000108657	protein coding
Trps1-202	15	0.14	-73.24	0.35	ENSMUST00000183421	protein coding
Trps1-203	15	0.39	-3.88	0.32	ENSMUST00000183757	protein coding
Trps1-206	15	0.59	3.1	0.48	ENSMUST00000184885	protein coding
Trps1-207	15	0.99	-133.67	0.3	ENSMUST00000185183	protein coding
Tsc22d1-201	14	222.89	1.49	1.23E-05	ENSMUST00000022587	protein coding
Tsc22d1-203	14	0.58	-91.59	0.33	ENSMUST00000101618	protein coding
Tsc22d1-205	14	0.85	2.48	0.09	ENSMUST00000133224	protein coding
Tsc22d1-206	14	0.89	3.21	0.08	ENSMUST00000134109	protein coding
Tsc22d1-207	14	0.44	-45.25	0.43	ENSMUST00000140251	protein coding
Tsc22d1-208	14	0.57	-67.72	0.37	ENSMUST00000142683	protein coding
Tsc22d1-211	14	1.21	4.78	0.14	ENSMUST00000175984	nonsense mediated decay
Tsc22d1-212	14	0.81	1.54	0.78	ENSMUST00000176581	protein coding
Tsc22d1-216	14	0.19	-24.32	0.52	ENSMUST00000177471	nonsense mediated decay
Usf1-201	1	28.79	1.11	0.81	ENSMUST00000001284	nonsense mediated decay
Usf1-207	1	9.02	1.16	0.63	ENSMUST00000160486	protein coding
Usf1-210	1	42.1	1.36	0.09	ENSMUST00000167546	protein coding
Vsx2-202	12	0.17	-1.24	0.85	ENSMUST00000169934	protein coding
Xbp1-201	11	87.86	1.8	2.90E-08	ENSMUST00000063084	protein coding
Xbp1-203	11	5.97	-1.3	0.67	ENSMUST00000149159	protein coding
Xbp1-204	11	1.41	-2.42	0.39	ENSMUST00000149623	protein coding
Xbp1-205	11	28.9	1.23	0.44	ENSMUST00000239150	protein coding
Zbed4-201	15	37.2	1.2	0.21	ENSMUST00000041297	protein coding
Zbed5-201	5	14.14	1.47	7.21E-03	ENSMUST00000041466	protein coding
Zbtb12-201	17	21.37	-1.2	0.59	ENSMUST00000052778	protein coding
Zbtb12-202	17	0.54	-31.9	0.47	ENSMUST00000173093	protein coding

Zbtb3-201	19	6.59	1.58	0.24	ENSMUST00000172175	protein coding
Zbtb45-202	7	0.04	-5.79	0.71	ENSMUST00000209997	protein coding
Zbtb45-203	7	4.84	-1.43	0.69	ENSMUST00000210108	protein coding
Zbtb45-204	7	3.29	-1.78	0.28	ENSMUST00000210282	protein coding
Zc3h8-201	2	22.23	1.56	5.06E-06	ENSMUST00000028866	protein coding
Zeb1-202	18	0.58	-7.77	0.68	ENSMUST00000159390	protein coding
Zeb1-205	18	0.09	-12.08	0.62	ENSMUST00000160910	protein coding
Zeb1-206	18	0.29	-52.57	0.42	ENSMUST00000161295	nonsense mediated decay
Zeb2-201	2	0.08	4.34	0.31	ENSMUST00000028229	protein coding
Zeb2-202	2	0.04	-35.41	0.46	ENSMUST00000068415	protein coding
Zeb2-203	2	0.06	65.58	0.06	ENSMUST00000076836	protein coding
Zeb2-211	2	0.02	1.52	0.82	ENSMUST00000153561	protein coding
Zeb2-212	2	0.02	2.22	0.66	ENSMUST00000176438	protein coding
Zeb2-213	2	0.03	2.92	0.55	ENSMUST00000176732	protein coding
Zeb2-214	2	0.02	-15.08	0.59	ENSMUST00000177302	protein coding
Zeb2-215	2	0.15	-88.43	0.33	ENSMUST00000200844	protein coding
Zeb2-221	2	0.01	3.36	0.5	ENSMUST00000201804	protein coding
Zeb2-222	2	0.02	-3.61	0.78	ENSMUST00000201969	protein coding
Zeb2-227	2	0.06	-3.61	0.78	ENSMUST00000202432	protein coding
Zfhx2-201	14	8.4	1	0.99	ENSMUST00000036328	protein coding
Zfhx4-201	3	5.01E-03	-12.08	0.62	ENSMUST00000026284	protein coding
Zfhx4-203	3	0.06	-99.36	0.32	ENSMUST00000175866	protein coding
Zfhx4-205	3	0.02	-48.31	0.43	ENSMUST00000176383	protein coding
Zfp108-202	7	0.07	-28.92	0.5	ENSMUST00000205982	protein coding
Zfp108-203	7	0.42	-3.62	0.26	ENSMUST00000206777	protein coding
Zfp109-201	7	5.01	1.2	0.74	ENSMUST00000037448	protein coding

Zfp11-201	5	3.13	1.56	0.3	ENSMUST00000049778	protein coding
Zfp114-201	7	0.56	1.65	0.4	ENSMUST00000086010	protein coding
Zfp114-202	7	0.71	-1.77	0.74	ENSMUST00000205309	protein coding
Zfp114-203	7	2.41	-1.39	0.64	ENSMUST00000206547	protein coding
Zfp13-201	17	0.56	-1.93	0.48	ENSMUST00000057029	protein coding
Zfp13-202	17	13.21	-1.52	0.1	ENSMUST00000115516	protein coding
Zfp13-203	17	5.57	-571.05	0.14	ENSMUST00000227952	protein coding
Zfp14-201	7	1.73	1.03	0.98	ENSMUST00000077787	protein coding
Zfp14-202	7	0.48	1.37	0.78	ENSMUST00000207072	protein coding
Zfp14-203	7	0.57	2.77	0.25	ENSMUST00000207873	protein coding
Zfp169-201	13	0.27	1.23	0.9	ENSMUST00000110110	protein coding
Zfp169-204	13	1.27	-80.69	0.34	ENSMUST00000176949	protein coding
Zfp235-201	7	5.41	1.32	0.21	ENSMUST00000056549	protein coding
Zfp235-202	7	0.76	3.69	0.09	ENSMUST00000205680	protein coding
Zfp239-201	6	0.95	-1.02	0.99	ENSMUST00000079405	protein coding
Zfp266-201	9	18.5	-1.28	0.31	ENSMUST00000068296	protein coding
Zfp266-202	9	26.74	1.24	0.13	ENSMUST00000174462	protein coding
Zfp266-204	9	1.07	1.02	0.99	ENSMUST00000213418	protein coding
Zfp266-205	9	0.64	2.96	0.1	ENSMUST00000215908	protein coding
Zfp273-201	13	0.71	1.08	0.95	ENSMUST00000012725	protein coding
Zfp273-203	13	2.05	-1.83	0.4	ENSMUST00000181391	protein coding
Zfp281-201	1	52.63	1.4	0.06	ENSMUST00000047734	protein coding
Zfp281-202	1	29.75	-1.29	0.52	ENSMUST00000112046	protein coding
Zfp286-201	11	3.26	-1,479.22	0.08	ENSMUST00000054654	protein coding
Zfp286-202	11	3.44	-1,008.82	0.11	ENSMUST00000108705	protein coding
Zfp286-211	11	0.07	-18.84	0.55	ENSMUST00000207597	protein coding

Zfp296-201	7	41.55	1.52	0.02	ENSMUST00000108453	protein coding
Zfp317-201	9	8.82	-1.29	0.44	ENSMUST00000079042	protein coding
Zfp317-203	9	9.88	1.12	0.74	ENSMUST00000213725	protein coding
Zfp317-205	9	2.51	1.31	0.41	ENSMUST00000215372	protein coding
Zfp322a-201	13	20.05	1.49	0.06	ENSMUST00000050101	protein coding
Zfp322a-202	13	7.68	1.46	0.3	ENSMUST00000125328	protein coding
Zfp422-201	6	16.68	1.16	0.41	ENSMUST00000057540	protein coding
Zfp422-202	6	0.76	3.34	0.14	ENSMUST00000079749	protein coding
Zfp422-203	6	0.48	-1.51	0.82	ENSMUST00000112880	protein coding
Zfp423-202	8	2.89	1.57	0.18	ENSMUST00000109655	protein coding
Zfp423-207	8	3.81	-7.45	0.06	ENSMUST00000174764	protein coding
Zfp438-202	18	2.5	-1.11	0.78	ENSMUST00000234241	protein coding
Zfp445-201	9	0.31	-139.51	0.3	ENSMUST00000056467	protein coding
Zfp445-202	9	8.58	-1.73	0.17	ENSMUST00000213971	protein coding
Zfp445-204	9	0.42	2.56	0.38	ENSMUST00000214558	protein coding
Zfp445-205	9	0.09	3.26	0.5	ENSMUST00000214626	protein coding
Zfp445-206	9	41.14	1.27	0.02	ENSMUST00000216063	protein coding
Zfp445-208	9	12.09	1.26	0.51	ENSMUST00000216721	protein coding
Zfp451-201	1	53.65	1.11	0.5	ENSMUST00000019861	protein coding
Zfp451-206	1	2.27	1.81	0.64	ENSMUST00000139143	nonsense mediated decay
Zfp451-208	1	1.08	1.22	0.86	ENSMUST00000151055	nonsense mediated decay
Zfp451-209	1	1.34	1.23	0.86	ENSMUST00000194656	protein coding
Zfp454-202	11	0.73	-3.9	0.33	ENSMUST00000109131	protein coding
Zfp454-206	11	3.19	-2.95	0.07	ENSMUST00000163301	protein coding
Zfp456-201	13	2.36	-2.28	0.22	ENSMUST00000057070	protein coding
Zfp456-202	13	0.09	-10.14	0.63	ENSMUST00000172266	protein coding

Zfp456-203	13	0.12	-7.96	0.67	ENSMUST00000225787	protein coding
Zfp459-203	13	3.53	-336.73	0.19	ENSMUST00000224113	protein coding
Zfp488-201	14	0.14	-1.34	0.8	ENSMUST00000166737	protein coding
Zfp493-201	13	7.57	-1.7	0.02	ENSMUST00000164936	protein coding
Zfp493-203	13	0.45	-4.07	0.46	ENSMUST00000181319	protein coding
Zfp493-204	13	0.06	8.44	0.31	ENSMUST00000220570	protein coding
Zfp518a-201	19	28.92	-1.53	0.05	ENSMUST00000050092	protein coding
Zfp518a-202	19	1.03	-974.23	0.13	ENSMUST00000235207	protein coding
Zfp518b-202	5	1.74	-1.4	0.81	ENSMUST00000178760	protein coding
Zfp532-201	18	3.2	-4.63	0.15	ENSMUST00000049016	protein coding
Zfp532-202	18	3.81	1.23	0.39	ENSMUST00000169679	protein coding
Zfp532-203	18	0.87	-97.17	0.32	ENSMUST00000182140	protein coding
Zfp532-206	18	3.17	-168.47	0.26	ENSMUST00000182655	protein coding
Zfp532-207	18	0.32	-44.95	0.43	ENSMUST00000182684	protein coding
Zfp532-208	18	7.52	-2.43	0.23	ENSMUST00000182852	protein coding
Zfp532-209	18	0.38	2.25	0.63	ENSMUST00000182973	protein coding
Zfp532-212	18	0.09	-10.14	0.63	ENSMUST00000183236	protein coding
Zfp532-214	18	1.85	-8.05	0.21	ENSMUST00000183319	protein coding
Zfp532-215	18	0.14	-10.46	0.63	ENSMUST00000183326	protein coding
Zfp532-216	18	8.79	-1.54	0.1	ENSMUST00000235493	protein coding
Zfp532-218	18	0.68	1.15	0.81	ENSMUST00000235822	protein coding
Zfp532-219	18	0.26	-1.94	0.76	ENSMUST00000236523	protein coding
Zfp532-220	18	0.43	-36.25	0.46	ENSMUST00000236633	protein coding
Zfp532-222	18	2.5	-2.22	0.07	ENSMUST00000237553	protein coding
Zfp532-223	18	0.94	-2.01	0.52	ENSMUST00000237712	protein coding
Zfp532-226	18	1.54	-136.34	0.3	ENSMUST00000237886	protein coding

Zfp532-227	18	0.09	2.87	0.57	ENSMUST00000237931	protein coding
Zfp536-203	7	0.43	-3.52	0.17	ENSMUST00000176114	protein coding
Zfp536-204	7	0.55	-5.99	0.07	ENSMUST00000176129	protein coding
Zfp536-208	7	0.03	8.44	0.31	ENSMUST00000176534	protein coding
Zfp536-211	7	0.01	23.93	0.1	ENSMUST00000206763	protein coding
Zfp551-202	7	1.73	-559.56	0.15	ENSMUST00000120220	protein coding
Zfp551-203	7	0.12	-7.91	0.67	ENSMUST00000123877	nonsense mediated decay
Zfp553-201	7	37.02	1.34	0.2	ENSMUST00000056232	protein coding
Zfp558-202	9	1.91	1.1	0.92	ENSMUST00000034647	protein coding
Zfp558-203	9	0.25	1.21	0.84	ENSMUST00000159596	nonsense mediated decay
Zfp566-201	7	20.83	1.27	0.33	ENSMUST00000088785	protein coding
Zfp57-204	17	0.66	-42.63	0.44	ENSMUST00000172527	protein coding
Zfp57-206	17	2.63	-2.21	0.41	ENSMUST00000172580	protein coding
Zfp57-207	17	1.13	-4.8	0.29	ENSMUST00000173588	protein coding
Zfp57-208	17	0.56	-2.09	0.71	ENSMUST00000173921	protein coding
Zfp57-213	17	1.66	-1.2	0.88	ENSMUST00000174747	protein coding
Zfp583-201	7	5.03	1.01	0.98	ENSMUST00000062765	protein coding
Zfp583-202	7	0.12	2.41	0.63	ENSMUST00000108560	protein coding
Zfp583-204	7	0.16	9.27	0.2	ENSMUST00000127658	protein coding
Zfp583-206	7	1.76	-449.05	0.17	ENSMUST00000165705	protein coding
Zfp59-201	7	0.02	-5.79	0.71	ENSMUST00000108331	protein coding
Zfp60-201	7	3.75	1.3	0.65	ENSMUST00000042641	protein coding
Zfp60-202	7	7.87	1.7	1.42E-05	ENSMUST00000108336	protein coding
Zfp60-205	7	0.08	16.19	0.16	ENSMUST00000130997	protein coding
Zfp60-206	7	0.04	2.09	0.69	ENSMUST00000136373	nonsense mediated decay
Zfp60-208	7	5.25	-1.41	0.46	ENSMUST00000167955	protein coding

Zfp607a-202	7	5.52	-2,869.66	0.06	ENSMUST00000205534	protein coding
Zfp607a-203	7	0.17	-8.53	0.23	ENSMUST00000205715	protein coding
Zfp607b-201	7	1.16	-1.34	0.71	ENSMUST00000076421	protein coding
Zfp61-201	7	7.02	1.75	0.06	ENSMUST00000077780	protein coding
Zfp61-207	7	0.42	-58.52	0.4	ENSMUST00000145131	protein coding
Zfp61-208	7	6.92	1.31	0.5	ENSMUST00000165241	protein coding
Zfp619-201	7	2.3	-1.02	0.95	ENSMUST00000108015	protein coding
Zfp62-202	11	0.8	1.74	0.59	ENSMUST00000109197	protein coding
Zfp62-203	11	6.97	1.39	0.39	ENSMUST00000109198	protein coding
Zfp62-205	11	0.44	3.34	0.3	ENSMUST00000133150	protein coding
Zfp62-207	11	1.28	1.02	0.98	ENSMUST00000136691	protein coding
Zfp62-208	11	0.04	8.44	0.31	ENSMUST00000137061	protein coding
Zfp644-201	5	11.05	1.7	5.47E-03	ENSMUST00000045466	protein coding
Zfp644-203	5	0.53	4.81	0.05	ENSMUST00000112695	protein coding
Zfp644-204	5	11.32	1.1	0.62	ENSMUST00000112696	protein coding
Zfp644-205	5	0.02	37.4	0.08	ENSMUST00000112698	protein coding
Zfp644-206	5	0.1	-12.15	0.62	ENSMUST00000122980	protein coding
Zfp644-207	5	0.69	-1.52	0.77	ENSMUST00000124263	protein coding
Zfp644-209	5	1.41	1.32	0.74	ENSMUST00000127434	protein coding
Zfp644-212	5	1.33	-1.08	0.96	ENSMUST00000135108	protein coding
Zfp65-201	13	8.8	1.23	0.42	ENSMUST00000073157	protein coding
Zfp65-202	13	0.2	1.46	0.82	ENSMUST00000127979	protein coding
Zfp65-203	13	4.89	1.22	0.76	ENSMUST00000130891	protein coding
Zfp654-202	16	12.83	1.45	7.46E-03	ENSMUST00000207826	protein coding
Zfp711-201	X	0.71	-4.44	0.16	ENSMUST00000071814	protein coding
Zfp711-202	X	0.15	-100.36	0.33	ENSMUST00000113409	protein coding

Zfp711-203	X	0.4	-41.9	0.44	ENSMUST00000122805	protein coding
Zfp712-201	13	1.5	1.23	0.77	ENSMUST00000167565	protein coding
Zfp729a-201	13	4.18	1.54	2.69E-07	ENSMUST00000012314	protein coding
Zfp74-202	7	9.25	1.68	2.09E-07	ENSMUST00000108205	protein coding
Zfp74-204	7	1.52	-1.09	0.93	ENSMUST00000108212	protein coding
Zfp740-201	15	66.82	1.21	0.06	ENSMUST00000118729	protein coding
Zfp740-202	15	7.94	1.7	8.31E-03	ENSMUST00000119168	protein coding
Zfp740-203	15	34.88	-1.17	0.58	ENSMUST00000119800	protein coding
Zfp740-208	15	9.31	1.3	0.64	ENSMUST00000139960	protein coding
Zfp740-209	15	4.4	1.16	0.78	ENSMUST00000141465	protein coding
Zfp740-210	15	2.43	1.25	0.78	ENSMUST00000229886	protein coding
Zfp740-212	15	0.34	-38.72	0.45	ENSMUST00000230867	protein coding
Zfp759-201	13	4.5	-1.1	0.82	ENSMUST00000052716	protein coding
Zfp763-201	17	8	1.38	0.39	ENSMUST00000087654	protein coding
Zfp784-201	7	0.2	1.24	0.86	ENSMUST00000062428	protein coding
Zfp827-201	8	0.12	1.47	0.78	ENSMUST00000087927	protein coding
Zfp827-202	8	2.02	-2.13	0.11	ENSMUST00000098614	protein coding
Zfp827-204	8	0.12	-14.61	0.59	ENSMUST00000129613	protein coding
Zfp827-205	8	0.71	-2.28	0.48	ENSMUST00000145827	nonsense mediated decay
Zfp827-206	8	0.08	3.68	0.46	ENSMUST00000148713	protein coding
Zfp827-209	8	0.99	-1.99	0.51	ENSMUST00000238669	protein coding
Zfp84-201	7	8.97	1.79	1.91E-11	ENSMUST00000032802	protein coding
Zfp850-203	7	18.32	-1.34	0.05	ENSMUST00000180502	protein coding
Zfp87-201	13	0.73	-7.85	0.27	ENSMUST00000180536	nonsense mediated decay
Zfp87-202	13	0.03	-3.61	0.78	ENSMUST00000181341	protein coding
Zfp87-203	13	1.73	2.37	0.14	ENSMUST00000181573	protein coding

Zfp87-204	13	11.78	1.53	0.06	ENSMUST00000181767	protein coding
Zfp874a-201	13	1.18	-2.91	0.28	ENSMUST00000057241	protein coding
Zfp874a-202	13	3.05	1.53	0.25	ENSMUST00000075255	protein coding
Zfp874a-203	13	0.4	-1.55	0.81	ENSMUST00000180580	nonsense mediated decay
Zfp874a-204	13	2.43	-1.42	0.71	ENSMUST00000223682	nonsense mediated decay
Zfp874a-208	13	3.34	-1.14	0.89	ENSMUST00000225479	protein coding
Zfp874a-209	13	1.09	-1.57	0.72	ENSMUST00000225778	protein coding
Zfp874b-201	13	8.19	1.81	2.84E-05	ENSMUST00000019572	protein coding
Zfp874b-203	13	2.72	-1.09	0.91	ENSMUST00000223868	protein coding
Zfp882-201	8	3.81	-1.84	0.15	ENSMUST00000110002	protein coding
Zfp882-203	8	0.72	-1.51	0.72	ENSMUST00000126607	protein coding
Zfp93-201	7	5.81	1.22	0.69	ENSMUST00000032696	protein coding
Zfp945-204	17	1.38	1.6	0.18	ENSMUST00000160457	protein coding
Zfp953-201	13	3.27	1.13	0.78	ENSMUST00000081582	protein coding
Zfp979-202	4	3.57	-1,361.53	0.09	ENSMUST00000105720	protein coding
Zfp979-204	4	1.66	-144.12	0.3	ENSMUST00000133006	protein coding
Zfp981-203	4	1.32	-576.96	0.14	ENSMUST00000179175	protein coding
Zfp993-201	4	11.27	-656.41	0.15	ENSMUST00000070932	protein coding
Zic3-201	X	17.99	1.21	0.54	ENSMUST00000088627	protein coding
Zic3-202	X	2.9	-1.02	0.96	ENSMUST00000088629	protein coding
Zic3-204	X	1.49	-128.13	0.3	ENSMUST00000137687	protein coding
Zkscan16-201	4	2.37	-1.32	0.65	ENSMUST00000107554	protein coding
Zkscan2-201	7	1.05	1.5	0.44	ENSMUST00000042470	protein coding
Zkscan2-203	7	0.47	3.19	0.16	ENSMUST00000128217	protein coding
Zkscan4-201	13	0.58	1.17	0.84	ENSMUST00000062609	protein coding
Zkscan4-202	13	0.11	6.02	0.29	ENSMUST00000225845	protein coding

Zkscan7-201	9	1.2	1.05	0.94	ENSMUST00000063980	protein coding
Zkscan7-202	9	0.28	1.83	0.42	ENSMUST00000215872	protein coding
Zscan10-201	17	49	1.56	5.82E-03	ENSMUST00000095595	protein coding
Zscan10-203	17	0.08	16.71	0.16	ENSMUST00000117606	protein coding
Zscan10-204	17	2.21	-1.06	0.93	ENSMUST00000118369	protein coding
Zscan10-205	17	32.18	-1.45	0.06	ENSMUST00000120967	protein coding
Zscan10-207	17	3.84	1.71	0.28	ENSMUST00000123866	protein coding
Zscan10-209	17	0.24	-67.46	0.38	ENSMUST00000129227	protein coding
Zscan10-211	17	1.01	-5.78	0.32	ENSMUST00000138487	protein coding
Zscan18-204	7	1.23	2.62	0.41	ENSMUST00000210891	nonsense mediated decay
Zscan5b-201	7	0.7	-205.19	0.23	ENSMUST00000072662	protein coding
Zscan5b-203	7	0.09	-1.56	0.82	ENSMUST00000165445	protein coding
Zup1-201	10	0.62	6.54	0.12	ENSMUST00000048222	protein coding
Zup1-202	10	8.26	1.52	0.04	ENSMUST00000218055	protein coding
Zup1-203	10	0.12	23.27	0.12	ENSMUST00000218222	protein coding
Zup1-204	10	1.81	-1.03	0.98	ENSMUST00000218275	nonsense mediated decay
Zup1-205	10	0.24	5.48	0.26	ENSMUST00000218880	protein coding
Zup1-206	10	3.86	-1.07	0.9	ENSMUST00000218892	protein coding
Zup1-208	10	0.08	-1.23	0.9	ENSMUST00000219878	nonsense mediated decay

S.3. Transcript Variants (TE) of Insignificant Genes (GE)

Table S3-1. Expression values & metadata of upregulated transcript variants of insignificant genes

Name	Chromosome	Max group mean	Fold change	FDR p-value	ENSEMBL	Biotype
Adnp-202	2	7.83	8.6	2.37E-22	ENSMUST00000088001	protein coding
Aebp2-202	6	14.56	17.59	3.77E-10	ENSMUST00000087614	protein coding
Akap8-205	17	12.63	5,709.50	1.08E-07	ENSMUST00000235764	nonsense mediated decay
Arid2-202	15	2.41	254.72	2.43E-03	ENSMUST00000134985	protein coding
Arnt-203	3	1.51	12.57	0.03	ENSMUST00000107160	protein coding
Arnt2-201	7	1.18	933.15	1.60E-03	ENSMUST00000085077	protein coding
Ash1l-202	3	5.39	7.12	1.37E-14	ENSMUST00000186583	protein coding
Atf2-202	2	1.97	154.75	9.86E-06	ENSMUST00000090802	protein coding
Atf2-203	2	3.58	1,806.67	3.83E-04	ENSMUST00000100009	protein coding
Atf2-206	2	14.03	3.79	9.96E-08	ENSMUST00000112016	protein coding
Bach2-202	4	0.82	194.46	9.12E-05	ENSMUST00000108180	protein coding
Baz2a-208	10	8.42	2.35	5.40E-03	ENSMUST00000220049	protein coding
Baz2a-209	10	6.24	8.42	1.44E-12	ENSMUST00000238829	protein coding
Bbx-201	16	4.42	3.84	4.27E-05	ENSMUST00000066037	protein coding
Bmal1-203	7	0.84	79.54	1.12E-03	ENSMUST00000210074	protein coding
Bptf-202	11	0.9	5.36	0.03	ENSMUST00000106762	protein coding
Bptf-203	11	10.78	2.18	2.84E-11	ENSMUST00000106763	protein coding
Bptf-208	11	26.2	2.64	1.21E-07	ENSMUST00000208369	protein coding
Camta1-201	4	2.73	2.07	1.93E-03	ENSMUST00000049790	protein coding
Camta2-203	11	0.46	280.08	2.56E-03	ENSMUST00000108544	protein coding
Camta2-205	11	7.49	2.14	0.01	ENSMUST00000119120	protein coding
Cc2d1a-201	8	24.62	2.15	4.32E-05	ENSMUST00000040383	protein coding
Cc2d1a-202	8	1.81	24.11	2.94E-05	ENSMUST00000117424	protein coding

Cenpa-204	5	3.38	64	1.76E-04	ENSMUST00000134846	nonsense mediated decay
Cenpa-209	5	4.2	14.57	0.04	ENSMUST00000199320	protein coding
Cic-212	7	13.85	2.79	3.41E-09	ENSMUST00000169266	protein coding
Cic-213	7	3.13	244.81	3.34E-03	ENSMUST00000169392	protein coding
Creb5-203	6	0.15	147.63	0.02	ENSMUST00000203528	protein coding
Crem-201	18	0.76	287.56	2.67E-03	ENSMUST00000025069	protein coding
Crem-203	18	0.83	306.85	2.46E-03	ENSMUST00000082141	protein coding
Crem-227	18	1.96	46.7	7.03E-05	ENSMUST00000149803	protein coding
Crem-234	18	1.5	381.93	2.14E-03	ENSMUST00000154470	protein coding
Crem-237	18	1.21	227.61	5.34E-03	ENSMUST00000156234	protein coding
Csrnp1-204	9	2.96	1,131.98	6.87E-05	ENSMUST00000215916	protein coding
Csrnp1-205	9	7.44	7.94	6.54E-03	ENSMUST00000216929	protein coding
Cxxc5-206	18	3.88	12.09	4.27E-04	ENSMUST00000237291	protein coding
Dnmt1-203	9	1.68	1,275.46	3.70E-05	ENSMUST00000178110	protein coding
Drap1-201	19	67.33	4.19	2.30E-11	ENSMUST00000025853	protein coding
Drap1-202	19	2.5	235.1	3.88E-03	ENSMUST00000113673	protein coding
Dzip1-201	14	7.2	2.59	1.79E-04	ENSMUST00000004055	protein coding
E2f3-201	13	19	3.21	9.00E-10	ENSMUST00000102948	protein coding
E2f6-201	12	14.85	5.73	1.75E-06	ENSMUST00000020908	protein coding
E2f6-203	12	0.49	21.55	0.01	ENSMUST00000220794	nonsense mediated decay
E4f1-204	17	2.58	849	1.69E-03	ENSMUST00000226754	nonsense mediated decay
E4f1-205	17	4.71	524.32	5.06E-07	ENSMUST00000226941	protein coding
Elf3-201	1	33.55	3.05	1.36E-05	ENSMUST00000003135	protein coding
Elk3-204	10	1.59	8.11	0.04	ENSMUST00000151153	protein coding
Esrrb-206	12	30.36	22.44	5.84E-46	ENSMUST00000167891	protein coding
Flywch1-202	17	3.64	318.02	3.70E-06	ENSMUST00000086325	protein coding

Foxn3-201	12	13.4	2.06	9.61E-08	ENSMUST00000046859	protein coding
Foxn3-209	12	2.32	857.97	1.23E-04	ENSMUST00000177451	protein coding
Foxo3-202	10	3.59	193.75	3.55E-06	ENSMUST00000105501	protein coding
Foxo4-201	X	89.91	2.11	9.54E-27	ENSMUST00000062000	protein coding
Foxo4-202	X	0.64	112.99	0.03	ENSMUST00000138437	protein coding
Gata4-201	14	5.32	10.05	1.04E-07	ENSMUST00000067417	protein coding
Gata4-202	14	6.1	14.15	6.51E-07	ENSMUST00000118022	protein coding
Gatad2a-206	8	2.73	1,279.49	3.91E-05	ENSMUST00000212478	protein coding
Glmp-209	3	20.62	2.51	1.53E-07	ENSMUST00000177005	protein coding
Gmeb1-203	4	3.43	4.39	0.04	ENSMUST00000105965	protein coding
Gmeb2-203	2	0.89	89.95	0.03	ENSMUST00000130475	protein coding
Gmeb2-205	2	0.81	38.05	3.53E-03	ENSMUST00000141110	nonsense mediated decay
Grhl1-202	12	11.5	2.14	2.41E-04	ENSMUST00000085553	protein coding
Gtf2ird1-201	5	1.56	712.65	5.97E-04	ENSMUST00000073161	protein coding
Gtf2ird1-203	5	2.89	1,306.98	3.76E-05	ENSMUST00000100650	protein coding
Gtf2ird1-204	5	2.3	1,085.61	7.72E-05	ENSMUST00000100652	protein coding
Gtf2ird1-209	5	12.55	1,352.22	5.90E-14	ENSMUST00000171794	protein coding
Gtf2ird1-229	5	3.31	13.46	1.05E-05	ENSMUST00000202554	protein coding
Hbp1-205	12	1.37	31.04	1.00E-02	ENSMUST00000176084	protein coding
Hdx-201	X	2.26	3.39	1.79E-05	ENSMUST00000038472	protein coding
Hmg20a-205	9	5.07	4.4	0.02	ENSMUST00000215269	protein coding
Hsf1-204	15	0.75	38.38	1.58E-03	ENSMUST00000226872	protein coding
Hsf1-211	15	1.03	4.6	0.02	ENSMUST00000228757	nonsense mediated decay
Hsf1-213	15	4.68	11.42	2.13E-04	ENSMUST00000228868	nonsense mediated decay
Ikzf4-209	10	0.64	133.44	1.34E-05	ENSMUST00000238610	protein coding
Irf1-201	11	4.19	287.35	1.00E-02	ENSMUST00000019043	protein coding

Irf1-207	11	1.12	73.5	0.04	ENSMUST00000138913	protein coding
Irf3-201	7	8.74	2.75	2.67E-03	ENSMUST00000003284	protein coding
Irf3-213	7	10.07	2.53	0.02	ENSMUST00000209066	protein coding
Kat7-204	11	32.98	7.12	6.90E-32	ENSMUST00000107733	protein coding
Kat7-205	11	37.28	4.86	6.10E-36	ENSMUST00000107734	protein coding
Kat7-212	11	36.51	4.9	9.67E-32	ENSMUST00000249615	protein coding
Kcmf1-206	6	2.09	146.01	0.02	ENSMUST00000206378	protein coding
Kcnip3-203	2	0.92	47.32	8.77E-04	ENSMUST00000103215	protein coding
Kmt2b-202	7	0.14	155.7	0.02	ENSMUST00000108154	protein coding
Lcor-201	19	5.68	2.04	2.40E-04	ENSMUST00000067795	protein coding
Lcorl-202	5	1.15	852.69	1.25E-04	ENSMUST00000045586	protein coding
Lin28a-204	4	1.43	16.76	0.01	ENSMUST00000176897	protein coding
Lin54-202	5	1.3	196.37	2.66E-05	ENSMUST00000239513	protein coding
Lin54-203	5	1.44	11.21	4.65E-03	ENSMUST00000239514	nonsense mediated decay
Lmx1a-201	1	0.18	78.64	0.05	ENSMUST00000028003	protein coding
Mafg-203	11	1.76	185.51	9.58E-03	ENSMUST00000106181	protein coding
Mafg-204	11	17.26	3.37	5.07E-04	ENSMUST00000106182	protein coding
Maz-201	7	26.9	9.69	6.19E-11	ENSMUST00000032916	protein coding
Mbd1-202	18	10.94	3.16	1.01E-07	ENSMUST00000224047	protein coding
Mbd6-202	10	12.33	2.07	4.70E-03	ENSMUST00000119078	protein coding
Mlx-201	11	25.84	4.62	9.44E-09	ENSMUST00000017945	protein coding
Mlxipl-208	5	1.51	20.94	0.02	ENSMUST00000154840	protein coding
Myc-206	15	24.51	4.53	2.37E-03	ENSMUST00000167731	protein coding
Mzf1-201	7	0.2	96.54	0.02	ENSMUST00000069289	protein coding
Mzf1-202	7	0.44	121.05	0.03	ENSMUST00000182087	protein coding
Nacc2-202	2	0.28	244.54	3.22E-03	ENSMUST00000114159	protein coding

Ncoa2-203	1	3.5	2.23	3.02E-03	ENSMUST00000081713	protein coding
Nfat5-201	8	0.51	304.88	0.02	ENSMUST00000075922	protein coding
Nfat5-203	8	0.71	539.7	1.33E-03	ENSMUST00000125721	protein coding
Nfat5-206	8	5.56	2.57	0.01	ENSMUST00000133026	nonsense mediated decay
Nfat5-214	8	5.22	2,123.60	3.69E-15	ENSMUST00000169453	protein coding
Nfe2l1-201	11	73.18	3.03	6.50E-74	ENSMUST00000081775	protein coding
Nfe2l1-203	11	7.15	25.81	2.05E-08	ENSMUST00000107658	protein coding
Nfya-212	17	4.8	2,299.23	5.94E-06	ENSMUST00000162460	protein coding
Nfyb-205	10	4.91	65	8.49E-05	ENSMUST00000142523	protein coding
Nfyc-201	4	53.28	3.03	5.50E-09	ENSMUST00000043429	protein coding
Nme2-201	11	834.13	3.15	2.88E-45	ENSMUST00000021217	protein coding
Nr1h2-203	7	14.28	3,663.92	4.69E-05	ENSMUST00000107911	protein coding
Nr1h2-204	7	1.78	416.34	1.97E-03	ENSMUST00000107912	protein coding
Nr3c1-203	18	2.66	2,206.70	2.50E-04	ENSMUST00000115567	protein coding
Nr4a1-202	15	12.97	2.04	4.17E-03	ENSMUST00000228985	protein coding
Nr6a1-201	2	24.36	2.24	2.61E-06	ENSMUST00000076275	protein coding
Nr6a1-202	2	14.99	27.06	6.04E-49	ENSMUST00000112877	protein coding
Nrf1-206	6	1.08	10.54	0.01	ENSMUST00000115204	protein coding
Nrf1-211	6	5.26	2.05	0.05	ENSMUST00000115212	protein coding
Pa2g4-202	10	417.87	6.67	5.63E-146	ENSMUST00000131728	protein coding
Pax6-208	2	0.16	9.91	0.05	ENSMUST00000111088	protein coding
Pbx2-201	17	43.52	2.18	3.30E-11	ENSMUST00000038149	protein coding
Pbx2-205	17	5.2	800.76	1.79E-04	ENSMUST00000183827	protein coding
Phf1-201	17	2.98	10.81	9.19E-04	ENSMUST00000073724	protein coding
Pknox1-209	17	1.25	72.43	0.04	ENSMUST00000235869	protein coding
Pknox1-210	17	1.22	30.75	4.80E-03	ENSMUST00000236493	protein coding

Pou2f1-209	1	0.7	230.03	9.28E-03	ENSMUST00000160908	protein coding
Pou2f2-203	7	0.16	46.63	0.01	ENSMUST00000108415	protein coding
Pou6f1-201	15	1.82	4.57	6.55E-03	ENSMUST00000073837	protein coding
Pou6f1-203	15	1.9	232.46	1.47E-06	ENSMUST00000176271	protein coding
Pou6f1-205	15	0.7	254.69	2.57E-03	ENSMUST00000177202	nonsense mediated decay
Prdm15-201	16	0.71	122.61	9.25E-05	ENSMUST00000095849	protein coding
Prrx2-201	2	1.72	5.63	0.02	ENSMUST00000041659	protein coding
Rara-201	11	0.21	91.68	0.03	ENSMUST00000068133	protein coding
Rara-204	11	23.25	4.58	1.89E-15	ENSMUST00000107475	protein coding
Rbck1-202	2	68.49	2.8	9.63E-11	ENSMUST00000109847	protein coding
Rela-205	19	1.44	70.17	0.04	ENSMUST00000237863	protein coding
Rela-206	19	1.12	298.47	0.02	ENSMUST00000238000	nonsense mediated decay
Relb-201	7	1.31	8.87	0.03	ENSMUST00000049912	protein coding
Rexo4-202	2	42.32	2.99	2.92E-03	ENSMUST00000114020	protein coding
Rfx1-201	8	19.14	2.1	4.48E-04	ENSMUST00000005600	protein coding
Rfx5-202	3	1.84	5.15	2.72E-03	ENSMUST00000107253	protein coding
Rfx5-207	3	5.96	3,310.82	8.13E-05	ENSMUST00000137088	protein coding
Rlf-203	4	0.55	464.69	7.85E-03	ENSMUST00000168615	protein coding
Rora-201	9	2.01	2.4	5.09E-04	ENSMUST00000034766	protein coding
Rreb1-203	13	4.03	2.44	5.25E-05	ENSMUST00000110238	protein coding
Rreb1-211	13	3.47	6.95	0.03	ENSMUST00000162849	protein coding
Rxrb-201	17	16.69	7	6.89E-09	ENSMUST00000044858	protein coding
Setdb1-201	3	5.36	2.06	8.63E-03	ENSMUST00000015841	protein coding
Ski-201	4	57.98	10.09	2.25E-79	ENSMUST00000030917	protein coding
Skil-201	3	5.48	2.35	2.55E-03	ENSMUST00000029194	protein coding
Skil-205	3	1.48	137.88	0.02	ENSMUST00000123532	protein coding

Smad4-201	18	77.96	2.09	4.88E-26	ENSMUST00000025393	protein coding
Smad5-202	13	1.18	60.28	4.59E-04	ENSMUST00000109874	protein coding
Snapc4-201	2	15.23	23.48	1.62E-08	ENSMUST00000035427	protein coding
Son-203	16	9.7	4.26	2.59E-15	ENSMUST00000117633	protein coding
Son-205	16	1.74	554.99	6.57E-03	ENSMUST00000122302	protein coding
Sox13-205	1	13.3	4.87	4.90E-11	ENSMUST00000153799	protein coding
Sp3-202	2	47.97	19.89	5.50E-54	ENSMUST00000102689	protein coding
Spen-202	4	8.25	2.01	6.00E-05	ENSMUST00000105786	protein coding
Srebf1-201	11	40.01	2.55	1.89E-09	ENSMUST00000020846	protein coding
Stat3-202	11	1.38	3.29	0.04	ENSMUST00000103114	protein coding
Tcf12-201	9	10.65	5.64	3.24E-11	ENSMUST00000034755	protein coding
Tcf20-203	15	6.88	9.87	3.75E-04	ENSMUST00000229439	protein coding
Tcf20-205	15	6.54	43.63	4.54E-05	ENSMUST00000230403	protein coding
Tcf3-202	10	25.07	2.31	0.01	ENSMUST00000020379	protein coding
Tcf3-207	10	11.17	40.22	1.44E-20	ENSMUST00000105343	protein coding
Tcf3-210	10	13.36	467.61	1.30E-23	ENSMUST00000105346	protein coding
Tcf7l2-211	19	0.53	33.31	5.30E-03	ENSMUST00000111658	protein coding
Tead1-201	7	3.15	18	2.43E-15	ENSMUST00000059768	protein coding
Tead1-207	7	1.53	241.68	3.42E-03	ENSMUST00000165036	protein coding
Tead1-211	7	1.46	39.29	6.36E-04	ENSMUST00000170352	protein coding
Tead1-212	7	0.62	89.94	0.03	ENSMUST00000171197	protein coding
Tead1-216	7	10.53	441.92	6.04E-49	ENSMUST00000239404	protein coding
Tead1-217	7	3.42	15.46	9.59E-23	ENSMUST00000239442	protein coding
Tead2-203	7	9.58	4.51	1.77E-17	ENSMUST00000107801	protein coding
Tead2-206	7	4.82	28.5	2.54E-04	ENSMUST00000209437	protein coding
Tead2-211	7	3.35	16.35	1.34E-03	ENSMUST00000210447	protein coding

Tead2-216	7	5.84	61.59	2.33E-04	ENSMUST00000211744	protein coding
Tef-201	15	20.71	2.54	9.71E-07	ENSMUST00000023024	protein coding
Tfap2a-206	13	0.47	121.15	0.03	ENSMUST00000224665	protein coding
Tfcp2-203	15	2.05	5.69	0.03	ENSMUST00000229581	protein coding
Tfdp2-204	9	4.79	11.45	2.01E-04	ENSMUST00000179065	protein coding
Tfe3-201	X	34.95	6.37	3.91E-14	ENSMUST00000077680	protein coding
Tfe3-202	X	1.46	71.38	0.04	ENSMUST00000079542	protein coding
Tfe3-207	X	0.59	237.71	0.02	ENSMUST00000115680	nonsense mediated decay
Tfe3-211	X	0.98	77.83	6.32E-04	ENSMUST00000137467	protein coding
Tfeb-201	17	14.42	4.15	1.08E-04	ENSMUST00000024786	protein coding
Thap7-201	16	8.58	5.65	3.50E-04	ENSMUST00000100125	protein coding
Thap7-211	16	1.13	170.5	0.01	ENSMUST00000232114	nonsense mediated decay
Tigd3-202	19	1.32	92.46	9.17E-04	ENSMUST00000236767	protein coding
Trp73-201	4	0.36	20.18	0.04	ENSMUST00000097762	protein coding
Ubp1-202	9	26.29	2.72	2.47E-07	ENSMUST00000084885	protein coding
Ubp1-203	9	2.97	165.46	9.03E-07	ENSMUST00000214095	protein coding
Usf2-202	7	12.24	9.45	1.07E-04	ENSMUST00000108119	protein coding
Ybx2-201	11	2.53	5.66	0.02	ENSMUST00000018698	protein coding
Zbtb14-202	17	6.11	3.33	3.32E-03	ENSMUST00000112674	protein coding
Zbtb14-203	17	2.55	4.07	7.33E-03	ENSMUST00000112676	protein coding
Zbtb18-212	1	4.27	2.61	0.04	ENSMUST00000195612	protein coding
Zbtb2-202	10	3.21	1,326.51	3.28E-05	ENSMUST00000100078	protein coding
Zbtb21-203	16	3.03	4.15	7.35E-04	ENSMUST00000113734	protein coding
Zbtb21-205	16	0.58	69.9	0.05	ENSMUST00000231263	protein coding
Zbtb21-208	16	2.45	22.45	1.19E-04	ENSMUST00000232187	protein coding
Zbtb34-202	2	4.72	4,458.32	3.43E-07	ENSMUST00000113158	protein coding

Zbtb44-201	9	16.57	3.22	2.77E-22	ENSMUST00000115222	protein coding
Zbtb5-204	4	0.31	192.23	0.01	ENSMUST00000180217	protein coding
Zbtb6-202	2	1.97	10.51	2.52E-03	ENSMUST00000112932	protein coding
Zbtb7a-202	10	8.84	2,129.48	6.26E-06	ENSMUST00000117956	protein coding
Zbtb7c-202	18	0.26	73.48	0.04	ENSMUST00000167921	protein coding
Zfand5-205	19	3.03	4.25	3.58E-07	ENSMUST00000237651	protein coding
Zfp12-201	5	2.15	2.81	0.03	ENSMUST00000032591	protein coding
Zfp12-203	5	2.16	1,197.00	6.79E-04	ENSMUST00000077485	protein coding
Zfp131-203	13	8.49	8.28	4.59E-04	ENSMUST00000223722	protein coding
Zfp160-202	17	0.69	189.15	0.01	ENSMUST00000231482	protein coding
Zfp160-205	17	0.59	74.73	0.04	ENSMUST00000232354	protein coding
Zfp207-203	11	2.46	3.69	0.02	ENSMUST00000108216	protein coding
Zfp207-204	11	17.3	2.53	7.40E-06	ENSMUST00000123726	nonsense mediated decay
Zfp207-207	11	11.54	2.26	4.48E-05	ENSMUST00000178665	nonsense mediated decay
Zfp207-208	11	11.92	2.24	1.72E-04	ENSMUST00000188489	protein coding
Zfp217-201	2	20.45	7.12	2.01E-23	ENSMUST00000063710	protein coding
Zfp219-204	14	32.82	3,759.76	2.39E-13	ENSMUST00000226522	protein coding
Zfp236-206	18	4	3.59	2.92E-03	ENSMUST00000183324	nonsense mediated decay
Zfp275-201	X	6.51	2.05	2.64E-03	ENSMUST00000033731	protein coding
Zfp280c-202	X	3.97	2.09	0.04	ENSMUST00000076635	protein coding
Zfp280d-201	9	10.24	1,215.12	1.59E-16	ENSMUST00000098576	protein coding
Zfp280d-205	9	3.09	113.11	7.38E-06	ENSMUST00000183468	protein coding
Zfp324-203	7	0.37	46.59	3.82E-03	ENSMUST00000128293	nonsense mediated decay
Zfp326-206	5	3.05	6.25	0.03	ENSMUST00000138615	nonsense mediated decay
Zfp335-203	2	0.19	112.08	0.04	ENSMUST00000183830	nonsense mediated decay
Zfp346-203	13	0.78	127.68	0.03	ENSMUST00000159278	protein coding

Zfp37-205	4	1.48	65.42	4.47E-04	ENSMUST00000221329	protein coding
Zfp385a-203	15	13.89	2.84	1.19E-03	ENSMUST00000229551	protein coding
Zfp397-204	18	2.75	6.65	0.04	ENSMUST00000225682	protein coding
Zfp410-201	12	19.05	12.23	2.44E-14	ENSMUST00000045931	protein coding
Zfp426-202	9	4.04	11.28	1.51E-05	ENSMUST00000115562	protein coding
Zfp426-212	9	0.96	89.07	0.03	ENSMUST00000169269	protein coding
Zfp446-202	7	9.4	3.08	4.70E-06	ENSMUST00000108535	protein coding
Zfp462-201	4	7.78	3.95	4.60E-04	ENSMUST00000030131	protein coding
Zfp513-202	5	22.21	3.13	9.74E-06	ENSMUST00000114590	protein coding
Zfp523-202	17	3.66	374.4	1.14E-06	ENSMUST00000073534	protein coding
Zfp574-202	7	2.99	4.31	1.09E-03	ENSMUST00000179556	protein coding
Zfp598-202	17	38.26	2.03	2.79E-03	ENSMUST00000234956	protein coding
Zfp637-208	6	0.78	70.17	0.04	ENSMUST00000136889	protein coding
Zfp64-201	2	72.33	2.04	7.43E-09	ENSMUST00000087971	protein coding
Zfp64-203	2	5.78	2.16	0.03	ENSMUST00000109162	protein coding
Zfp646-204	7	3.84	2.5	6.32E-04	ENSMUST00000206340	protein coding
Zfp652-203	11	0.73	92.8	3.91E-05	ENSMUST00000133070	nonsense mediated decay
Zfp655-206	5	4.51	3.43	0.03	ENSMUST00000200039	protein coding
Zfp667-201	7	6.71	4.98	9.25E-08	ENSMUST00000086327	protein coding
Zfp667-205	7	0.58	277.77	2.57E-03	ENSMUST00000170776	protein coding
Zfp668-203	7	2.78	187.35	7.58E-07	ENSMUST00000106262	protein coding
Zfp687-205	3	5.1	5.34	2.33E-04	ENSMUST00000137799	nonsense mediated decay
Zfp688-206	7	12.03	2.86	0.02	ENSMUST00000148483	nonsense mediated decay
Zfp7-205	15	46.58	3.4	1.88E-24	ENSMUST00000230106	protein coding
Zfp777-201	6	4.13	1,686.48	5.86E-04	ENSMUST00000095944	protein coding
Zfp800-202	6	1.44	804.97	1.46E-04	ENSMUST00000115320	protein coding

Zfp853-203	5	0.42	33.87	4.65E-03	ENSMUST00000212715	protein coding
Zfp90-201	8	1.37	463.77	6.90E-04	ENSMUST00000034382	protein coding
Zfp943-202	17	1.62	11.09	2.11E-03	ENSMUST00000153985	protein coding
Zfp995-205	17	5.85	4.91	4.23E-04	ENSMUST00000190066	protein coding
Zfx-201	X	4.93	4,436.61	5.33E-05	ENSMUST00000088102	protein coding
Zgpat-201	2	4.46	5.81	2.72E-03	ENSMUST00000029105	protein coding
Zgpat-202	2	15.59	2.63	7.49E-04	ENSMUST00000108807	protein coding
Zhx1-202	15	0.3	196.8	0.01	ENSMUST00000110168	protein coding
Zkscan1-203	5	1.13	225.04	4.53E-05	ENSMUST00000110962	protein coding
Zkscan5-201	5	0.9	10.38	0.02	ENSMUST00000031601	protein coding
Zkscan6-201	11	0.68	203.98	0.01	ENSMUST00000018491	protein coding
Zkscan8-201	13	7.13	2.59	1.27E-06	ENSMUST00000045228	protein coding
Zscan21-204	5	12.31	3,497.75	5.32E-05	ENSMUST00000110960	protein coding
Zscan22-204	7	0.55	128.14	0.02	ENSMUST00000120809	protein coding
Zscan29-205	2	2.57	226.06	5.20E-07	ENSMUST00000163766	protein coding
Zxdc-202	6	11.53	2.07	1.08E-04	ENSMUST00000075117	protein coding
Zzz3-202	3	8.06	3.68	6.26E-11	ENSMUST00000106100	protein coding
Zzz3-204	3	9.84	2.04	1.18E-04	ENSMUST00000106103	protein coding
Zzz3-210	3	2.06	6.74	0.01	ENSMUST00000200570	protein coding

Table S3-2. Expression values & metadata of downregulated transcript variants of insignificant genes

Name	Chromosome	Max group mean	Fold change	FDR p-value	ENSEMBL	Biotype
Aebp2-205	6	28.48	-76.39	1.76E-23	ENSMUST00000160836	protein coding
Ahctf1-202	1	5.03	-3.86	0.04	ENSMUST00000125816	nonsense mediated decay
Ahctf1-203	1	12.72	-22.58	8.52E-08	ENSMUST00000127250	nonsense mediated decay
Akap8-206	17	13.56	-7.85	5.39E-10	ENSMUST00000236267	nonsense mediated decay
Akap8l-204	17	1.57	-20.11	0.01	ENSMUST00000235614	nonsense mediated decay
Arnt2-207	7	1.2	-1,111.18	0.01	ENSMUST00000208232	protein coding
Atf2-212	2	8.38	-3.32	0.01	ENSMUST00000136958	protein coding
Atf2-219	2	7.56	-1,925.86	7.49E-04	ENSMUST00000173010	nonsense mediated decay
Atf3-203	1	8.64	-2,553.40	2.49E-04	ENSMUST00000195117	protein coding
Bach2-207	4	0.32	-416.83	0.04	ENSMUST00000171600	protein coding
Baz2a-203	10	5.85	-3.28	3.45E-03	ENSMUST00000217851	protein coding
Bbx-205	16	1.28	-8.18	5.56E-03	ENSMUST00000114488	protein coding
Bmal1-206	7	9.71	-7.36	1.01E-13	ENSMUST00000211770	protein coding
Bptf-207	11	6.7	-4.41	0.02	ENSMUST00000149486	protein coding
Camta1-203	4	1.26	-135.56	1.79E-05	ENSMUST00000105668	protein coding
Carf-201	1	2.07	-10.54	4.47E-04	ENSMUST00000027171	protein coding
Cenpa-201	5	60.08	-9,249.44	9.86E-06	ENSMUST00000031073	nonsense mediated decay
Cenpa-210	5	21.92	-2.22	2.80E-03	ENSMUST00000199617	nonsense mediated decay
Cenps-206	4	9.33	-12.02	2.62E-04	ENSMUST00000176124	protein coding
Champ1-202	8	11.16	-4.06	3.37E-09	ENSMUST00000128557	protein coding
Chchd3-202	6	5.28	-3.77	0.02	ENSMUST00000115091	protein coding
Cic-204	7	5.84	-413.51	8.48E-03	ENSMUST00000163901	protein coding
Creb1-209	1	11.34	-5.91	1.29E-03	ENSMUST00000190348	protein coding
Deaf1-202	7	6.15	-565.33	5.18E-03	ENSMUST00000209397	nonsense mediated decay

Dmtf1-207	5	13.93	-5,206.54	3.80E-05	ENSMUST00000183973	protein coding
Dnmt1-207	9	1.01	-876.36	2.13E-03	ENSMUST00000216540	protein coding
Drap1-209	19	83.91	-2.07	3.56E-05	ENSMUST00000136579	protein coding
Dzip1-202	14	2.05	-7.52	3.67E-05	ENSMUST00000047208	protein coding
E2f3-203	13	12.55	-4.64	9.28E-09	ENSMUST00000221536	protein coding
E4f1-202	17	1.53	-10.92	3.88E-03	ENSMUST00000226654	nonsense mediated decay
Erf-202	7	26.32	-4.84	2.05E-06	ENSMUST00000116343	protein coding
Foxn3-202	12	2.58	-28.29	1.76E-04	ENSMUST00000085108	protein coding
Foxn3-207	12	1.76	-45.33	3.90E-03	ENSMUST00000177269	protein coding
Gata4-203	14	6.78	-5.32	1.02E-03	ENSMUST00000121312	protein coding
Gatad2b-201	3	14.59	-2.25	3.78E-04	ENSMUST00000049382	protein coding
Gatad2b-204	3	2.16	-5.22	0.04	ENSMUST00000199607	protein coding
Gmeb1-201	4	2.5	-968.78	0.02	ENSMUST00000030733	protein coding
Gmeb1-207	4	7.16	-7.04	4.22E-24	ENSMUST00000168553	protein coding
Gpbp1-204	13	51.05	-6.56	2.42E-23	ENSMUST00000136471	nonsense mediated decay
Gpbp1l1-202	4	33.4	-3.23	4.55E-10	ENSMUST00000106475	protein coding
Grhl1-201	12	2.15	-1,022.58	1.83E-03	ENSMUST00000020985	protein coding
Gtf2ird1-202	5	6.4	-5.52	8.63E-07	ENSMUST00000074114	protein coding
Gtf2ird1-206	5	12.7	-3.79	3.69E-06	ENSMUST00000111244	protein coding
Gtf2ird1-212	5	1.62	-10.67	3.15E-03	ENSMUST00000200944	protein coding
Gtf2ird1-215	5	4.38	-52.64	6.87E-04	ENSMUST00000201495	nonsense mediated decay
Gtf2ird1-220	5	4.53	-48.43	7.35E-04	ENSMUST00000202104	protein coding
Gtf2ird1-231	5	7.77	-2.77	0.02	ENSMUST00000202829	protein coding
Hbp1-208	12	17.12	-8.35	4.06E-06	ENSMUST00000176520	protein coding
Hbp1-209	12	4.93	-8.18	6.56E-03	ENSMUST00000176643	protein coding
Hif1a-202	12	36.68	-2.86	6.73E-21	ENSMUST00000110461	protein coding

Hmbox1-210	14	1.8	-7.36	0.03	ENSMUST00000177326	protein coding
Homez-204	14	4.55	-6.76	0.01	ENSMUST00000176259	protein coding
Hsf1-214	15	9.36	-6.54	1.85E-06	ENSMUST00000229363	protein coding
Ikzf4-210	10	0.53	-420.03	0.01	ENSMUST00000238712	protein coding
Irf3-203	7	7.9	-654.96	3.86E-03	ENSMUST00000207128	protein coding
Irf9-205	14	4.08	-8.06	3.70E-03	ENSMUST00000138037	protein coding
Kat7-201	11	44.98	-13.79	1.17E-68	ENSMUST00000072621	protein coding
Kat7-202	11	63.81	-17	2.47E-101	ENSMUST00000092766	protein coding
Kat7-206	11	22.22	-2.3	5.76E-06	ENSMUST00000138671	protein coding
Kdm2a-212	19	10.81	-7.54	1.79E-05	ENSMUST00000176653	protein coding
Kdm2a-213	19	15.01	-4.49	2.03E-10	ENSMUST00000235335	nonsense mediated decay
Kdm2b-202	5	7.87	-4.5	1.28E-03	ENSMUST00000046073	protein coding
Kdm2b-203	5	17.84	-3.63	3.50E-09	ENSMUST00000086200	protein coding
Kdm2b-207	5	1.19	-32.93	9.17E-03	ENSMUST00000127403	nonsense mediated decay
Lcor-203	19	1.26	-76.38	1.68E-04	ENSMUST00000172371	protein coding
Lcorl-205	5	1.53	-5.73	0.03	ENSMUST00000121573	protein coding
Max-202	12	38.75	-2.6	5.06E-08	ENSMUST00000110395	protein coding
Max-205	12	8.01	-6.35	3.86E-03	ENSMUST00000218640	protein coding
Maz-204	7	118.39	-3.83	1.76E-87	ENSMUST00000206254	protein coding
Mbd3-202	10	29.56	-22.28	1.47E-29	ENSMUST00000105347	protein coding
Mbd3-206	10	76.24	-3.15	5.31E-10	ENSMUST00000142997	protein coding
Mbd4-204	6	2.95	-2.88	0.02	ENSMUST00000147282	nonsense mediated decay
Meis2-201	2	0.61	-13.04	0.01	ENSMUST00000028639	protein coding
Mga-215	2	12.52	-3.6	3.09E-09	ENSMUST00000156510	protein coding
Mterf3-214	13	1.63	-15.19	0.04	ENSMUST00000224085	nonsense mediated decay
Mterf4-203	1	2.11	-3.16	0.04	ENSMUST00000112944	protein coding

Mxi1-205	19	13.79	-3,061.90	3.50E-03	ENSMUST0000023 5880	protein coding
Mybl1-202	1	6.25	-2.3	2.46E-03	ENSMUST0000011 5468	protein coding
Myc-202	15	12.76	-2.54	0.04	ENSMUST0000015 9327	protein coding
Mynn-206	3	11.6	-2.08	3.78E-04	ENSMUST0000019 5751	nonsense mediated decay
Ncoa1-202	12	1.66	-20.8	1.58E-05	ENSMUST0000021 7674	protein coding
Nfat5-211	8	1.91	-1,473.86	7.60E-04	ENSMUST0000015 1114	protein coding
Nfat5-213	8	0.66	-631.63	0.02	ENSMUST0000015 4474	nonsense mediated decay
Nfatc3-202	8	6.58	-2	2.11E-03	ENSMUST0000021 1991	protein coding
Nfatc3-206	8	6.48	-3,216.45	1.32E-04	ENSMUST0000021 2742	protein coding
Nfe2l1-202	11	16.25	-9,755.29	8.92E-06	ENSMUST0000010 7657	protein coding
Nfe2l1-204	11	7.2	-3.73	1.08E-04	ENSMUST0000010 7659	protein coding
Nfe2l1-210	11	42.43	-17.13	2.69E-114	ENSMUST0000016 7110	protein coding
Nfe2l1-212	11	5.21	-6.33	0.01	ENSMUST0000016 9828	protein coding
Nfya-203	17	18.64	-2.14	5.88E-05	ENSMUST0000015 9063	protein coding
Nfyc-202	4	3.53	-226.46	0.03	ENSMUST0000009 7906	protein coding
Nfyc-203	4	55.25	-16,105.36	2.31E-06	ENSMUST0000011 8902	protein coding
Nme2-202	11	337.9	-4.24	1.70E-44	ENSMUST0000007 2566	protein coding
Nr3c1-202	18	0.84	-35.02	2.05E-03	ENSMUST0000009 7592	protein coding
Nr6a1-210	2	11.7	-9.41	3.55E-04	ENSMUST0000016 8098	protein coding
Nrf1-203	6	6.72	-6.54	4.77E-03	ENSMUST0000006 9831	protein coding
Nrf1-214	6	28.34	-2.47	2.86E-05	ENSMUST0000013 2866	protein coding
Nrf1-216	6	7.98	-4.12	0.03	ENSMUST0000013 3928	protein coding
Nrf1-219	6	8.21	-24.65	3.28E-06	ENSMUST0000016 7972	protein coding
Pa2g4-201	10	538.86	-9.73	4.56E-138	ENSMUST0000002 6425	protein coding
Patz1-207	11	7.93	-5.74	1.21E-04	ENSMUST0000013 4089	protein coding
Pcgf2-206	11	28.82	-2.07	2.42E-04	ENSMUST0000016 9807	protein coding

Pcgf2-207	11	3.67	-4.48	1.28E-03	ENSMUST0000017 9765	protein coding
Phf1-206	17	2.09	-9.57	4.23E-03	ENSMUST0000023 7412	protein coding
Plagl2-202	2	3.71	-2,475.53	2.54E-04	ENSMUST0000010 9795	protein coding
Pou2f1-201	1	3.91	-1,365.37	1.72E-03	ENSMUST0000002 7850	protein coding
Pou2f1-205	1	0.16	-29.78	3.05E-04	ENSMUST0000011 1427	protein coding
Pou2f1-216	1	0.98	-57.24	2.52E-03	ENSMUST0000018 4643	protein coding
Pou6f1-202	15	2.73	-15	7.10E-06	ENSMUST0000017 6140	nonsense mediated decay
Preb-204	5	14.01	-6.31	2.54E-04	ENSMUST0000020 1002	protein coding
Rara-203	11	15.47	-18.62	3.69E-27	ENSMUST0000010 7474	protein coding
Rara-207	11	7.55	-3,973.95	7.96E-05	ENSMUST0000016 4748	protein coding
Rbck1-201	2	26.39	-4.56	5.85E-14	ENSMUST0000002 8964	protein coding
Rela-204	19	1.74	-229.72	0.03	ENSMUST0000023 7838	nonsense mediated decay
Relb-209	7	1.55	-366.6	0.01	ENSMUST0000020 8087	nonsense mediated decay
Repin1-207	6	1.4	-649.81	6.08E-03	ENSMUST0000016 3452	protein coding
Rfx5-201	3	1.05	-677.22	4.79E-03	ENSMUST0000002 9772	protein coding
Rfx5-204	3	0.91	-329.33	0.02	ENSMUST0000010 7255	protein coding
Safb-210	17	6.65	-4.25	0.03	ENSMUST0000018 2951	nonsense mediated decay
Ski-202	4	27.12	-11.51	7.13E-63	ENSMUST0000008 4103	protein coding
Skil-202	3	1.1	-21.62	9.76E-03	ENSMUST0000011 7728	protein coding
Skil-203	3	25.02	-3.22	5.16E-07	ENSMUST0000011 8204	protein coding
Son-208	16	21.91	-2.05	1.58E-11	ENSMUST0000014 0312	nonsense mediated decay
Sp1-202	15	5.51	-2.78	1.70E-04	ENSMUST0000016 3709	protein coding
Sp2-202	11	11.83	-2.48	7.70E-04	ENSMUST0000010 7623	protein coding
Srcap-205	7	0.73	-522.26	8.28E-03	ENSMUST0000018 6954	protein coding
Tbp-204	17	13.67	-12.19	1.86E-05	ENSMUST0000011 9879	protein coding
Tcf12-215	9	1.09	-258.24	0.02	ENSMUST0000018 4448	protein coding

Tcf3-205	10	25.21	-1,539.19	1.16E-15	ENSMUST00000105341	protein coding
Tcf3-206	10	1.28	-152.92	0.04	ENSMUST00000105342	protein coding
Tcf3-208	10	3.93	-1,796.32	4.60E-04	ENSMUST00000105344	protein coding
Tead2-202	7	11.64	-8.23	2.87E-06	ENSMUST00000097216	protein coding
Terf2-203	8	15.23	-2.34	1.50E-05	ENSMUST00000116425	protein coding
Terf2-207	8	1.69	-5.92	0.01	ENSMUST00000142616	nonsense mediated decay
Tfap4-202	16	1.74	-216.31	0.03	ENSMUST00000229956	protein coding
Tfdp1-201	8	178.3	-2.81	5.41E-54	ENSMUST00000170909	protein coding
Tfdp2-211	9	6.15	-163.18	1.48E-06	ENSMUST00000189606	protein coding
Tfeb-204	17	1.65	-604.73	7.34E-03	ENSMUST00000113288	protein coding
Thap4-205	1	21.69	-6.63	4.11E-05	ENSMUST00000189728	protein coding
Tigd3-201	19	3.19	-3.37	0.04	ENSMUST00000055911	protein coding
Trp73-205	4	0.59	-91.41	8.89E-04	ENSMUST00000133533	protein coding
Ubp1-205	9	23.61	-4.31	9.63E-05	ENSMUST00000216558	protein coding
Usf2-209	7	39.13	-3.92	1.06E-14	ENSMUST00000170699	nonsense mediated decay
Usf3-204	16	2.58	-2.04	0.04	ENSMUST00000169582	protein coding
Wiz-204	17	9.5	-2.53	1.92E-04	ENSMUST00000163107	nonsense mediated decay
Ybx2-202	11	2.83	-96.89	5.05E-04	ENSMUST00000108601	protein coding
Zbed3-202	13	6.59	-1,996.64	4.27E-04	ENSMUST00000221807	protein coding
Zbtb21-202	16	7.23	-2.72	5.45E-06	ENSMUST00000063605	protein coding
Zbtb25-201	12	4.29	-2.85	0.05	ENSMUST00000167011	protein coding
Zbtb25-202	12	2.86	-2.78	0.01	ENSMUST00000176102	protein coding
Zbtb33-202	X	4.11	-2.77	0.03	ENSMUST00000115142	protein coding
Zbtb33-203	X	1.07	-343.02	0.01	ENSMUST00000131124	protein coding
Zbtb43-202	2	16.82	-2.85	1.06E-03	ENSMUST00000095035	protein coding
Zbtb44-202	9	12.03	-2,431.34	2.88E-04	ENSMUST00000167346	protein coding

Zbtb44-205	9	31.84	-7.51	2.03E-33	ENSMUST00000216649	protein coding
Zbtb44-206	9	40.24	-4.66	3.58E-29	ENSMUST00000217092	protein coding
Zfp110-205	7	31.83	-3.25	2.18E-12	ENSMUST00000168247	protein coding
Zfp111-203	7	3.09	-2.23	1.41E-03	ENSMUST00000086006	protein coding
Zfp131-201	13	6.04	-363.9	1.48E-12	ENSMUST00000177916	protein coding
Zfp131-206	13	23.26	-16.99	8.18E-19	ENSMUST00000223813	protein coding
Zfp142-202	1	2.04	-3.02	0.03	ENSMUST00000066986	protein coding
Zfp142-204	1	10.93	-3.02	1.45E-07	ENSMUST00000127921	nonsense mediated decay
Zfp182-201	X	2.71	-3.98	0.02	ENSMUST00000040628	protein coding
Zfp2-202	11	0.59	-260.34	0.02	ENSMUST00000109129	protein coding
Zfp207-202	11	122.68	-2.57	1.73E-25	ENSMUST00000053740	protein coding
Zfp217-202	2	34.11	-10.15	7.36E-36	ENSMUST00000109155	protein coding
Zfp219-201	14	18.53	-2.09	3.11E-03	ENSMUST00000067549	protein coding
Zfp263-206	16	4.43	-3.43	2.62E-05	ENSMUST00000161630	nonsense mediated decay
Zfp263-207	16	6.46	-2.56	0.05	ENSMUST00000162207	protein coding
Zfp280c-203	X	1.71	-1,068.34	0.01	ENSMUST00000088898	protein coding
Zfp280d-211	9	8	-11.94	4.26E-14	ENSMUST00000184036	protein coding
Zfp280d-216	9	3.53	-3.11	0.03	ENSMUST00000184399	nonsense mediated decay
Zfp354a-201	11	1.49	-392.06	0.01	ENSMUST00000020628	protein coding
Zfp354b-203	11	0.61	-14.48	0.04	ENSMUST00000164280	protein coding
Zfp384-205	6	5.93	-4.46	5.78E-03	ENSMUST00000112424	protein coding
Zfp384-206	6	10.17	-2.16	9.43E-03	ENSMUST00000112425	protein coding
Zfp410-206	12	6.26	-4.07	1.47E-03	ENSMUST00000221656	nonsense mediated decay
Zfp410-213	12	19.45	-2.3	3.43E-04	ENSMUST00000222606	nonsense mediated decay
Zfp414-205	17	4.13	-86.25	6.30E-05	ENSMUST00000170225	protein coding
Zfp428-203	7	22.66	-3.21	2.31E-09	ENSMUST00000177205	protein coding

Zfp507-206	7	2.16	-4.17	0.05	ENSMUST00000206615	protein coding
Zfp513-201	5	7.45	-2.06	0.02	ENSMUST00000031562	protein coding
Zfp516-206	18	5.78	-314.89	0.01	ENSMUST00000236858	protein coding
Zfp608-203	18	0.17	-29.68	0.02	ENSMUST00000237844	nonsense mediated decay
Zfp637-204	6	1.13	-21.66	0.01	ENSMUST00000112861	protein coding
Zfp69-202	4	2.45	-670.92	3.82E-03	ENSMUST00000106281	protein coding
Zfp777-205	6	3.21	-5.63	0.02	ENSMUST00000148362	protein coding
Zfp78-202	7	0.8	-190.73	0.03	ENSMUST00000108559	protein coding
Zfp78-205	7	0.89	-35.19	2.42E-03	ENSMUST00000208030	protein coding
Zfp942-201	17	1.07	-239.85	0.02	ENSMUST00000074295	protein coding
Zfp946-201	17	0.37	-20.4	0.02	ENSMUST00000088763	protein coding
Zfp946-204	17	0.71	-16.29	0.04	ENSMUST00000167740	protein coding
Zkscan3-207	13	10.99	-3.01	7.40E-06	ENSMUST00000223831	protein coding
Zkscan8-203	13	2.14	-5.87	0.01	ENSMUST00000156674	protein coding
Zkscan8-204	13	2.92	-4.73	1.13E-08	ENSMUST00000224362	protein coding
Zscan12-202	13	7.76	-2.72	0.04	ENSMUST00000099720	protein coding
Zscan21-201	5	13.15	-7.17	9.33E-21	ENSMUST00000062350	protein coding
Zscan21-205	5	15.93	-16.74	1.61E-23	ENSMUST00000110961	protein coding
Zscan26-201	13	16.25	-5.91	1.11E-17	ENSMUST00000032820	protein coding
Zscan29-201	2	0.66	-255.93	0.02	ENSMUST00000079024	protein coding
Zzz3-203	3	3.44	-3,999.84	7.44E-05	ENSMUST00000106101	protein coding

Table S3-3. Expression values & metadata of insignificant transcript variants of insignificant genes						
**Name**	**Chromosome**	**Max group mean**	**Fold change**	**FDR p-value**	**ENSEMBL**	**Biotype**
1700057G04Rik-204	9	0.06	7.56	NaN	ENSMUST00000135182	nonsense mediated decay
Adnp-201	2	13.16	1	1	ENSMUST00000057793	protein coding
Adnp2-201	18	34.33	-1.38	9.86E-06	ENSMUST00000066743	protein coding
Aebp2-201	6	13.57	-1.83	2.16E-05	ENSMUST00000032359	protein coding
Aebp2-203	6	0.37	3.13	0.09	ENSMUST00000095350	protein coding
Aebp2-206	6	0.27	1.06	0.98	ENSMUST00000161335	protein coding
Aebp2-208	6	1.85	1.74	0.38	ENSMUST00000162903	protein coding
Aebp2-210	6	15.9	-1.37	0.14	ENSMUST00000249348	protein coding
Aebp2-211	6	0.26	2.36	0.61	ENSMUST00000249349	protein coding
Aebp2-212	6	1.2	-2.25	0.15	ENSMUST00000249350	nonsense mediated decay
Ahctf1-201	1	110.03	-1.58	1.29E-09	ENSMUST00000027768	protein coding
Ahctf1-204	1	0.3	1.03	0.99	ENSMUST00000140489	protein coding
Ahctf1-205	1	0.15	-13.01	0.38	ENSMUST00000145968	protein coding
Ahr-201	12	0.3	-53.4	0.13	ENSMUST00000110811	nonsense mediated decay
Ahr-202	12	1.06	-1.44	0.53	ENSMUST00000116436	protein coding
Ahrr-201	13	0.44	1.85	0.41	ENSMUST00000022059	protein coding
Ahrr-202	13	0.03	7.89	NaN	ENSMUST00000109640	protein coding
Aire-202	10	0.12	-1.06	0.98	ENSMUST00000105395	protein coding
Aire-204	10	0.17	42.62	0.11	ENSMUST00000128241	protein coding
Aire-205	10	0.31	6.36	0.2	ENSMUST00000130972	protein coding
Aire-206	10	4.93	-1.81	0.03	ENSMUST00000131028	nonsense mediated decay
Aire-207	10	0.27	1.15	0.95	ENSMUST00000140636	protein coding
Aire-213	10	2.36	-5.86	0.1	ENSMUST00000148469	protein coding
Aire-214	10	0.37	4.35	0.45	ENSMUST00000154374	protein coding

Aire-215	10	0.11	-7.22	0.3	ENSMUST00000155021	protein coding
Akap8-201	17	43.68	-1.24	4.91E-03	ENSMUST00000002699	protein coding
Akap8-203	17	0.8	-7.49	0.14	ENSMUST00000235360	protein coding
Akap8-204	17	1.87	1.56	0.74	ENSMUST00000235702	protein coding
Akap8-208	17	0.17	10.85	NaN	ENSMUST00000236580	protein coding
Akap8-211	17	54.96	-1.95	2.33E-08	ENSMUST00000236954	nonsense mediated decay
Akap8-213	17	10.29	-1.31	0.33	ENSMUST00000237381	nonsense mediated decay
Akap8-215	17	1.27	-1.77	0.56	ENSMUST00000237860	protein coding
Akap8l-201	17	44.25	1.47	5.56E-03	ENSMUST00000050214	protein coding
Akap8l-202	17	0.26	-2.14	0.7	ENSMUST00000235265	protein coding
Akap8l-209	17	12.55	1.19	0.5	ENSMUST00000236503	protein coding
Akap8l-211	17	0.53	-2.02	0.7	ENSMUST00000236765	protein coding
Akap8l-213	17	1.64	-1.94	0.45	ENSMUST00000237936	nonsense mediated decay
Akna-201	4	7	1.14	0.6	ENSMUST00000035724	protein coding
Alx3-201	3	0.07	-4.79	0.39	ENSMUST00000014747	protein coding
Alx3-202	3	0.07	-7.65	NaN	ENSMUST00000233202	protein coding
Arhgap35-201	7	37.78	-1.25	1.24E-03	ENSMUST00000075845	protein coding
Arid2-201	15	40.07	1.3	9.89E-05	ENSMUST00000096250	protein coding
Arid5b-201	10	0.06	36.5	0.13	ENSMUST00000020106	nonsense mediated decay
Arid5b-203	10	12.71	-1.56	0.07	ENSMUST00000218532	protein coding
Arid5b-204	10	40.7	1.48	9.15E-08	ENSMUST00000219238	protein coding
Arnt-201	3	28.16	-1.46	3.90E-03	ENSMUST00000090804	protein coding
Arnt-202	3	31.16	1.34	0.04	ENSMUST00000102749	protein coding
Arnt-204	3	5.2	-1.15	0.75	ENSMUST00000107161	protein coding
Arnt-205	3	0.9	-1.67	0.67	ENSMUST00000136413	protein coding
Arnt2-204	7	0.06	7.56	NaN	ENSMUST00000207769	protein coding

Arnt2-206	7	0.09	7.89	NaN	ENSMUST00000208204	nonsense mediated decay
Arx-201	X	0.02	-9.72	0.42	ENSMUST00000046565	protein coding
Arx-202	X	0.02	-2.29	0.67	ENSMUST00000113947	protein coding
Ascl1-201	10	0.02	1.01	1	ENSMUST00000020243	protein coding
Ascl3-201	7	0.15	14.15	0.24	ENSMUST00000035372	protein coding
Ash1l-201	3	8.2	-1.56	3.45E-04	ENSMUST00000090933	protein coding
Atf2-201	2	2.75	1.85	0.33	ENSMUST00000055833	protein coding
Atf2-204	2	5.35	1.2	0.54	ENSMUST00000112007	protein coding
Atf2-205	2	17.06	-1.32	0.15	ENSMUST00000112010	protein coding
Atf2-207	2	22.56	-1.8	0.02	ENSMUST00000112017	protein coding
Atf2-208	2	2.65	-2.43	0.05	ENSMUST00000124737	protein coding
Atf2-210	2	2	-7.41	0.05	ENSMUST00000128531	protein coding
Atf2-216	2	0.07	7.56	NaN	ENSMUST00000154456	protein coding
Atf3-201	1	10.21	1.99	0.02	ENSMUST00000027941	protein coding
Atf6b-201	17	36.21	1.29	0.1	ENSMUST00000015605	protein coding
Atf6b-202	17	0.29	-108.42	0.09	ENSMUST00000173984	protein coding
Atf6b-204	17	0.28	-1.58	0.82	ENSMUST00000174519	nonsense mediated decay
Atf6b-205	17	0.24	-15.47	0.34	ENSMUST00000174614	nonsense mediated decay
Atf7-202	15	0.29	2.19	0.55	ENSMUST00000108828	protein coding
Atf7-203	15	0.04	13.58	0.28	ENSMUST00000169033	protein coding
Atf7-204	15	0.44	37.43	0.1	ENSMUST00000183452	nonsense mediated decay
Atf7-206	15	0.11	-13.01	0.38	ENSMUST00000183765	protein coding
Atf7-208	15	0.29	20.74	0.18	ENSMUST00000184077	protein coding
Atf7-209	15	2.74	2.19	0.16	ENSMUST00000184485	protein coding
Atf7-210	15	16.1	1.85	1.21E-09	ENSMUST00000184616	protein coding
Atf7-211	15	0.61	-3.22	0.44	ENSMUST00000184906	protein coding

Atmin-201	8	46.37	1.18	0.03	ENSMUST00000109099	protein coding
Atoh1-201	6	0.03	2.37	NaN	ENSMUST00000101351	protein coding
Atoh7-201	10	0.1	4.48	0.31	ENSMUST00000044059	protein coding
Bach1-201	16	27.65	1.86	3.50E-09	ENSMUST00000026703	protein coding
Bach2-201	4	0.57	-2.05	0.4	ENSMUST00000037416	protein coding
Barx2-201	9	0.24	1.23	0.9	ENSMUST00000116615	protein coding
Batf2-207	19	0.04	-9.72	0.42	ENSMUST00000237511	protein coding
Baz2a-201	10	83.72	-1.53	1.79E-07	ENSMUST00000045621	protein coding
Baz2a-202	10	35.94	-1	0.99	ENSMUST00000170054	protein coding
Baz2a-205	10	3.78	-79.64	0.08	ENSMUST00000219072	protein coding
Bbx-202	16	0.52	-1.5	0.82	ENSMUST00000089399	protein coding
Bbx-203	16	2.38	1.59	0.4	ENSMUST00000089404	protein coding
Bbx-208	16	25.14	1.86	1.15E-14	ENSMUST00000138166	protein coding
BC025920-201	10	1.65	1.42	0.66	ENSMUST00000099442	protein coding
BC025920-202	10	0.29	1.35	0.85	ENSMUST00000119492	protein coding
BC025920-203	10	0.23	-1.26	0.9	ENSMUST00000119753	protein coding
BC025920-204	10	0.19	42.3	0.11	ENSMUST00000121138	protein coding
Bcl6b-201	11	10.71	-1.23	0.34	ENSMUST00000000326	protein coding
Bhlha15-201	5	2.65	1.8	0.21	ENSMUST00000060747	protein coding
Bhlhe22-201	3	0.06	-1.65	0.75	ENSMUST00000026120	protein coding
Bhlhe40-201	6	57.55	1.37	1.32E-03	ENSMUST00000032194	protein coding
Bhlhe40-202	6	0.2	13.58	0.27	ENSMUST00000163617	protein coding
Bmal1-201	7	10.02	1.19	0.56	ENSMUST00000047321	protein coding
Bmal1-204	7	0.13	-57.77	0.14	ENSMUST00000210238	protein coding
Bptf-201	11	49.01	1.23	4.11E-03	ENSMUST00000057892	protein coding
Bptf-204	11	1.52	1.72	0.46	ENSMUST00000133317	protein coding

Brf2-201	8	20.9	1.01	0.96	ENSMUST00000033877	protein coding
Brf2-202	8	0.25	14.15	0.25	ENSMUST00000209770	nonsense mediated decay
Brf2-204	8	1.56	1.94	0.42	ENSMUST00000210552	nonsense mediated decay
Brf2-206	8	1.52	-2.35	0.38	ENSMUST00000211151	nonsense mediated decay
Brf2-207	8	0.7	-3.62	0.32	ENSMUST00000211236	protein coding
Bsx-201	9	0.03	-13.87	0.36	ENSMUST00000067375	protein coding
Camta1-204	4	6.96	1.12	0.71	ENSMUST00000105670	protein coding
Camta1-205	4	0.08	-5.59	NaN	ENSMUST00000131948	protein coding
Camta1-207	4	3.94	-1.55	0.24	ENSMUST00000140030	protein coding
Camta1-209	4	9.34	-1.44	0.05	ENSMUST00000153938	nonsense mediated decay
Camta2-201	11	0.06	37.53	0.13	ENSMUST00000036299	protein coding
Camta2-202	11	0.39	-1.04	1	ENSMUST00000100933	protein coding
Camta2-204	11	2.48	4.02	0.06	ENSMUST00000108545	protein coding
Camta2-208	11	0.75	1.43	0.8	ENSMUST00000145823	nonsense mediated decay
Carf-202	1	1.9	-3.14	0.34	ENSMUST00000124986	protein coding
Carf-203	1	0.85	1.3	0.78	ENSMUST00000130075	nonsense mediated decay
Carf-204	1	0.79	-1.51	0.78	ENSMUST00000132949	nonsense mediated decay
Carf-206	1	0.09	-30.57	0.24	ENSMUST00000180952	protein coding
Carf-207	1	0.2	-57.77	0.14	ENSMUST00000186107	nonsense mediated decay
Carf-209	1	1.91	-1.06	0.91	ENSMUST00000187978	protein coding
Cdc5lrt5-201	10	0.01	-5.57	NaN	ENSMUST00000177762	protein coding
Cdc5lrt6-201	10	0.05	-19.18	0.29	ENSMUST00000179851	protein coding
Cdc5lrt9-201	10	0.13	38.32	0.12	ENSMUST00000177744	protein coding
Cdx4-201	X	0.07	-1.46	0.83	ENSMUST00000033689	protein coding
Cebpe-201	14	0.11	1.47	0.83	ENSMUST00000064290	protein coding
Cebpg-201	7	3.66	2.39	0.28	ENSMUST00000070191	protein coding

Cebpg-202	7	16.62	-1.32	0.03	ENSMUST00000130491	protein coding
Cebpz-201	17	55.22	1.01	0.94	ENSMUST00000024885	protein coding
Cebpz-204	17	70.6	-1.95	3.63E-19	ENSMUST00000233484	nonsense mediated decay
Cenpa-202	5	116.94	1.74	4.99E-06	ENSMUST00000133316	nonsense mediated decay
Cenpa-205	5	56.12	1.94	1.12E-04	ENSMUST00000144742	protein coding
Cenpa-206	5	0.99	2.12	0.59	ENSMUST00000149759	protein coding
Cenpb-201	2	31.93	1.31	0.03	ENSMUST00000089510	protein coding
Cenps-201	4	23.89	-1.31	0.31	ENSMUST00000030813	protein coding
Cenps-202	4	1.2	3.06	0.35	ENSMUST00000105695	protein coding
Cenps-203	4	0.62	-1.51	0.81	ENSMUST00000105696	nonsense mediated decay
Cenps-207	4	5.72	-1.59	0.55	ENSMUST00000177408	protein coding
Cenpt-201	8	59.32	1.04	0.84	ENSMUST00000040776	protein coding
Cenpt-204	8	4.7	-2.11	0.11	ENSMUST00000212431	nonsense mediated decay
Cenpt-205	8	0.39	-1.06	0.98	ENSMUST00000212552	nonsense mediated decay
Cenpt-209	8	0.44	-1.1	0.96	ENSMUST00000212839	nonsense mediated decay
Champ1-201	8	41.13	1.03	0.82	ENSMUST00000051870	protein coding
Chchd3-201	6	119.58	-1.54	3.16E-09	ENSMUST00000066379	protein coding
Chchd3-203	6	0.11	24.03	0.16	ENSMUST00000124436	nonsense mediated decay
Chchd3-205	6	0.89	-102.85	0.07	ENSMUST00000127666	protein coding
Cic-201	7	7.78	-1.34	0.36	ENSMUST00000005578	protein coding
Cic-202	7	16.78	-1.09	0.64	ENSMUST00000163320	protein coding
Cic-207	7	5.17	-1.53	0.2	ENSMUST00000165239	protein coding
Clock-201	5	6.59	-1.4	0.12	ENSMUST00000075159	protein coding
Clock-204	5	1.56	-2.51	0.06	ENSMUST00000202122	protein coding
Clock-205	5	1.56	-1.66	0.11	ENSMUST00000202651	protein coding
Creb1-201	1	5.59	-1.11	0.73	ENSMUST00000049932	protein coding

Creb1-202	1	8.4	-1.24	0.18	ENSMUST00000087366	protein coding
Creb1-203	1	2.14	-1.66	0.51	ENSMUST00000171164	protein coding
Creb1-207	1	0.59	2.97	0.52	ENSMUST00000187811	protein coding
Creb1-210	1	1.54	-2.01	0.41	ENSMUST00000190876	nonsense mediated decay
Creb3l1-201	2	7.17	-1.34	0.15	ENSMUST00000028663	protein coding
Creb5-201	6	0.22	1.01	1	ENSMUST00000047450	protein coding
Creb5-212	6	0.49	-2.15	0.27	ENSMUST00000205120	protein coding
Crem-202	18	0.15	-23.27	0.26	ENSMUST00000049942	protein coding
Crem-206	18	0.23	10.65	0.15	ENSMUST00000124747	protein coding
Crem-207	18	0.21	-30.57	0.24	ENSMUST00000126578	protein coding
Crem-211	18	0.03	7.56	NaN	ENSMUST00000130455	protein coding
Crem-212	18	1.12	-17.6	0.06	ENSMUST00000130599	protein coding
Crem-213	18	0.54	-2.33	0.52	ENSMUST00000131899	protein coding
Crem-215	18	0.45	2.67	0.5	ENSMUST00000134027	nonsense mediated decay
Crem-216	18	0.08	-10.55	0.41	ENSMUST00000136961	protein coding
Crem-217	18	1.5	1.04	0.98	ENSMUST00000137568	protein coding
Crem-218	18	0.34	11.17	0.16	ENSMUST00000139537	protein coding
Crem-219	18	0.13	19.13	0.21	ENSMUST00000140332	protein coding
Crem-221	18	3.13	-1.58	0.53	ENSMUST00000142690	protein coding
Crem-222	18	0.23	1.28	0.9	ENSMUST00000144496	protein coding
Crem-223	18	0.26	-35.07	0.22	ENSMUST00000146265	protein coding
Crem-225	18	0.23	20.74	0.18	ENSMUST00000148305	nonsense mediated decay
Crem-228	18	0.34	3.98	0.38	ENSMUST00000150235	protein coding
Crem-230	18	0.07	25.54	0.17	ENSMUST00000151311	protein coding
Crem-232	18	0.1	-2.86	0.56	ENSMUST00000152900	protein coding
Crem-233	18	1.55	1.11	0.9	ENSMUST00000154135	protein coding

Crem-236	18	0.38	-1.39	0.84	ENSMUST00000154715	protein coding
Crem-239	18	0.1	34.03	0.13	ENSMUST00000165086	protein coding
Csrnp1-201	9	32.79	1.32	0.05	ENSMUST00000035101	protein coding
Csrnp1-202	9	13.7	-1.22	0.71	ENSMUST00000213936	protein coding
Csrnp1-203	9	0.69	1.76	0.7	ENSMUST00000214058	protein coding
Csrnp2-201	15	10.36	-1.53	0.04	ENSMUST00000061457	protein coding
Ctcf-201	8	96.5	-1.63	1.95E-14	ENSMUST00000005841	protein coding
Cux2-201	5	0.47	2.03	0.52	ENSMUST00000086317	protein coding
Cux2-202	5	0.48	2.08	0.29	ENSMUST00000111752	protein coding
Cux2-205	5	0.18	-12.38	0.37	ENSMUST00000134326	protein coding
Cux2-207	5	0.03	4.26	NaN	ENSMUST00000154139	protein coding
Cux2-210	5	2.83	2.34	0.33	ENSMUST00000168288	protein coding
Cxxc1-201	18	33.74	-1.08	0.64	ENSMUST00000025444	protein coding
Cxxc4-202	3	0.05	-2.69	0.45	ENSMUST00000181904	protein coding
Cxxc5-201	18	27.59	1.13	0.58	ENSMUST00000060722	protein coding
Cxxc5-202	18	0.74	-73.91	0.1	ENSMUST00000235161	protein coding
Cxxc5-203	18	4.6	3.08	0.14	ENSMUST00000235169	protein coding
Cxxc5-205	18	0.25	1.5	0.82	ENSMUST00000235404	protein coding
Dach1-201	14	0.06	-1.25	0.88	ENSMUST00000069334	protein coding
Dach1-202	14	0.02	16.57	0.23	ENSMUST00000071533	protein coding
Dbp-201	7	2.53	1.93	0.26	ENSMUST00000080885	protein coding
Dbp-203	7	0.17	-35.07	0.22	ENSMUST00000211357	protein coding
Dbp-204	7	2.36	-1.46	0.65	ENSMUST00000211513	protein coding
Dbx1-201	7	0.03	2.41	NaN	ENSMUST00000032717	protein coding
Ddit3-201	10	16.14	-1.68	0.08	ENSMUST00000026475	protein coding
Ddit3-203	10	0.87	-1.52	0.81	ENSMUST00000139091	protein coding

Deaf1-201	7	18.63	1.69	0.03	ENSMUST00000080553	protein coding
Deaf1-203	7	5.78	-2.49	0.11	ENSMUST00000209600	protein coding
Deaf1-204	7	7.54	-1.04	0.95	ENSMUST00000209608	nonsense mediated decay
Deaf1-205	7	4.32	-1.26	0.68	ENSMUST00000210062	nonsense mediated decay
Deaf1-206	7	0.38	-5.71	0.38	ENSMUST00000210816	nonsense mediated decay
Deaf1-207	7	2.33	2.4	0.14	ENSMUST00000210830	nonsense mediated decay
Deaf1-208	7	1.95	-7.57	0.07	ENSMUST00000211146	nonsense mediated decay
Deaf1-209	7	2.85	1.9	0.15	ENSMUST00000211537	protein coding
Dmrta1-201	4	0.08	2.1	0.62	ENSMUST00000052478	protein coding
Dmrtb1-201	4	1.04	-3.12	0.12	ENSMUST00000069271	protein coding
Dmrtb1-203	4	2.09	-1.36	0.71	ENSMUST00000131776	protein coding
Dmtf1-201	5	9.67	-1.04	0.92	ENSMUST00000071921	protein coding
Dmtf1-202	5	27.05	-1.48	3.87E-03	ENSMUST00000095017	protein coding
Dmtf1-204	5	1.63	1.32	0.68	ENSMUST00000183448	nonsense mediated decay
Dmtf1-205	5	0.06	7.56	NaN	ENSMUST00000183525	protein coding
Dmtf1-209	5	1.16	1.01	1	ENSMUST00000184159	nonsense mediated decay
Dmtf1-212	5	0.23	-1.17	0.9	ENSMUST00000184401	nonsense mediated decay
Dmtf1-213	5	2.29	-1.83	0.27	ENSMUST00000184620	nonsense mediated decay
Dmtf1-214	5	0.31	3.58	0.46	ENSMUST00000184888	nonsense mediated decay
Dmtf1-217	5	1.1	-9.47	0.18	ENSMUST00000185100	protein coding
Dnmt1-201	9	102.56	1.83	5.85E-14	ENSMUST00000004202	protein coding
Dnmt1-202	9	73.38	-1.76	1.01E-17	ENSMUST00000177754	protein coding
Dot1l-201	10	41.67	1.39	1.82E-05	ENSMUST00000105336	protein coding
Dot1l-202	10	0.67	1.43	0.65	ENSMUST00000127740	nonsense mediated decay
Dot1l-203	10	6.28	2.11	0.14	ENSMUST00000138505	protein coding
Dot1l-205	10	1.19	5.36	0.08	ENSMUST00000149394	nonsense mediated decay

Dot1l-206	10	3.12	-2.3	0.09	ENSMUST00000150338	protein coding
Dr1-201	5	50.93	1.24	0.07	ENSMUST00000031190	protein coding
Drap1-203	19	6.2	-1.73	0.39	ENSMUST00000113674	protein coding
Drap1-210	19	7.47	-1.07	0.92	ENSMUST00000148219	protein coding
Drgx-202	14	0.04	-13.87	0.36	ENSMUST00000186452	protein coding
Drgx-204	14	0.02	-10.55	0.41	ENSMUST00000189022	protein coding
Drgx-205	14	0.04	-5.57	NaN	ENSMUST00000228878	protein coding
E2f1-201	2	17.98	-1.87	2.98E-03	ENSMUST00000000894	protein coding
E2f1-202	2	26.85	-1.05	0.82	ENSMUST00000103145	protein coding
E2f3-204	13	7.16	-1.44	0.19	ENSMUST00000222730	protein coding
E2f4-201	8	180.98	-1.52	1.44E-09	ENSMUST00000015003	protein coding
E2f5-201	3	22.76	-1.49	0.07	ENSMUST00000029069	protein coding
E2f6-204	12	18.21	-1.88	3.64E-03	ENSMUST00000221541	nonsense mediated decay
E2f6-206	12	0.33	2.23	0.64	ENSMUST00000221934	nonsense mediated decay
E2f7-201	10	13.79	1.25	0.28	ENSMUST00000073781	protein coding
E2f7-204	10	3.2	-1.23	0.84	ENSMUST00000173471	protein coding
E2f7-205	10	0.56	2.16	0.53	ENSMUST00000173634	protein coding
E2f7-206	10	0.17	-1.28	0.91	ENSMUST00000173948	nonsense mediated decay
E2f7-209	10	0.78	1.25	0.82	ENSMUST00000174857	protein coding
E2f8-201	7	18.78	-1.17	0.42	ENSMUST00000058745	protein coding
E2f8-202	7	10.65	1.08	0.83	ENSMUST00000119223	protein coding
E430018J23Rik-201	7	1.71	1.18	0.82	ENSMUST00000074249	protein coding
E430018J23Rik-202	7	0.08	7.56	NaN	ENSMUST00000106303	protein coding
E430018J23Rik-203	7	0.74	1.05	0.96	ENSMUST00000165495	protein coding
E4f1-201	17	4.2	-1	1	ENSMUST00000056032	protein coding
E4f1-208	17	5.29	1.15	0.82	ENSMUST00000228882	protein coding

Eea1-201	10	6.96	1.82	3.18E-03	ENSMUST00000053484	protein coding
Eea1-203	10	0.43	1.28	0.82	ENSMUST00000218291	nonsense mediated decay
Eea1-204	10	0.02	4.26	NaN	ENSMUST00000218517	nonsense mediated decay
Egr3-201	14	0.03	-7.84	NaN	ENSMUST00000035908	protein coding
Egr3-204	14	0.13	2.09	0.56	ENSMUST00000225200	protein coding
Elf3-202	1	34.26	1.27	0.38	ENSMUST00000185752	protein coding
Elk1-201	X	15.79	-1.33	0.2	ENSMUST00000009550	protein coding
Elk3-201	10	5.99	1.79	1.33E-03	ENSMUST00000008542	protein coding
Elk3-202	10	2.79	1.24	0.79	ENSMUST00000129827	nonsense mediated decay
Elk4-201	1	1.72	-1.68	0.52	ENSMUST00000027696	protein coding
Elk4-202	1	7.57	1.1	0.75	ENSMUST00000086556	protein coding
Elk4-206	1	0.74	-2.06	0.42	ENSMUST00000146432	protein coding
Elk4-207	1	7.76	-1.07	0.84	ENSMUST00000147218	nonsense mediated decay
En2-201	5	1.63	1	1	ENSMUST00000036177	protein coding
Erf-201	7	77.18	-1.45	3.44E-05	ENSMUST00000045847	protein coding
Esr1-201	10	0.01	-1.84	0.78	ENSMUST00000067086	protein coding
Esr1-203	10	0.01	-1.26	NaN	ENSMUST00000105589	protein coding
Esr1-204	10	0.02	20.81	0.18	ENSMUST00000105590	protein coding
Esrrb-202	12	2.1	-1.36	0.65	ENSMUST00000110203	protein coding
Esrrb-203	12	281.8	-1.93	1.61E-20	ENSMUST00000110204	protein coding
Esrrb-204	12	2.97	-3.42	0.11	ENSMUST00000116402	protein coding
Etv2-201	7	1.56	1.57	0.62	ENSMUST00000108147	protein coding
Etv3-201	3	0.8	1.11	0.91	ENSMUST00000117293	protein coding
Etv3-202	3	2.39	2.33	0.14	ENSMUST00000119109	protein coding
Etv3-205	3	13.37	1.14	0.57	ENSMUST00000170036	protein coding
Etv3l-201	3	0.03	7.89	NaN	ENSMUST00000238853	protein coding

Etv3l-202	3	0.02	-1.26	NaN	ENSMUST00000238894	protein coding
Etv4-201	11	94.64	-1.86	2.83E-15	ENSMUST00000017868	protein coding
Etv4-202	11	43.95	-1.41	0.01	ENSMUST00000107176	protein coding
Etv4-212	11	4.89	-1.94	0.25	ENSMUST00000164750	protein coding
Evx1-201	6	0.22	-1.11	0.94	ENSMUST00000031787	protein coding
Evx1-202	6	0.07	7.89	NaN	ENSMUST00000129243	protein coding
Fev-201	1	0.21	1.63	0.75	ENSMUST00000068631	protein coding
Fev-202	1	0.08	10.85	NaN	ENSMUST00000159232	protein coding
Fezf2-201	14	6.35E-03	-3.31	NaN	ENSMUST00000022262	protein coding
Fezf2-202	14	0.01	4.26	NaN	ENSMUST00000224023	protein coding
Figla-201	6	0.35	-42.08	0.17	ENSMUST00000032070	protein coding
Flywch1-201	17	1.62	-1.4	0.64	ENSMUST00000045517	protein coding
Flywch1-203	17	7.34	1.33	0.73	ENSMUST00000226460	protein coding
Flywch1-204	17	17.69	1.01	0.97	ENSMUST00000227120	protein coding
Foxa3-201	7	1.68	-1	1	ENSMUST00000036018	protein coding
Foxb1-201	9	0.01	4.26	NaN	ENSMUST00000071281	protein coding
Foxb2-201	19	0.08	17.44	0.2	ENSMUST00000072915	protein coding
Foxd1-201	13	0.12	1.81	0.68	ENSMUST00000105098	protein coding
Foxe3-201	4	0.05	7.56	NaN	ENSMUST00000050940	protein coding
Foxh1-201	15	38.73	-1.14	0.6	ENSMUST00000037824	protein coding
Foxj3-201	4	12.94	-1.36	0.12	ENSMUST00000044564	protein coding
Foxj3-202	4	13.63	1.24	0.36	ENSMUST00000106310	protein coding
Foxj3-205	4	0.12	-2.29	0.66	ENSMUST00000137560	protein coding
Foxj3-206	4	0.03	7.56	NaN	ENSMUST00000138845	protein coding
Foxj3-207	4	0.09	4.26	NaN	ENSMUST00000160219	protein coding
Foxj3-209	4	0.06	-1.36	0.87	ENSMUST00000162267	protein coding

Foxj3-210	4	0.08	-5.57	NaN	ENSMUST00000176012	protein coding
Foxk2-201	11	35.13	-1.34	3.67E-05	ENSMUST00000106113	protein coding
Foxm1-201	6	37.42	1.22	0.03	ENSMUST00000073316	protein coding
Foxm1-202	6	4.27	-1.05	0.95	ENSMUST00000112148	protein coding
Foxm1-207	6	6.29	-1.94	0.17	ENSMUST00000203040	protein coding
Foxm1-208	6	0.31	-4.1	0.42	ENSMUST00000203258	protein coding
Foxn1-201	11	0.05	2.75	0.53	ENSMUST00000108294	protein coding
Foxn3-205	12	0.47	-1.16	0.93	ENSMUST00000176928	protein coding
Foxn3-212	12	2.46	1.49	0.72	ENSMUST00000222458	nonsense mediated decay
Foxn3-214	12	0.07	-1.26	NaN	ENSMUST00000223484	protein coding
Foxo1-201	3	18.71	1.6	4.67E-06	ENSMUST00000053764	protein coding
Foxo3-201	10	0.17	64.59	0.06	ENSMUST00000056974	protein coding
Foxo3-203	10	31.74	-1.06	0.45	ENSMUST00000105502	protein coding
Foxo3-204	10	0.51	3.21	0.27	ENSMUST00000175881	protein coding
Foxo6-201	4	0.98	1.03	0.98	ENSMUST00000102656	protein coding
Foxp2-201	6	0.01	-10.55	0.41	ENSMUST00000031545	protein coding
Foxp2-205	6	0.01	-5.57	NaN	ENSMUST00000115474	protein coding
Foxp2-207	6	3.44E-03	4.26	NaN	ENSMUST00000115477	protein coding
Foxp2-216	6	0.08	-5.57	NaN	ENSMUST00000154448	protein coding
Foxp3-201	X	0.05	-2.06	0.72	ENSMUST00000045566	protein coding
Foxp3-202	X	0.04	-1.41	0.87	ENSMUST00000115738	protein coding
Foxp3-203	X	5.96E-03	4.26	NaN	ENSMUST00000115739	protein coding
Foxp3-206	X	0.01	-5.57	NaN	ENSMUST00000234363	protein coding
Foxp3-209	X	0.05	1.34	NaN	ENSMUST00000234896	protein coding
Foxp4-201	17	12.56	1.08	0.83	ENSMUST00000097311	protein coding
Foxp4-202	17	21.64	2.07	0.21	ENSMUST00000113262	protein coding

Foxp4-203	17	0.36	-1.89	0.73	ENSMUST000001 13263	protein coding
Foxp4-204	17	18.46	-1.65	3.81E-03	ENSMUST000001 13265	protein coding
Foxp4-208	17	1	15.83	0.08	ENSMUST000001 54108	protein coding
Foxq1-201	13	1.45	1.34	0.53	ENSMUST000000 42118	protein coding
Foxr1-201	9	1.31	-1.97	0.38	ENSMUST000000 98837	protein coding
Foxr1-202	9	0.09	-1.29	0.9	ENSMUST000002 15375	protein coding
Foxr1-203	9	1.3	1.05	0.96	ENSMUST000002 15980	protein coding
Foxs1-201	2	0.03	-1.77	0.78	ENSMUST000000 99200	protein coding
Gata4-204	14	1.16	-118.26	0.06	ENSMUST000001 32122	protein coding
Gata4-206	14	1.03	-95.42	0.09	ENSMUST000001 56782	protein coding
Gata5-201	2	0.04	5.48	0.3	ENSMUST000000 15771	protein coding
Gatad2a-201	8	19.77	1.91	5.77E-03	ENSMUST000000 65169	protein coding
Gatad2a-202	8	94.83	-1.13	0.19	ENSMUST000001 16463	protein coding
Gatad2a-203	8	8.95	-1.43	0.11	ENSMUST000001 77851	protein coding
Gatad2a-204	8	0.26	-1.51	0.83	ENSMUST000002 11960	protein coding
Gatad2a-205	8	1.19	-5.21	0.26	ENSMUST000002 12277	protein coding
Gatad2b-203	3	0.36	-1.74	0.73	ENSMUST000001 97988	protein coding
Gatad2b-205	3	24.94	-1.41	7.53E-06	ENSMUST000001 99754	protein coding
Gatad2b-208	3	1.45	1.09	0.94	ENSMUST000002 06907	protein coding
Gcm2-201	13	0.08	-38.9	0.18	ENSMUST000000 21791	protein coding
Gcm2-202	13	0.04	-5.57	NaN	ENSMUST000002 25271	protein coding
Gfi1-202	5	0.08	1.88	0.68	ENSMUST000000 65478	protein coding
Gfi1-204	5	0.07	7.56	NaN	ENSMUST000001 59263	protein coding
Gli3-201	13	2.6	-1.01	0.98	ENSMUST000001 10510	protein coding
Gli3-202	13	0.17	-7.3	0.25	ENSMUST000001 30065	protein coding
Glis2-201	16	49.82	1.97	7.31E-10	ENSMUST000000 14447	protein coding

Glmp-201	3	24.2	-1.12	0.66	ENSMUST00000001454	protein coding
Glmp-202	3	0.58	-1.26	0.89	ENSMUST00000131666	nonsense mediated decay
Glmp-206	3	34.56	1.22	0.57	ENSMUST00000154381	protein coding
Glmp-207	3	66.74	1.93	7.89E-04	ENSMUST00000176425	protein coding
Glmp-208	3	1.98	-139.17	0.06	ENSMUST00000176519	protein coding
Glyr1-201	16	52.42	-1.41	9.29E-05	ENSMUST00000023189	protein coding
Glyr1-202	16	74.88	-1.38	1.19E-05	ENSMUST00000115844	protein coding
Gm20422-201	8	0.55	-3.28	0.38	ENSMUST00000126915	protein coding
Gm20422-202	8	0.02	4.26	NaN	ENSMUST00000149782	protein coding
Gm28041-201	13	0.25	2.55	0.56	ENSMUST00000166080	nonsense mediated decay
Gm28044-201	13	0.62	2.83	0.47	ENSMUST00000044819	nonsense mediated decay
Gm28230-201	2	0.04	2.79	0.55	ENSMUST00000053932	nonsense mediated decay
Gm44505-201	19	2.09	1.2	0.81	ENSMUST00000142247	nonsense mediated decay
Gm44973-201	7	0.3	-3.54	0.45	ENSMUST00000207050	protein coding
Gm50253-201	17	3.07	-1.52	0.24	ENSMUST00000133257	nonsense mediated decay
Gm7072-201	17	9.72	1.11	0.73	ENSMUST00000233465	protein coding
Gmeb1-202	4	14.63	-1.17	0.57	ENSMUST00000105964	protein coding
Gmeb2-201	2	17.16	1.07	0.78	ENSMUST00000049032	protein coding
Gpbp1-201	13	42.95	1.22	0.1	ENSMUST00000047627	protein coding
Gpbp1-202	13	57.69	1.61	1.38E-06	ENSMUST00000091236	protein coding
Gpbp1l1-201	4	38.2	-1.32	0.01	ENSMUST00000030460	protein coding
Grhl3-201	4	22.95	-1.03	0.9	ENSMUST00000105855	protein coding
Gsc2-201	16	0.47	-1.14	0.92	ENSMUST00000012279	protein coding
Gsx2-201	5	0.04	-10.18	0.42	ENSMUST00000040477	protein coding
Gsx2-202	5	0.06	-11.79	0.39	ENSMUST00000160104	protein coding
Gtf2b-201	3	61.47	-1.25	0.08	ENSMUST00000029938	protein coding

Gtf2ird1-207	5	1.74	-1.58	0.67	ENSMUST00000111245	protein coding
Gtf2ird1-208	5	4.8	1.73	0.44	ENSMUST00000167084	protein coding
Gtf2ird1-210	5	0.69	-3	0.44	ENSMUST00000200798	nonsense mediated decay
Gtf2ird1-213	5	0.88	-10.7	0.16	ENSMUST00000201441	protein coding
Gtf2ird1-214	5	1.02	-92.68	0.1	ENSMUST00000201447	protein coding
Gtf2ird1-216	5	0.28	-27.72	0.25	ENSMUST00000201526	nonsense mediated decay
Gtf2ird1-221	5	0.14	-13.87	0.36	ENSMUST00000202165	protein coding
Gtf2ird1-224	5	0.13	5.49	0.32	ENSMUST00000202280	protein coding
Gtf2ird1-225	5	5.34	1.59	0.33	ENSMUST00000202321	nonsense mediated decay
Gtf3a-201	5	46.11	1.31	0.27	ENSMUST00000132102	protein coding
Gtf3a-204	5	59.7	1.91	5.02E-04	ENSMUST00000146511	protein coding
Gzf1-201	2	33.09	1.67	5.62E-06	ENSMUST00000028928	protein coding
Gzf1-202	2	0.27	1.18	0.93	ENSMUST00000131292	protein coding
Hbp1-201	12	3.69	1.33	0.65	ENSMUST00000167458	protein coding
Hbp1-202	12	38.57	1.26	0.06	ENSMUST00000172314	protein coding
Hbp1-204	12	0.83	-1.62	0.73	ENSMUST00000175686	protein coding
Hbp1-206	12	1.94	1.44	0.69	ENSMUST00000176103	protein coding
Hdx-202	X	1.36	-2.65	0.07	ENSMUST00000113422	protein coding
Helt-201	8	0.03	7.56	NaN	ENSMUST00000058636	protein coding
Hes1-201	16	27.38	-1.09	0.75	ENSMUST00000023171	protein coding
Hes2-201	4	0.6	1.33	0.77	ENSMUST00000030782	protein coding
Hes3-201	4	0.06	5.48	0.31	ENSMUST00000094438	protein coding
Hes6-201	1	14.63	-1.04	0.88	ENSMUST00000086851	protein coding
Hey1-201	3	3.03	1.39	0.46	ENSMUST00000042412	protein coding
Hif1a-201	12	32.05	1.19	0.16	ENSMUST00000021530	protein coding
Hif1a-203	12	0.54	2.28	0.51	ENSMUST00000110464	protein coding

Hinfp-201	9	5.87	-1.33	0.64	ENSMUST00000034629	protein coding
Hinfp-202	9	0.15	-1.29	0.89	ENSMUST00000214660	protein coding
Hinfp-205	9	16.72	-1.27	0.04	ENSMUST00000216508	protein coding
Hmbox1-201	14	14.54	1.26	0.31	ENSMUST00000022544	protein coding
Hmbox1-202	14	4.95	1.17	0.72	ENSMUST00000067843	protein coding
Hmbox1-203	14	1.1	-1.26	0.82	ENSMUST00000175744	protein coding
Hmbox1-204	14	0.08	6.51	0.26	ENSMUST00000175905	protein coding
Hmbox1-205	14	4.03	2.84	0.17	ENSMUST00000176128	protein coding
Hmbox1-207	14	0.16	-1.74	0.78	ENSMUST00000176489	protein coding
Hmg20a-201	9	22.51	1.02	0.89	ENSMUST00000034879	protein coding
Hmg20a-202	9	0.05	-3.31	NaN	ENSMUST00000213242	protein coding
Hmg20a-203	9	0.42	1.48	0.71	ENSMUST00000214771	protein coding
Hmg20a-204	9	0.17	1.2	0.9	ENSMUST00000214869	protein coding
Hmg20a-206	9	2	2.21	0.17	ENSMUST00000217518	protein coding
Hnf4g-202	3	5.50E-03	4.26	NaN	ENSMUST00000108394	protein coding
Homez-201	14	0.06	49.57	0.08	ENSMUST00000081162	protein coding
Homez-202	14	8.44	1.99	8.29E-04	ENSMUST00000142283	protein coding
Homez-203	14	0.2	13.58	0.28	ENSMUST00000146642	protein coding
Hoxb5-201	11	0.02	-1.26	NaN	ENSMUST00000049272	protein coding
Hoxc12-201	15	0.36	-1.49	0.77	ENSMUST00000055562	protein coding
Hoxc13-201	15	3.07	1.93	0.22	ENSMUST00000001700	protein coding
Hoxc5-201	15	0.11	3.22	0.45	ENSMUST00000001709	protein coding
Hoxd1-201	2	0.38	1.36	0.82	ENSMUST00000047793	protein coding
Hoxd12-201	2	5.49E-03	-3.31	NaN	ENSMUST00000001878	protein coding
Hoxd3-203	2	7.83E-03	4.26	NaN	ENSMUST00000111983	protein coding
Hoxd4-201	2	0.03	10.51	NaN	ENSMUST00000047904	protein coding

Hoxd4-202	2	0.09	1.74	0.75	ENSMUST00000111980	protein coding
Hsf1-201	15	1.86	4.74	0.2	ENSMUST00000072838	protein coding
Hsf1-202	15	0.32	-1.64	0.78	ENSMUST00000226238	protein coding
Hsf1-203	15	2.15	1.4	0.62	ENSMUST00000226860	nonsense mediated decay
Hsf1-205	15	25.69	1.28	0.38	ENSMUST00000227478	protein coding
Hsf1-207	15	2.17	-1.82	0.43	ENSMUST00000228371	protein coding
Hsf1-209	15	0.7	-1.13	0.95	ENSMUST00000228688	protein coding
Hsf2-201	10	18.46	-1.41	0.19	ENSMUST00000079833	protein coding
Hsf2-204	10	3.34	1.17	0.81	ENSMUST00000220042	protein coding
Hsf2-206	10	1.63	2.15	0.27	ENSMUST00000220353	nonsense mediated decay
Ikzf1-201	11	0.07	-17.06	0.32	ENSMUST00000018798	protein coding
Ikzf1-204	11	0.04	25.54	0.17	ENSMUST00000076700	protein coding
Ikzf1-205	11	0.06	-5.57	NaN	ENSMUST00000126058	protein coding
Ikzf1-207	11	0.07	-1.57	0.8	ENSMUST00000240540	protein coding
Ikzf1-208	11	0.08	4.26	NaN	ENSMUST00000240541	protein coding
Ikzf1-209	11	5.47E-03	4.26	NaN	ENSMUST00000240542	protein coding
Ikzf1-210	11	0.02	-1.2	0.93	ENSMUST00000240543	protein coding
Ikzf1-211	11	0.07	7.89	NaN	ENSMUST00000240544	protein coding
Ikzf2-201	1	0.22	1.4	0.69	ENSMUST00000027146	protein coding
Ikzf2-202	1	0.22	-2.77	0.44	ENSMUST00000187184	protein coding
Ikzf2-206	1	0.12	-1.77	0.78	ENSMUST00000190016	protein coding
Ikzf2-207	1	0.06	2.31	0.6	ENSMUST00000190771	protein coding
Ikzf2-209	1	0.08	-14.63	0.34	ENSMUST00000191262	protein coding
Ikzf4-201	10	1.68	2.19	0.32	ENSMUST00000133342	protein coding
Ikzf4-203	10	0.05	-17.06	0.32	ENSMUST00000221150	protein coding
Ikzf4-204	10	0.34	1.61	0.75	ENSMUST00000222067	protein coding

Ikzf4-206	10	0.23	16.25	0.24	ENSMUST00000222901	nonsense mediated decay
Ikzf4-208	10	0.47	1.33	0.79	ENSMUST00000223162	nonsense mediated decay
Ikzf5-201	7	11.68	1.05	0.83	ENSMUST00000046306	protein coding
Ikzf5-202	7	1.3	2.78	0.36	ENSMUST00000121033	protein coding
Ikzf5-203	7	1.29	-2.94	0.34	ENSMUST00000128432	protein coding
Ikzf5-204	7	0.38	-1.51	0.82	ENSMUST00000142349	protein coding
Ikzf5-205	7	0.31	-24.3	0.27	ENSMUST00000154602	protein coding
Ikzf5-206	7	0.52	-3.96	0.4	ENSMUST00000207243	protein coding
Irf1-202	11	49.25	1.32	0.04	ENSMUST00000108920	protein coding
Irf1-203	11	0.13	-1.25	0.86	ENSMUST00000108922	protein coding
Irf1-204	11	0.22	24.28	0.16	ENSMUST00000123376	protein coding
Irf1-208	11	0.15	-20.37	0.29	ENSMUST00000140866	protein coding
Irf1-209	11	0.88	1.06	0.98	ENSMUST00000142221	nonsense mediated decay
Irf3-202	7	8.61	1.04	0.95	ENSMUST00000107834	protein coding
Irf3-206	7	4.52	2.38	0.22	ENSMUST00000207521	protein coding
Irf3-211	7	0.37	17.45	0.19	ENSMUST00000208958	protein coding
Irf5-201	6	2.84	1.77	0.51	ENSMUST00000004392	protein coding
Irf5-202	6	0.72	-1.04	1	ENSMUST00000163511	protein coding
Irf5-204	6	0.23	2.64	0.57	ENSMUST00000164922	protein coding
Irf5-205	6	0.28	-74.45	0.09	ENSMUST00000167252	protein coding
Irf8-201	8	1.53	-1.21	0.81	ENSMUST00000047737	protein coding
Irf8-204	8	0.14	24.91	0.17	ENSMUST00000160943	protein coding
Irf9-202	14	3.85	1.15	0.85	ENSMUST00000130697	protein coding
Irf9-203	14	2.37	1.18	0.78	ENSMUST00000134863	protein coding
Irf9-207	14	0.16	16.25	0.24	ENSMUST00000226275	protein coding
Irx6-202	8	0.03	1.44	0.83	ENSMUST00000167261	protein coding

Isx-201	8	0.12	-34.2	0.2	ENSMUST00000034034	protein coding
Jrk-201	15	5.74	-1.79	6.55E-03	ENSMUST00000050234	protein coding
Jrkl-201	9	4.64	1.63	0.2	ENSMUST00000110582	protein coding
Kat7-203	11	1.39	-1.93	0.5	ENSMUST00000103159	protein coding
Kcmf1-201	6	20.8	1.03	0.91	ENSMUST00000068697	protein coding
Kcmf1-204	6	110.11	1.4	9.17E-06	ENSMUST00000204598	protein coding
Kcmf1-205	6	0.19	2.62	0.57	ENSMUST00000204708	protein coding
Kcnip3-201	2	0.74	-1.74	0.61	ENSMUST00000028850	protein coding
Kcnip3-202	2	0.84	1.85	0.54	ENSMUST00000088538	protein coding
Kdm2a-201	19	0.56	2.47	0.13	ENSMUST00000047898	protein coding
Kdm2a-202	19	35.52	1.01	0.92	ENSMUST00000075856	protein coding
Kdm2a-203	19	0.06	16.72	0.24	ENSMUST00000116571	protein coding
Kdm2a-206	19	0.78	-1.11	0.95	ENSMUST00000175959	protein coding
Kdm2a-208	19	0.54	-8.49	0.12	ENSMUST00000176483	protein coding
Kdm2a-211	19	1.14	-6.6	0.2	ENSMUST00000176532	nonsense mediated decay
Kdm2b-201	5	31.9	1.92	2.08E-07	ENSMUST00000031435	protein coding
Kdm2b-204	5	1.54	-1.92	0.2	ENSMUST00000118027	protein coding
Kdm2b-205	5	0.12	-78.93	0.11	ENSMUST00000121739	protein coding
Kdm2b-214	5	0.18	26.28	0.15	ENSMUST00000145082	protein coding
Kdm2b-217	5	0.08	1.04	0.99	ENSMUST00000152872	protein coding
Kin-201	2	27.25	1.09	0.71	ENSMUST00000042512	protein coding
Klf1-201	8	0.51	1.81	0.68	ENSMUST00000067060	protein coding
Klf1-202	8	0.14	-8.14	0.26	ENSMUST00000239392	protein coding
Klf11-201	12	17.43	1.52	0.03	ENSMUST00000020982	protein coding
Klf11-202	12	0.06	4.26	NaN	ENSMUST00000139940	protein coding
Klf11-204	12	0.03	4.26	NaN	ENSMUST00000146894	protein coding

Klf16-201	10	57.13	1.6	2.66E-05	ENSMUST00000038558	protein coding
Klf7-201	1	1.3	1.13	0.9	ENSMUST00000055001	protein coding
Klf7-202	1	5.4	-1.09	0.75	ENSMUST00000114086	protein coding
Klf7-203	1	0.74	-5.73	0.13	ENSMUST00000135075	protein coding
Klf9-201	19	64.18	-1.55	3.94E-13	ENSMUST00000036884	protein coding
Kmt2b-201	7	24.57	-1.24	0.01	ENSMUST00000006470	protein coding
Kmt2b-206	7	9.67	-1.13	0.62	ENSMUST00000131002	protein coding
L3mbtl1-201	2	0.61	1.21	0.83	ENSMUST00000035751	protein coding
L3mbtl1-202	2	0.24	-2.07	0.69	ENSMUST00000124264	protein coding
L3mbtl1-207	2	0.02	-3.31	NaN	ENSMUST00000156954	protein coding
Lbx1-201	19	0.3	3	0.41	ENSMUST00000099401	protein coding
Lcor-202	19	0.15	1.05	0.98	ENSMUST00000163929	protein coding
Lcor-204	19	1.13	1.46	0.77	ENSMUST00000237208	protein coding
Lcor-206	19	8.43	-1.35	0.18	ENSMUST00000238398	protein coding
Lcor-207	19	6.57	1.25	0.42	ENSMUST00000238431	protein coding
Lcorl-201	5	1.3	1.27	0.71	ENSMUST00000016026	protein coding
Lcorl-208	5	0.05	4.26	NaN	ENSMUST00000187615	nonsense mediated decay
Lcorl-210	5	0.51	2.86	0.34	ENSMUST00000190036	protein coding
Lcorl-212	5	0.73	-1.07	0.91	ENSMUST00000238522	protein coding
Lhx3-202	2	0.15	2.52	0.54	ENSMUST00000054099	protein coding
Lhx8-203	3	0.03	-5.57	NaN	ENSMUST00000204171	nonsense mediated decay
Lhx8-204	3	0.16	5.48	0.28	ENSMUST00000204403	protein coding
Lhx8-205	3	0.04	-1.11	0.96	ENSMUST00000205251	protein coding
Lin28a-201	4	203.04	-1.7	2.83E-15	ENSMUST00000051674	protein coding
Lin28a-202	4	0.04	-3.31	NaN	ENSMUST00000176113	protein coding
Lin28a-203	4	0.14	-1.55	0.83	ENSMUST00000176292	protein coding

Lin54-201	5	23.49	1.52	1.57E-04	ENSMUST00000239512	protein coding
Lmx1a-202	1	0.17	-5.06	0.29	ENSMUST00000111377	protein coding
Lyl1-201	8	0.11	-1.42	0.83	ENSMUST00000037165	protein coding
Mafa-201	15	2.19	1.11	0.88	ENSMUST00000062002	protein coding
Mafg-201	11	56.74	1.9	3.21E-22	ENSMUST00000058162	protein coding
Mafg-202	11	2.67	3.05	0.23	ENSMUST00000106180	protein coding
Max-201	12	69.98	-1.34	6.41E-03	ENSMUST00000082136	protein coding
Max-206	12	6.36	-3.04	0.21	ENSMUST00000218653	nonsense mediated decay
Maz-202	7	0.21	1.16	0.94	ENSMUST00000205461	protein coding
Maz-203	7	25.53	1.34	0.35	ENSMUST00000205568	protein coding
Maz-205	7	0.18	1.44	0.84	ENSMUST00000206291	protein coding
Mbd1-201	18	8.96	1.54	0.06	ENSMUST00000097530	protein coding
Mbd1-204	18	24.68	1.77	1.79E-05	ENSMUST00000224332	protein coding
Mbd1-207	18	0.23	-108.42	0.09	ENSMUST00000239157	protein coding
Mbd2-201	18	25.87	1.14	0.65	ENSMUST00000074058	protein coding
Mbd2-202	18	0.81	-1.16	0.9	ENSMUST00000114946	protein coding
Mbd2-203	18	3.94	-3.7	0.23	ENSMUST00000127260	nonsense mediated decay
Mbd3-201	10	19.59	1.71	0.02	ENSMUST00000092295	protein coding
Mbd3-203	10	9.88	-1.5	0.41	ENSMUST00000105348	protein coding
Mbd3-204	10	54.6	1.6	0.06	ENSMUST00000105349	protein coding
Mbd4-201	6	13.65	-1.49	0.11	ENSMUST00000032469	protein coding
Mbd4-202	6	2.34	-1.17	0.8	ENSMUST00000122816	protein coding
Mbd4-205	6	0.81	-19.67	0.08	ENSMUST00000203643	protein coding
Mbd6-201	10	11.58	1.31	0.29	ENSMUST00000026476	protein coding
Mbd6-203	10	3.09	1.43	0.51	ENSMUST00000126243	nonsense mediated decay
Mbd6-204	10	0.13	13.38	0.28	ENSMUST00000136169	nonsense mediated decay

Mbd6-205	10	0.17	13.38	0.28	ENSMUST00000154851	protein coding
Mbd6-206	10	2.65	1.78	0.43	ENSMUST00000156208	protein coding
Mbd6-207	10	0.48	41.17	0.11	ENSMUST00000172567	protein coding
Mecom-201	3	0.16	2.37	NaN	ENSMUST00000061088	protein coding
Mecom-202	3	0.04	-1.56	0.82	ENSMUST00000108270	protein coding
Mecom-203	3	0.05	1.89	0.72	ENSMUST00000108271	protein coding
Mecom-204	3	9.54E-03	-5.57	NaN	ENSMUST00000166001	protein coding
Mecom-206	3	0.04	-1.35	0.89	ENSMUST00000172694	protein coding
Mecom-207	3	0.04	16.72	0.24	ENSMUST00000172697	protein coding
Mecom-208	3	0.01	-7.88	NaN	ENSMUST00000172754	nonsense mediated decay
Mecom-210	3	0.03	-3.38	0.51	ENSMUST00000173059	protein coding
Mecom-212	3	0.03	-1.11	0.96	ENSMUST00000173495	protein coding
Mecom-213	3	0.11	43.82	0.09	ENSMUST00000173899	protein coding
Mecom-215	3	0.03	-10.18	0.42	ENSMUST00000174413	protein coding
Mecp2-201	X	10.79	1.05	0.87	ENSMUST00000033770	protein coding
Mecp2-202	X	2.81	-1.13	0.66	ENSMUST00000100750	protein coding
Mecp2-203	X	1.39	2.01	0.52	ENSMUST00000123362	protein coding
Mef2c-201	13	0.03	-33.35	0.21	ENSMUST00000005722	protein coding
Mef2c-205	13	0.06	-9.72	0.42	ENSMUST00000195984	protein coding
Mef2c-208	13	0.02	4.26	NaN	ENSMUST00000196730	protein coding
Mef2c-211	13	0.06	-7.65	NaN	ENSMUST00000197145	protein coding
Mef2c-212	13	0.05	7.55	0.18	ENSMUST00000197146	protein coding
Mef2c-216	13	0.06	4.26	NaN	ENSMUST00000198064	protein coding
Mef2c-219	13	0.02	-5.57	NaN	ENSMUST00000198217	protein coding
Mef2c-221	13	0.04	-3.31	NaN	ENSMUST00000198916	protein coding
Mef2c-223	13	0.03	16.25	0.24	ENSMUST00000199105	protein coding

Mef2c-230	13	0.09	1.14	0.95	ENSMUST00000200138	protein coding
Meis2-202	2	0.15	-2.48	0.53	ENSMUST00000074285	protein coding
Meis2-203	2	0.61	1.25	0.82	ENSMUST00000102538	protein coding
Meis2-205	2	0.41	1.44	0.73	ENSMUST00000110907	protein coding
Meis2-206	2	0.4	1.9	0.54	ENSMUST00000110908	protein coding
Meox2-201	12	0.03	-10.12	0.41	ENSMUST00000041183	protein coding
Mesp1-201	7	0.14	2.47	0.55	ENSMUST00000032760	protein coding
Mga-201	2	44.91	-1.82	2.25E-15	ENSMUST00000046717	protein coding
Mga-203	2	0.12	-1.36	0.83	ENSMUST00000110773	protein coding
Mga-204	2	5.45	1.33	0.14	ENSMUST00000110774	protein coding
Mixl1-201	1	1.68	1.54	0.55	ENSMUST00000027778	protein coding
Mkx-201	18	4	1.63	0.25	ENSMUST00000079788	protein coding
Mkx-202	18	0.53	-5.32	0.2	ENSMUST00000176608	protein coding
Mlx-202	11	38.14	-1.52	0.03	ENSMUST00000107302	protein coding
Mlx-203	11	4.37	2	0.23	ENSMUST00000107303	protein coding
Mlx-207	11	1.82	2.86	0.4	ENSMUST00000149597	protein coding
Mlxip-201	5	27.35	1.46	7.32E-07	ENSMUST00000068237	protein coding
Mlxip-202	5	0.12	4.11	0.4	ENSMUST00000111596	protein coding
Mlxip-203	5	0.73	1.3	0.78	ENSMUST00000135961	nonsense mediated decay
Mlxipl-201	5	0.31	1.53	0.72	ENSMUST00000005507	protein coding
Mlxipl-202	5	0.24	23.67	0.17	ENSMUST00000123370	protein coding
Mlxipl-203	5	0.42	11.76	0.05	ENSMUST00000128691	nonsense mediated decay
Mlxipl-204	5	0.36	-1.88	0.65	ENSMUST00000129008	nonsense mediated decay
Mlxipl-206	5	0.65	2.34	0.22	ENSMUST00000142385	nonsense mediated decay
Mlxipl-207	5	0.81	1.05	0.97	ENSMUST00000153519	nonsense mediated decay
Mnt-201	11	25.71	1.65	3.01E-05	ENSMUST00000000291	protein coding

Mnt-202	11	0.22	-22.22	0.28	ENSMUST00000132150	protein coding
Msantd1-201	5	0.05	2.31	0.58	ENSMUST00000050535	protein coding
Msantd1-203	5	0.12	-14.79	0.33	ENSMUST00000202205	protein coding
Msantd1-204	5	0.13	1.42	0.84	ENSMUST00000212362	protein coding
Mterf1a-201	5	7.01	-1.75	0.33	ENSMUST00000044746	protein coding
Mterf1a-202	5	1.02	-1.37	0.73	ENSMUST00000117463	protein coding
Mterf1b-201	5	3.67	-1.37	0.65	ENSMUST00000177258	protein coding
Mterf2-201	10	10.99	-1.67	0.05	ENSMUST00000050813	protein coding
Mterf2-202	10	0.1	1.34	NaN	ENSMUST00000214193	protein coding
Mterf2-203	10	1.46	-5.56	0.25	ENSMUST00000214607	protein coding
Mterf2-204	10	0.19	17.44	0.2	ENSMUST00000217027	protein coding
Mterf3-201	13	35.76	-1.55	0.02	ENSMUST00000021991	protein coding
Mterf3-202	13	0.55	-70.4	0.1	ENSMUST00000172597	protein coding
Mterf3-205	13	0.87	1.01	1	ENSMUST00000173158	nonsense mediated decay
Mterf3-206	13	1.75	-1.4	0.73	ENSMUST00000173407	nonsense mediated decay
Mterf3-207	13	0.47	2.87	0.52	ENSMUST00000173773	protein coding
Mterf3-208	13	1.27	1.03	0.99	ENSMUST00000173891	protein coding
Mterf3-209	13	2.02	-2.39	0.33	ENSMUST00000173910	nonsense mediated decay
Mterf3-212	13	0.88	1.15	0.93	ENSMUST00000174339	protein coding
Mterf4-201	1	46.54	-1.13	0.59	ENSMUST00000027492	protein coding
Mterf4-202	1	3.72	-1.18	0.75	ENSMUST00000112942	protein coding
Mtf1-201	4	11.11	1.39	0.02	ENSMUST00000030723	protein coding
Mtf1-202	4	1.29	4.37	0.06	ENSMUST00000106193	protein coding
Mtf1-203	4	8.19	-1.92	0.31	ENSMUST00000138807	protein coding
Mtf1-205	4	0.36	1.29	0.88	ENSMUST00000175875	protein coding
Mxd1-201	6	3.11	1.47	0.34	ENSMUST00000001184	protein coding

Mxd3-201	13	5.79	-1.21	0.73	ENSMUST00000021941	protein coding
Mxi1-201	19	12.27	1.57	0.04	ENSMUST00000003870	protein coding
Mxi1-202	19	0.11	8.48	0.11	ENSMUST00000025998	protein coding
Mxi1-203	19	18.31	-1.25	0.33	ENSMUST00000111737	protein coding
Mxi1-204	19	0.26	36.5	0.14	ENSMUST00000235201	protein coding
Mxi1-208	19	0.57	1.25	0.87	ENSMUST00000237480	protein coding
Mxi1-209	19	0.67	-1.35	0.83	ENSMUST00000237837	protein coding
Mybl1-201	1	5.36	1.17	0.59	ENSMUST00000088658	protein coding
Mybl2-201	2	378.14	-1.19	0.03	ENSMUST00000018005	protein coding
Mybl2-205	2	9.59	-1.43	0.56	ENSMUST00000142729	protein coding
Myc-201	15	28.42	1.6	0.01	ENSMUST00000022971	protein coding
Myc-203	15	0.77	-1	1	ENSMUST00000159338	protein coding
Myc-204	15	15.02	-1.61	0.08	ENSMUST00000160009	protein coding
Myc-205	15	28.19	1.56	0.02	ENSMUST00000161976	protein coding
Mycs-201	X	0.01	-5.59	NaN	ENSMUST00000058404	protein coding
Myf5-201	10	0.01	4.26	NaN	ENSMUST00000000445	protein coding
Myf6-201	10	0.02	-5.57	NaN	ENSMUST00000044210	protein coding
Mynn-201	3	9.59	-1.85	0.01	ENSMUST00000047502	protein coding
Mynn-202	3	1.66	-1.04	0.95	ENSMUST00000192715	protein coding
Myod1-201	7	0.05	3.41	0.49	ENSMUST00000072514	protein coding
Myog-201	1	0.08	1.45	0.83	ENSMUST00000027730	protein coding
Myrfl-201	10	0.19	-1.14	0.93	ENSMUST00000048229	protein coding
Mysm1-201	4	13.34	-1.6	1.10E-05	ENSMUST00000075872	protein coding
Myt1-203	2	0.01	10.6	NaN	ENSMUST00000108757	protein coding
Myt1-204	2	0.02	-2.89	0.59	ENSMUST00000129843	protein coding
Myt1-205	2	0.01	1.34	NaN	ENSMUST00000129856	protein coding

Myt1l-201	12	0.01	-1.16	0.95	ENSMUST00000021009	protein coding
Myt1l-202	12	9.46E-03	1.04	0.99	ENSMUST00000049784	protein coding
Myt1l-204	12	0.02	-10.11	0.41	ENSMUST00000218198	protein coding
Myt1l-205	12	4.96E-03	4.26	NaN	ENSMUST00000218583	protein coding
Mzf1-204	7	0.95	1.29	0.79	ENSMUST00000182490	protein coding
Mzf1-205	7	0.17	1.95	0.7	ENSMUST00000182515	protein coding
Nacc2-201	2	2.35	1.07	0.87	ENSMUST00000028300	protein coding
Nacc2-206	2	0.11	2.37	NaN	ENSMUST00000140993	protein coding
Naif1-201	2	4.69	1.08	0.79	ENSMUST00000048431	protein coding
Ncoa1-201	12	3.21	-1.14	0.65	ENSMUST00000085814	protein coding
Ncoa1-203	12	0.06	2.44	0.63	ENSMUST00000217794	nonsense mediated decay
Ncoa1-205	12	0.24	1.48	0.81	ENSMUST00000218191	protein coding
Ncoa1-206	12	0.36	-8.59	0.26	ENSMUST00000219275	protein coding
Ncoa1-207	12	0.88	-1.35	0.82	ENSMUST00000219373	protein coding
Ncoa1-209	12	1	1.69	0.47	ENSMUST00000220434	protein coding
Ncoa2-201	1	8.09	1.17	0.5	ENSMUST00000006037	protein coding
Ncoa2-202	1	13.98	1.98	3.80E-09	ENSMUST00000068304	protein coding
Ncoa2-208	1	0.11	7.56	NaN	ENSMUST00000145280	protein coding
Ncoa3-201	2	37.34	-1.3	6.01E-05	ENSMUST00000088095	protein coding
Ncoa3-202	2	59.45	1.05	0.63	ENSMUST00000109252	protein coding
Neurod2-201	11	0.01	7.56	NaN	ENSMUST00000041685	protein coding
Neurog1-201	13	0.05	1.11	0.95	ENSMUST00000058475	protein coding
Neurog2-201	3	0.06	20.74	0.18	ENSMUST00000029587	protein coding
Nfat5-202	8	8.85	-1.91	1.06E-06	ENSMUST00000077440	protein coding
Nfat5-207	8	0.34	-1.14	0.95	ENSMUST00000144100	protein coding
Nfat5-208	8	2.83	-1.02	0.97	ENSMUST00000147588	protein coding

Nfatc3-201	8	31.82	-1.01	0.94	ENSMUST00000109308	protein coding
Nfe2l1-211	11	33.13	1.17	0.34	ENSMUST00000167149	protein coding
Nfe2l2-201	2	89.77	1.19	0.02	ENSMUST00000102672	protein coding
Nfe2l3-201	6	1.29	1.55	0.58	ENSMUST00000005103	protein coding
Nfe2l3-203	6	0.06	3.41	0.48	ENSMUST00000160133	protein coding
Nfil3-201	13	9.34	-1.15	0.57	ENSMUST00000071065	protein coding
Nfx1-201	4	21.86	-1.35	0.03	ENSMUST00000030133	protein coding
Nfx1-202	4	51.93	-1.6	2.53E-07	ENSMUST00000091614	protein coding
Nfx1-203	4	35.14	-1.35	4.59E-04	ENSMUST00000098143	protein coding
Nfxl1-201	5	0.76	7.8	0.13	ENSMUST00000074948	protein coding
Nfxl1-202	5	31.32	1.74	5.24E-08	ENSMUST00000087216	protein coding
Nfxl1-204	5	1.03	2.15	0.44	ENSMUST00000135318	nonsense mediated decay
Nfya-201	17	19.08	-1.71	6.83E-03	ENSMUST00000046719	protein coding
Nfya-202	17	11.33	-1.7	0.06	ENSMUST00000078800	protein coding
Nfya-205	17	2.91	3.11	0.14	ENSMUST00000159535	protein coding
Nfya-206	17	0.27	51.01	0.1	ENSMUST00000160319	protein coding
Nfya-209	17	0.69	-74.9	0.1	ENSMUST00000161256	protein coding
Nfyb-204	10	70.82	-1.53	8.52E-09	ENSMUST00000130911	protein coding
Nfyc-204	4	1.69	-1.3	0.76	ENSMUST00000120779	protein coding
Nfyc-206	4	0.52	3.13	0.45	ENSMUST00000134979	protein coding
Nfyc-207	4	0.12	-9.72	0.42	ENSMUST00000136236	protein coding
Nfyc-209	4	0.96	-1.38	0.82	ENSMUST00000145658	protein coding
Nhlh2-202	3	0.04	-17.92	0.32	ENSMUST00000196324	protein coding
Nkrf-201	X	18.23	-1.38	0.13	ENSMUST00000057093	protein coding
Nkrf-202	X	5.32	1.48	0.15	ENSMUST00000239259	protein coding
Nkx2-1-202	12	0.02	2.37	NaN	ENSMUST00000178477	protein coding

Nkx3-1-201	14	2.52	1.86	0.17	ENSMUST00000022646	protein coding
Nkx3-2-201	5	0.06	14.15	0.23	ENSMUST00000060820	protein coding
Nkx6-2-201	7	0.85	-3.13	0.29	ENSMUST00000097974	nonsense mediated decay
Nkx6-2-202	7	2.56	1.3	0.75	ENSMUST00000106095	protein coding
Nobox-201	6	0.04	1.44	0.84	ENSMUST00000031749	protein coding
Nobox-202	6	0.04	-1	1	ENSMUST00000172554	protein coding
Noto-201	6	0.03	-7.84	NaN	ENSMUST00000089578	protein coding
Npas4-203	19	0.07	1.02	1	ENSMUST00000237881	nonsense mediated decay
Nr1h2-201	7	17.32	-1.49	0.1	ENSMUST00000073488	protein coding
Nr1h2-202	7	12.28	1.15	0.69	ENSMUST00000107910	protein coding
Nr1h2-207	7	0.48	1.95	0.7	ENSMUST00000128600	protein coding
Nr1h2-212	7	2.99	-1.51	0.61	ENSMUST00000142298	protein coding
Nr1h2-213	7	0.07	-21.64	0.29	ENSMUST00000167197	protein coding
Nr1h2-215	7	0.58	33.92	0.11	ENSMUST00000207737	protein coding
Nr1h2-216	7	0.06	13.58	0.27	ENSMUST00000208322	protein coding
Nr1h2-217	7	0.39	-3.17	0.48	ENSMUST00000208366	protein coding
Nr1h4-201	10	0.05	-1.87	0.73	ENSMUST00000058126	protein coding
Nr1i2-201	16	0.1	-1.23	0.9	ENSMUST00000023504	protein coding
Nr1i3-202	1	0.01	-3.31	NaN	ENSMUST00000075469	protein coding
Nr1i3-208	1	0.14	1.33	0.84	ENSMUST00000155126	protein coding
Nr2c2-201	6	5.15	-1.09	0.78	ENSMUST00000113460	protein coding
Nr2c2-202	6	1.84	-1.68	0.49	ENSMUST00000113463	protein coding
Nr2c2-203	6	0.17	1.86	0.72	ENSMUST00000133133	protein coding
Nr2c2-205	6	8.96	-1.65	0.03	ENSMUST00000146175	nonsense mediated decay
Nr3c1-201	18	0.91	-1.45	0.67	ENSMUST00000025300	protein coding
Nr3c1-204	18	3.96	1.35	0.25	ENSMUST00000115571	protein coding

Nr3c1-205	18	0.18	-19.04	0.29	ENSMUST000001 24115	protein coding
Nr3c1-206	18	0.36	4.48	0.33	ENSMUST000001 31885	protein coding
Nr3c1-207	18	0.1	-5.59	NaN	ENSMUST000001 50483	protein coding
Nr3c1-208	18	1.91	2.47	0.25	ENSMUST000001 52853	protein coding
Nr3c2-201	8	7.87E-03	4.26	NaN	ENSMUST000000 34031	protein coding
Nr3c2-202	8	0.11	52.64	0.08	ENSMUST000001 09911	protein coding
Nr3c2-203	8	0.16	-1.18	0.88	ENSMUST000001 09912	protein coding
Nr3c2-206	8	0.07	-7.65	NaN	ENSMUST000001 28862	protein coding
Nr3c2-208	8	0.11	-57.29	0.12	ENSMUST000001 48106	nonsense mediated decay
Nr4a1-201	15	45.31	1.37	0.03	ENSMUST000000 23779	protein coding
Nr4a1-203	15	0.41	-2.1	0.67	ENSMUST000002 30814	protein coding
Nr4a2-201	2	0.14	2.18	0.68	ENSMUST000000 28166	protein coding
Nr4a2-202	2	0.21	-1.42	0.84	ENSMUST000001 12627	protein coding
Nr4a2-203	2	0.29	1.33	0.86	ENSMUST000001 12629	protein coding
Nr4a2-206	2	0.03	-17.06	0.32	ENSMUST000001 83542	nonsense mediated decay
Nr6a1-207	2	0.04	-5.59	NaN	ENSMUST000001 42113	protein coding
Nr6a1-208	2	0.24	-2.25	0.67	ENSMUST000001 42130	protein coding
Nrf1-202	6	2.42	-1.04	1	ENSMUST000000 69808	protein coding
Nrf1-204	6	1.82	-1.41	0.66	ENSMUST000001 15199	protein coding
Nrf1-205	6	1.11	-1.89	0.41	ENSMUST000001 15200	protein coding
Nrf1-207	6	16.91	1	1	ENSMUST000001 15206	protein coding
Nrf1-208	6	0.11	-44.56	0.18	ENSMUST000001 15208	protein coding
Nrf1-210	6	13.18	1.34	0.19	ENSMUST000001 15211	protein coding
Nrf1-212	6	1.81	1.04	0.98	ENSMUST000001 23194	protein coding
Nrf1-218	6	0.14	-11.79	0.39	ENSMUST000001 48990	protein coding
Nrf1-220	6	5.15	-11.8	0.37	ENSMUST000001 70535	protein coding

Nrl-203	14	0.01	4.26	NaN	ENSMUST00000178694	protein coding
Nrl-207	14	0.02	-3.31	NaN	ENSMUST00000228902	protein coding
Olig3-201	10	0.03	-12.19	0.38	ENSMUST00000053225	protein coding
Osr1-201	12	0.01	4.26	NaN	ENSMUST00000057021	protein coding
Osr2-201	15	0.27	1.58	0.74	ENSMUST00000022952	protein coding
Osr2-204	15	0.08	-17.06	0.32	ENSMUST00000228152	protein coding
Otp-201	13	0.03	-1.05	0.99	ENSMUST00000022195	protein coding
Ovol3-201	7	0.09	10.85	NaN	ENSMUST00000189482	protein coding
Patz1-201	11	23.98	-1.62	8.60E-04	ENSMUST00000057089	protein coding
Patz1-202	11	6.03	3.41	0.08	ENSMUST00000093402	protein coding
Patz1-203	11	12.56	-1.26	0.28	ENSMUST00000094471	protein coding
Patz1-205	11	2.21	2.59	0.09	ENSMUST00000110043	protein coding
Patz1-212	11	0.16	2.02	0.68	ENSMUST00000154319	protein coding
Pax1-201	2	0.17	1.29	0.86	ENSMUST00000109968	protein coding
Pax2-201	19	0.2	3.06	0.36	ENSMUST00000004340	protein coding
Pax2-202	19	0.02	-5.57	NaN	ENSMUST00000173346	protein coding
Pax2-203	19	0.04	-2.27	0.69	ENSMUST00000174490	protein coding
Pax4-205	6	0.01	-3.31	NaN	ENSMUST00000174194	protein coding
Pax6-201	2	0.1	1.06	0.98	ENSMUST00000090391	protein coding
Pax6-202	2	0.27	-1.6	0.68	ENSMUST00000090397	protein coding
Pax6-203	2	0.09	37.08	0.11	ENSMUST00000111082	protein coding
Pax6-204	2	0.74	-1.14	0.9	ENSMUST00000111083	protein coding
Pax6-205	2	0.07	3.35	0.49	ENSMUST00000111085	protein coding
Pax6-207	2	0.29	-2.15	0.66	ENSMUST00000111087	protein coding
Pax6-214	2	0.08	-7.88	NaN	ENSMUST00000142772	protein coding
Pax6-224	2	0.05	10.85	NaN	ENSMUST00000156216	protein coding

Pax6-225	2	0.11	4.11	0.42	ENSMUST00000167211	protein coding
Pbx2-203	17	28.44	-1.09	0.77	ENSMUST00000173328	protein coding
Pbx2-204	17	0.8	1.36	0.84	ENSMUST00000174532	protein coding
Pcgf2-201	11	0.95	1.44	0.73	ENSMUST00000018681	protein coding
Pcgf2-202	11	48.87	-1.45	0.02	ENSMUST00000103148	protein coding
Pcgf6-201	19	68.84	-1.88	2.73E-10	ENSMUST00000026032	protein coding
Pcgf6-203	19	1.72	-1.19	0.84	ENSMUST00000235633	nonsense mediated decay
Phf1-202	17	0.06	1.38	0.88	ENSMUST00000236269	nonsense mediated decay
Phf1-205	17	2.84	1.47	0.4	ENSMUST00000237189	protein coding
Phf20-201	2	58.95	-1.46	2.94E-09	ENSMUST00000037401	protein coding
Pin1-201	9	58.68	1.41	4.10E-05	ENSMUST00000034689	protein coding
Pin1rt1-201	2	0.04	7.56	NaN	ENSMUST00000099659	protein coding
Pknox1-201	17	16.49	1.2	0.39	ENSMUST00000097352	protein coding
Pknox1-202	17	2.25	1.22	0.81	ENSMUST00000175806	protein coding
Pknox1-205	17	0.39	-10.22	0.18	ENSMUST00000176701	protein coding
Pknox1-207	17	0.19	-1.66	0.8	ENSMUST00000235255	protein coding
Pknox1-208	17	0.32	-10.81	0.43	ENSMUST00000235401	nonsense mediated decay
Pknox1-212	17	1.65	1.49	0.62	ENSMUST00000237248	protein coding
Pknox1-213	17	0.24	-45.21	0.18	ENSMUST00000237748	protein coding
Pknox1-214	17	0.15	-1.84	0.78	ENSMUST00000237887	nonsense mediated decay
Pknox2-201	9	0.07	34.53	0.14	ENSMUST00000039674	protein coding
Pknox2-207	9	0.33	-1.13	0.91	ENSMUST00000177218	protein coding
Plagl1-201	10	0.02	10.6	NaN	ENSMUST00000121325	protein coding
Plagl1-202	10	0.07	3.45	0.43	ENSMUST00000121646	protein coding
Plagl1-203	10	0.04	-1.35	0.88	ENSMUST00000121766	protein coding
Plagl1-208	10	0.31	-31.46	0.22	ENSMUST00000130313	protein coding

Plagl1-222	10	0.05	4.26	NaN	ENSMUST000001 45103	protein coding
Plagl2-201	2	11.09	-1.12	0.67	ENSMUST000000 56924	protein coding
Plscr2-201	9	0.02	7.56	NaN	ENSMUST000000 34932	protein coding
Plscr2-202	9	0.08	-2.14	0.7	ENSMUST000001 13044	protein coding
Plscr2-203	9	0.02	-3.31	NaN	ENSMUST000001 26911	protein coding
Pou1f1-203	16	0.02	4.26	NaN	ENSMUST000001 76330	protein coding
Pou2af1-202	9	0.02	4.26	NaN	ENSMUST000002 14322	protein coding
Pou2f1-206	1	1.53	1.86	0.44	ENSMUST000001 11429	protein coding
Pou2f1-207	1	1.24	-1.11	0.95	ENSMUST000001 59212	protein coding
Pou2f1-208	1	18.91	-1.44	1.31E-05	ENSMUST000001 60260	protein coding
Pou2f1-212	1	0.31	10.85	NaN	ENSMUST000001 76800	protein coding
Pou2f1-214	1	0.58	-3.08	0.39	ENSMUST000001 77358	protein coding
Pou2f1-217	1	0.45	3.64	0.45	ENSMUST000001 84799	protein coding
Pou2f2-201	7	0.1	2.18	0.65	ENSMUST000000 98679	protein coding
Pou2f2-205	7	0.08	-3.23	0.55	ENSMUST000001 08417	protein coding
Pou2f2-206	7	0.22	2.03	0.57	ENSMUST000001 08418	protein coding
Pou2f2-207	7	0.23	1.74	0.75	ENSMUST000001 47146	protein coding
Pou2f2-209	7	0.71	-1.86	0.37	ENSMUST000001 75774	protein coding
Pou2f2-210	7	8.19E-03	-3.31	NaN	ENSMUST000001 76408	protein coding
Pou3f2-201	4	4.02E-03	1.34	NaN	ENSMUST000001 78174	protein coding
Pou3f4-201	X	0.07	-34.89	0.19	ENSMUST000000 78229	protein coding
Pou4f1-201	14	0.91	-1.71	0.46	ENSMUST000000 53016	protein coding
Pou5f2-201	13	0.02	1.34	NaN	ENSMUST000001 75955	protein coding
Pou6f1-204	15	0.21	9.53	0.19	ENSMUST000001 76300	nonsense mediated decay
Pou6f2-202	13	0.06	-4.33	0.42	ENSMUST000001 75703	protein coding
Ppara-201	15	0.14	-5.27	0.32	ENSMUST000000 57979	protein coding

Ppara-202	15	0.45	3.6	0.07	ENSMUST00000109422	protein coding
Ppara-203	15	0.14	1.98	0.68	ENSMUST00000109423	protein coding
Prdm10-201	9	0.17	2.07	0.67	ENSMUST00000074510	protein coding
Prdm10-202	9	4.04	1.14	0.83	ENSMUST00000117389	protein coding
Prdm10-204	9	7.16	-1.32	0.22	ENSMUST00000215499	protein coding
Prdm10-205	9	3.02	-1.29	0.6	ENSMUST00000215847	protein coding
Prdm12-201	2	0.05	-1.7	0.75	ENSMUST00000113470	protein coding
Prdm13-201	4	0.15	3.09	0.37	ENSMUST00000076206	protein coding
Prdm15-203	16	4.53	-1.58	0.06	ENSMUST00000121584	protein coding
Prdm15-210	16	1.6	-1.25	0.73	ENSMUST00000142295	nonsense mediated decay
Prdm2-201	4	25.63	-1.22	5.56E-03	ENSMUST00000105778	protein coding
Prdm2-202	4	0.04	4.26	NaN	ENSMUST00000134791	protein coding
Preb-201	5	7.13	-1.14	0.66	ENSMUST00000074840	protein coding
Preb-206	5	0.03	-1.03	0.99	ENSMUST00000201821	protein coding
Preb-209	5	39.32	-1.14	0.4	ENSMUST00000202567	protein coding
Prmt3-201	7	42.28	-1.51	4.81E-04	ENSMUST00000032715	protein coding
Prmt3-206	7	0.24	3.68	0.4	ENSMUST00000147401	nonsense mediated decay
Prr12-201	7	30.18	1.73	6.25E-07	ENSMUST00000057293	protein coding
Prrx2-202	2	3.34	-2.22	0.24	ENSMUST00000113592	protein coding
Ptf1a-201	2	0.03	7.56	NaN	ENSMUST00000028068	protein coding
Rag1-201	2	0.01	10.85	NaN	ENSMUST00000078494	protein coding
Rara-202	11	12.29	-1.15	0.61	ENSMUST00000107473	protein coding
Rarb-202	14	0.03	4.26	NaN	ENSMUST00000223576	nonsense mediated decay
Rarb-203	14	0.03	4.26	NaN	ENSMUST00000223976	protein coding
Rarb-206	14	0.11	-12.38	0.37	ENSMUST00000225594	nonsense mediated decay
Rarb-207	14	0.02	-1.8	0.78	ENSMUST00000225921	protein coding

Rax-201	18	0.25	3.26	0.34	ENSMUST00000025396	protein coding
Rax-207	18	0.02	-7.65	NaN	ENSMUST00000238035	protein coding
Rbck1-207	2	2.4	1.95	0.51	ENSMUST00000144865	protein coding
Rela-201	19	20.86	1.16	0.5	ENSMUST00000025867	protein coding
Relb-202	7	6.99	2.47	0.12	ENSMUST00000094762	protein coding
Relb-206	7	0.03	4.26	NaN	ENSMUST00000141586	protein coding
Relb-208	7	0.81	1.94	0.58	ENSMUST00000153309	protein coding
Repin1-201	6	0.35	12.27	0.08	ENSMUST00000009420	protein coding
Repin1-202	6	4	1.9	0.31	ENSMUST00000118229	protein coding
Repin1-204	6	3.45	1.26	0.83	ENSMUST00000135151	protein coding
Repin1-206	6	0.15	25.54	0.17	ENSMUST00000154010	protein coding
Rexo4-201	2	51.62	-1.53	5.01E-03	ENSMUST00000064244	protein coding
Rexo4-207	2	1.58	3.11	0.27	ENSMUST00000136710	protein coding
Rfx1-204	8	0.4	1.58	0.68	ENSMUST00000211046	protein coding
Rfx3-201	19	0.79	-1.19	0.8	ENSMUST00000046898	protein coding
Rfx3-202	19	1.8	1.62	0.11	ENSMUST00000165566	protein coding
Rfx3-204	19	0.21	13.46	0.08	ENSMUST00000172907	protein coding
Rfx3-207	19	0.17	-10.18	0.42	ENSMUST00000174420	protein coding
Rfx3-208	19	1.04	-1.93	0.39	ENSMUST00000174850	protein coding
Rfx5-205	3	0.15	3.44	0.44	ENSMUST00000107260	protein coding
Rfx5-206	3	0.04	4.26	NaN	ENSMUST00000132393	protein coding
Rfx5-210	3	0.18	1.82	0.75	ENSMUST00000142311	protein coding
Rfx5-212	3	2.87	-1.29	0.66	ENSMUST00000144132	nonsense mediated decay
Rfx5-217	3	0.34	-7.01	0.33	ENSMUST00000152869	protein coding
Rfx6-204	10	5.74E-03	-3.31	NaN	ENSMUST00000219364	protein coding
Rfx6-205	10	0.02	-1.01	1	ENSMUST00000219771	nonsense mediated decay

Rfx7-201	9	26.04	-1.7	1.98E-13	ENSMUST00000163401	protein coding
Rfx7-202	9	7.37	-1.58	0.36	ENSMUST00000183372	protein coding
Rfx7-204	9	0.13	28.54	0.16	ENSMUST00000184015	protein coding
Rfx8-202	1	0.31	1.9	0.65	ENSMUST00000151913	protein coding
Rlf-201	4	37.11	-1.06	0.55	ENSMUST00000056635	protein coding
Rora-202	9	0.9	1.28	0.8	ENSMUST00000113624	protein coding
Rorc-201	3	0.03	-10.11	0.4	ENSMUST00000029795	protein coding
Rorc-202	3	0.05	14.84	0.24	ENSMUST00000197040	protein coding
Rorc-204	3	0.03	1.41	0.86	ENSMUST00000200009	protein coding
Rreb1-201	13	7.39	1.51	0.27	ENSMUST00000037232	protein coding
Rreb1-202	13	0.06	37.53	0.13	ENSMUST00000110237	protein coding
Rreb1-204	13	0.51	30.63	0.12	ENSMUST00000122842	protein coding
Rreb1-206	13	0.2	-2.14	0.64	ENSMUST00000124373	nonsense mediated decay
Rreb1-207	13	10.71	2.56	0.08	ENSMUST00000128570	protein coding
Rreb1-208	13	2.56	2.25	0.54	ENSMUST00000138043	protein coding
Rreb1-209	13	0.56	-3.07	0.45	ENSMUST00000138110	protein coding
Rreb1-210	13	7.75	1.67	0.07	ENSMUST00000149745	protein coding
Rslcan18-201	13	0.8	-1.97	0.41	ENSMUST00000091526	protein coding
Rxrb-202	17	29.51	-1.15	0.44	ENSMUST00000116612	protein coding
Rxrb-203	17	3.83	1.15	0.84	ENSMUST00000173354	protein coding
Rxrb-207	17	2.22	2.45	0.41	ENSMUST00000174299	protein coding
Safb-201	17	85.48	1.24	2.43E-03	ENSMUST00000095224	protein coding
Safb-207	17	23.95	-1.1	0.59	ENSMUST00000182533	protein coding
Safb-208	17	1.36	63.58	0.05	ENSMUST00000182800	protein coding
Safb2-201	17	47.24	1.41	2.06E-03	ENSMUST00000075510	protein coding
Safb2-202	17	1.09	-1.03	0.98	ENSMUST00000124111	protein coding

Safb2-205	17	2.75	-1.93	0.44	ENSMUST00000131056	protein coding
Safb2-206	17	0.12	-32.66	0.23	ENSMUST00000133604	nonsense mediated decay
Safb2-210	17	0.03	-3.31	NaN	ENSMUST00000142752	protein coding
Safb2-211	17	0.36	56.9	0.08	ENSMUST00000142940	protein coding
Safb2-212	17	7.25	1.47	0.19	ENSMUST00000144255	nonsense mediated decay
Safb2-216	17	0.86	-1.97	0.56	ENSMUST00000155983	nonsense mediated decay
Sall3-201	18	1.86	-1.49	0.2	ENSMUST00000057950	protein coding
Sall3-203	18	1.83	2.49	0.21	ENSMUST00000238808	protein coding
Sall4-201	2	83.97	-1.09	0.44	ENSMUST00000029061	protein coding
Sall4-202	2	6.75	27.82	0.18	ENSMUST00000075044	protein coding
Sall4-203	2	2.85	1.42	0.56	ENSMUST00000103074	protein coding
Sall4-206	2	3.48	2.1	0.36	ENSMUST00000137536	protein coding
Sall4-207	2	9.05	-3.75	0.06	ENSMUST00000150588	protein coding
Scrt1-201	15	0.04	-1.43	0.83	ENSMUST00000096365	protein coding
Scrt2-201	2	0.06	-4.38	0.38	ENSMUST00000064061	protein coding
Sebox-202	11	0.1	-1.94	0.71	ENSMUST00000125670	protein coding
Setdb1-202	3	59.6	-1.19	0.01	ENSMUST00000107170	protein coding
Setdb1-203	3	15.74	-1.88	7.88E-04	ENSMUST00000107171	protein coding
Sgsm2-201	11	4.91	-1.09	0.78	ENSMUST00000057631	protein coding
Sgsm2-202	11	2.72	1.96	0.19	ENSMUST00000081799	protein coding
Six4-201	12	7.15	-1.47	0.08	ENSMUST00000043208	protein coding
Six4-202	12	0.62	-1.69	0.45	ENSMUST00000175693	protein coding
Six5-201	7	3.94	1.59	0.36	ENSMUST00000049454	protein coding
Six6-201	12	0.03	-2.16	0.64	ENSMUST00000021519	protein coding
Skil-204	3	32.89	-1.22	0.01	ENSMUST00000118470	protein coding
Smad1-202	8	14.04	1.14	0.65	ENSMUST00000066091	protein coding

Smad1-203	8	0.3	1	1	ENSMUST00000109885	protein coding
Smad4-202	18	18.13	-1.77	0.01	ENSMUST00000114939	protein coding
Smad5-201	13	17.37	-1.72	7.10E-06	ENSMUST00000069557	protein coding
Smad5-203	13	0.52	2.46	0.25	ENSMUST00000109876	protein coding
Smyd3-202	1	7.62	1.22	0.73	ENSMUST00000111134	protein coding
Smyd3-204	1	7.24	-1.58	0.06	ENSMUST00000128302	protein coding
Smyd3-207	1	3.46	1.07	0.93	ENSMUST00000194237	protein coding
Snapc2-201	8	40.9	1.71	0.01	ENSMUST00000011981	protein coding
Snapc2-203	8	0.11	16.72	0.24	ENSMUST00000208316	protein coding
Snapc2-204	8	1.52	3.52	0.14	ENSMUST00000208459	protein coding
Snapc4-202	2	27.64	-1.03	0.93	ENSMUST00000114115	protein coding
Snapc4-203	2	0.22	-1.59	0.82	ENSMUST00000123934	protein coding
Sohlh1-201	2	0.11	-23.93	0.25	ENSMUST00000076989	protein coding
Son-201	16	25.31	1.57	7.16E-08	ENSMUST00000114036	protein coding
Son-202	16	43.8	-1.1	0.29	ENSMUST00000114037	protein coding
Son-204	16	14.17	-1.4	5.27E-03	ENSMUST00000119368	protein coding
Son-212	16	11.39	-1.02	0.95	ENSMUST00000147891	nonsense mediated decay
Sox12-201	2	18.53	-1.05	0.79	ENSMUST00000182625	protein coding
Sox13-201	1	1.34	-6.37	0.09	ENSMUST00000094551	protein coding
Sox13-203	1	14.61	-1.82	4.17E-03	ENSMUST00000144386	protein coding
Sox2-201	3	191.92	-1.09	0.31	ENSMUST00000099151	protein coding
Sox4-201	13	11.42	1.22	0.4	ENSMUST00000067230	protein coding
Sox5-202	6	0.02	7.56	NaN	ENSMUST00000077160	protein coding
Sox5-204	6	0.03	-2.29	0.67	ENSMUST00000111748	protein coding
Sox5-205	6	0.02	-14.61	0.33	ENSMUST00000111749	protein coding
Sox5-211	6	0.06	-1.77	0.78	ENSMUST00000144289	protein coding

Sox5-214	6	9.37E-03	10.51	NaN	ENSMUST00000170367	protein coding
Sox6-203	7	0.1	-44.85	0.18	ENSMUST00000166207	protein coding
Sox6-204	7	0.06	-1.2	0.91	ENSMUST00000166877	protein coding
Sox6-206	7	0.02	10.6	NaN	ENSMUST00000205405	protein coding
Sox6-207	7	0.13	-1.8	0.78	ENSMUST00000205479	protein coding
Sox6-210	7	0.05	1.37	0.84	ENSMUST00000206034	protein coding
Sox6-211	7	0.08	4.26	NaN	ENSMUST00000206123	protein coding
Sox6-212	7	0.03	-2.92	0.58	ENSMUST00000206369	protein coding
Sp1-201	15	60.38	-1.29	7.60E-04	ENSMUST00000001326	protein coding
Sp1-203	15	0.43	37.87	0.12	ENSMUST00000165837	protein coding
Sp1-204	15	0.79	2.72	0.45	ENSMUST00000168802	protein coding
Sp1-205	15	3.11	-1.43	0.73	ENSMUST00000169619	protein coding
Sp1-206	15	0.21	7.56	NaN	ENSMUST00000170884	protein coding
Sp2-201	11	1.1	-1.52	0.61	ENSMUST00000062652	protein coding
Sp2-203	11	19.8	1.25	0.33	ENSMUST00000107624	protein coding
Sp3-201	2	59.64	-1.29	7.35E-04	ENSMUST00000066003	protein coding
Sp5-201	2	0.18	-1.89	0.61	ENSMUST00000100043	protein coding
Sp7-201	15	0.04	-19.35	0.31	ENSMUST00000078508	protein coding
Sp7-202	15	0.05	-1.51	0.83	ENSMUST00000229464	protein coding
Sp9-201	2	0.02	-3.82	0.43	ENSMUST00000090813	protein coding
Spen-201	4	20.85	1.31	1.76E-03	ENSMUST00000078886	protein coding
Spz1-201	13	0.02	4.26	NaN	ENSMUST00000050658	protein coding
Srcap-201	7	0.51	2.11	0.33	ENSMUST00000066582	protein coding
Srcap-203	7	0.1	53.7	0.07	ENSMUST00000186672	protein coding
Srcap-206	7	15.74	-1.05	0.7	ENSMUST00000187040	protein coding
Srcap-209	7	6.95	2.14	0.11	ENSMUST00000188124	protein coding

Srcap-210	7	0.13	-1.11	0.95	ENSMUST00000189136	protein coding
Srcap-213	7	18.38	-1.49	1.64E-04	ENSMUST00000190390	protein coding
Srebf1-203	11	2.67	-1.6	0.36	ENSMUST00000134660	protein coding
Srebf1-207	11	18.17	1.31	0.19	ENSMUST00000144942	protein coding
St18-207	1	0.04	-5.59	NaN	ENSMUST00000139838	protein coding
St18-212	1	6.35E-03	1.34	NaN	ENSMUST00000151281	protein coding
Stat2-201	10	12.45	-1.12	0.68	ENSMUST00000085708	protein coding
Stat3-201	11	6.31	1.37	0.3	ENSMUST00000092671	protein coding
Stat3-203	11	77.73	1.12	0.14	ENSMUST00000127638	protein coding
Stat6-201	10	31.47	1.47	1.12E-03	ENSMUST00000092074	protein coding
Tal2-201	4	0.13	-1.26	0.88	ENSMUST00000030124	protein coding
Tbp-201	17	30.29	-1.44	0.09	ENSMUST00000014911	protein coding
Tbp-202	17	1.79	1.73	0.45	ENSMUST00000117593	protein coding
Tbp-203	17	12.89	-1.07	0.82	ENSMUST00000118001	protein coding
Tbp-205	17	3.46	-1.41	0.69	ENSMUST00000143924	protein coding
Tbp-206	17	1.27	1.59	0.71	ENSMUST00000147081	protein coding
Tbp-209	17	1.82	-2.29	0.39	ENSMUST00000155051	protein coding
Tbp-210	17	0.79	-10.83	0.09	ENSMUST00000159197	protein coding
Tbp-211	17	1.2	-1.19	0.57	ENSMUST00000162505	protein coding
Tbpl1-201	10	23	1.06	0.84	ENSMUST00000095794	protein coding
Tbpl1-202	10	25.98	1.54	0.02	ENSMUST00000127698	protein coding
Tbx5-201	5	0.06	2.15	0.65	ENSMUST00000018407	protein coding
Tbx5-203	5	0.04	-5.57	NaN	ENSMUST00000202723	protein coding
Tbx6-201	7	1.05	1.5	0.63	ENSMUST00000094037	protein coding
Tbx6-202	7	0.09	-1.29	0.9	ENSMUST00000172352	protein coding
Tcf12-202	9	0.55	1.91	0.43	ENSMUST00000183404	protein coding

Tcf12-203	9	0.12	10.6	NaN	ENSMUST00000183492	protein coding
Tcf12-204	9	0.06	1.4	0.87	ENSMUST00000183594	nonsense mediated decay
Tcf12-207	9	0.23	-156.84	0.06	ENSMUST00000183918	protein coding
Tcf12-210	9	0.95	-124.76	0.08	ENSMUST00000184072	protein coding
Tcf12-211	9	0.77	3	0.45	ENSMUST00000184107	nonsense mediated decay
Tcf12-214	9	0.26	-3.13	0.55	ENSMUST00000184416	nonsense mediated decay
Tcf12-216	9	1.45	-2	0.59	ENSMUST00000184523	protein coding
Tcf12-218	9	1.01	1.96	0.51	ENSMUST00000184783	protein coding
Tcf12-220	9	0.12	7.12	0.22	ENSMUST00000184867	nonsense mediated decay
Tcf12-221	9	12.65	-1.8	0.01	ENSMUST00000185117	protein coding
Tcf20-201	15	80.06	1.45	2.35E-07	ENSMUST00000048966	protein coding
Tcf20-202	15	16.59	1.08	0.71	ENSMUST00000109510	protein coding
Tcf20-204	15	0.67	7.55	0.15	ENSMUST00000229547	protein coding
Tcf21-201	10	0.05	1	1	ENSMUST00000049930	protein coding
Tcf3-201	10	9.94	-1.44	0.2	ENSMUST00000020377	protein coding
Tcf3-203	10	0.24	65.54	0.07	ENSMUST00000105339	protein coding
Tcf3-204	10	4.57	-1.23	0.71	ENSMUST00000105340	protein coding
Tcf3-209	10	0.99	-1.04	1	ENSMUST00000105345	protein coding
Tcf3-213	10	0.2	-3.14	0.53	ENSMUST00000156244	protein coding
Tcf7l2-201	19	2.11	1.4	0.45	ENSMUST00000041717	protein coding
Tcf7l2-202	19	1.08	2.49	0.36	ENSMUST00000061496	protein coding
Tcf7l2-203	19	0.07	1.18	0.94	ENSMUST00000111646	protein coding
Tcf7l2-209	19	3.3	1.97	0.21	ENSMUST00000111656	protein coding
Tcf7l2-210	19	0.44	2.34	0.49	ENSMUST00000111657	protein coding
Tcf7l2-212	19	0.2	-1.68	0.78	ENSMUST00000111659	protein coding
Tcf7l2-221	19	2.35	2.22	0.27	ENSMUST00000153888	protein coding

Tead1-202	7	66.5	1.63	7.78E-12	ENSMUST00000069256	protein coding
Tead1-206	7	0.02	4.26	NaN	ENSMUST00000164363	protein coding
Tead1-208	7	2.81	-1.83	0.41	ENSMUST00000167060	protein coding
Tead1-210	7	0.53	-1.13	0.94	ENSMUST00000168981	protein coding
Tead1-213	7	0.13	1.11	0.95	ENSMUST00000171373	protein coding
Tead2-201	7	190.14	1.95	4.69E-16	ENSMUST00000033060	protein coding
Tead2-205	7	0.12	10.51	NaN	ENSMUST00000209343	protein coding
Tead2-207	7	0.53	-33.8	0.21	ENSMUST00000209478	protein coding
Tef-202	15	7.29	1.37	0.22	ENSMUST00000109553	protein coding
Tef-206	15	0.43	29.72	0.13	ENSMUST00000168200	protein coding
Tef-210	15	1.39	1.65	0.73	ENSMUST00000172208	protein coding
Terf2-201	8	0.72	1.89	0.51	ENSMUST00000068388	protein coding
Terf2-202	8	5.79	1.09	0.85	ENSMUST00000068421	protein coding
Terf2-204	8	9.69	-1.69	0.04	ENSMUST00000133925	protein coding
Tfap2a-201	13	0.36	-2.34	0.43	ENSMUST00000021787	protein coding
Tfap2a-202	13	1.83	2.11	0.31	ENSMUST00000110193	protein coding
Tfap2a-203	13	0.21	1.13	0.96	ENSMUST00000223869	protein coding
Tfap2a-209	13	0.36	3.67	0.4	ENSMUST00000225180	protein coding
Tfap2d-201	1	0.03	-10.55	0.41	ENSMUST00000037294	protein coding
Tfap4-201	16	12	-1.51	0.04	ENSMUST00000005862	protein coding
Tfap4-203	16	0.78	-97.26	0.09	ENSMUST00000230875	protein coding
Tfcp2-201	15	0.49	-4.19	0.38	ENSMUST00000009877	protein coding
Tfcp2-202	15	0.61	-5.91	0.22	ENSMUST00000229265	protein coding
Tfcp2-204	15	6.65	-1.49	0.09	ENSMUST00000229696	protein coding
Tfcp2-208	15	0.24	14.15	0.25	ENSMUST00000231138	protein coding
Tfdp1-202	8	12.34	1.03	0.96	ENSMUST00000209282	protein coding

Tfdp1-203	8	31.53	-1.85	1.56E-03	ENSMUST00000209396	protein coding
Tfdp1-204	8	98.01	1.73	2.25E-15	ENSMUST00000209885	protein coding
Tfdp1-206	8	3.36	-1.06	0.96	ENSMUST00000210165	protein coding
Tfdp1-207	8	13.45	-1.49	0.07	ENSMUST00000210501	nonsense mediated decay
Tfdp2-201	9	17.78	-1.16	0.24	ENSMUST00000034982	protein coding
Tfdp2-202	9	0.29	-1.52	0.83	ENSMUST00000165120	protein coding
Tfdp2-203	9	7.69	-1.45	0.25	ENSMUST00000165768	protein coding
Tfdp2-205	9	0.15	-57.08	0.15	ENSMUST00000179416	protein coding
Tfdp2-206	9	2.09	1.13	0.89	ENSMUST00000185644	protein coding
Tfdp2-207	9	0.6	3.27	0.35	ENSMUST00000186609	protein coding
Tfdp2-208	9	9.1	1.84	0.18	ENSMUST00000188008	protein coding
Tfdp2-209	9	10.81	1.72	0.06	ENSMUST00000188750	protein coding
Tfdp2-210	9	0.15	-1.06	0.98	ENSMUST00000188829	protein coding
Tfdp2-215	9	1.13	-1.47	0.78	ENSMUST00000191133	protein coding
Tfe3-203	X	1.69	-1.91	0.49	ENSMUST00000101695	protein coding
Tfe3-204	X	40.81	-1.33	0.02	ENSMUST00000115677	protein coding
Tfe3-205	X	14.01	-1.96	9.22E-03	ENSMUST00000115678	protein coding
Tfe3-206	X	8.05	1.49	0.09	ENSMUST00000115679	protein coding
Tfe3-214	X	4.51	-1.18	0.87	ENSMUST00000144900	protein coding
Tfeb-202	17	8	1.65	0.14	ENSMUST00000086932	protein coding
Tfeb-203	17	0.4	-3.51	0.3	ENSMUST00000113284	protein coding
Tfeb-206	17	0.03	-3.31	NaN	ENSMUST00000125177	protein coding
Tfeb-208	17	0.17	-10.11	0.4	ENSMUST00000130208	protein coding
Tfeb-211	17	0.96	53.92	0.07	ENSMUST00000141631	protein coding
Tfeb-212	17	10.55	-1.67	0.14	ENSMUST00000146782	protein coding
Tfeb-213	17	0.03	-3.31	NaN	ENSMUST00000159641	protein coding

Tfeb-214	17	0.15	7.56	NaN	ENSMUST00000160373	protein coding
Thap1-201	8	7.61	-1.32	0.29	ENSMUST00000036807	protein coding
Thap1-203	8	6.08	-1.62	0.45	ENSMUST00000130231	protein coding
Thap11-201	8	69.02	1.39	4.41E-03	ENSMUST00000040445	protein coding
Thap12-201	7	39.19	-1.18	0.1	ENSMUST00000033009	protein coding
Thap12-202	7	2.32	1.68	0.56	ENSMUST00000126356	nonsense mediated decay
Thap12-204	7	1.83	-2.46	0.25	ENSMUST00000153566	nonsense mediated decay
Thap4-201	1	36.13	-1.17	0.24	ENSMUST00000112905	nonsense mediated decay
Thap4-202	1	0.61	1.26	0.89	ENSMUST00000187445	nonsense mediated decay
Thap4-204	1	12.29	1.63	0.39	ENSMUST00000189472	protein coding
Thap4-206	1	2.76	1.94	0.12	ENSMUST00000190116	protein coding
Thap7-202	16	3.49	2.05	0.43	ENSMUST00000231288	protein coding
Thap7-203	16	0.79	10.22	0.12	ENSMUST00000231424	nonsense mediated decay
Thap7-205	16	8.2	-1.85	0.21	ENSMUST00000231548	protein coding
Thap7-209	16	4.22	3.02	0.27	ENSMUST00000232041	protein coding
Thra-201	11	6.45	2.06	0.07	ENSMUST00000064187	protein coding
Thra-202	11	5.6	1.97	0.2	ENSMUST00000103139	protein coding
Thra-203	11	2.21	-4.42	0.06	ENSMUST00000124072	protein coding
Thra-208	11	0.23	-1.08	0.97	ENSMUST00000153043	protein coding
Thrb-201	14	0.06	-3.06	0.46	ENSMUST00000022303	protein coding
Thrb-202	14	0.02	1.72	0.78	ENSMUST00000022304	protein coding
Thrb-203	14	0.03	-10.18	0.42	ENSMUST00000091471	protein coding
Thrb-207	14	0.13	-2.29	0.67	ENSMUST00000224934	protein coding
Tigd2-201	6	6.84	-1.11	0.71	ENSMUST00000062626	protein coding
Tigd4-201	3	6.72E-03	1.34	NaN	ENSMUST00000062623	protein coding
Tigd5-201	15	0.96	-1.41	0.62	ENSMUST00000192937	protein coding

Tlx2-201	6	0.06	-1.58	0.81	ENSMUST00000089641	protein coding
Tlx2-202	6	0.12	4.13	0.38	ENSMUST00000174674	protein coding
Tmf1-201	6	9.37	1.29	0.36	ENSMUST00000095664	protein coding
Tmf1-202	6	17.32	-1.08	0.8	ENSMUST00000124173	nonsense mediated decay
Trp63-204	16	0.02	-1.76	0.79	ENSMUST00000115305	protein coding
Trp63-205	16	6.56E-03	-5.59	NaN	ENSMUST00000115306	protein coding
Trp73-202	4	0.12	4.17	0.41	ENSMUST00000097763	nonsense mediated decay
Trp73-203	4	0.04	-11.79	0.39	ENSMUST00000105643	protein coding
Trp73-204	4	0.27	6.15	0.19	ENSMUST00000105644	protein coding
Trp73-207	4	0.13	-15.96	0.33	ENSMUST00000155642	protein coding
Trp73-208	4	0.23	5.15	0.27	ENSMUST00000239448	protein coding
Tshz2-201	2	0.13	2.28	0.57	ENSMUST00000109157	protein coding
Tshz2-202	2	0.11	-2.9	0.44	ENSMUST00000109159	protein coding
Ttf1-201	2	23.9	1.13	0.38	ENSMUST00000100237	protein coding
Ubp1-201	9	46.1	-1.65	2.15E-06	ENSMUST00000009885	protein coding
Usf2-201	7	31.62	1.84	4.07E-03	ENSMUST00000058860	protein coding
Usf2-203	7	0.36	46.53	0.11	ENSMUST00000162228	protein coding
Usf2-204	7	0.66	-1.84	0.73	ENSMUST00000166340	nonsense mediated decay
Usf2-208	7	0.29	35.19	0.11	ENSMUST00000170442	protein coding
Usf2-211	7	0.38	33.6	0.14	ENSMUST00000172417	protein coding
Usf3-201	16	0.9	-1.1	0.95	ENSMUST00000088356	protein coding
Usf3-202	16	2.86	-1.16	0.66	ENSMUST00000119746	protein coding
Vax1-201	19	0.14	20.74	0.17	ENSMUST00000172821	protein coding
Vsx1-201	2	0.02	3.41	0.46	ENSMUST00000046095	protein coding
Wiz-203	17	3.12	-1.4	0.4	ENSMUST00000087703	protein coding
Wiz-205	17	0.07	-9.72	0.42	ENSMUST00000165912	protein coding

Wiz-207	17	6.69	1.35	0.33	ENSMUST00000169488	protein coding
Wiz-209	17	0.48	33.93	0.1	ENSMUST00000170603	protein coding
Wiz-210	17	0.71	-1.49	0.58	ENSMUST00000170617	nonsense mediated decay
Wiz-211	17	0.21	3.14	0.39	ENSMUST00000171728	nonsense mediated decay
Wiz-213	17	0.2	2.77	0.5	ENSMUST00000236409	protein coding
Ybx2-205	11	8.15	1.91	0.23	ENSMUST00000149194	protein coding
Ybx3-201	6	193.73	-1.46	1.40E-05	ENSMUST00000032309	protein coding
Ybx3-202	6	150.78	-1.89	9.03E-21	ENSMUST00000087865	protein coding
Yy1-201	12	42.87	1.05	0.6	ENSMUST00000021692	protein coding
Zbed3-201	13	16.58	-1.02	0.95	ENSMUST00000045909	protein coding
Zbed3-203	13	0.41	-4.22	0.38	ENSMUST00000222456	protein coding
Zbed6-201	1	63.51	1.27	0.01	ENSMUST00000179598	protein coding
Zbtb1-201	12	4.37	-1.63	0.02	ENSMUST00000042779	protein coding
Zbtb11-201	16	21.75	-1.4	3.93E-03	ENSMUST00000050248	protein coding
Zbtb14-201	17	12.56	-1.05	0.89	ENSMUST00000062369	protein coding
Zbtb17-201	4	21.59	1.19	0.48	ENSMUST00000006377	protein coding
Zbtb18-201	1	1.88	-1.58	0.45	ENSMUST00000077225	protein coding
Zbtb18-202	1	3.25	-1.34	0.41	ENSMUST00000094276	protein coding
Zbtb18-203	1	0.14	-5.57	NaN	ENSMUST00000192699	protein coding
Zbtb18-204	1	0.27	-37.51	0.21	ENSMUST00000192851	protein coding
Zbtb18-206	1	5.27	-1.18	0.53	ENSMUST00000193480	protein coding
Zbtb18-208	1	1.47	-5.3	0.21	ENSMUST00000194319	protein coding
Zbtb18-209	1	0.83	7.55	0.18	ENSMUST00000195002	protein coding
Zbtb18-210	1	0.19	-15.96	0.33	ENSMUST00000195388	protein coding
Zbtb18-211	1	0.97	-2.08	0.6	ENSMUST00000195549	protein coding
Zbtb2-201	10	11.8	-1.91	4.07E-03	ENSMUST00000100077	protein coding

Zbtb21-201	16	16.3	1.24	0.37	ENSMUST00000052089	protein coding
Zbtb21-206	16	0.86	1.97	0.65	ENSMUST00000231560	protein coding
Zbtb22-201	17	21.71	1.82	2.67E-03	ENSMUST00000053429	protein coding
Zbtb22-202	17	2.03	1.36	0.83	ENSMUST00000174463	protein coding
Zbtb24-201	10	10.41	-1.21	0.45	ENSMUST00000080771	protein coding
Zbtb24-202	10	0.15	52.99	0.08	ENSMUST00000213797	protein coding
Zbtb24-205	10	3.11	-1.84	0.2	ENSMUST00000216656	protein coding
Zbtb25-203	12	0.21	-17.06	0.32	ENSMUST00000176187	protein coding
Zbtb25-204	12	7.95	1.09	0.86	ENSMUST00000176278	protein coding
Zbtb25-205	12	2.56	-2.22	0.23	ENSMUST00000176509	protein coding
Zbtb26-201	2	5.55	1.34	0.25	ENSMUST00000067043	protein coding
Zbtb26-202	2	3.73	-2.21	0.08	ENSMUST00000102789	protein coding
Zbtb32-201	7	3.32	1.32	0.76	ENSMUST00000108150	protein coding
Zbtb32-202	7	15.85	-1.57	0.05	ENSMUST00000108151	protein coding
Zbtb33-201	X	8.16	1.62	0.04	ENSMUST00000049740	protein coding
Zbtb34-201	2	17.62	-1.88	8.85E-06	ENSMUST00000091037	protein coding
Zbtb37-201	1	3.08	-1.92	0.24	ENSMUST00000159250	protein coding
Zbtb37-202	1	0.26	2.03	0.68	ENSMUST00000160056	nonsense mediated decay
Zbtb37-203	1	5.23	1.42	8.31E-03	ENSMUST00000162226	protein coding
Zbtb37-204	1	0.54	2.48	0.5	ENSMUST00000163079	nonsense mediated decay
Zbtb37-205	1	0.3	3.54	0.36	ENSMUST00000171748	protein coding
Zbtb37-207	1	0.29	-1.4	0.86	ENSMUST00000177003	protein coding
Zbtb38-201	9	0.92	-2.36	0.36	ENSMUST00000093798	protein coding
Zbtb38-203	9	1.77	1.23	0.78	ENSMUST00000126066	non_stop_decay
Zbtb38-204	9	0.99	-3.16	0.36	ENSMUST00000128269	protein coding
Zbtb38-208	9	0.39	7.17	0.11	ENSMUST00000140121	protein coding

Zbtb38-212	9	6.46	1.04	0.91	ENSMUST00000152594	protein coding
Zbtb39-201	10	15.62	1.28	0.03	ENSMUST00000054287	protein coding
Zbtb40-201	4	6.62	-1.21	0.38	ENSMUST00000049583	protein coding
Zbtb40-203	4	1.54	-1.28	0.81	ENSMUST00000218160	protein coding
Zbtb41-201	1	4.21	1.51	0.11	ENSMUST00000039867	protein coding
Zbtb41-202	1	6.27	-1.94	2.15E-03	ENSMUST00000199011	nonsense mediated decay
Zbtb41-203	1	6.56	-1.72	0.06	ENSMUST00000200243	protein coding
Zbtb43-201	2	11.37	-1.03	0.92	ENSMUST00000028125	protein coding
Zbtb43-203	2	4.69	-2.51	0.05	ENSMUST00000113156	protein coding
Zbtb43-204	2	2.62	-1.11	0.92	ENSMUST00000126442	protein coding
Zbtb43-205	2	1.17	-125.15	0.07	ENSMUST00000155198	protein coding
Zbtb44-203	9	0.31	-16.38	0.32	ENSMUST00000213202	nonsense mediated decay
Zbtb44-204	9	0.06	16.72	0.24	ENSMUST00000214585	protein coding
Zbtb48-201	4	11.61	-1.08	0.82	ENSMUST00000066715	protein coding
Zbtb48-203	4	1.03	2.45	0.57	ENSMUST00000131935	protein coding
Zbtb48-206	4	1.05	1.07	0.95	ENSMUST00000155389	nonsense mediated decay
Zbtb48-208	4	2.62	-1.21	0.84	ENSMUST00000156748	nonsense mediated decay
Zbtb49-201	5	1.8	2.27	0.27	ENSMUST00000094833	protein coding
Zbtb49-202	5	0.77	-1.12	0.91	ENSMUST00000114113	protein coding
Zbtb49-203	5	0.13	-2.29	0.67	ENSMUST00000123106	nonsense mediated decay
Zbtb49-205	5	1	-1.38	0.78	ENSMUST00000126267	nonsense mediated decay
Zbtb49-206	5	1.12	-1.35	0.8	ENSMUST00000129161	nonsense mediated decay
Zbtb49-207	5	6.12E-03	-3.31	NaN	ENSMUST00000136475	nonsense mediated decay
Zbtb49-209	5	0.31	-97.26	0.1	ENSMUST00000138820	nonsense mediated decay
Zbtb49-210	5	1.27	2.05	0.33	ENSMUST00000143436	nonsense mediated decay
Zbtb49-211	5	1.26	8.07	0.14	ENSMUST00000146859	protein coding

Zbtb5-201	4	4.55	-1.15	0.61	ENSMUST00000055028	protein coding
Zbtb5-202	4	2.39	2.08	0.09	ENSMUST00000107817	protein coding
Zbtb6-201	2	10.44	1.62	0.04	ENSMUST00000053098	protein coding
Zbtb7a-201	10	6.58	-1.53	0.03	ENSMUST00000048128	protein coding
Zbtb7a-203	10	0.48	-6.66	0.24	ENSMUST00000119606	protein coding
Zbtb7a-204	10	0.06	-16.95	0.31	ENSMUST00000121840	protein coding
Zbtb7a-205	10	0.3	38.75	0.11	ENSMUST00000125261	protein coding
Zbtb7c-201	18	1.79	-1.35	0.61	ENSMUST00000058997	protein coding
Zbtb7c-203	18	0.03	16.25	0.24	ENSMUST00000236462	nonsense mediated decay
Zbtb8b-201	4	5.09	-1.89	0.07	ENSMUST00000053042	protein coding
Zbtb8b-202	4	0.84	11.84	0.32	ENSMUST00000106046	protein coding
Zbtb9-201	17	14.28	-1.15	0.58	ENSMUST00000120016	protein coding
Zbtb9-202	17	0.65	-2.43	0.4	ENSMUST00000237633	nonsense mediated decay
Zfand5-201	19	38.15	-1.03	0.86	ENSMUST00000025659	protein coding
Zfand5-204	19	7.17	1.24	0.79	ENSMUST00000149144	nonsense mediated decay
Zfat-202	15	1.89	1.42	0.46	ENSMUST00000160248	protein coding
Zfat-204	15	7.5	1.77	5.30E-03	ENSMUST00000162054	protein coding
Zfat-205	15	0.02	-13.87	0.36	ENSMUST00000162173	protein coding
Zfp1-201	8	13.49	-1.35	0.25	ENSMUST00000077791	protein coding
Zfp1-202	8	9.78	-1.9	0.11	ENSMUST00000211926	protein coding
Zfp1-203	8	5.74	1.91	0.29	ENSMUST00000212072	protein coding
Zfp1-204	8	0.83	-1.2	0.83	ENSMUST00000212206	nonsense mediated decay
Zfp110-201	7	28.38	-1.22	0.15	ENSMUST00000004614	protein coding
Zfp111-201	7	8.43	-1.44	0.15	ENSMUST00000056683	protein coding
Zfp111-202	7	0.08	-36.85	0.21	ENSMUST00000073833	protein coding
Zfp113-201	5	3.06	1.26	0.37	ENSMUST00000049393	protein coding

Zfp113-202	5	0.39	3.26	0.45	ENSMUST00000132318	protein coding
Zfp113-204	5	1.9	-1.25	0.8	ENSMUST00000165640	protein coding
Zfp12-202	5	9.63	1.06	0.85	ENSMUST00000075916	nonsense mediated decay
Zfp12-205	5	1.16	2.27	0.53	ENSMUST00000162861	protein coding
Zfp128-202	7	0.91	1.11	0.88	ENSMUST00000144578	protein coding
Zfp131-202	13	13.13	1.66	0.09	ENSMUST00000178271	protein coding
Zfp131-204	13	1.39	-7.79	0.11	ENSMUST00000223797	protein coding
Zfp131-205	13	2.01	-1.58	0.63	ENSMUST00000223812	protein coding
Zfp131-207	13	2.56	1.57	0.49	ENSMUST00000224946	protein coding
Zfp131-209	13	0.18	-2.32	0.65	ENSMUST00000225484	nonsense mediated decay
Zfp142-201	1	13.38	-1.44	2.06E-03	ENSMUST00000027315	protein coding
Zfp142-203	1	1.42	-1.57	0.39	ENSMUST00000113737	protein coding
Zfp142-208	1	0.53	-1.1	0.91	ENSMUST00000156613	nonsense mediated decay
Zfp143-201	7	15.19	-1	1	ENSMUST00000084727	protein coding
Zfp143-202	7	0.34	-1.53	0.7	ENSMUST00000169638	protein coding
Zfp143-204	7	0.2	-2.12	0.67	ENSMUST00000209505	protein coding
Zfp143-205	7	19.6	-1.82	0.01	ENSMUST00000211798	protein coding
Zfp146-201	7	43.07	-1.46	3.64E-04	ENSMUST00000062181	protein coding
Zfp148-201	16	10.23	-1.26	0.32	ENSMUST00000089677	protein coding
Zfp148-202	16	5.76	1.28	0.23	ENSMUST00000165418	protein coding
Zfp148-205	16	7.67	-1.34	0.28	ENSMUST00000232023	protein coding
Zfp160-201	17	5.81	-1.21	0.47	ENSMUST00000088811	protein coding
Zfp160-204	17	0.22	1.18	0.94	ENSMUST00000232320	protein coding
Zfp160-206	17	2.62	-1.91	0.32	ENSMUST00000232473	protein coding
Zfp160-207	17	2.13	1.13	0.89	ENSMUST00000232595	protein coding
Zfp160-208	17	0.6	-4.84	0.31	ENSMUST00000232663	protein coding

Zfp180-201	7	14.09	-1.34	0.13	ENSMUST00000068975	protein coding
Zfp180-203	7	1.55	1.28	0.82	ENSMUST00000206184	nonsense mediated decay
Zfp180-205	7	0.12	4.11	0.39	ENSMUST00000207002	protein coding
Zfp182-203	X	1.95	-1.07	0.91	ENSMUST00000115334	protein coding
Zfp189-201	4	0.23	15.92	0.27	ENSMUST00000042964	protein coding
Zfp189-202	4	5.78	-1.48	0.13	ENSMUST00000107696	protein coding
Zfp2-201	11	2.52	1.63	0.39	ENSMUST00000109128	protein coding
Zfp2-203	11	5.87	-1.36	0.26	ENSMUST00000116378	protein coding
Zfp207-201	11	163.72	-1.24	7.04E-03	ENSMUST00000017567	protein coding
Zfp207-205	11	0.33	-1.26	0.9	ENSMUST00000153824	protein coding
Zfp207-206	11	7.03	1.19	0.66	ENSMUST00000165565	protein coding
Zfp212-201	6	16.26	-1.21	0.49	ENSMUST00000009411	protein coding
Zfp213-201	17	6.22	1.29	0.45	ENSMUST00000095606	protein coding
Zfp219-202	14	2.56	-1.47	0.71	ENSMUST00000166169	protein coding
Zfp219-205	14	2.55	-1.78	0.45	ENSMUST00000226527	protein coding
Zfp219-206	14	0.39	-30.46	0.23	ENSMUST00000226554	protein coding
Zfp219-207	14	1.08	-1.42	0.8	ENSMUST00000226605	protein coding
Zfp219-208	14	2.05	1.99	0.47	ENSMUST00000226964	protein coding
Zfp219-210	14	0.05	4.26	NaN	ENSMUST00000228051	protein coding
Zfp219-211	14	0.28	-4.91	0.38	ENSMUST00000228162	protein coding
Zfp219-212	14	9.24	-1.09	0.82	ENSMUST00000228580	protein coding
Zfp219-213	14	2.9	-2.14	0.34	ENSMUST00000228747	protein coding
Zfp236-201	18	3.07	1.2	0.51	ENSMUST00000171071	protein coding
Zfp236-202	18	5.72	1.19	0.5	ENSMUST00000182122	protein coding
Zfp236-204	18	0.79	2.6	0.38	ENSMUST00000182866	protein coding
Zfp24-201	18	11.64	-1.83	6.52E-04	ENSMUST00000066497	protein coding

Zfp24-202	18	3.56	-1.45	0.67	ENSMUST00000148525	protein coding
Zfp24-203	18	21.34	-1.92	3.86E-03	ENSMUST00000153337	protein coding
Zfp248-201	6	4.48	1.01	0.99	ENSMUST00000069292	protein coding
Zfp248-203	6	0.65	-1.97	0.63	ENSMUST00000159403	protein coding
Zfp248-205	6	4.16	-2.32	0.08	ENSMUST00000161519	protein coding
Zfp251-201	15	10.45	1.18	0.52	ENSMUST00000080406	protein coding
Zfp251-202	15	1.16	4.46	0.06	ENSMUST00000229494	protein coding
Zfp263-201	16	14.98	1.28	0.33	ENSMUST00000023176	protein coding
Zfp275-202	X	8.2	1.34	0.21	ENSMUST00000114499	protein coding
Zfp277-201	12	37.12	-1.63	3.09E-04	ENSMUST00000069637	protein coding
Zfp277-202	12	0.71	-1.15	0.91	ENSMUST00000069692	protein coding
Zfp280b-201	10	57.61	-1.5	2.61E-10	ENSMUST00000061617	protein coding
Zfp280b-202	10	2.76	1.58	0.65	ENSMUST00000218627	protein coding
Zfp280c-201	X	11.05	-1.38	0.18	ENSMUST00000072292	protein coding
Zfp280c-204	X	5.6	-1.37	0.25	ENSMUST00000114940	protein coding
Zfp280d-203	9	8.5	-1.76	0.1	ENSMUST00000183410	protein coding
Zfp280d-208	9	0.64	-1.72	0.49	ENSMUST00000183801	nonsense mediated decay
Zfp280d-212	9	0.41	2.13	0.36	ENSMUST00000184053	nonsense mediated decay
Zfp280d-214	9	4.31	-1.77	0.45	ENSMUST00000184216	protein coding
Zfp280d-217	9	0.93	3.14	0.12	ENSMUST00000184517	protein coding
Zfp280d-219	9	1.36	-1.65	0.65	ENSMUST00000184786	nonsense mediated decay
Zfp280d-221	9	1.27	-1.01	1	ENSMUST00000185020	nonsense mediated decay
Zfp282-201	6	6.69	1.87	6.72E-03	ENSMUST00000061890	protein coding
Zfp287-201	11	0.82	-1.22	0.82	ENSMUST00000005399	protein coding
Zfp287-203	11	0.47	2.09	0.55	ENSMUST00000128370	protein coding
Zfp287-204	11	9.94E-03	1.34	NaN	ENSMUST00000149228	nonsense mediated decay

Zfp287-205	11	0.52	3.05	0.35	ENSMUST00000150336	protein coding
Zfp287-206	11	1.16	-1.31	0.73	ENSMUST00000185656	protein coding
Zfp292-201	4	19.87	-1.18	0.09	ENSMUST00000047950	protein coding
Zfp292-202	4	0.48	-49.51	0.13	ENSMUST00000098163	protein coding
Zfp3-201	11	4.72	1.32	0.66	ENSMUST00000060444	protein coding
Zfp30-201	7	3.63	1.6	0.34	ENSMUST00000032803	protein coding
Zfp30-202	7	0.05	1.3	0.9	ENSMUST00000122387	protein coding
Zfp318-201	17	8.21	-1.18	0.23	ENSMUST00000113481	protein coding
Zfp318-204	17	4.65	-1.29	0.24	ENSMUST00000138127	protein coding
Zfp318-205	17	1.65	-6.36	0.09	ENSMUST00000152472	nonsense mediated decay
Zfp324-201	7	2.18	-1.09	0.88	ENSMUST00000038701	protein coding
Zfp324-202	7	0.38	1.97	0.45	ENSMUST00000124387	nonsense mediated decay
Zfp326-201	5	22.46	1.48	0.07	ENSMUST00000031227	protein coding
Zfp326-209	5	13.18	-1.55	0.09	ENSMUST00000150440	protein coding
Zfp329-201	7	5.09	1.13	0.68	ENSMUST00000072222	protein coding
Zfp329-202	7	0.42	2.02	0.55	ENSMUST00000108546	protein coding
Zfp329-203	7	3.15	-2.15	0.09	ENSMUST00000121215	nonsense mediated decay
Zfp335-201	2	21.61	1.82	4.54E-05	ENSMUST00000041361	protein coding
Zfp341-201	2	6.19	1.35	0.26	ENSMUST00000081926	protein coding
Zfp341-203	2	0.23	-110.68	0.08	ENSMUST00000126421	nonsense mediated decay
Zfp346-201	13	26.68	1.16	0.24	ENSMUST00000021937	protein coding
Zfp346-202	13	0.23	37.21	0.1	ENSMUST00000159147	protein coding
Zfp346-205	13	0.3	-31.98	0.22	ENSMUST00000160660	nonsense mediated decay
Zfp346-207	13	5.63	-1.32	0.73	ENSMUST00000161077	protein coding
Zfp346-208	13	0.25	-2.75	0.6	ENSMUST00000161315	protein coding
Zfp346-209	13	0.04	4.26	NaN	ENSMUST00000161551	nonsense mediated decay

Zfp346-211	13	0.94	-1.21	0.86	ENSMUST00000162428	nonsense mediated decay
Zfp354a-202	11	4.93	1.19	0.73	ENSMUST00000102766	protein coding
Zfp354a-203	11	1.86	1.22	0.78	ENSMUST00000109119	protein coding
Zfp354a-204	11	1.18	1.05	0.95	ENSMUST00000109122	protein coding
Zfp354b-201	11	0.95	2.54	0.22	ENSMUST00000109124	protein coding
Zfp354c-201	11	4.13	-2.71	0.09	ENSMUST00000000632	protein coding
Zfp354c-202	11	5.87	-1.54	0.06	ENSMUST00000109135	protein coding
Zfp365-201	10	7.03	1.02	0.94	ENSMUST00000064656	protein coding
Zfp366-201	13	0.01	1.45	0.83	ENSMUST00000056558	protein coding
Zfp367-201	13	10.47	-1.35	0.17	ENSMUST00000059817	protein coding
Zfp367-202	13	1.04	1.47	0.67	ENSMUST00000117241	protein coding
Zfp369-202	13	13.07	-1.42	0.1	ENSMUST00000126879	protein coding
Zfp369-203	13	6.94	1.07	0.91	ENSMUST00000130799	protein coding
Zfp369-204	13	4.18	1.37	0.7	ENSMUST00000155732	protein coding
Zfp37-201	4	7.23	1.86	4.04E-04	ENSMUST00000068822	protein coding
Zfp37-202	4	1.2	-1.04	1	ENSMUST00000129511	protein coding
Zfp37-203	4	0.68	8.78	0.14	ENSMUST00000212325	protein coding
Zfp37-206	4	4.68	1.19	0.83	ENSMUST00000222050	protein coding
Zfp37-207	4	1.79	-2.91	0.27	ENSMUST00000222748	protein coding
Zfp383-201	7	9.31	-1.43	0.15	ENSMUST00000186475	protein coding
Zfp383-202	7	1.22	-1.38	0.69	ENSMUST00000187028	nonsense mediated decay
Zfp384-201	6	8.03	1.29	0.4	ENSMUST00000046064	protein coding
Zfp384-202	6	6.08	1.56	0.27	ENSMUST00000054553	protein coding
Zfp384-203	6	15.91	-1.95	2.80E-03	ENSMUST00000084275	protein coding
Zfp384-204	6	0.01	-5.59	NaN	ENSMUST00000088308	protein coding
Zfp384-207	6	7.57E-03	4.26	NaN	ENSMUST00000112427	protein coding

Zfp384-208	6	2.32	3.79	0.15	ENSMUST00000112428	protein coding
Zfp384-216	6	1.19	-3.01	0.35	ENSMUST00000152752	protein coding
Zfp384-217	6	2.18	2.74	0.27	ENSMUST00000249621	protein coding
Zfp385a-201	15	12.52	-1.04	0.91	ENSMUST00000168828	protein coding
Zfp385b-201	2	0.14	52.97	0.08	ENSMUST00000090766	protein coding
Zfp385b-202	2	0.01	4.26	NaN	ENSMUST00000111829	protein coding
Zfp385b-203	2	0.07	-1.38	0.86	ENSMUST00000111830	protein coding
Zfp385b-204	2	0.02	-10.18	0.42	ENSMUST00000111831	protein coding
Zfp39-201	11	7.43	1.34	0.23	ENSMUST00000102703	protein coding
Zfp395-201	14	16.39	1.09	0.71	ENSMUST00000066994	protein coding
Zfp395-202	14	0.1	1.34	NaN	ENSMUST00000224623	protein coding
Zfp397-201	18	8.05	-1.03	0.93	ENSMUST00000060762	protein coding
Zfp398-201	6	2.02	-1.47	0.51	ENSMUST00000079881	protein coding
Zfp398-202	6	1.19	-1.66	0.35	ENSMUST00000114598	protein coding
Zfp407-201	18	0.17	-2.08	0.71	ENSMUST00000125450	protein coding
Zfp407-202	18	8.42	-1.04	0.87	ENSMUST00000125763	protein coding
Zfp408-201	2	4.04	1.03	0.95	ENSMUST00000099714	protein coding
Zfp408-202	2	8	1.48	0.11	ENSMUST00000111333	protein coding
Zfp408-204	2	0.68	-1.04	0.98	ENSMUST00000145582	nonsense mediated decay
Zfp41-201	15	1.48	-1.17	0.64	ENSMUST00000054555	protein coding
Zfp41-203	15	0.09	1.33	0.89	ENSMUST00000161752	protein coding
Zfp41-204	15	13.93	-1.54	0.06	ENSMUST00000161785	protein coding
Zfp410-203	12	1.02	1.34	0.73	ENSMUST00000220931	protein coding
Zfp410-208	12	0.9	1.19	0.87	ENSMUST00000222258	protein coding
Zfp410-210	12	0.18	-13.87	0.36	ENSMUST00000222450	protein coding
Zfp410-211	12	4.59	-3.49	0.12	ENSMUST00000222471	protein coding

Zfp410-212	12	1.29	-1.26	0.83	ENSMUST00000222506	nonsense mediated decay
Zfp410-215	12	1	1.01	1	ENSMUST00000222832	protein coding
Zfp414-201	17	25.61	1.48	0.2	ENSMUST00000073570	protein coding
Zfp414-202	17	1.51	2.01	0.59	ENSMUST00000165504	protein coding
Zfp414-203	17	5.91	-1.41	0.52	ENSMUST00000166627	protein coding
Zfp420-201	7	1.91	2.12	0.43	ENSMUST00000057652	protein coding
Zfp420-202	7	4.52	-2.14	0.16	ENSMUST00000074876	protein coding
Zfp426-201	9	4.85	1.23	0.73	ENSMUST00000080386	protein coding
Zfp426-203	9	2.01	1.88	0.33	ENSMUST00000163348	protein coding
Zfp426-204	9	0.46	2.21	0.61	ENSMUST00000163427	nonsense mediated decay
Zfp426-205	9	0.15	13.58	0.28	ENSMUST00000164250	protein coding
Zfp426-206	9	0.63	1.2	0.92	ENSMUST00000164799	protein coding
Zfp426-207	9	0.06	7.56	NaN	ENSMUST00000164825	protein coding
Zfp426-210	9	1.13	1.53	0.56	ENSMUST00000167457	protein coding
Zfp426-211	9	0.71	1.39	0.8	ENSMUST00000168095	nonsense mediated decay
Zfp426-213	9	1.79	-1.19	0.78	ENSMUST00000169558	protein coding
Zfp428-201	7	20.13	-1.48	0.14	ENSMUST00000071361	protein coding
Zfp428-202	7	4.48	1.84	0.32	ENSMUST00000176880	protein coding
Zfp428-204	7	0.37	24.03	0.16	ENSMUST00000177228	protein coding
Zfp444-201	7	1.99	1.73	0.45	ENSMUST00000054680	protein coding
Zfp444-202	7	0.09	-1.58	0.8	ENSMUST00000108565	protein coding
Zfp444-203	7	19.7	1.43	0.15	ENSMUST00000108566	protein coding
Zfp444-204	7	0.32	2.15	0.41	ENSMUST00000108567	protein coding
Zfp444-206	7	0.55	10.1	0.16	ENSMUST00000134933	protein coding
Zfp444-207	7	0.28	3.07	0.5	ENSMUST00000136777	protein coding
Zfp444-210	7	0.36	20.74	0.18	ENSMUST00000207176	protein coding

Zfp446-201	7	1.66	1.59	0.34	ENSMUST00000045810	protein coding
Zfp446-203	7	7.26	1.71	0.02	ENSMUST00000108536	protein coding
Zfp446-204	7	0.18	-1.15	0.94	ENSMUST00000108537	protein coding
Zfp449-201	X	4.62	-1.29	0.37	ENSMUST00000101560	protein coding
Zfp455-201	13	0.1	-42.19	0.18	ENSMUST00000117110	protein coding
Zfp455-202	13	1.29	1.9	0.41	ENSMUST00000120861	protein coding
Zfp457-201	13	0.19	1.2	0.91	ENSMUST00000049705	protein coding
Zfp457-203	13	0.1	-1.22	0.9	ENSMUST00000224325	protein coding
Zfp457-205	13	0.23	-2.14	0.7	ENSMUST00000225338	protein coding
Zfp458-201	13	0.87	-1.1	0.91	ENSMUST00000045969	protein coding
Zfp458-202	13	0.07	7.56	NaN	ENSMUST00000223990	protein coding
Zfp458-204	13	0.17	80.08	0.05	ENSMUST00000225772	protein coding
Zfp462-202	4	12.76	-1.06	0.83	ENSMUST00000079605	protein coding
Zfp462-203	4	39.39	1.27	1.15E-03	ENSMUST00000098070	protein coding
Zfp462-204	4	17.23	2.22	0.22	ENSMUST00000133895	protein coding
Zfp473-201	7	1.94	1.57	0.49	ENSMUST00000060270	protein coding
Zfp473-202	7	2.96	-1.73	0.55	ENSMUST00000118162	protein coding
Zfp473-203	7	9.46	-1.4	0.16	ENSMUST00000120074	protein coding
Zfp473-204	7	11.03	-1.57	0.05	ENSMUST00000120798	protein coding
Zfp473-206	7	4.1	1.21	0.81	ENSMUST00000140599	protein coding
Zfp473-207	7	0.39	-1.91	0.31	ENSMUST00000149011	protein coding
Zfp507-201	7	18.09	1.37	0.01	ENSMUST00000061586	protein coding
Zfp507-203	7	0.69	-1.34	0.83	ENSMUST00000187282	protein coding
Zfp507-204	7	0.37	2.04	0.72	ENSMUST00000205670	protein coding
Zfp511-201	7	31.63	1.59	0.04	ENSMUST00000168194	protein coding
Zfp511-204	7	4.9	1.82	0.2	ENSMUST00000210882	protein coding

Zfp511-206	7	0.11	13.58	0.28	ENSMUST00000211789	nonsense mediated decay
Zfp512-201	5	27.78	-1.3	0.04	ENSMUST00000076264	protein coding
Zfp512-202	5	0.34	4.06	0.36	ENSMUST00000200782	protein coding
Zfp512-203	5	3.43	-2.59	0.14	ENSMUST00000201450	protein coding
Zfp512-207	5	0.76	1.27	0.86	ENSMUST00000202061	protein coding
Zfp512-208	5	1.45	-1.03	0.99	ENSMUST00000202244	protein coding
Zfp512b-201	2	3.44	-1.19	0.53	ENSMUST00000108789	protein coding
Zfp512b-202	2	15.74	1.05	0.8	ENSMUST00000128553	protein coding
Zfp512b-204	2	0.29	1.03	0.99	ENSMUST00000132538	nonsense mediated decay
Zfp512b-207	2	0.42	1.59	0.78	ENSMUST00000140103	protein coding
Zfp512b-209	2	2.3	4.52	0.15	ENSMUST00000153998	protein coding
Zfp513-207	5	0.14	-7.65	NaN	ENSMUST00000201968	protein coding
Zfp516-201	18	13.57	-1.7	1.10E-05	ENSMUST00000071233	protein coding
Zfp516-202	18	1.03	3.41	0.07	ENSMUST00000171238	protein coding
Zfp516-204	18	1.58	-89.17	0.1	ENSMUST00000235902	protein coding
Zfp516-205	18	1.18	-2.15	0.48	ENSMUST00000236468	protein coding
Zfp516-207	18	0.64	4.93	0.27	ENSMUST00000237340	protein coding
Zfp521-201	18	0.07	1.31	0.87	ENSMUST00000025288	protein coding
Zfp521-204	18	0.09	1.47	0.83	ENSMUST00000234410	protein coding
Zfp521-205	18	0.05	-28.94	0.23	ENSMUST00000234480	nonsense mediated decay
Zfp523-201	17	1.25	2.6	0.33	ENSMUST00000002318	protein coding
Zfp523-203	17	0.81	2.32	0.5	ENSMUST00000129522	nonsense mediated decay
Zfp523-204	17	0.05	7.56	NaN	ENSMUST00000133868	protein coding
Zfp523-207	17	0.81	-2.63	0.53	ENSMUST00000155030	protein coding
Zfp523-208	17	1.01	-1.44	0.78	ENSMUST00000161275	nonsense mediated decay
Zfp523-209	17	0.15	-1.28	0.9	ENSMUST00000232845	nonsense mediated decay

Zfp523-212	17	5.65	-1.14	0.71	ENSMUST00000233613	protein coding
Zfp523-213	17	0.04	13.58	0.27	ENSMUST00000233841	nonsense mediated decay
Zfp523-214	17	0.01	-5.57	NaN	ENSMUST00000233904	protein coding
Zfp526-201	7	9.98	1.8	5.27E-03	ENSMUST00000055604	protein coding
Zfp541-201	7	0.03	-2.88	0.55	ENSMUST00000108509	protein coding
Zfp541-203	7	0.02	-2.12	0.69	ENSMUST00000210805	protein coding
Zfp560-201	9	1.82	-2.05	0.14	ENSMUST00000068079	protein coding
Zfp560-202	9	5.85	-1.29	0.73	ENSMUST00000143992	protein coding
Zfp574-201	7	22.78	1.18	0.48	ENSMUST00000053410	protein coding
Zfp58-201	13	4.57	-1.7	0.31	ENSMUST00000076123	protein coding
Zfp58-202	13	0.37	1.26	0.89	ENSMUST00000091523	protein coding
Zfp58-203	13	0.05	4.26	NaN	ENSMUST00000163534	protein coding
Zfp58-204	13	2.26	-2.37	0.31	ENSMUST00000171518	protein coding
Zfp580-201	7	11.76	2.17	0.13	ENSMUST00000069324	protein coding
Zfp580-202	7	0.22	-3.11	0.48	ENSMUST00000208570	protein coding
Zfp592-201	7	13.52	1.2	0.12	ENSMUST00000107353	protein coding
Zfp595-201	13	1.16	1.07	0.95	ENSMUST00000109735	protein coding
Zfp595-203	13	0.76	-1.62	0.51	ENSMUST00000168892	protein coding
Zfp595-204	13	0.01	-5.57	NaN	ENSMUST00000169142	nonsense mediated decay
Zfp595-205	13	0.44	-1.32	0.85	ENSMUST00000171466	protein coding
Zfp597-201	16	7.91	1.46	0.06	ENSMUST00000090522	protein coding
Zfp598-201	17	44	-1.35	0.01	ENSMUST00000047179	protein coding
Zfp605-201	5	1.88	-2.22	0.21	ENSMUST00000086686	protein coding
Zfp605-202	5	7.04	-1.87	1.35E-03	ENSMUST00000112528	protein coding
Zfp605-204	5	1.85	1.07	0.95	ENSMUST00000147631	protein coding
Zfp606-201	7	2.12	-1.07	0.91	ENSMUST00000098822	protein coding

Zfp606-202	7	1	2.28	0.28	ENSMUST00000123589	protein coding
Zfp606-204	7	1.84	-1.46	0.43	ENSMUST00000151933	nonsense mediated decay
Zfp608-201	18	0.18	1.12	0.92	ENSMUST00000064763	protein coding
Zfp609-201	9	60.3	1.71	3.66E-17	ENSMUST00000159109	protein coding
Zfp609-203	9	0.16	2.6	0.6	ENSMUST00000160747	protein coding
Zfp612-201	8	1.25	3.29	0.21	ENSMUST00000058804	protein coding
Zfp612-202	8	1.74	-2.07	0.1	ENSMUST00000212754	protein coding
Zfp618-201	4	0.27	3.92	0.32	ENSMUST00000030043	protein coding
Zfp618-202	4	0.34	-1.04	0.98	ENSMUST00000064814	protein coding
Zfp618-203	4	0.61	1.02	0.99	ENSMUST00000107415	protein coding
Zfp626-201	7	0.02	-3.31	NaN	ENSMUST00000080175	protein coding
Zfp626-202	7	8.01	-1.71	0.01	ENSMUST00000205671	protein coding
Zfp629-201	7	0.31	13.51	0.06	ENSMUST00000058038	protein coding
Zfp629-202	7	1.51	1.23	0.73	ENSMUST00000084564	protein coding
Zfp629-204	7	0.05	1.41	0.87	ENSMUST00000128731	protein coding
Zfp629-207	7	0.07	1.38	0.88	ENSMUST00000134446	protein coding
Zfp637-201	6	1.4	-1.52	0.65	ENSMUST00000112858	protein coding
Zfp637-202	6	0.21	13.38	0.28	ENSMUST00000112859	protein coding
Zfp637-203	6	0.26	43.66	0.1	ENSMUST00000112860	protein coding
Zfp637-209	6	0.18	1.5	0.84	ENSMUST00000137224	protein coding
Zfp637-210	6	12.41	-1.66	0.15	ENSMUST00000164472	protein coding
Zfp637-211	6	0.12	1.18	0.94	ENSMUST00000223041	protein coding
Zfp639-201	3	22.09	-1.08	0.81	ENSMUST00000029203	protein coding
Zfp639-202	3	14.28	1.4	0.16	ENSMUST00000191783	protein coding
Zfp639-203	3	5.11	-3.74	0.15	ENSMUST00000192985	protein coding
Zfp639-204	3	0.93	-91.26	0.1	ENSMUST00000193119	protein coding

Zfp639-205	3	3.75	2.12	0.12	ENSMUST00000193287	protein coding
Zfp64-202	2	14.85	1.77	0.18	ENSMUST00000109161	protein coding
Zfp64-204	2	1.47	6.2	0.2	ENSMUST00000124599	protein coding
Zfp646-201	7	25.24	-1.01	0.94	ENSMUST00000050383	protein coding
Zfp646-202	7	5.1	1.1	0.86	ENSMUST00000131000	protein coding
Zfp647-201	15	0.39	-1.42	0.86	ENSMUST00000048854	protein coding
Zfp647-202	15	6.61	-1.41	0.3	ENSMUST00000229055	protein coding
Zfp647-204	15	1.4	-1.26	0.8	ENSMUST00000230677	protein coding
Zfp648-201	1	0.04	7.89	NaN	ENSMUST00000086195	protein coding
Zfp652-201	11	14.33	1.34	0.31	ENSMUST00000091565	protein coding
Zfp652-202	11	16.43	1.23	0.06	ENSMUST00000107717	protein coding
Zfp652-204	11	3.4	1.16	0.74	ENSMUST00000148945	nonsense mediated decay
Zfp653-201	9	2.75	-1.29	0.66	ENSMUST00000043922	protein coding
Zfp653-202	9	2.26	1.2	0.83	ENSMUST00000179605	protein coding
Zfp655-201	5	2.45	-1.83	0.44	ENSMUST00000085661	protein coding
Zfp655-202	5	36.32	-1.28	0.03	ENSMUST00000167316	protein coding
Zfp655-203	5	3.86	1.04	0.97	ENSMUST00000196069	protein coding
Zfp655-204	5	1.38	-1.68	0.68	ENSMUST00000199003	protein coding
Zfp655-205	5	3.66	1.41	0.5	ENSMUST00000199322	protein coding
Zfp661-201	2	4.26	-2.39	0.08	ENSMUST00000077422	protein coding
Zfp661-202	2	0.22	-1.64	0.62	ENSMUST00000110366	protein coding
Zfp661-203	2	4.57	1.13	0.85	ENSMUST00000110368	protein coding
Zfp661-204	2	0.93	-1.69	0.73	ENSMUST00000164551	protein coding
Zfp664-201	5	70.77	1.14	0.06	ENSMUST00000111417	protein coding
Zfp667-202	7	11.15	1.38	0.16	ENSMUST00000108562	protein coding
Zfp667-204	7	0.68	-2.38	0.53	ENSMUST00000153840	protein coding

Zfp668-201	7	0.83	1.46	0.61	ENSMUST00000054415	protein coding
Zfp668-202	7	2.44	-1.72	0.42	ENSMUST00000106261	protein coding
Zfp668-204	7	3.49	-1.01	0.99	ENSMUST00000106263	protein coding
Zfp668-205	7	0.28	-1.98	0.71	ENSMUST00000144721	protein coding
Zfp672-201	11	2.1	1.64	0.41	ENSMUST00000057836	protein coding
Zfp672-202	11	0.15	2.14	0.65	ENSMUST00000064786	protein coding
Zfp672-203	11	1.54	1.85	0.36	ENSMUST00000065533	protein coding
Zfp672-204	11	0.76	3.36	0.09	ENSMUST00000108829	protein coding
Zfp672-207	11	1.15	-2.7	0.29	ENSMUST00000155662	protein coding
Zfp687-201	3	35.79	1.4	3.12E-03	ENSMUST00000019482	protein coding
Zfp687-203	3	0.06	4.26	NaN	ENSMUST00000128438	protein coding
Zfp687-204	3	2.96	1.57	0.62	ENSMUST00000132195	protein coding
Zfp687-208	3	4.77	2.38	0.25	ENSMUST00000149747	protein coding
Zfp688-201	7	8.34	1.59	0.36	ENSMUST00000106300	protein coding
Zfp688-202	7	3.05	-2.59	0.38	ENSMUST00000126756	protein coding
Zfp688-203	7	0.28	19.56	0.21	ENSMUST00000129038	nonsense mediated decay
Zfp689-201	7	3.89	1.3	0.52	ENSMUST00000053392	protein coding
Zfp689-202	7	1.21	1.18	0.87	ENSMUST00000106299	protein coding
Zfp69-201	4	4.53	2.51	0.06	ENSMUST00000106280	protein coding
Zfp69-203	4	1.01	1.76	0.65	ENSMUST00000130702	protein coding
Zfp691-201	4	1.46	-1.18	0.83	ENSMUST00000052715	protein coding
Zfp691-202	4	9.65	1.47	0.21	ENSMUST00000106355	protein coding
Zfp691-204	4	2.43	-1.79	0.43	ENSMUST00000179290	protein coding
Zfp692-201	11	16.58	1.28	0.4	ENSMUST00000049353	protein coding
Zfp692-202	11	7.13	2.08	0.15	ENSMUST00000153510	protein coding
Zfp7-201	15	55.99	-1.29	0.01	ENSMUST00000023179	protein coding

Zfp7-203	15	0.2	1.11	0.95	ENSMUST00000229831	protein coding
Zfp7-204	15	11.79	1.71	0.29	ENSMUST00000229990	protein coding
Zfp704-201	3	9.3	-1.7	1.61E-06	ENSMUST00000041124	protein coding
Zfp704-203	3	9.74	-1.16	0.64	ENSMUST00000193947	protein coding
Zfp706-201	15	77.76	-1.23	3.45E-03	ENSMUST00000078976	protein coding
Zfp706-203	15	6.34	-4.2	0.09	ENSMUST00000226453	protein coding
Zfp706-204	15	141.56	-1.88	1.48E-03	ENSMUST00000226671	protein coding
Zfp706-205	15	3.56	-1.13	0.91	ENSMUST00000228275	protein coding
Zfp729b-201	13	7.89	-1.76	4.91E-03	ENSMUST00000012873	protein coding
Zfp729b-202	13	0.78	-1.01	1	ENSMUST00000138725	protein coding
Zfp738-201	13	0.07	-7.84	NaN	ENSMUST00000110973	protein coding
Zfp738-202	13	0.33	1.42	0.86	ENSMUST00000125495	protein coding
Zfp738-203	13	1.92	-1.84	4.80E-03	ENSMUST00000137496	protein coding
Zfp738-204	13	0.51	-3.58	0.41	ENSMUST00000175678	protein coding
Zfp738-205	13	1.27	-128.96	0.05	ENSMUST00000175821	protein coding
Zfp746-201	6	5.53	-1.59	0.03	ENSMUST00000073124	protein coding
Zfp746-203	6	3.49	1.18	0.78	ENSMUST00000203609	protein coding
Zfp748-202	13	8.23	-1.88	4.80E-04	ENSMUST00000181892	protein coding
Zfp750-201	11	2.38	-1.38	0.56	ENSMUST00000092298	protein coding
Zfp760-201	17	11.05	-1.88	2.48E-03	ENSMUST00000073312	protein coding
Zfp764-201	7	5.39	1.31	0.57	ENSMUST00000059199	protein coding
Zfp770-201	2	17.17	-1.07	0.73	ENSMUST00000050668	protein coding
Zfp772-201	7	5.02	-1.02	0.97	ENSMUST00000074455	protein coding
Zfp777-202	6	13.7	-1.03	0.91	ENSMUST00000114583	protein coding
Zfp777-203	6	1.4	2.14	0.58	ENSMUST00000125385	protein coding
Zfp777-204	6	1.23	-129.55	0.06	ENSMUST00000147281	protein coding

Zfp78-203	7	0.47	1.97	0.38	ENSMUST00000207314	protein coding
Zfp78-204	7	0.33	4.53	0.29	ENSMUST00000207347	protein coding
Zfp78-206	7	0.87	2.9	0.24	ENSMUST00000208390	protein coding
Zfp78-207	7	0.3	7.55	0.2	ENSMUST00000208763	protein coding
Zfp780b-201	7	0.17	-2.4	0.61	ENSMUST00000081618	protein coding
Zfp780b-203	7	3.44	1.27	0.68	ENSMUST00000205874	protein coding
Zfp780b-204	7	7.37	-1.37	0.2	ENSMUST00000206685	protein coding
Zfp800-201	6	0.61	-3.34	0.23	ENSMUST00000035930	protein coding
Zfp800-203	6	4.87	-1	1	ENSMUST00000115321	protein coding
Zfp800-204	6	0.24	65.76	0.05	ENSMUST00000123098	protein coding
Zfp800-205	6	3.25	4.14	0.07	ENSMUST00000155494	protein coding
Zfp804a-201	2	5.03E-03	4.26	NaN	ENSMUST00000047527	protein coding
Zfp82-201	7	6.68	1.87	0.2	ENSMUST00000080834	protein coding
Zfp82-203	7	1.1	-3.76	0.24	ENSMUST00000182546	protein coding
Zfp82-204	7	0.15	-14.76	0.35	ENSMUST00000182919	protein coding
Zfp82-207	7	0.72	1.91	0.46	ENSMUST00000183190	protein coding
Zfp821-201	8	3.91	2.23	0.33	ENSMUST00000034163	protein coding
Zfp821-203	8	2.05	1.44	0.58	ENSMUST00000212000	protein coding
Zfp821-204	8	0.29	-77.07	0.1	ENSMUST00000212192	protein coding
Zfp821-206	8	5.64	1.72	0.32	ENSMUST00000212964	protein coding
Zfp830-201	11	40.04	1.35	0.18	ENSMUST00000056677	protein coding
Zfp831-201	2	0.01	-4.82	0.39	ENSMUST00000059452	protein coding
Zfp85-202	13	2.54	-1.3	0.64	ENSMUST00000144183	protein coding
Zfp853-201	5	0.04	-1.46	0.85	ENSMUST00000180336	protein coding
Zfp853-202	5	0.26	1.03	0.99	ENSMUST00000212355	protein coding
Zfp872-201	9	0.04	7.56	NaN	ENSMUST00000091508	protein coding

Zfp872-202	9	0.13	1.4	0.78	ENSMUST00000178901	protein coding
Zfp879-201	11	0.48	1.2	0.86	ENSMUST00000049625	protein coding
Zfp879-202	11	0.05	-17.51	0.31	ENSMUST00000109133	protein coding
Zfp879-203	11	0.21	-79.06	0.09	ENSMUST00000109134	protein coding
Zfp90-203	8	0.71	13.06	0.07	ENSMUST00000212606	protein coding
Zfp90-205	8	5.12	-1.94	0.18	ENSMUST00000212874	protein coding
Zfp90-206	8	1.24	1.31	0.74	ENSMUST00000213045	nonsense mediated decay
Zfp91-201	19	30.4	-1.11	0.28	ENSMUST00000038627	protein coding
Zfp94-201	7	4.46	-1.41	0.44	ENSMUST00000032673	protein coding
Zfp94-202	7	2.11	-3.71	0.13	ENSMUST00000108436	protein coding
Zfp942-202	17	9.67	1.49	0.08	ENSMUST00000091879	protein coding
Zfp943-201	17	3.79	1.49	0.5	ENSMUST00000055349	protein coding
Zfp943-203	17	0.12	-3.31	0.45	ENSMUST00000174015	nonsense mediated decay
Zfp944-201	17	6.8	-1.22	0.4	ENSMUST00000115535	protein coding
Zfp946-202	17	2.94	-1.28	0.68	ENSMUST00000120222	protein coding
Zfp954-201	7	17.16	1.59	0.04	ENSMUST00000056246	protein coding
Zfp956-201	6	9.24	-1.47	0.12	ENSMUST00000101445	protein coding
Zfp956-202	6	1.14	2.38	0.39	ENSMUST00000140719	nonsense mediated decay
Zfp957-201	14	8.59E-03	4.26	NaN	ENSMUST00000040802	protein coding
Zfp994-201	17	2.08	1.24	0.73	ENSMUST00000179996	protein coding
Zfp995-201	17	4.17	-3.86	0.07	ENSMUST00000106026	protein coding
Zfpm2-201	15	0.06	1.12	0.94	ENSMUST00000053467	protein coding
Zfta-202	19	5.58	-1.56	0.04	ENSMUST00000159348	protein coding
Zfx-202	X	3.36	-1.21	0.82	ENSMUST00000113925	protein coding
Zfx-203	X	11.69	-1.19	0.24	ENSMUST00000113926	protein coding
Zfx-204	X	1.68	1.6	0.71	ENSMUST00000113927	protein coding

Zfx-207	X	0.22	-1.06	0.98	ENSMUST00000137853	protein coding
Zfy1-201	Y	0.05	-23.02	0.26	ENSMUST00000065545	protein coding
Zglp1-201	9	0.18	-1.23	0.92	ENSMUST00000115494	protein coding
Zgpat-203	2	12.99	1.16	0.56	ENSMUST00000116366	nonsense mediated decay
Zgpat-206	2	2.58	-3.29	0.14	ENSMUST00000156258	protein coding
Zhx1-201	15	2.87	-1.77	0.14	ENSMUST00000070143	protein coding
Zhx1-203	15	0.19	-3.58	0.45	ENSMUST00000175805	protein coding
Zhx1-206	15	0.35	-4.05	0.3	ENSMUST00000177276	protein coding
Zik1-201	7	7.95	-1.85	3.86E-03	ENSMUST00000032551	protein coding
Zkscan1-201	5	3.3	1.12	0.71	ENSMUST00000019660	protein coding
Zkscan1-202	5	0.44	-1.04	1	ENSMUST00000066617	protein coding
Zkscan1-204	5	2.37	-1.12	0.84	ENSMUST00000110963	protein coding
Zkscan14-201	5	6.44	1.25	0.65	ENSMUST00000031632	protein coding
Zkscan14-202	5	0.77	-99.35	0.09	ENSMUST00000162220	protein coding
Zkscan14-203	5	0.45	1.03	0.98	ENSMUST00000198959	protein coding
Zkscan3-201	13	6.54	1.84	0.19	ENSMUST00000070785	protein coding
Zkscan3-202	13	0.31	3	0.46	ENSMUST00000116433	protein coding
Zkscan3-203	13	2.52	-1.53	0.53	ENSMUST00000116434	protein coding
Zkscan3-204	13	3.67	1.35	0.21	ENSMUST00000117721	protein coding
Zkscan3-208	13	0.53	1.14	0.9	ENSMUST00000224820	protein coding
Zkscan5-202	5	20.79	-1.51	2.92E-03	ENSMUST00000085671	protein coding
Zkscan5-204	5	0.37	-1.46	0.84	ENSMUST00000161881	protein coding
Zkscan5-205	5	0.15	27.33	0.14	ENSMUST00000161896	nonsense mediated decay
Zkscan5-206	5	0.11	2.02	0.7	ENSMUST00000162168	nonsense mediated decay
Zkscan6-202	11	10.24	1.34	0.22	ENSMUST00000071465	protein coding
Zkscan8-202	13	0.16	1.78	0.75	ENSMUST00000110481	protein coding

Zmat1-201	X	0.06	27.8	0.16	ENSMUST00000064659	protein coding
Zmat1-202	X	0.18	-2.93	0.44	ENSMUST00000113185	protein coding
Zscan12-201	13	5.53	1.06	0.91	ENSMUST00000053293	protein coding
Zscan12-203	13	2.31	-1.73	0.31	ENSMUST00000225545	protein coding
Zscan20-202	4	4.47	-1.2	0.5	ENSMUST00000097877	protein coding
Zscan20-203	4	1.34	-1.02	0.99	ENSMUST00000135309	nonsense mediated decay
Zscan20-204	4	0.72	-2.7	0.47	ENSMUST00000141040	protein coding
Zscan20-205	4	0.08	-1.26	NaN	ENSMUST00000147876	protein coding
Zscan21-202	5	5.05	1.78	0.51	ENSMUST00000080732	protein coding
Zscan21-203	5	0.28	1.36	0.83	ENSMUST00000110959	protein coding
Zscan21-206	5	0.7	-6.52	0.2	ENSMUST00000136425	protein coding
Zscan21-207	5	0.25	-20.37	0.29	ENSMUST00000142185	protein coding
Zscan22-201	7	2.83	-1.46	0.45	ENSMUST00000055528	protein coding
Zscan22-202	7	7.38	-1.03	0.93	ENSMUST00000117189	protein coding
Zscan22-203	7	1.89	-1.33	0.8	ENSMUST00000119989	protein coding
Zscan25-201	5	11.5	-1.1	0.73	ENSMUST00000094116	protein coding
Zscan25-202	5	2.4	-1.84	0.57	ENSMUST00000199563	nonsense mediated decay
Zscan25-203	5	1.82	7.45	0.1	ENSMUST00000200246	protein coding
Zscan26-202	13	21.22	2	2.07E-06	ENSMUST00000110485	protein coding
Zscan29-202	2	4.73	1.58	0.03	ENSMUST00000110661	protein coding
Zscan29-203	2	5.54	1.35	0.37	ENSMUST00000146243	protein coding
Zxdc-203	6	2.31	2.21	0.09	ENSMUST00000113539	protein coding
Zxdc-205	6	3.69	1.42	0.64	ENSMUST00000203493	protein coding
Zzz3-201	3	23.11	-1.34	0.04	ENSMUST00000089982	protein coding
